# Base‐Promoted Homolytic Aromatic Substitution (BHAS) Reactions and Hydrodehalogenations Driven by Green Light and an Iron(III)‐NHC Photoredox Catalyst

**DOI:** 10.1002/chem.202500409

**Published:** 2025-03-12

**Authors:** Lisa H. M. de Groot, Clara García‐Mateos, Catherine E. Johnson, Valtýr Freyr Hlynsson, Alpesh K. Sharma, Reiner Lomoth, Kenneth Wärnmark

**Affiliations:** ^1^ Centre for Analysis and Synthesis (CAS) Department of Chemistry Lund University SE-22100 Lund Sweden; ^2^ Department of Chemistry – Ångström Laboratory Uppsala University SE-75120 Uppsala Sweden

**Keywords:** Iron, NHC ligand, Photoredox catalysis, Green light, BHAS

## Abstract

An Fe(III)‐NHC complex has been employed for the green light driven catalysis of base‐promoted homolytic aromatic substitution (BHAS) reactions. Tributylamine was used as a sacrificial electron donor, together with potassium carbonate as base in dimethyl sulfoxide as solvent. In contrast to previously studied photocatalysts, the excited Fe(III)‐NHC complex is not reducing the arylhalide substrates. Instead, the latter are activated by α‐aminoalkyl radicals formed upon reductive quenching of the photocatalyst by tributylamine. Avoiding strongly reducing photocatalysts as well as strong base, these mild reaction conditions allowed for the expansion of the substrate scope to accommodate also aldehyde and ester substituents. 100 % conversion was obtained after 48 h of irradiation. In this way a wide variety of cyclized products and their corresponding hydrodehalogenated products were obtained as isolated and pure compounds, in the vast majority of cases.

## Graphical abstract

Herein we present the application of an Fe(III)‐NHC complex in the intramolecular base‐promoted homolytic aromatic substitution reaction. Milder reaction conditions than previously reported were employed, which tolerated functional groups like an aldehyde and ester. The reaction leads to the predominant formation of the cyclized product (**CP**) over the hydrodehalogenated product (**HP**). A mechanism is proposed, supported by extensive mechanistic studies.

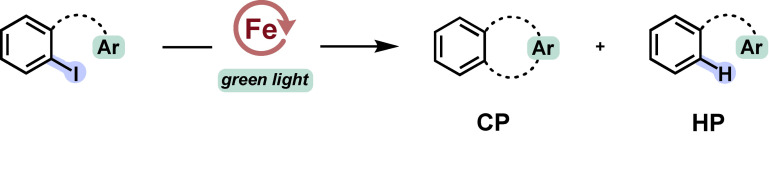

## Introduction

**Figure 1:**
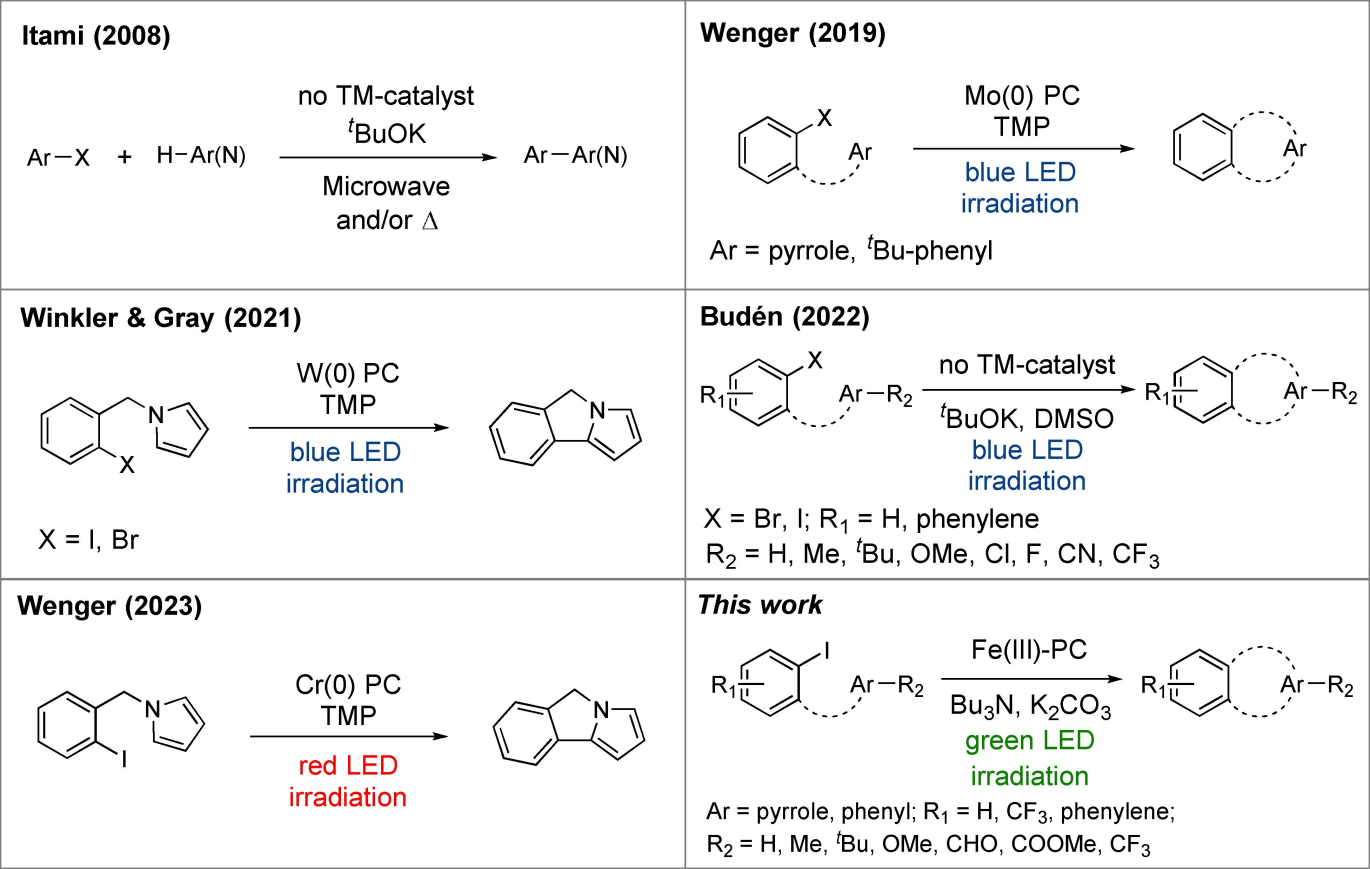
Overview of the different methods of driving BHAS reactions.

Base‐promoted homolytic aromatic substitution (BHAS) reactions have been established as a facile method for C−C bond formation between two aromatic moieties.^[1]^ This reaction type is, as the name implies, promoted by a base, traditionally potassium *tert*‐butoxide (^*t*^BuOK), in combination with harsh reaction conditions, such as heating or microwave irradiation (Figure 1, left top).[^2^, ^3^, ^4^, ^5^, ^6^, ^7^] Efforts have been made to render the reaction conditions more benign with help of photoredox catalysis or photoinitiation.[^8^, ^9^, ^10^, ^11^, ^12^] In a recent report, Budén and co‐workers demonstrated the synthesis of a range of 6*H*‐benzo[*c*]chromenes from iodo‐ or bromo‐substituted substrates in presence of ^*t*^BuOK in dimethyl sulfoxide (DMSO) by utilizing blue light irradiation instead of extensive heating or microwave irradiation (Figure 1, right middle).^[13]^

To further improve the reaction conditions, the use of the harsh ^*t*^BuOK base could be circumvented by for example introducing molybdenum‐ (Figure 1, right top) or tungsten‐based (Figure 1, left middle) transition metal (TM) photoredox catalysts (PCs), in both cases employing 2,2,6,6‐tetramethylpiperidine (TMP) as a base and blue light irradiation.[^14^, ^15^] Another strategy was recently employed by Wenger and co‐workers, in which a Cr(0) isocyanide complex was used to catalyse the BHAS reaction under irradiation with red (λ=623 nm, 3.8 W) light in combination with a potent reductant (tetrakis(dimethylamino)ethylene) for regeneration of the photocatalyst (Figure 1, left bottom).^[16]^ These PCs are characterized by very strongly reducing excited states (ESs) (−2.2–−2.9 V vs Fc^+/0^) able to efficiently induce the initial reductive dehalogenation of the arylhalide substrates with significant driving force for the photoinduced electron transfer. The use of these exceptionally strong photoreductants might however interfere with a broader substrate scope including e. g. arylhalides with ester and aldehyde functional groups that are potentially reduced under such conditions, with reduction potentials estimated between −2.7–−2.3 V vs Fc^+/0^ (see ESI).

In the search of PCs that exhibit more moderate reduction potentials, iron‐based PCs could be of interest. The field of photoredox catalysis based on iron complexes has been expanding over the last decade, mainly because of iron being an Earth‐abundant element, and increasingly more applications of such complexes are being discovered.[^17^, ^18^]

**Figure 2:**
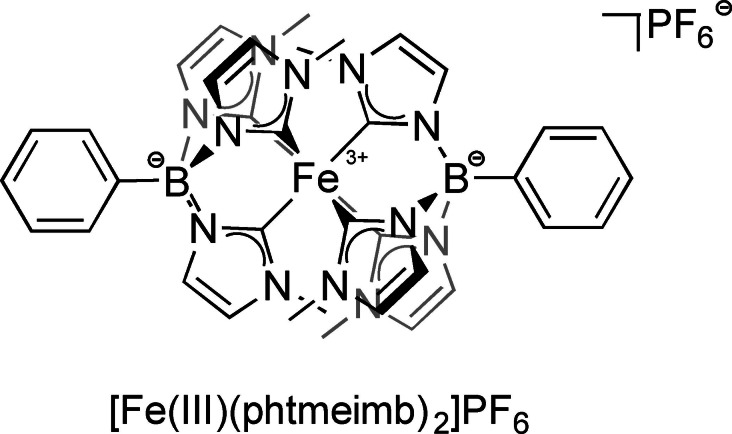
Structure of the Fe‐NHC complex [Fe(III)(phtmeimb)_2_]PF_6_.

In this report we demonstrate the application of [Fe(III)(phtmeimb)_2_]PF_6_ (phtmeimb = phenyltris(3‐methyl‐imidazolin‐2‐ylidene)borate) (Figure 2) as PC in the green light‐driven photoredox catalytic intramolecular BHAS reaction of halogenated substrates using potassium carbonate (K_2_CO_3_) as base and tributylamine (Bu_3_N) as a sacrificial electron donor (SED) in DMSO‐*d*_6_ as solvent. Featuring a wide variety of substrates, the corresponding desired cyclized product (**CP**) was obtained as the main product, as well as a hydrodehalogenated product (also known as hydrogen addition product, **HP**) as side‐product. Our mechanistic studies show that the reaction is initiated by reductive quenching of [Fe(III)(phtmeimb)_2_]PF_6_ with tributylamine (Bu_3_N) that generates α‐aminoalkyl radicals presumably responsible for the activation of the arylhalide substrates via single electron transfer (SET) or halogen atom transfer (XAT). The milder reaction conditions allow for the presence of ester and aldehyde functional groups on the substrates, which have not been reported for this type of reaction to date.

## Results and Discussion

### BHAS Reaction Optimizations

Following the previous findings on BHAS reactions driven by TM‐complexes based on tungsten and molybdenum, various reaction conditions were tested on iodo‐containing substrates.[^14^, ^15^] When the reaction conditions that were previously found for the W‐ and Mo‐based PCs were applied to the halogenated substrates with the [Fe(III)(phtmeimb)_2_]PF_6_ PC together with Bu_3_N as SED, a hydrodehalogenated side‐product (**HP**) was observed in combination with the desired **CP**. However, it is the **CP** that is of main interest. For example, the 6*H*‐benzo[*c*]chromene^[19]^ and pyrrolo[2,1‐*a*]isoquinoline^[20]^ moieties, which can be produced using the BHAS reaction,[^13^, ^14^, ^15^, ^16^] can be found in pharmaceutical and natural products. The formation of **HP** in combination with **CP** is a known challenge of systems employing amine‐based SEDs (single‐electron donors).^[21]^ Upon further exploration of the reaction system, it was found that the choice of added base, SED and solvent play a major role in the obtained product distribution between **CP** and **HP**. Although the **HP** is not of main interest in this study, it is noted that previously various protocols for the dehalogenation of the compound class corresponding to the substrates used in the BHAS reaction have been established, due to the importance of this transformation.[^22^, ^23^, ^24^]

The reaction conditions were optimized with the aim of favouring the formation of the **CP** over the **HP**. Table 1 presents an overview of the initial optimization and control reactions of the BHAS reaction of substrate **1**. Full conversion of substrate **1** (50 mM in DMSO) was observed when employing Bu_3_N and TMP (2 equiv. each) under 48 h of green LED (λ=530, 3.03 W/slot) irradiation under argon atmosphere (entry 2), compared to an 82 % conversion after 24 h (entry 1). Other Fe‐based TM‐complexes did not show any substrate conversion (entry 3–5) under these conditions whereas the [Ru(bpy)_3_]Cl_2_ PC showed significantly less conversion (entry 6) after a 24 hour reaction time. In the absence of PC, SED/base or light, no conversion was observed (entries 7–9). When the previously established reaction conditions with Bu_3_N and TMP were applied to the reaction with substrate **2**, 96 % instead of 100 % of the starting material was converted to **CP** and **HP** (Table 2, entry 1). The product ratio between **CP‐2** and **HP‐2** was 53 : 43, less favourable with respect to **CP** than the observed product ratio for substrate **1** (75 : 25). Since substrate **2** and its corresponding products exhibit superior stability compared to substrate **1**, continued optimizations were performed using substrate **2** instead of substrate **1**. When TMP was replaced with K_2_CO_3_, and the amount of Bu_3_N was reduced from 2 to 1 equiv, full conversion was obtained after 48 h of irradiation, and an improved 74 : 26 **CP**–**HP** product ratio was observed for substrate **2** (Table 2, entry 2). Applying the improved Bu_3_N−K_2_CO_3_ (1 : 2 equiv) dual base‐system to substrate **1** also improved the product ratio for that reaction from 75 : 25 to 83 : 17 **CP‐1**–**HP‐1** (Table 1, entry 10). Control reactions with the new base system (Table 1, entries 11–13) and longer reaction time had a similar outcome and did not outperform the reaction conditions in entry 10.

**Table 1:** Optimization and control reactions of the BHAS reaction using 50 mM substrate **1** in DMSO‐*d*_6_ and 2 mol % PC under green irradiation and inert atmosphere.

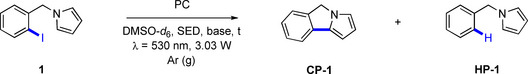					
Entry	PC	SED‐Base	Reaction time	Conversion (%)^[a]^	**CP**:**HP** ratio^[a]^
1	[Fe(phtmeimb)_2_]PF_6_	Bu_3_N−TMP (2 : 2 equiv)	24 h	82	79 : 21
2	[Fe(phtmeimb)_2_]PF_6_	Bu_3_N−TMP (2 : 2 equiv)	48 h	100	75 : 25
3	[Fe(btz)_3_](PF_6_)_3_	Bu_3_N−TMP (2 : 2 equiv)	24 h	–	–
4	FeBr_2_	Bu_3_N−TMP (2 : 2 equiv)	24 h	–	–
5	[Fe(bpy)_3_](PF_6_)_2_	Bu_3_N−TMP (2 : 2 equiv)	24 h	–	–
6	[Ru(bpy)_3_]Cl_2_	Bu_3_N−TMP (2 : 2 equiv)	24 h	54	59 : 41
7	–	Bu_3_N−TMP (2 : 2 equiv)	24 h	–	–
8	[Fe(phtmeimb)_2_]PF_6_	–	24 h	–	–
9	[Fe(phtmeimb)_2_]PF_6_	Bu_3_N−TMP (2 : 2 equiv)	24 h^[b]^	–	–
10	[Fe(phtmeimb)_2_]PF_6_	Bu_3_N−K_2_CO_3_ (1 : 2 equiv)	48 h	100	83 : 17
11	–	Bu_3_N−K_2_CO_3_ (1 : 2 equiv)	48 h	–	–
12	[Fe(btz)_3_](PF_6_)_3_	Bu_3_N−K_2_CO_3_ (1 : 2 equiv)	48 h	–	–
13	[Ru(bpy)_3_]Cl_2_	Bu_3_N−K_2_CO_3_ (1 : 2 equiv)	48 h	50	70 : 30

[a] ^1^H NMR yield determined from the crude reaction mixture. [b] No irradiation.

**Table 2:** Optimization and control reactions of the BHAS reaction using 50 mM substrate **2** in DMSO‐*d*_6_ and 2 mol % PC under green irradiation and inert atmosphere.

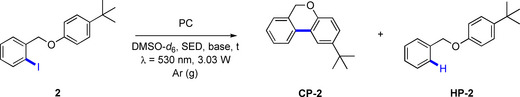					
Entry	PC	SED‐Base	Reaction time	Conversion (%)^[a]^	**CP**:**HP** ratio^[a]^
1	[Fe(phtmeimb)_2_]PF_6_	Bu_3_N−TMP (2 : 2 equiv)	48 h	96	55 : 45
2	[Fe(phtmeimb)_2_]PF_6_	Bu_3_N−K_2_CO_3_ (1 : 2 equiv)	48 h	100	74 : 26
3	[Fe(phtmeimb)_2_]PF_6_	Bu_3_N−K_2_CO_3_ (1 : 2 equiv)	22 h	21	67 : 33
4	–	Bu_3_N−K_2_CO_3_ (1 : 2 equiv)	48 h	–	–
5	[Fe(phtmeimb)_2_]PF_6_	Bu_3_N−K_2_CO_3_ (1 : 2 equiv)	48 h^[b]^	–	–
6	[Fe(phtmeimb)_2_]PF_6_	‐	48 h	–	–
7	[Fe(phtmeimb)_2_]PF_6_	K_2_CO_3_ (2 equiv)	48 h	–	–
8	[Fe(phtmeimb)_2_]PF_6_	Bu_3_N (1 equiv)	48 h	29	72 : 28
9	[Fe(phtmeimb)_2_]PF_6_	Bu_3_N (3 equiv)	48 h	91	63 : 37
10	[Fe(btz)_3_](PF_6_)_3_	Bu_3_N−K_2_CO_3_ (1 : 2 equiv)	48 h	–	–
11	FeBr_2_	Bu_3_N−K_2_CO_3_ (1 : 2 equiv)	48 h	–	‐
12	[Fe(bpy)_3_](PF_6_)_2_	Bu_3_N−K_2_CO_3_ (1 : 2 equiv)	48 h	–	–
13	[Ru(bpy)_3_]Cl_2_	Bu_3_N−K_2_CO_3_ (1 : 2 equiv)	48 h	54	69 : 31
14	[Fe(phtmeimb)_2_]PF_6_	Bu_3_N−K_2_CO_3_ (1 : 2 equiv)	48 h^[c]^	20	35 : 65

[a] ^1^H NMR yield determined from the crude reaction mixture. [b] No irradiation. [c] 3 equiv. of TEMPO added.

Control reactions were also performed for substrate **2**, employing the newly found conditions (Table 2). In absence of either the [Fe(phtmeimb)_2_]PF_6_ PC or irradiation, no conversion of starting material was observed (Table 2, entry 4 and 5). Furthermore, removal of the bases altogether, or performing the reaction in presence of only K_2_CO_3_ also completely hampered the reaction (Table 2, entry 6 and 7). In presence of 1 equiv. of Bu_3_N (without K_2_CO_3_), 29 % conversion was noted, which was increased to 91 % upon addition of 3 equiv. of Bu_3_N (without K_2_CO_3_) (Table 2, entries 8 and 9). However, as expected, a worse **CP**–**HP** ratio was obtained. Addition of other iron salts or iron complexes as PCs did not result in any conversion of the starting material (Table 2, entries 10–12). Employing the archetype [Ru(bpy)_3_]Cl_2_ complex as PC resulted in 54 % conversion of the substrate (Table 2, entry 13). This lower conversion can partially be attributed to the poor absorption of this PC at the employed wavelength, as well as to its relatively lower photostability. Addition of 3 equiv. of TEMPO (2,2,6,6‐tetramethylpiperidinyloxyl) as a radical trap severely reduced the reactivity, as only 20 % conversion was observed (Table 2, entry 14), indicating a radical mechanism. It was also found that the addition of formic acid as a proton source drives the product ratio to almost exclusively favour the **HP** (Table S9).^[25]^ Further optimizations can be found in the Supporting Information.

### Substrate Scope

Having found the optimized reaction conditions for substrates **1** and **2**, the substrate scope (Table 3) was established. Table 3 shows a summary of the results. The substrates were chosen to lead to synthetically valuable cyclized products upon application of the reaction conditions. Due to the C−I bond being more easily reductively cleaved than the C−Br bond,^[26]^ *o*‐substituted iodobenzyl groups were employed. Furthermore, the phenyl moiety is substituted in the *para* position, as this was shown in previous investigations to have the most pronounced effect on the reactivity.^[13]^ The substrate scope includes a variety of electron donating and electron withdrawing functional groups on the phenyl moiety (substrate **2**–**5**, **7**–**9**), to probe the electronic effect of such substituents on the reaction yield. Among these are an aldehyde and ester‐substituted substrate (**7** and **8**), which are commonly sensitive to the traditional BHAS reaction conditions. The effect of the exchange of the phenoxy moiety for heterocyclic aromatic ring systems such as a pyrrole (**1**) on BHAS reactivity was also investigated. Other substrates featured the inverted connection of the benzyloxy bridge (**6**), a bromo‐substituted substrate (**10**) and a bromonaphthalene‐containing substrate (**11**). Lastly, the effect of introducing electron withdrawing (**12**) groups on the iodophenyl ring was probed.

**Table 3:** Conversions, product ratios and yields for the BHAS reaction on the different substrates.

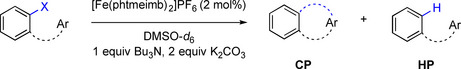					
Entry	Substrate	Conversion (%)	Crude yield (%)^[a]^, isolated yield (%))		**CP**:**HP** ratio
			CP	HP	
1	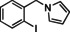	100	77, 59	16, 11	83 : 17
	**1**				
2	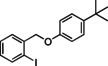	100	53, 46	19, 23	74 : 26
	**2**				
3	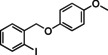	100	58, 35	13, 8	82 : 18
	**3**				
4	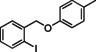	100	52, 26	21, 9	71 : 29
	**4**				
5	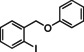	100	55, 30	26, 6	68 : 32
	**5**				
6	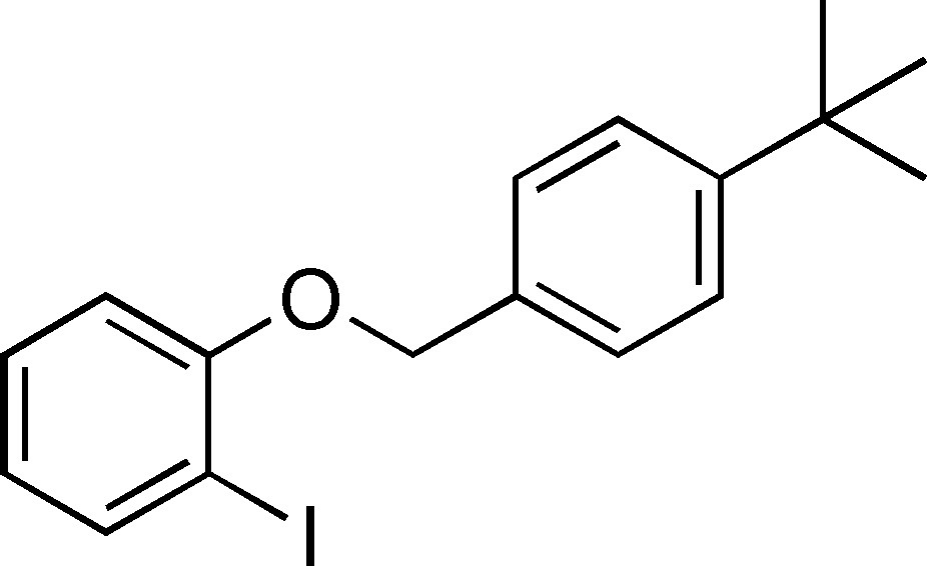	100^[b]^	**CP‐6A**: 34, 19 **CP‐6B**: 8, 3	2, n.d.	77 : 19 : 4^[c]^
	**6**				
7	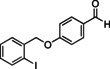	97	34, n.d.	22, n.d.	58 : 39
	**7**				
8	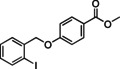	100	44, n.d.	26, n.d.	63 : 37
	**8**				
9	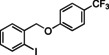	100	66, 11	10, 8	68 : 32
	**9**				
10	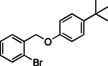	0	–	–	–
	**10**				
11	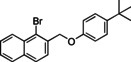	100	34, 23	5, 3	44 : 7 : 49^[d]^
	**11**				
12	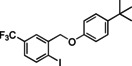	100	68, 47	4, 10	93 : 7
	**12**				

[a] Determined from by ^1^H NMR using 1,3,5‐trimethoxybenzene as internal standard (IS). n. d.=not determined. [b] 140 h [c] **CP‐6A**:**CP‐6B**:**HP‐6** [d] **CP‐11**:**HP‐11**:**AP‐11**

**Figure 3:**
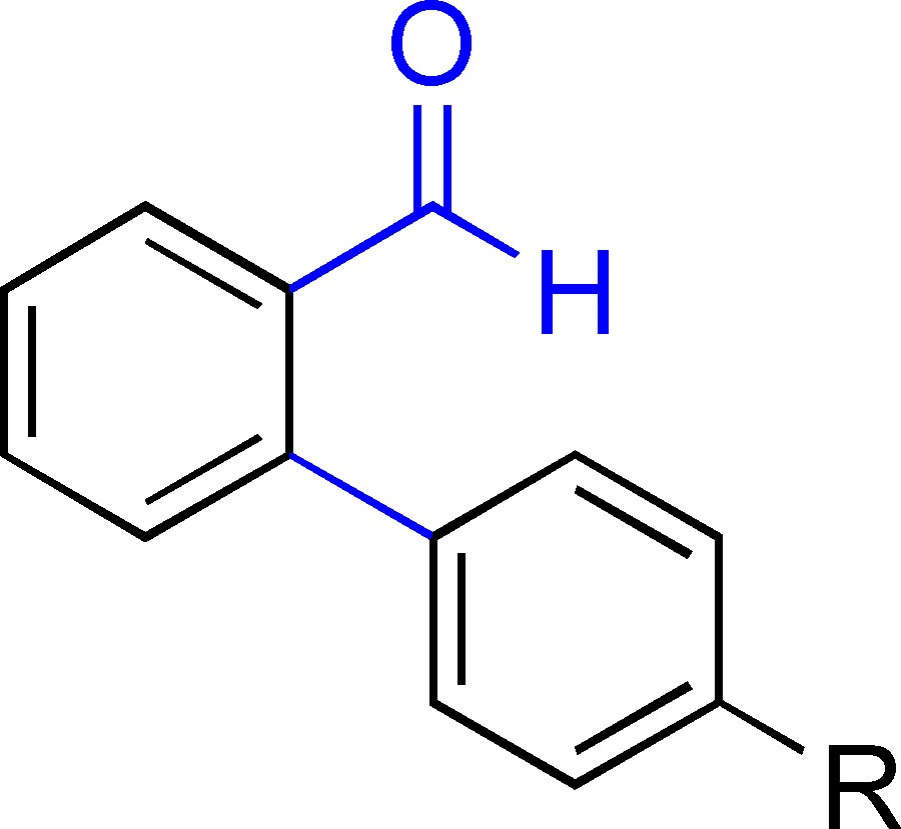
General structure of the aldehyde side‐product (**AP**). R=electron donating substituent or H.

Full conversion was observed for almost all the substrates, as determined by ^1^H NMR analysis of the crude reaction mixtures (Table 3). Although the **CP** and **HP** are found as the major product and side‐product, respectively, it is noteworthy that the substrate is not exclusively converted to the corresponding **CP** and **HP**. Traces of other aromatic compounds are present in the ^1^H NMR spectra, and for some substrates, an aldehyde‐containing product (**AP**, Figure 3) could be observed as well. Isolating the product mixtures by traditional silica column chromatography proved to be challenging as the **CP** and **HP** exhibit similar polarities. Nonetheless, analytically pure products could be isolated in the vast majority of cases, after a workup of the reaction mixtures followed by gravitational silica column chromatography (petroleum ether (PE)), with further purification employing either a Sephadex LH‐20 column (1×100 cm, methanol), or preparative reverse phase high‐performance liquid chromatography (HPLC, acetonitrile‐water). See Supporting Information for details.

It was found that electron donating substituents on the phenyl ring (**2**–**4**) positively influence the BHAS reactivity, compared to its non‐substituted counterpart **5**. Electron withdrawing groups negatively impact the reactivity, which led to either a lower yield, lower **CP**–**HP** ratio, or warranted the necessity for longer irradiation times (**7**–**9**). Inverting the connectivity of the benzyloxy bridge also negatively impacted the reactivity (**6**), which needed a significantly longer reaction time (140 h) to achieve full conversion. A mixture of regioisomers were obtained in this case (**CP‐6A** and **CP‐6B**), and only traces of **HP**. This is likely because of the influence of the electron‐withdrawing group that activates the phenyl ring towards cyclization and competition between the 5‐*endo*‐trig and the 6‐*exo*‐trig pathways, which resulted in two cyclized products that were separated by preparative HPLC.^[13]^

The presence of an aldehyde and an ester functionality (substrates **7** and **8**) in the phenyl ring was tolerated under these conditions, although unidentified products were also observed in the crude. Separation of **CP** and **HP** was in these cases even more challenging than for the previously mentioned substrates, but the formation of both products was confirmed for the aldehyde‐substituted substrate (**7**). The cyclized product was also confirmed by ^1^H NMR of the ester‐substituted substrate (**8**) in one chromatographic fraction (see SI). However, **HP** was in this case not successfully isolated and could thus not be irrefutably confirmed by ^1^H NMR analysis. The *para*‐trifluoromethyl‐substituted substrate (**9**) achieved full conversion and both products were isolated by preparative HPLC. The bromo‐substituted substrate (**10**) was, as expected due to the comparatively less reactive C−Br bond, not reactive under these reaction conditions. On the other hand, the Br‐naphthalene containing substrate (**11**) was reactive enough leading to full conversion after 48 h. However, the crude product mixture only showed limited presence of the expected **CP** and **HP**, and instead a variety of different aromatic products was observed based on ^1^H NMR, among which an aldehyde side‐product (18 % isolated yield) was identified by spectroscopic techniques (Figure 3). A proposed mechanism for the formation of this **AP** is shown in Figure S21. This was in line with previous findings on such products that could be formed following different cyclization pathways under BHAS conditions.^[13]^ The presence of such product compelled a more in‐dept investigation of the ^1^H NMR spectra of the other reaction crudes. This resulted in the discovery of traces of aldehyde products for the substrates featuring electron‐donating substituents **2**, **3**, and **4**, as well as for the non‐substituted substrate **5**.

Finally, the presence of an electron withdrawing group on the iodophenyl ring (substrate **12**) positively impacted the BHAS reaction towards the formation of **CP**.

### Mechanistic Studies

Electrochemical data (see Supporting Information) indicates that the potentials required for reduction of the substrates used in this study are close to the potential reported for the iodobenzene moiety (approx. −2.3 V vs Fc^+/0^).^[27]^ The ES of [Fe(phtmeimb)_2_]PF_6_ (Fe(IV/III*)=−1.88 V vs Fc^+/0^)^[28]^ is therefore not expected to reduce this type of substrate. Luminescence quenching data (see Supporting Information) revealed however some reactivity of the ES towards **1** and **6** while no quenching was observed with **2**. These substrates were chosen due to their differences in scaffold (phenyl (**2**) vs pyrrole (**1**) moiety) and observed reactivity (substrate **6** reacted slower compared to the majority of other substrates). Electrochemical data (ESI) suggests that the quenching reaction might be attributed to electron transfer resulting in the oxidation of the pyrrole or alkoxyphenyl moiety, respectively. This notion was corroborated by the observed quenching with pyrroles (ESI Figures S10 and S11, Table 4) while expectedly little, if any, quenching was observed with iodobenzene (Figure S12). In case of substrate **1** (Figure S7 and S20) and the pyrrole quenchers (Figures S15 and S16), flash photolysis data further support reductive quenching of the PC. However, the unexpected reductive quenching by some of the substrates is apparently not involved in product formation as successful transformations relied on the addition of an electron donor in form of trialkylamines.

**Table 4:** Excited state quenching data and cage escape yields for [Fe(phtmeimb)_2_]^+^ (Τ_0_=excited state lifetime, *K*_SV_=Stern‐Volmer constant, *k*_q_=bimolecular rate constant, *η*_q_=quenching yield, *η*_ce_=cage escape yield) in different solvents. See SI for detailed calculations of CEY.

	Τ_0_=1.43 ns^[a]^			
	*K*_SV_/M^−1^	*k*_q_/10^9^ M^−1^s^−1^	*η*_q_ ([Q]/mM)	*η*_ce_
Substrate **1**	0.67^[b]^	0.47	0.07 (50)	0.03
Substrate **2**	0^[c]^	0^[c]^	n.a.^[d]^ (50)	n.a.
Substrate **6**	0.16	0.11	n.a. (50)	n.a.
Iodobenzene	0.01	0.01	n.a. (50)	n.a.
*N*‐Methylpyrrole	2.83	1.95	0.09 (50)	0.02
*N*‐Benzylpyrrole	1.43	0.99	0.07 (50)	0.02
Bu_3_N	0.67	0.44	0.10 (50)	<0.04^[e]^
Et_3_N	3.74	2.58	0.19 (50)	0.02
TMP	2.58	1.78	0.22 (100)	0.02

	Τ_0_=2 ns^[f]^			
Bu_3_N	18.03	9.20	0.45 (50)	0.02
Et_3_N	15.24	7.78	0.49 (50)	0.02
TMP	4.09	2.09	0.32 (100)	0.02

[a] In DMSO. [b] From steady state emission quenching. [c] No observable quenching. [d] Not applicable. [e] Upper limit (no obvious observable signal within detector limits, hence assume any available signal was less than the lowest observable signal, 0.0010 mOD). [f] In acetonitrile.

As the reduced PC is clearly not sufficiently reducing (*E*°(III/II)=−1.16 V vs Fc^+/0^)^[28]^ to activate the aryliodide substrates, the role of the amine can be attributed to the formation of α‐aminoalkyl radicals via deprotonation of the amine cation radical. While α‐aminoalkyls are rather strong reductants (−2.1 V vs Fc^+/0^),[^29^, ^30^] activation of the aryliodides via SET might not be feasible and the dehalogenation step could primarily rely on the established XAT reactivity of these radicals.[^31^, ^32^] With potentials around 0.4 V required for their oxidation, trialkylamines have been shown to efficiently quench the ES of [Fe(phtmeimb)_2_]PF_6_ (*E*°(*III/II)=0.97 V vs Fc^+/0^).^[28]^ Also Bu_3_N was found to quench the ES of [Fe(phtmeimb)_2_]PF_6_ (Figure S13 and S14) even if the reaction in DMSO was significantly slower than for e. g. Et_3_N (Table 4). As flash photolysis experiments failed to observe the quenching products within detection limits, we can estimate an upper limit of the cage escape yield of around 4 % (see Supporting Information) which is at the lower end of the range of values typically found for the reductive quenching of this PC by trialkylamines.

**Figure 4:**
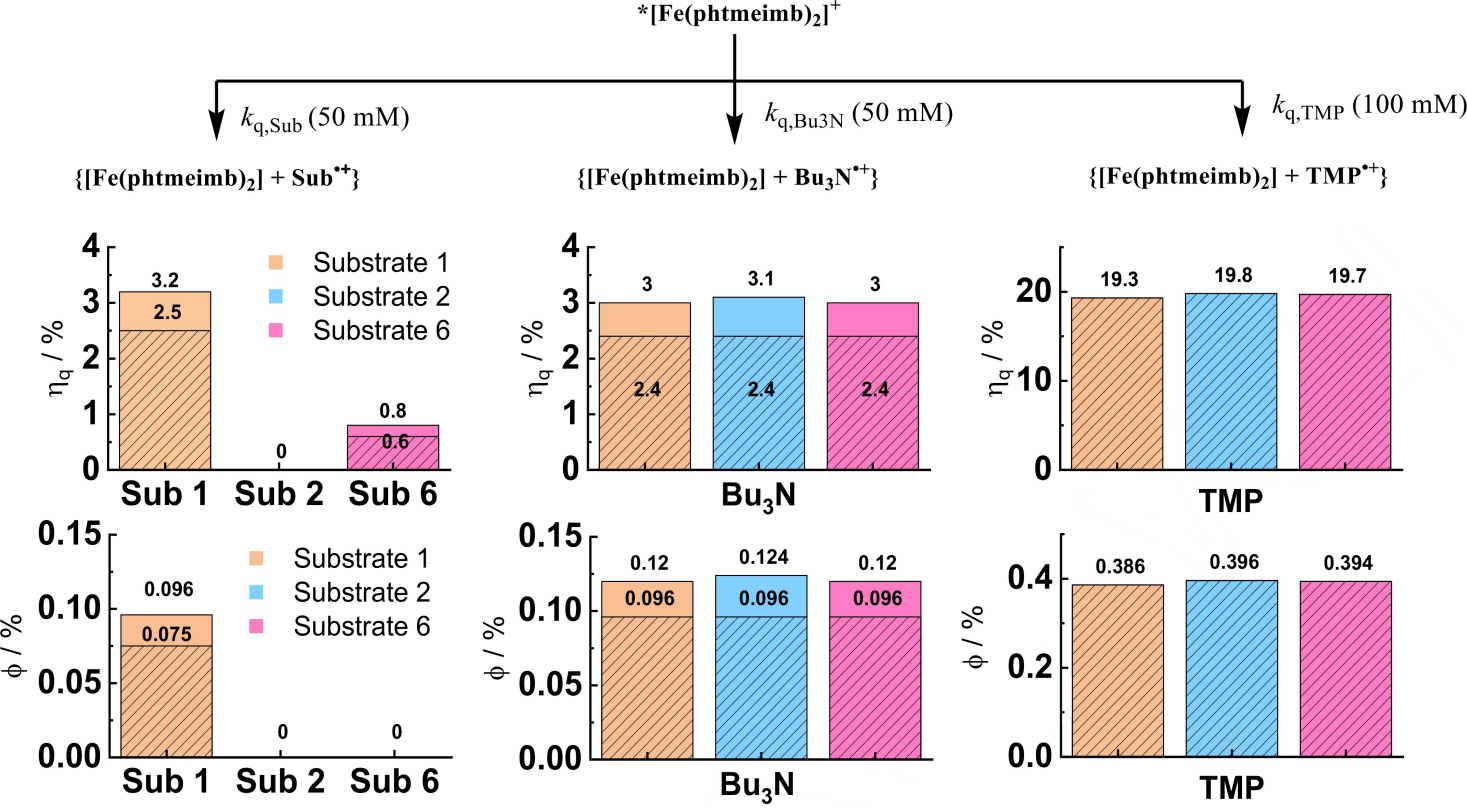
Quenching yields (*η*_q_) and quantum yields (Φ) of charge‐separated products of the competing quenching pathways. The unshaded bars represent quenching by substrate **1**, **2** or **6** in combination with Bu_3_N. The shaded bars represent quenching by substrate **1**, **2** or **6** in combination with Bu_3_N and TMP. See Supporting Information for details.

While the amine cannot only serve as electron donor but also as base, optimized conditions included the addition of base in form of TMP or carbonate. The former can be expected to have a dual role as base and electron donor and fluorescence quenching and flash photolysis data confirmed rapid quenching due to reductive electron transfer. Optimal results in the photoredox catalysis (PRC) studies were however obtained with the carbonate base and the quenching by TMP is hence probably not a productive quenching channel. To quantify the branching into the competing quenching channels under the conditions of the PRC experiments, their respective yields have been calculated from the bimolecular rate constants and the concentrations employed. The results are summarized in Figure 4 that compares data for three different substrates in combination with only Bu_3_N (additional base is carbonate) or in combination with Bu_3_N and TMP. Under the former conditions, about 3 % of the excited PS undergo reductive quenching by the Bu_3_N electron donor. This value is nearly independent of the very variable extent of competing reductive quenching by the different substrates that reaches comparable yield in case of **1**. Addition of TMP provides the most efficient quenching channel with yields approaching 20 %, yet disfavouring the slower quenching by Bu_3_N only moderately. Due to the inefficient quenching and low cage escape yield, the quantum yield of primary electron transfer products formed by Bu_3_N will not exceed values of about 0.1 % even under conditions resulting in minimal competition. Estimates of the overall quantum yield for the product formation (0.3–0.4 %, Table S13) actually exceed those for the electron transfer products formed via quenching with Bu_3_N. This result points to a chain propagation mechanism triggered by the photoinduced oxidation of Bu_3_N.

**Scheme 1:**
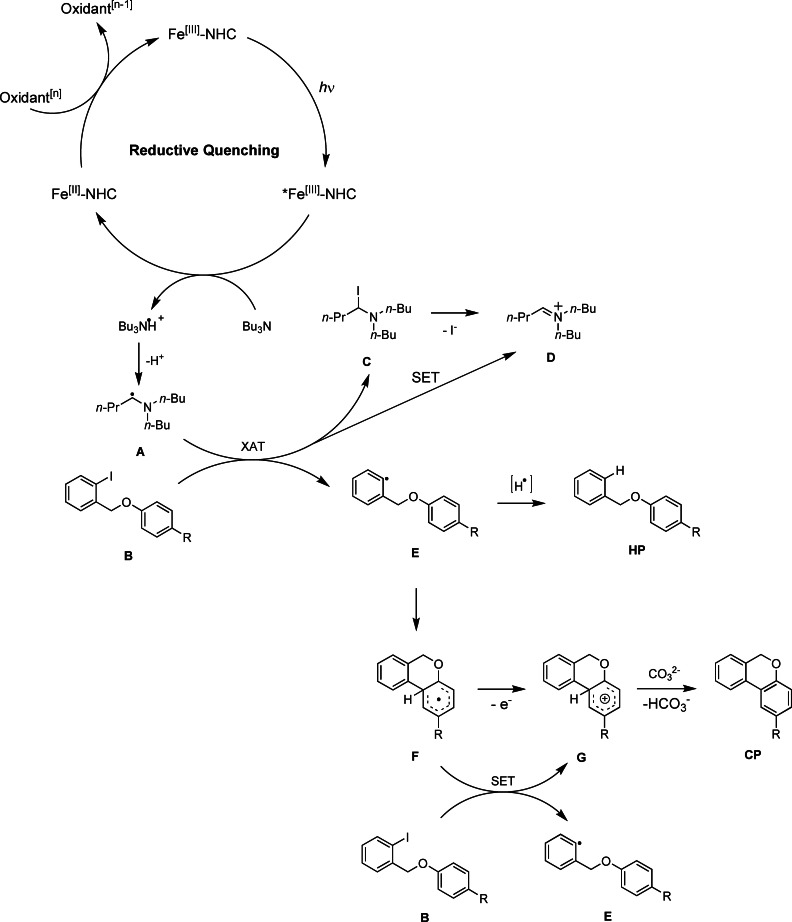
Proposed reaction mechanism for the BHAS reaction employing [Fe(phtmeimb)_2_]^+^ as PC.

Therefore, we propose in Scheme 1 a catalytic cycle that starts with the reductive quenching of the Fe(III)‐ES by Bu_3_N. The resulting Bu_3_N⋅^+^ radical cation, or the subsequently generated α‐aminoalkyl radical **A**, induces homolytic cleavage of the C−I bond of a general substrate **B**, either through a SET or, more likely, an XAT (halogen atom transfer) event.[^31^, ^32^] Such a mechanism, in which a photogenerated α‐aminoalkyl radical participates in SET, HAT (hydrogen atom transfer) or XAT, has been previously shown to be able to assist in such transformations.^[27]^

The reactive phenyl radical intermediate **E** can then proceed to abstract an electron and a proton (either through HAT, or a consecutive reduction and protonation) to form the **HP**, or undergo ring‐closure to form intermediate **F**, followed by the removal of an electron (resulting in intermediate **G**) and a proton. This deprotonation is most likely carried out by the carbonate base, giving the **CP** as final product. The nature of the oxidant that restores the Fe(II) species to the original Fe(III)‐PC has not been unambiguously identified. However, it is not unlikely that the Fe(II) species can reduce intermediate **E**, aiding in the formation of **HP**.

The **HP** could also be formed by subtracting a proton and electron from the Bu_3_N⋅^+^ radical cation, as this is a known pathway that can induce formation of hydrodehalogenated products. However, since only 1 equiv. of Bu_3_N is needed to result in full consumption of the starting material, this would suggest that a radical propagation pathway is potentially at play. This is further corroborated by the observation of the sub‐stoichiometric amounts of 0.2 equiv. of Bu_3_N allowing for 42 % conversion, and 0.5 equiv. of Bu_3_N leading to full conversion in the case of the model substrate **2**. As mentioned before, addition of TEMPO hampers the reactivity and reduces the conversion to 20 %, which also supports the presence of a radical mechanism.

Because the reaction mechanism in Scheme 1 features two oxidation events (Fe(II)‐NHC to Fe(III)‐NHC and intermediate **F** to **G**) and just one reduction (substrate **B** to intermediate **E**), it is not balanced, and therefore implies that internal radical propagation pathways are necessary for the reaction to proceed catalytically. For example, the homolytic cleavage of the C−I bond in substrate **B** could be induced by SET with the cyclized radical intermediate **F**, rather than exclusively by SET from Bu_3_N⋅^+^, or its deprotonated counterpart, the α‐aminoalkyl radical **A**.

The substrates show some variable degree of reductive quenching. The effect of this reaction on the yield of the productive quenching by Bu_3_N is however negligible under the conditions employed (Figure 4) and has therefore probably no impact on the further course of the reaction unless products of the undesired quenching by the substrate interfere with reactions leading to the alternative products. In absence of corresponding evidence it seems more likely that differences in the selectivity **CP–HP** between substrates are due to intrinsically different reactivity. However, the effect of different conditions on the selectivity of **CP** over **HP** suggest that **CP** formation is favored by conditions that result in a low rate of α‐amino radical formation thereby favoring product formation via chain propagation. Higher Bu_3_N concentration, Et_3_N instead of Bu_3_N, or [Ru(bpy)_3_)]Cl_2_ as PS, all favor more efficient α‐amino radical formation (Table 4) but lower **CP–HP** ratios.

The reaction times are rather long (48 h), most likely due to inefficient quenching and charge‐separated product formation, resulting in low reaction quantum yields (0.3–0.4 % as determined by actinometry, Table S13). Making the quenching of the ES more efficient by for example introducing Et_3_N instead of Bu_3_N, or exchanging DMSO for acetonitrile, increases the quenching rates (Table 4 and Supporting Information), but reduces the overall reactivity or reaction selectivity for **CP** over **HP**. This could be attributed to the previously mentioned fast degradation pathway of the amine radical cation in which a proton is donated to form **HP**. Under such reaction conditions, the **HP** formation more considerably outcompetes the initial ring‐closure which thereby hampers the formation of **CP**. This makes the balance between obtaining significant **CP** selectivity as well as satisfactory reactivity challenging to achieve. However, the longevity of the employed Fe(III)‐PC in combination with the use of green LED irradiation can counteract the disadvantage of long reaction times, providing full conversion and favouring **CP** formation.

## Conclusions

In this report we have showcased the successful application of [Fe(phtmeimb)_2_]PF_6_ as an Fe‐PC in intramolecular BHAS reactions, employing green light (λ=530 nm), a mild base (K_2_CO_3_) and an SED (Bu_3_N). After initial optimizations, the found reaction conditions were applied to numerous substrates, featuring a variety of functional groups, leading to the predominant formation of the cyclized product (**CP**) over the hydrodehalogenated side‐product (**HP**), significantly enhancing the substrate scope for the BHAS reaction in general. This methodology offers a novel, more benign, approach to the well‐known BHAS reaction, no longer excluding substrates that feature functional groups sensitive to strongly basic or highly reducing conditions such as aldehydes and esters. As such, our study highlights that the excited state (photo)redox properties of a photocatalyst, and reaction conditions such as choice of base, must be included in the synthesis planning even at the expense of longer reaction times, to allow for the inclusion of redox‐ and base sensitive substrates. The proposed reaction mechanism is initiated by the photoinduced formation of α‐aminoalkyl radicals that enable dehalogenation of the aryliodide substrates via SET or XAT. Despite the low quantum yields, essentially quantitative conversion was accomplished after corresponding reaction times taking advantage of the excellent photostability of the [Fe(III)(phtmeimb)_2_]PF_6_ PS, further expanding the to date limited number of photoredox reactions driven by outer‐sphere mechanisms of iron complexes.[^17^, ^18^, ^22^, ^33^, ^34^, ^35^, ^36^, ^37^, ^38^]

## Supporting Information Summary

The authors have cited additional references within the Supporting Information.[^24^, ^28^, ^39^, ^40^, ^41^, ^42^, ^43^, ^44^, ^45^, ^46^, ^47^, ^48^, ^49^, ^50^]

## Conflict of Interests

The authors declare no conflict of interest.

1

## Supporting information

As a service to our authors and readers, this journal provides supporting information supplied by the authors. Such materials are peer reviewed and may be re‐organized for online delivery, but are not copy‐edited or typeset. Technical support issues arising from supporting information (other than missing files) should be addressed to the authors.

Supporting Information

## Data Availability

The data that support the findings of this study are available from the corresponding author upon reasonable request.
