# Supplementary material for: Base‐Promoted Homolytic Aromatic Substitution (BHAS) Reactions and Hydrodehalogenations Driven by Green Light and an Iron(III)‐NHC Photoredox Catalyst
Source: Chemistry. 2025 Mar 12;31(21):e202500409. doi: 10.1002/chem.202500409 (PMC11979687; doi:10.1002/chem.202500409)
Supplement: Supplementary file 1 — Supporting Information [file CHEM-31-e202500409-s001.pdf]

Chemistry–A European Journal

Supporting Information

Base-Promoted Homolytic Aromatic Substitution (BHAS) Reactions and Hydrodehalogenations Driven by Green Light and an Iron(III)-NHC Photoredox Catalyst

Lisa H. M. de Groot, Clara García-Mateos, Catherine E. Johnson, Valtýr Freyr Hlynsson, Alpesh K. Sharma, Reiner Lomoth,* and Kenneth Wärnmark*

Supporting Information

Base-promoted homolytic aromatic substitution (BHAS) reactions and hydrodehalogenations driven by green light and an iron(III)-NHC photoredox catalyst.

Lisa H. M. de Groot,+[a] Clara García-Mateos,+[a] Catherine E. Johnson,+[b] Valtýr Freyr Hlynsson,+[a] Alpesh K. Sharma,+[a] Reiner Lomoth,*[b] and Kenneth Wärnmark*[a]

[a]Centre for Analysis and Synthesis (CAS), Department of Chemistry, Lund University, SE-22100 Lund, Sweden;

[b]Department of Chemistry – Ångström Laboratory, Uppsala University, SE-75120 Uppsala, Sweden

+These authors contributed equally to this work

*Corresponding Authors

E-mail: reiner.lomoth@kemi.uu.se

E-mail: kenneth.warnmark@chem.lu.se

General Information **4**
Materials and instruments 4
Experimental Section **6**
General procedure for the intramolecular BHAS reaction 6
Reaction Optimizations **7**
Initial reaction optimizations using 1-(2-iodobenzyl)-1*H*-pyrrole (**1**) as substrate 7
Continued reaction optimizations using 1-((4-(*tert*-butyl)phenoxy)methyl)-2-iodobenzene (**2**) as substrate 10
Product Isolation **12**
5*H*-pyrrolo[2,1-*a*]isoindole (**CP 1**) 12
1-Benzyl-1*H*-pyrrole (**HP 1**) 12
2-(*tert*-Butyl)-6*H*-benzo[*c*]chromene (**CP 2**) 13
1-(Benzyloxy)-4-(*tert*-butyl)benzene (**HP 2**) 13
2-Methoxy-6*H*-benzo[*c*]chromene (**CP 3**) 13
1-(Benzyloxy)-4-methoxybenzene (**HP 3**) 14
2-Methyl-6*H*-benzo[*c*]chromene (**CP 4**) 14
1-(Benzyloxy)-4-methylbenzene (**HP 4**) 15
6*H*-benzo[*c*]chromene (**CP 5**) 15
Benzyloxybenzene (**HP 5**) 15
8-(*tert*-Butyl)-6*H*-benzo[*c*]chromene (**CP1 6**) 16
9-(*tert*-Butyl)-6*H*-benzo[*c*]chromene (**CP2 6**) 16
6*H*-benzo[*c*]chromene-2-carbaldehyde (**CP 7**) 17
4-(Benzyloxy)benzaldehyde (**HP 7**) 17
Methyl 6*H*-benzo[*c*]chromene-2-carboxylate (**CP 8**) 18
2-(Trifluoromethyl)-6*H*-benzo[*c*]chromene (**CP 9**) 18
1-(Benzyloxy)-4-(trifluoromethyl)benzene (**HP 9**) 19
2-(*tert*-Butyl)-6*H*-naphtho[2,1-*c*]chromene (**CP 11**) 19
2-((4-(*tert*-Butyl)phenoxy)methyl)naphthalene (**HP 11**) 20
2-(*tert*-Butyl)-8-(trifluoromethyl)-6*H*-benzo[*c*]chromene (**CP 12**) 20
1-((4-(*tert*-Butyl)phenoxy)methyl)-3 (trifluoromethyl)benzene (**HP 12**) 21
Literature studies of oxidative quenching **22**
Mechanistic Investigations **29**
Radical scavenging experiment 29
Quantum yield measurements (actinometry) 29
Cyclic voltammetry measurements 33
Excited state lifetime of $[Fe(phtmeimb)_2](PF_6)$ in DMSO 35
Excited state quenching 35
Cage escape yield measurements 40

Aldehyde side-product......44
NMR Spectra......45
5H-Pyrrolo[2,1-a]isoindole (**CP 1**)......45
1-Benzyl-1H-pyrrole (**HP 1**)......47
2-(tert-Butyl)-6H-benzo[c]chromene (**CP 2**)......49
1-(Benzyloxy)-4-(tert-butyl)benzene (**HP 2**)......51
2-Methoxy-6H-benzo[c]chromene (**CP 3**)......53
1-(Benzyloxy)-4-methoxybenzene (**HP 3**)......56
2-Methyl-6H-benzo[c]chromene (**CP 4**)......58
1-(Benzyloxy)-4-methylbenzene (**HP 4**)......61
6H-benzo[c]chromene (**CP 5**)......63
Benzyloxybenzene (**HP 5**)......66
8-(tert-Butyl)-6H-benzo[c]chromene (**CP1 6**)......68
9-(tert-Butyl)-6H-benzo[c]chromene (**CP2 6**)......70
6H-benzo[c]chromene-2-carbaldehyde (**CP 7**)......72
4-(Benzyloxy)benzaldehyde (**HP 7**)......73
Methyl 6H-benzo[c]chromene-2-carboxylate (**CP 8**)......73
2-(Trifluoromethyl)-6H-benzo[c]chromene (**CP 9**)......74
1-(Benzyloxy)-4-(trifluoromethyl)benzene (**HP 9**)......74
2-(tert-Butyl)-6H-naphtho[2,1-c]chromene (**CP 11**)......75
2-((4-(tert-Butyl)phenoxy)methyl)naphthalene (**HP 11**)......77
2-(tert-Butyl)-8-(trifluoromethyl)-6H-benzo[c]chromene (**CP 12**)......80
1-((4-(tert-Butyl)phenoxy)methyl)-3 (trifluoromethyl)benzene (**HP 12**)......82

General Information

Materials and instruments

Materials. All solvents used in work-up procedures, for silica gel column chromatography and/or purification were obtained from commercial suppliers and used without further purification. Reagents *i.e.*, additives and deuterated solvents for NMR-spectroscopy were purchased from Sigma-Aldrich and Acros Organics and used without further purification. Tributylamine was distilled and potassium carbonate was dried under vacuum and heating before use. The substrates were prepared according to a literature protocol,[39] unless otherwise specified in the respective experimental part. $[Fe(phtmeimb)_2](PF_6)$, $[Fe(btz)_3](PF_6)_3$ and $[Fe(bpy)_3](PF_6)_2$ were prepared according to literature protocols.[28,40,41]

Photoreactions. Photoreactions were performed in a TAK120 AC photoreactor purchased from HK Testsysteme GmbH. The irradiation was performed using the green LED array (λ = 530 nm, 3.03 W/vial) in 6 mL clear glass crimp vials with septum. The temperature during reaction was maintained at 27–30°C using air cooling.

Chromatography. Precoated Merck silica gel 60 F254 plates were used for thin-layer-chromatography (TLC) analysis and products were visualised using UV light or a *p*-anisaldehyde stain which was developed under heating. Flash and gravitational silica gel chromatography was performed using Merck silica gel (pore size 60 Å, 230–400 mesh particle size, particle size 0.043–0.063 mm). Preparative reverse phase HPLC was performed on an Agilent Technologies 1260 Infinity HPLC with Waters Symmetry C18 column, 5 µm, 19 × 100 mm. Size exclusion chromatography was performed on Sephadex.

Characterization. NMR spectra were recorded at ambient temperature on a BrukerAvance II 400 MHz NMR spectrometer (400/101 MHz $^{1}H/^{13}C$ or 500 MHz ^{1}H). Chemical shifts (δ) for ^{1}H and ^{13}C NMR spectra were reported in parts per million (ppm), relative to the residual solvent peak of the respective NMR solvent ($CDCl_3$ (δ_H = 7.26 ppm and δ_C = 77.16 ppm) or DMSO-d_6 (δ_H = 2.50 ppm and δ_C = 39.52 ppm)). Coupling constants (J) are given in Hertz (Hz), with the multiplicities being denoted as follows: singlet (s), doublet (d), triplet (t), quartet (q), quintet (qi), multiplet (m). NMR spectra for ^{13}C were recorded with decoupling from ^{1}H. The spectra were assigned by 2D-NMR techniques. Electrospray ionization–high resolution mass spectrometry (ESI–HRMS) was recorded on a Waters Micromass Q-Tof micro mass spectrometer. Elemental analyses were performed by Mikroanalytisches Laboratorium KOLBE (Müllheim an der Ruhr, Germany). Melting points were corrected against vanillin (melting point 81(1) °C).[42]

UV-Vis spectroscopy. UV-Vis absorption spectra were recorded on a Probe Drum Lab-in-a-box spectrometer or a Varian Cary 50 Spectrophotometer. Samples were contained in a 10 mm absorption quartz cuvette.

Emission and excitation spectra measurements. Steady-state emission (excitation wavelength 520 nm) and excitation spectra measurements were performed on FS5 (Edinburgh Instruments) or Fluorolog-3 (Horiba) fluorimeters with slit widths set to 4 nm spectral resolution. Emission and excitation spectra from both instruments were background subtracted and corrected for the wavelength dependent instrument response.

Solutions of $[Fe(phtmeimb)_2]PF_6$ were prepared in dimethyl sulfoxide (spectroscopic grade Uvasol®, ≥99.9%, from Merck) or acetonitrile (spectroscopic grade Uvasol®, ≥99.9%, from Merck) with absorption of around 0.05 ± 0.005 at 502 nm. UV-Vis absorption and two emission spectra were taken for each quencher concentration and emission intensities were corrected for minor differences in absorbance at the excitation wavelength. In addition, background emission measurements for just the quencher at the same concentrations were measured. Stern-Volmer plots were constructed from the emission intensities taken at 680 nm (in DMSO) and 650 nm (in acetonitrile) from the averaged and background subtracted spectra and fitted with Origin software.

Emission lifetimes were determined by TCSPC performed with the FS5 (Edinburgh Instruments) fluorimeter. Emission decays were recorded with slit width of 8 nm and counts of around 65,000. The instrument response function (IRF) was collected with a scattering sample (LUDOX). The kinetic decays were fitted along with the IRF using the in-built software.

Nanosecond transient absorption measurements. Nanosecond transient absorption measurements were obtained with a LP920-S laser flash photolysis spectrometer (Edinburgh Instruments) equipped with an iStar CCD camera (Andor Technology) for transient spectra and a LP920-K PMT detector connected to a TDS 3052 500 MHz 5 GS/s oscilloscope (Tektronix) for single wavelength kinetics. Probe light was provided by a pulsed XBO 450 W Xenon Arc Lamp (Osram) and samples were excited at 465 nm with 8 ns pulses provided by a

frequency tripled Q-switched Nd:YAG laser (EKSPLA NT342B) combined with an optical parametric oscillator (OPO). All measurements were performed at right angle in a 10 × 10 mm quartz cuvette with samples deaerated by purging with argon and an absorption of around 0.5 at the excitation wavelength.

Femtosecond transient absorption measurements. Fs-TAS was performed probing in the UV-Vis region using a Newport TAS. A Coherent Libra Ti:sapphire amplifier (1.5 mJ, 3kHz, 800 nm, fwhm 40 fs) was used and split into pump and probe beams. Excitation wavelength of 475 nm was generated by directing the pump beam into the optical parametric amplifiers (TOPAS-Prime and NIRUVVIS, Light Conversion) and then focused and centered on the 1 mm cuvette with a pump power adjusted to 3.0 ± 0.2 mW. The 800 nm fundamental of the amplifier was focused on a CaF_2 crystal (Crystran), generating the white light supercontinuum probe. A silicon diode array (Newport custom made) was used to record the probe spectrum. A mechanical chopper blocked every other pump pulse, and the transient absorption at each time point was calculated for an average of 1000 ms chopped/un-chopped pulse pairs. To record the transient absorption spectra at different time points, an optical delay line was used to scan the delay of the probe beam relative to the pump beam from -5 ps to 8 ns. A total of ten scans were collected and averaged for each sample. Prior to analysis, the data was corrected for the spectral chirp using Surface Xplorer v4, where single wavelength fits were also performed.

Cyclic Voltammetry measurements. All cyclic voltammetry measurements were performed in acetonitrile (spectroscopic grade Uvasol®, ≥99.9%, Merck; dried over 3Å molecular sieves activated at 300 °C for 15 hours) with 0.1 M tetrabutylammonium hexafluorophosphate (electrochemical grade, Sigma Aldrich; dried at 80 °C under vacuum) and purged with solvent-saturated argon. The samples were prepared with concentrations of 1 mM.

The measurements were carried out in a three-electrode electrochemical cell, using an AUTOLAB potentiostat (PGSTAT302) controlled with GPES software (Version 4.9). The working electrode was a freshly-polished (with Buehler alumina paste) glassy carbon electrode (CH Instruments, 1 mm diameter); the reference electrode was an aqueous $Ag/AgNO_3$ solution (CH Instruments; 10 mM of $AgNO_3$ dissolved in dried acetonitrile; 0.088 V vs. ferrocene) and a Pt wire in a separate compartment was used as counter electrode.

Experimental Section

General procedure for the intramolecular BHAS reaction

Photochemical reactions were conducted in a TAK120 AC photoreactor purchased from HK Testsysteme GmbH. A 6 mL clear glass crimp top vial equipped with a magnetic stir bar was charged with the photocatalyst (PC) (2 mol%, 2 μmol), substrate (0.1 mmol, 1 equiv), tributylamine (Bu_3N) (0.1 mmol, 1 equiv), potassium carbonate (K_2CO_3) (0.2 mmol, 2 equiv) and deuterated dimethyl sulfoxide (DMSO-d_6) (solvent) (2 mL).

After sealing the vial using an aluminium cap with a septum, the reaction solution was flushed with argon for 15 min. The sample was then irradiated in the photoreactor at 530 nm (3.03 W per slot) under vigorous stirring for 48 h, unless otherwise specified.

Condition and scope screenings were also performed in deuterated solvents and NMR yields were determined via ^{1}H NMR spectroscopy by integration against the internal standard (IS) 1,3,5-trimethoxybenzene (16.82 mg, 0.1 mmol per sample). The IS was added to the reaction crude upon completion of the reaction and the mixture was stirred thoroughly before taking a 50 μL aliquot, which was diluted with 350 μL of deuterated solvent before conducting ^{1}H NMR analysis.

Isolation was performed on reaction samples to which no IS was added, and those in which full conversion of starting material was achieved, as judged by ^{1}H NMR analysis of a 50 μL aliquot, which was diluted with 350 μL of deuterated solvent. The sample used for ^{1}H NMR analysis was recombined with the reaction crude prior to product isolation. For more facile product characterisation, multiple crude reaction batches were combined (the individual number of which is indicated for the respective products) and extracted three times using ethyl acetate (EtOAc) and water. The combined organic phases were washed with brine, before drying over anhydrous magnesium sulphate ($MgSO_4$). The slurry was filtered and concentrated under reduced pressure. The reaction crude was subsequently purified using silica gel column chromatography, and continued purification was done using reverse phase preparative HPLC (acetonitrile–water mixture, and 0.1% formic acid), unless otherwise specified.

Reaction Optimizations

All optimization reactions were conducted following the general procedure described in the experimental section, using either 1-(2-iodobenzyl)-1*H*-pyrrole or 1-((4-(*tert*-butyl)phenoxy)methyl)-2-iodobenzene as starting material as well as the indicated sacrificial electron donors and bases. All equivalent amounts and photocatalyst (PC) loadings were given in relation to the substrate (0.1 mmol) and the reactions were performed in deuterated dimethyl sulfoxide (DMSO-d_6), unless otherwise stated. NMR conversions were determined by the integration of the benzylic signals of the **CP** (cyclized product) and the **HP** (hydrogen addition product) in the ^{1}H NMR of the crude versus the remaining benzylic signal belonging to the substrate.

Initial reaction optimizations using 1-(2-iodobenzyl)-1*H*-pyrrole (1) as substrate

Table S1. Optimization of the catalyst loading using the general procedure. All experiments were performed using 1-(2-iodobenzyl)-1*H*-pyrrole **1** (0.1 mmol) in DMSO-d_6 (2 mL) under 530 nm irradiation and argon atmosphere in absence of an additional base. phtmeimb = phenyl(tris(3-methylimidazol-1-ylidene))borate, Bu_3N = tributylamine.

Entry	PC	[PC] (mol%)	Sacrificial electron donor	t (h)	^{1}H NMR yield (%**CP**, %**HP**)
**1**	$[Fe(phtmeimb)_2]PF_6$	1	Bu_3N (2 equiv)	24	31, 5
**2**	$[Fe(phtmeimb)_2]PF_6$	2	Bu_3N (2 equiv)	24	48, 8
**3**	$[Fe(phtmeimb)_2]PF_6$	2	Bu_3N (2 equiv)	48	58, 13
**4**	$[Fe(phtmeimb)_2]PF_6$	4	Bu_3N (2 equiv)	24	46, 9
**5**	$[Fe(phtmeimb)_2]PF_6$	4	Bu_3N (2 equiv)	48	53, 10

Table S2. Variation of the sacrificial electron donor using the general procedure. All experiments were performed using 1-(2-iodobenzyl)-1*H*-pyrrole **1** (0.1 mmol) in DMSO-d_6 (2 mL) under 530 nm irradiation and argon atmosphere in absence of an additional base. Et_3N = triethylamine, DIPEA = *N*,*N*-diisopropylethylamine, nr = no reaction.

Entry	PC	[PC] (mol%)	Sacrificial electron donor	t (h)	^{1}H NMR yield (%**CP**, %**HP**)
**1**	$[Fe(phtmeimb)_2]PF_6$	1	Et_3N (2 equiv)	24	nr
**2**	$[Fe(phtmeimb)_2]PF_6$	1	Et_3N (5 equiv)	24	nr
**3**	$[Fe(phtmeimb)_2]PF_6$	1	DIPEA (2 equiv)	48	35, 30
**4**	$[Fe(phtmeimb)_2]PF_6$	1	Bu_3N (2 equiv)	24	31, 5
**5**	$[Fe(phtmeimb)_2]PF_6$	1	Bu_3N (5 equiv)	24	42, 8

Table S3. Variation of the solvent using the general procedure. All experiments were performed using 1-(2-iodobenzyl)-1*H*-pyrrole **1** (0.1 mmol) and Bu_3N (2 equiv) as sacrificial electron donor under 530 nm irradiation and argon atmosphere in absence of an additional base. DMF = dimethylformamide, MeCN = acetonitrile.

Entry	PC	[PC] (mol%)	Solvent	t (h)	^{1}H NMR yield (%**CP**, %**HP**)
**1**	$[Fe(phtmeimb)_2]PF_6$	1	DMF-d_7	24	25, 60
**2**	$[Fe(phtmeimb)_2]PF_6$	1	MeCN-d_3	24	35, 25
**3**	$[Fe(phtmeimb)_2]PF_6$	1	DMSO-d_6	24	31, 5

Table S4. Variation of the base system using the general procedure. All experiments were performed using 1-(2-iodobenzyl)-1*H*-pyrrole **1** (0.1 mmol), with $[Fe(phtmeimb)_2]PF_6$ as PC (2 mol%) in DMSO-d_6 (2 mL) under 530 nm irradiation and argon atmosphere. TMP = 2,2,6,6-tetramethylpiperidine.

Entry	Sacrificial electron donor	Additional base	t (h)	^{1}H NMR (%**CP**, %**HP**)
**1**	Bu_3N (2 equiv)	TMP (1 equiv)	48	51, 19
**2**	Bu_3N (1 equiv)	TMP (1 equiv)	48	16, 0
**3**	Bu_3N (1 equiv)	TMP (2 equiv)	48	37, 17
**4**	Bu_3N (2 equiv)	TMP (2 equiv)	48	75, 25
**5**	Bu_3N (3 equiv)	TMP (3 equiv)	48	72, 23
**6**	Bu_3N (4 equiv)	TMP (2 equiv)	48	68, 20

Table S5. Control experiments using the general procedure. All experiments were performed using 1-(2-iodobenzyl)-1*H*-pyrrole **1** (0.1 mmol) and Bu_3N (2 equiv) as sacrificial electron donor and TMP (2 equiv) as additional base in DMSO-d_6 (2 mL) under 530 nm irradiation and argon atmosphere. btz = (3,3'-dimethyl-1,1'-bis(*p*-tolyl)-4,4'-bis(1,2,3-triazol-5-ylidene)), bpy = 2,2-bipyridine.

Entry	PC	[PC] (mol%)	t (h)	^{1}H NMR yield (%**CP**, %**HP**)
**1**	$[Fe(phtmeimb)_2]PF_6$	2	24	65, 17
**2**	$[Fe(btz)_3](PF_6)_3$	2	24	nr
**3**	$FeBr_2$	2	24	nr
**4**	$[Fe(bpy)_3](PF_6)_2$	2	24	nr
**5**	$[Ru(bpy)_3]Cl_2$	2	24	32, 22
**6**	–	–	24	nr
**7**[a]	$[Fe(phtmeimb)_2]PF_6$	2	24	nr
**8**[b]	$[Fe(phtmeimb)_2]PF_6$	2	24	nr
**9**[c]	$[Fe(phtmeimb)_2]PF_6$	2	24	31, 5
**10**[d]	$[Fe(phtmeimb)_2]PF_6$	2	24	0, 5

[a]No irradiation. [b]No sacrificial electron donor and no additional base added. [c]Only in presence of Bu_3N (2 equiv), without TMP. [d]Only in presence of TMP (2 equiv), without Bu_3N.

The following optimized reaction conditions were initially established: $[Fe(phtmeimb)_2]PF_6$ (2 mol%) as PC, Bu_3N (2 equiv) as sacrificial electron donor, in combination with TMP (2 equiv) as an additional base, in DMSO as reaction solvent (Table S4, entry 4).

Upon completion of the initial optimizations of the reaction conditions, using 1-(2-iodobenzyl)-1*H*-pyrrole as the substrate, it was found that this substrate degrades under ambient conditions after prolonged storage time. Therefore, 1-((4-(*tert*-butyl)phenoxy)methyl)-2-iodobenzene was used as the substrate when conducting further optimizations, as this is a more stable and hence more facile substrate to conduct experiments with.

Continued reaction optimizations using 1-((4-(*tert*-butyl)phenoxy)methyl)-2-iodobenzene (2) as substrate

Table S6. Variation of the base system using the general procedure. All experiments were performed using 1-((4-(*tert*-butyl)phenoxy)methyl)-2-iodobenzene **2** (0.1 mmol), with $[Fe(phtmeimb)_2]^+$ as PC (2 mol%) in DMSO-d_6 (2 mL) under 530 nm irradiation and argon atmosphere.

Entry	Sacrificial electron donor	Additional base	t (h)	^{1}H NMR yield (%**CP**, %**HP**)
**1**	Bu_3N (2 equiv)	TMP (2 equiv)	48	53, 43
**2**	Bu_3N (1 equiv)	–	48	21, 8
**3**	Bu_3N (3 equiv)	–	48	57, 34
**4**	Bu_3N (5 equiv)	–	48	9, 5
**5**	Bu_3N (2 equiv)	K_2CO_3 (2 equiv)	48	59, 41
**6**	–	K_2CO_3 (4 equiv)	48	nr
**7**	–	K_2CO_3 (2 equiv)	48	nr
**8**	Bu_3N (1 equiv)	K_2CO_3 (2 equiv)	48	74, 26

For 1-((4-(*tert*-butyl)phenoxy)methyl)-2-iodobenzene it was found that 53% **CP** and 43% **HP** could be achieved using the previously established optimized reaction conditions (Table S6, entry 1). However, upon further investigation of the base system, K_2CO_3 (2 equiv) was found to be a superior base to TMP and could be used together with just 1 equiv of Bu_3N to achieve the optimal reaction outcome (Table S6, entry 8). Control experiments were conducted for this substrate as well (Table S7).

Table S7. Control experiments using the general procedure. All experiments were performed using 1-((4-(*tert*-butyl)phenoxy)methyl)-2-iodobenzene **2** (0.1 mmol) and Bu_3N (1 equiv) as sacrificial electron donor and K_2CO_3 (2 equiv) as additional base in DMSO-d_6 (2 mL) under 530 nm irradiation and argon atmosphere. [a]No irradiation. [b]No sacrificial electron donor and no additional base added. [c]TEMPO (2,2,6,6-tetramethylpiperidine-1-oxyl) added as a radical trapping agent.

Entry	PC	[PC] (mol%)	t (h)	^{1}H NMR yield (%**CP**, %**HP**)
**1**	$[Fe(phtmeimb)_2]PF_6$	2	22	14, 7
**2**	$[Fe(btz)_3](PF_6)_3$	2	48	nr
**3**	$FeBr_2$	2	48	nr
**4**	$[Fe(bpy)_3](PF_6)_2$	2	48	nr
**5**	$[Ru(bpy)_3]Cl_2$	2	48	37, 17
**6**	–	–	48	nr
**7**[a]	$[Fe(phtmeimb)_2]PF_6$	2	48	nr
**8**[b]	$[Fe(phtmeimb)_2]PF_6$	2	48	nr
**9**[c]	$[Fe(phtmeimb)_2]PF_6$	2	48	7, 13

The newly found optimized reaction conditions were also applied to 1-(2-iodobenzyl)-1*H*-pyrrole, providing an improved optimised NMR conversion ratio of 83% **CP** and 17% **HP** (Table S8). Key control experiments were repeated for this substrate (Table S8).

Table S8. Control experiments using the general procedure. All experiments were performed using 1-(2-iodobenzyl)-1*H*-pyrrole (0.1 mmol) and Bu_3N (1 equiv) as sacrificial electron donor and K_2CO_3 (2 equiv) as additional base in DMSO-d_6 (2 mL) under 530 nm irradiation and argon atmosphere.

Entry	PC	[PC] (mol%)	t (h)	^{1}H NMR yield (%**CP**, %**HP**)
**1**	$[Fe(phtmeimb)_2]PF_6$	2	48	83, 17
**2**	$[Fe(btz)_3](PF_6)_3$	2	48	nr
**3**	–	–	48	nr
**4**	$[Ru(bpy)_3]Cl_2$	2	48	35, 17

Previous research has shown that efficient dehalogenation can be achieved using Bu_3N (5 equiv) as a sacrificial electron donor and formic acid (5 equiv) as an additive in acetonitrile with 5 mol% of an organic photocatalyst (10-phenylphenothiazine (PTH)) under 380 nm irradiation.[25] Employing Bu_3N (5 equiv) and formic acid (5 equiv) in deuterated acetonitrile as solvent, in presence of our PC (2 mol%) under 530 nm irradiation led to a large preference for the formation of **HP** instead of **CP** (Table S9), making this a useful protocol for the formation of the **HP** product.

Table S9. Exploration of an acid additive using the general procedure. All experiments were performed using $[Fe(phtmeimb)_2]PF_6$ as PC (2 mol%) in MeCN-d_3 (2 mL) under 530 nm irradiation and argon atmosphere.

Entry	Substrate	Sacrificial electron donor	Additive	t (h)	^{1}H NMR yield (%**CP**, %**HP**)
**1**	1-(2-iodobenzyl)-1*H*-pyrrole	Bu_3N (5 equiv)	HCOOH (5 equiv)	24	2, 70
**2**	1-(2-iodobenzyl)-1*H*-pyrrole	Bu_3N (5 equiv)	HCOOH (5 equiv)	48	18, 82
**3**	1-((4-(*tert*-butyl)phenoxy)methyl)-2-iodobenzene	Bu_3N (5 equiv)	HCOOH (5 equiv)	24	2, 64
**4**	1-((4-(*tert*-butyl)phenoxy)methyl)-2-iodobenzene	Bu_3N (5 equiv)	HCOOH (5 equiv)	48	10, 90

Product Isolation

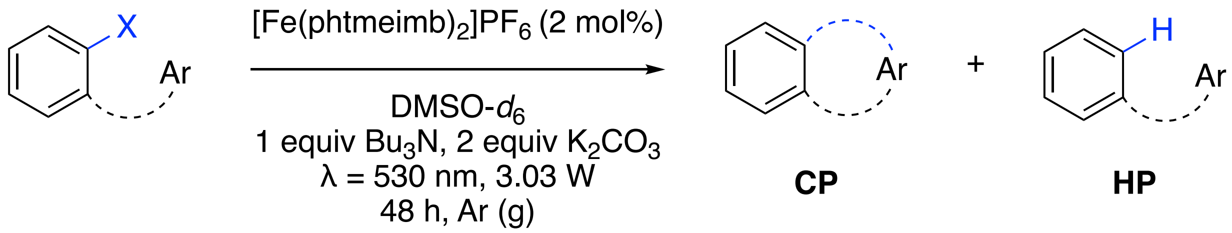

The following reactions were performed using the standard protocol as specified in the general procedure. The substrates were synthesized according to a literature protocol,[39] unless otherwise specified.

5*H*-pyrrolo[2,1-*a*]isoindole (CP 1)

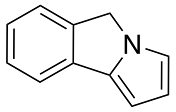

1-(2-Iodobenzyl)-1*H*-pyrrole (28.3 mg, 0.1 mmol, 1 equiv), tributylamine (24 µL, 0.1 mmol, 1 equiv), K_2CO_3 (27.6 mg, 0.2 mmol, 2 equiv), $[Fe(phtmeimb)_2](PF_6)$ (1.73 mg, 2.0 µmol, 0.02 equiv), DMSO-d_6 (2 mL) and a stir bar were combined in a 6 mL crimp vial. After closing, the suspension was purged with argon for 15 min. The suspension was subjected to 48 h of irradiation with green light (530 nm, 3.03 W) in a TAK120 AC photoreactor under vigorous stirring. ^{1}H NMR of a 50 µL aliquot, diluted with 350 µL of DMSO-d_6 showed full consumption of the starting material (product ratio **CP 1**/**HP 1** = 83:17).

The contents of five parallel reactions were added to 100 mL EtOAc in a separatory funnel. The solution was washed with 3 x 200 mL of H_2O before extracting the combined aqueous phases with 100 mL EtOAc. The combined organic phases were washed with brine, dried over anhydrous $MgSO_4$, filtrated, and concentrated in vacuo. The crude was purified by silica gel flash chromatography (2 x 10 cm, petroleum ether (PE)), after which the mixed product fractions were subjected to a Sephadex LH-20 column (1 x 100 cm, MeOH), affording 45.7 mg (0.29 mmol, 59%) of **CP 1** as a white solid.

R_f = 0.58 (EtOAc/PE 1:9); mp 87.9 – 89.4 °C (lit. mp 86–88 °C)[43]; ^{1}H NMR (400 MHz, $CDCl_3$, δ) 7.47 (dt, J = 7.7, 0.9 Hz, 1H), 7.35 – 7.29 (m, 2H), 7.14 (td, J = 7.5, 1.1 Hz, 1H), 6.98 – 6.91 (m, 1H), 6.34 (dd, J = 3.6, 2.6 Hz, 1H), 6.28 (dd, J = 3.6, 1.1 Hz, 1H), 4.93 (s, 2H) ppm; ^{13}C NMR (101 MHz, $CDCl_3$, δ) 140.4, 138.7, 134.0, 128.1, 125.0, 123.1, 118.8, 116.6, 112.9, 98.1, 50.4 ppm. HRMS-EI: m/z calcd for $C_{11}H_9N$: 156.0813; found: 156.0812 $[M]^+$; elemental analysis calcd (%) for $C_{11}H_9N \cdot 0.1\ H_2O$: C 84.06, H 5.96, N 8.87; found: C 84.15, H 5.91, N 8.92.

1-Benzyl-1*H*-pyrrole (HP 1)

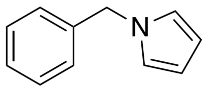

Following the procedure for **CP 1** for purification, 8.3 mg (0.05 mmol, 11%) of **HP 1** was obtained as a colourless oil.

R_f = 0.67 (EtOAc/PE 1:9); ^{1}H NMR (400 MHz, $CDCl_3$, δ) 7.35 – 7.27 (m, 3H), 7.13 – 7.11 (m, 2H), 6.70 (t, J = 2.1 Hz, 2H), 6.20 (t, J = 2.1 Hz, 2H), 5.08 (s, 2H) ppm; ^{13}C NMR (101 MHz, $CDCl_3$, δ) 138.3, 128.9, 127.8, 127.1, 121.3, 108.6, 53.5 ppm; HRMS-EI: m/z calcd for $C_{11}H_{11}N$: 158.0970; found: 158.0960 $[M]^+$; elemental analysis calcd (%) for $C_{11}H_{11}N \cdot 0.15\ H_2O$: C 82.97, H 7.46, N 8.43; found: C 82.62, H 7.12, N 8.76.

2-(*tert*-Butyl)-6*H*-benzo[*c*]chromene (CP 2)

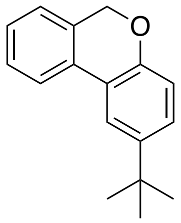

1-((4-(*tert*-Butyl)phenoxy)methyl)-2-iodobenzene (36.6 mg, 0.1 mmol, 1 equiv), tributylamine (24 μL, 0.1 mmol, 1 equiv), K_2CO_3 (27.6 mg, 0.2 mmol, 2 equiv), [Fe(phtmeimb)$_2$](PF$_6$) (1.73 mg, 2.0 μmol, 0.02 equiv), DMSO-*d*$_6$ (2 mL) and a stir bar were combined in a 6 mL crimp vial. After closing, the suspension was purged with argon for 15 min. The suspension was subjected to 48 h of irradiation with green light (530 nm, 3.03 W) in a TAK 120 AC photoreactor under vigorous stirring. ^{1}H NMR of a 50 μL aliquot, diluted with 350 μL of DMSO-*d*$_6$ showed full consumption of the starting material (product ratio **CP 2**/**HP 2** = 74:26).

The contents of five parallel reactions were added to 100 mL EtOAc in a separatory funnel. The solution was washed with 3 x 200 mL of H_2O before extracting the combined aqueous phases with 100 mL EtOAc. The combined organic layers were washed with brine, dried over anhydrous $MgSO_4$, filtrated, and concentrated in vacuo. The crude was purified by silica gel flash chromatography (2 x 10 cm, PE), after which the mixed product fractions were subjected to a Sephadex LH-20 column (1 x 100 cm, MeOH), affording 55.4 mg (0.23 mmol, 46%) of **CP 2** as a colourless oil.

R$_f$ = 0.83 (EtOAc/PE 1:19); ^{1}H NMR (400 MHz, $CDCl_3$, δ) 7.75 (d, *J* = 2.4 Hz, 1H), 7.73 (d, *J* = 7.7 Hz, 1H), 7.40 – 7.36 (m, 1H), 7.29 – 7.25 (m, 2H), 7.16 – 7.14 (m, 1H), 6.93 (d, *J* = 8.5 Hz, 1H), 5.10 (s, 2H), 1.37 (s, 9H) ppm; ^{13}C NMR (101 MHz, $CDCl_3$, δ) 152.7, 145.0, 131.8, 130.7, 128.5, 127.6, 126.8, 124.8, 122.3, 122.0, 120.0, 116.9, 68.7, 34.6, 31.7 ppm; HRMS-EI: m/z calcd for $C_{17}H_{18}O$: 238.1358; found: 238.1346 $[M]^+$; elemental analysis calcd (%) for $C_{17}H_{18}O\cdot 0.1\ H_2O$: C 85.03, H 7.64; found: C 85.14, H 7.83.

1-(Benzyloxy)-4-(*tert*-butyl)benzene (HP 2)

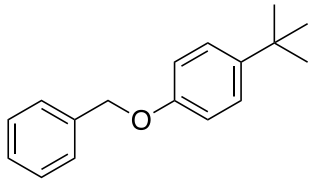

Following the procedure for **CP 2** for purification, 27.4 mg (0.11 mmol, 23%) of **HP 2** was obtained as a white solid.

R$_f$ = 0.93 (EtOAc/PE 1:19); mp 65.2 – 65.6 °C (lit. mp 63–65 °C)[44]; ^{1}H NMR (400 MHz, $CDCl_3$, δ) 7.45 – 7.43 (m, 2H), 7.41 – 7.37 (m, 2H), 7.34 – 7.29 (m, 3H), 6.94 – 6.90 (m, 2H), 5.05 (s, 2H), 1.30 (s, 9H) ppm; ^{13}C NMR (101 MHz, $CDCl_3$, δ) 156.7, 143.8, 137.5, 128.7, 128.0, 127.6, 126.4, 114.4, 70.2, 34.2, 31.7 ppm; HRMS-EI: m/z calcd for $C_{17}H_{20}O$: 240.1514; found: 240.1525 $[M]^+$; elemental analysis calcd (%) for $C_{17}H_{20}O\cdot 0.05\ H_2O$: C 84.64, H 8.40; found: C 84.49, H 8.36.

2-Methoxy-6*H*-benzo[*c*]chromene (CP 3)

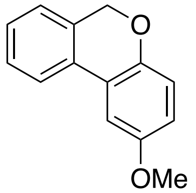

1-Iodo-2-((4-methoxyphenoxy)methyl)benzene (34.0 mg, 0.1 mmol, 1 equiv), tributylamine (24 μL, 0.1 mmol, 1 Eq), K_2CO_3 (27.6 mg, 0.2 mmol, 2 equiv), [Fe(phtmeimb)$_2$](PF$_6$) (1.73 mg, 2.0 μmol, 0.02 equiv), DMSO-*d*$_6$ (2 mL) and a stir bar were combined in a 6 mL crimp vial. After closing, the suspension was purged with argon

for 15 min. The suspension was subjected to 48 h of irradiation with green light (530 nm, 3.03 W) in a TAK 120 AC photoreactor under vigorous stirring. ^{1}H NMR of a 50 μL aliquot, diluted with 350 μL of DMSO-d_6 showed full consumption of the starting material (product ratio **CP 3**/**HP 3** = 82:18).

The contents of five parallel reactions were added to 100 mL EtOAc in a separatory funnel. The solution was washed with 3 x 200 mL of H_2O before extracting the combined aqueous phases with 100 mL EtOAc. The combined organic layers were washed with brine, dried over anhydrous $MgSO_4$, filtrated, and concentrated in vacuo. The crude was purified by silica gel flash chromatography (2 x 13 cm, 1% EtOAc in PE), after which the mixed product fractions were subjected to preparative reverse phase HPLC (MeCN/H_2O 50:50 → 65:35 → 100:0 with 0.1% formic acid (FA) in both eluents) affording 40.1 mg (0.19 mmol, 35%) of **CP 3** as a pale-yellow oil.

R_f = 0.41 (EtOAc/PE 1:19); ^{1}H NMR (500 MHz, $CDCl_3$, δ) 7.67 (d, J = 7.7 Hz, 1H), 7.38 (td, J = 7.6, 1.3 Hz, 1H), 7.29 (td, J = 7.5, 1.2 Hz, 1H), 7.26 (d, J = 3.1 Hz, 1H), 7.16 (d, J = 7.6 Hz, 1H), 6.94 (d, J = 8.7 Hz, 1H), 6.82 (dd, J = 8.8, 3.0 Hz, 1H), 5.07 (s, 2H), 3.85 (s, 3H) ppm; ^{13}C NMR (126 MHz, $CDCl_3$, δ) 154.9, 149.0, 132.0, 130.3, 128.5, 127.9, 124.8, 123.7, 122.2, 118.1, 115.2, 108.5, 68.7, 55.9 ppm. HRMS-EI: m/z calcd for $C_{14}H_{11}O_2$: 211.0759; found 211.0767 $[M-H]^+$; elemental analysis calcd (%) for $C_{14}H_{12}O_2 \cdot 0.2\ H_2O$: C 77.90, H 5.79; found: C 77.88, H 5.88.

1-(Benzyloxy)-4-methoxybenzene (HP 3)

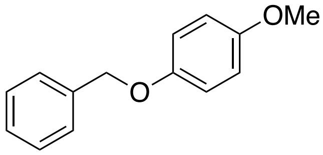

Following the procedure for **CP 3** for purification, 9.2 mg (0.042 mmol, 8%) of **HP 3** was obtained as a white solid.

R_f = 0.41 (EtOAc/PE 1:19); mp 71.5 – 72.1 °C (lit. mp 72 – 74 °C)[45]; ^{1}H NMR (400 MHz, $CDCl_3$, δ) 7.44 – 7.42 (m, 2H), 7.40 – 7.36 (m, 2H), 7.34 – 7.30 (m, 1H), 6.94 – 6.90 (m, 2H), 6.86 – 6.82 (m, 2H), 5.02 (s, 2H), 3.77 (s, 3H) ppm; ^{13}C NMR (101 MHz, $CDCl_3$, δ) 154.1, 153.1, 137.4, 128.7, 128.0, 127.6, 116.0, 114.8, 70.8, 55.9 ppm; HRMS-EI; m/z calcd for $C_{14}H_{15}O_2$: 215.1072; found: 215.1077 $[M+H]^+$; elemental analysis calcd (%) for $C_{14}H_{14}O_2$: C 78.48, H 6.59; found: C 78.13, H 6.61.

2-Methyl-6*H*-benzo[*c*]chromene (CP 4)

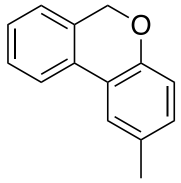

1-Iodo-2-((*p*-tolyloxy)methyl)benzene (32.4 mg, 0.1 mmol, 1 equiv), tributylamine (24 μL, 0.1 mmol, 1 Eq), K_2CO_3 (27.6 mg, 0.2 mmol, 2 equiv), [Fe(phtmeimb)$_2$](PF$_6$) (1.73 mg, 2.0 μmol, 0.02 equiv), DMSO-d_6 (2 mL) and a stir bar were combined in a 6 mL crimp vial. After closing, the suspension was purged with argon for 15 min. The suspension was subjected to 48 h of irradiation with green light (530 nm, 3.03 W) in a TAK 120 AC photoreactor under vigorous stirring. ^{1}H NMR of a 50 μL aliquot, diluted with 350 μL of DMSO-d_6 showed full consumption of the starting material (product ratio **CP 4**/**HP 4** = 71:29).

The contents of five parallel reactions were added to 100 mL EtOAc in a separatory funnel. The solution was washed with 3 x 200 mL of H_2O before extracting the combined aqueous phases with 100 mL EtOAc. The combined organic layers were washed with brine, dried over anhydrous $MgSO_4$, filtrated, and concentrated in vacuo. The crude was purified by silica gel flash chromatography (2 x 11 cm, PE), after which the mixed product fractions were subjected to preparative reverse phase HPLC (MeCN/H_2O 50:50 → 65:35 → 100:0 with 0.1% formic acid (FA) in both eluents) affording 25.0 mg (0.13 mmol, 26%) of **CP 4** as a colourless oil.

R_f = 0.33 (EtOAc/PE 1:49); ^{1}H NMR (400 MHz, $CDCl_3$, δ) 7.70 (dd, J = 7.7, 1.2 Hz, 1H), 7.55 (d, J = 2.1 Hz, 1H), 7.38 (td, J = 7.6, 1.4 Hz, 1H), 7.28 (td, J = 7.4, 1.2 Hz, 1H), 7.17 – 7.14 (m, 1H), 7.07 – 7.04 (m, 1H), 6.91 (d, J = 8.2 Hz, 1H), 5.10 (s, 2H), 2.38 (s, 3H) ppm; ^{13}C NMR (101 MHz, $CDCl_3$, δ) 152.8, 131.7, 131.5, 130.4, 130.2, 128.5, 127.7, 124.8, 123.8, 122.8, 122.1, 117.2, 68.7, 21.1 ppm.

1-(Benzyloxy)-4-methylbenzene (HP 4)

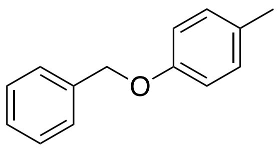

Following the procedure for **CP 4** for purification, 9.0 mg (0.045 mmol, 9%) of **HP 4** was obtained as a white solid.

Rf = 0.40 (EtOAc/PE 1:49); ^{1}H NMR (400 MHz, $CDCl_3$, δ) 7.45 – 7.31 (m, 5H), 7.11 – 7.08 (m, 2H), 6.91 – 6.87 (m, 2H), 5.05 (s, 2H), 2.30 (s, 3H) ppm; ^{13}C NMR (101 MHz, $CDCl_3$, δ) 156.9, 137.4, 130.3, 130.1, 128.7, 128.0, 127.6, 114.9, 70.2, 20.6 ppm; elemental analysis calcd (%) for $C_{14}H_{14}O$: C 84.81, H 7.12; found: C 84.59, H 7.21.

6*H*-benzo[*c*]chromene (CP 5)

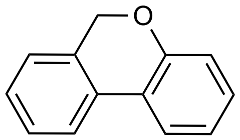

1-Iodo-2-(phenoxymethyl)benzene (31.0 mg, 0.1 mmol, 1 equiv), tributylamine (24 µL, 0.1 mmol, 1 Eq), K_2CO_3 (27.6 mg, 0.2 mmol, 2 equiv), [Fe(phtmeimb)$_2$](PF_6) (1.73 mg, 2.0 µmol, 0.02 equiv), DMSO-*d*$_6$ (2 mL) and a stir bar were combined in a 6 mL crimp vial. After closing, the suspension was purged with argon for 15 min. The suspension was subjected to 48 h of irradiation with green light (530 nm, 3.03 W) in a TAK 120 AC photoreactor under vigorous stirring. ^{1}H NMR of a 50 µL aliquot, diluted with 350 µL of DMSO-*d*$_6$ showed full consumption of the starting material (product ratio **CP 5**/**HP 5** = 68:32).

The contents of five parallel reactions were added to 100 mL EtOAc in a separatory funnel. The solution was washed with 3 x 200 mL of H_2O before extracting the combined aqueous phases with 100 mL EtOAc. The combined organic layers were washed with brine, dried over anhydrous $MgSO_4$, filtrated, and concentrated in vacuo. The crude was purified by silica gel flash chromatography (2 x 11 cm, PE), after which the mixed product fractions were subjected to preparative reverse phase HPLC (MeCN/H_2O 50:50 → 65:35 → 100:0 with 0.1% formic acid (FA) in both eluents) affording 27.0 mg (0.15 mmol, 30%) of **CP 5** as a colourless oil.

Rf = 0.34 (EtOAc/PE 1:49); ^{1}H NMR (400 MHz, $CDCl_3$, δ) 7.74 (dd, *J* = 7.7, 1.6 Hz, 1H), 7.71 (d, *J* = 7.8 Hz, 1H), 7.41 – 7.35 (m, 1H), 7.29 (td, *J* = 7.6, 1.3 Hz, 1H), 7.25 – 7.21 (m, 1H), 7.16 (d, *J* = 7.4 Hz, 1H), 7.06 (td, *J* = 7.5, 1.2 Hz, 1H), 7.00 (dd, *J* = 8.1, 1.2 Hz, 1H), 5.13 (s, 2H) ppm; ^{13}C NMR (101 MHz, $CDCl_3$, δ) 154.9, 131.6, 130.3, 129.6, 128.6, 127.8, 124.8, 123.4, 123.1, 122.3, 122.2, 117.5, 68.6 ppm; HRMS-EI: m/z calcd for $C_{13}H_{10}O$: 182.0732; found: 182.0741 [M]$^+$; elemental analysis calcd (%) for $C_{13}H_{10}O$·0.1 H_2O: C 84.85, H 5.59; found: C 84.79, H 5.58.

Benzyloxybenzene (HP 5)

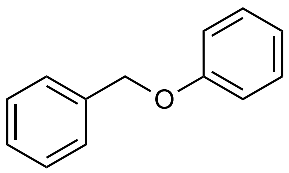

Following the procedure for **CP 5** for purification, 5.2 mg (0.028 mmol, 6%) of **HP 5** was obtained as a colourless oil.

Rf = 0.30 (EtOAc/PE 1:49); ^{1}H NMR (400 MHz, $CDCl_3$, δ) 7.47 – 7.42 (m, 2H), 7.42 – 7.36 (m, 2H), 7.35 – 7.32 (m, 1H), 7.32 – 7.27 (m, 2H), 7.00 (q, *J* = 1.2 Hz, 1H), 6.99 – 6.93 (m, 2H), 5.08 (s, 2H) ppm; ^{13}C NMR (101 MHz, $CDCl_3$, δ) 159.0, 137.2, 129.6, 128.7, 128.1, 127.6, 121.1, 115.0, 70.1 ppm; HRMS-EI: m/z calcd for $C_{13}H_{12}O$: 185.0966; found: 185.0978 [M+H]$^+$; elemental analysis calcd (%) for $C_{13}H_{12}O$·0.05 H_2O: C 84.34, H 6.59; found: C 84.21, H 6.61.

8-(*tert*-Butyl)-6*H*-benzo[*c*]chromene (CP 6A)

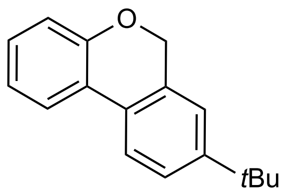

1-((4-(*tert*-Butyl)benzyl)oxy)-2-iodobenzene (36.6 mg, 0.1 mmol, 1 equiv), tributylamine (24 μL, 0.1 mmol, 1 Eq), K_2CO_3 (27.6 mg, 0.2 mmol, 2 equiv), [Fe(phtmeimb)$_2$](PF$_6$) (1.73 mg, 2.0 μmol, 0.02 equiv), DMSO-*d*$_6$ (2 mL) and a stir bar were combined in a 6 mL crimp vial. After closing, the suspension was purged with argon for 15 min. The suspension was subjected to 140 h of irradiation with green light (530 nm, 3.03 W) in a TAK 120 AC photoreactor under vigorous stirring. ^{1}H NMR of a 50 μL aliquot, diluted with 350 μL of DMSO-*d*$_6$ showed full consumption of the starting material (product ratio **CP 6A**/**CP 6B**/ **HP 6** = 77:19:4). Only traces of **HP 6** were obtained so it was not isolated. Only traces of **HP 6** were obtained so it was not isolated.

The contents of five parallel reactions were added to 100 mL EtOAc in a separatory funnel. The solution was washed with 3 x 200 mL of H_2O before extracting the combined aqueous phases with 100 mL EtOAc. The combined organic layers were washed with brine, dried over anhydrous $MgSO_4$, filtrated, and concentrated in vacuo. The crude was purified by silica gel flash chromatography (2 x 9.5 cm, 2% EtOAc in PE), after which the mixed product fractions were subjected to preparative reverse phase HPLC (MeCN/H_2O 50:50 → 65:35 → 100:0 with 0.1% formic acid (FA) in both eluents) affording 22.4 mg (0.093 mmol, 19%) of **CP1 6** as a colourless oil.

R_f = 0.34 (EtOAc/PE 1:49); ^{1}H NMR (400 MHz, $CDCl_3$) δ 7.71 (dd, *J* = 7.8, 1.6 Hz, 1H), 7.64 (d, *J* = 8.2 Hz, 1H), 7.41 (dd, *J* = 8.2, 2.1 Hz, 1H), 7.23 – 7.18 (m, 1H), 7.17 (d, *J* = 2.0 Hz, 1H), 7.08 – 7.00 (t, 1H), 6.98 (d, *J* = 8.1 Hz, 1H), 5.13 (s, 2H), 1.35 (s, 9H) ppm; ^{13}C NMR (101 MHz, $CDCl_3$) δ 154.8, 151.1, 131.2, 129.1, 127.5, 125.6, 123.2, 123.1, 122.2, 121.9, 121.8, 117.4, 69.0, 34.8, 31.4 ppm; HRMS-EI: m/z calcd for $C_{17}H_{18}O$: 239.1436; found: 239.1435 $[M+H]^+$; elemental analysis calcd (%) for $C_{17}H_{18}O\cdot0.05\ H_2O$: C 85.35, H 7.63; found: C 85.34, H 7.66.

9-(*tert*-Butyl)-6*H*-benzo[*c*]chromene (CP2 6)

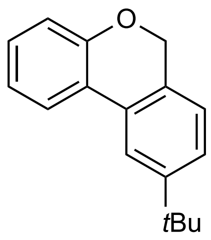

Following the procedure for **CP1 6** for purification, 4.0 mg (0.016 mmol, 3%) of **CP2 6** was obtained as a colourless oil.

R_f = 0.34 (EtOAc/PE 1:49); ^{1}H NMR (400 MHz, $CDCl_3$) δ 7.77 (d, *J* = 7.8 Hz, 0H), 7.73 (s, 1H), 7.33 (dd, *J* = 7.9, 1.9 Hz, 1H), 7.22 (d, *J* = 7.1 Hz, 1H), 7.13 – 7.04 (m, 2H), 6.99 (d, *J* = 8.1 Hz, 1H), 5.10 (s, 2H), 1.38 (s, 9H); HRMS-EI: m/z calcd for $C_{17}H_{18}O$: 239.1436; found: 239.1438 $[M+H]^+$; elemental analysis calcd (%) for $C_{17}H_{18}O\cdot0.05\ H_2O$: C 85.35, H 7.63; found: C 85.17, H 7.58.

6*H*-benzo[*c*]chromene-2-carbaldehyde (CP 7)

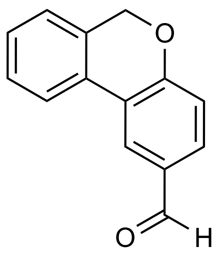

4-((2-Iodobenzyl)oxy)benzaldehyde (33.8 mg, 0.1 mmol, 1 equiv), tributylamine (24 µL, 0.1 mmol, 1 Eq), K_2CO_3 (27.6 mg, 0.2 mmol, 2 equiv), [Fe(phtmeimb)$_2$](PF$_6$) (1.73 mg, 2.0 µmol, 0.02 equiv), DMSO-d_6 (2 mL) and a stir bar were combined in a 6 mL crimp vial. After closing, the suspension was purged with argon for 15 min. The suspension was subjected to 140 h of irradiation with green light (530 nm, 3.03 W) in a TAK 120 AC photoreactor under vigorous stirring. ^{1}H NMR of a 50 µL aliquot, diluted with 350 µL of DMSO-d_6 showed almost consumption (97%) of the starting material (product ratio **CP 7**/**HP 7** = 60:40).

The contents of five parallel reactions were added to 100 mL EtOAc in a separatory funnel. The solution was washed with 3 x 200 mL of H_2O before extracting the combined aqueous phases with 100 mL EtOAc. The combined organic layers were washed with brine, dried over anhydrous $MgSO_4$, filtrated, and concentrated in vacuo. The crude was purified by silica gel flash chromatography (2 x 9.5 cm, 2% EtOAc in PE), after which the mixed product fractions were subjected to preparative reverse phase HPLC (MeCN/H_2O 50:50 → 65:35 → 100:0). The product was only isolated to confirm by ^{1}H NMR that the desired product **CP 7** was obtained. The ^{1}H NMR matches with previously reported data.[39]

^{1}H NMR (500 MHz, $CDCl_3$) δ 9.96 (s, 1H), 8.28 (d, *J* = 1.9 Hz, 1H), 7.80 (d, *J* = 7.8 Hz, 1H), 7.77 (dt, *J* = 8.3, 1.6 Hz, 1H), 7.43 (t, *J* = 7.6 Hz, 1H), 7.35 (t, *J* = 7.4 Hz, 1H), 7.18 (d, *J* = 7.4 Hz, 1H), 7.10 (d, *J* = 8.3 Hz, 1H), 5.24 (s, 2H).

4-(Benzyloxy)benzaldehyde (HP 7)

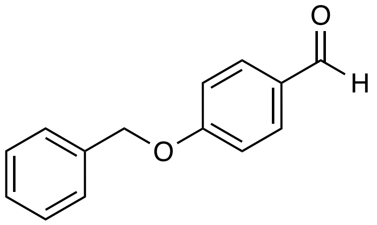

Following the procedure for **CP 7** for purification, the product was as well confirmed by ^{1}H NMR and matches with previously reported data.[46]

^{1}H NMR (500 MHz, $CDCl_3$) δ 9.89 (s, 1H), 7.87 – 7.82 (m, 2H), 7.46 – 7.38 (m, 4H), 7.38 – 7.33 (m, 1H), 7.08 (d, *J* = 8.6 Hz, 2H), 5.16 (s, 2H).

Methyl 6*H*-benzo[*c*]chromene-2-carboxylate (CP 8)

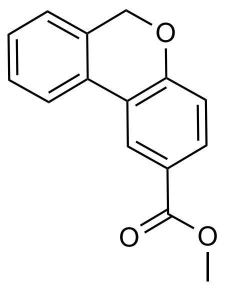

Methyl 4-((2-iodobenzyl)oxy)benzoate (36.8 mg, 0.1 mmol, 1 equiv), tributylamine (24 μL, 0.1 mmol, 1 Eq), K_2CO_3 (27.6 mg, 0.2 mmol, 2 equiv), [Fe(phtmeimb)$_2$](PF$_6$) (1.73 mg, 2.0 μmol, 0.02 equiv), DMSO-d_6 (2 mL) and a stir bar were combined in a 6 mL crimp vial. After closing, the suspension was purged with argon for 15 min. The suspension was subjected to 140 h of irradiation with green light (530 nm, 3.03 W) in a TAK 120 AC photoreactor under vigorous stirring. ^{1}H NMR of a 50 μL aliquot, diluted with 350 μL of DMSO-d_6 showed full consumption of the starting material (product ratio **CP 8**/**HP 8** = 70:30).

The contents of five parallel reactions were added to 100 mL EtOAc in a separatory funnel. The solution was washed with 3 x 200 mL of H_2O before extracting the combined aqueous phases with 100 mL EtOAc. The combined organic layers were washed with brine, dried over anhydrous $MgSO_4$, filtrated, and concentrated in vacuo. The crude was purified by silica gel flash chromatography (2 x 9.5 cm, 2% EtOAc in PE), after which the mixed product fractions were subjected to preparative reverse phase HPLC (MeCN/H_2O 50:50 → 65:35 → 100:0). The desired product **CP 8** was confirmed by the ^{1}H NMR of one chromatographic fraction which matches previously reported data.[39] **HP 8** was not obtained in pure fractions. Thus, it could not be confirmed by ^{1}H NMR.

^{1}H NMR (500 MHz, $CDCl_3$) δ 8.45 (d, J = 2.1 Hz, 1H), 7.92 (dd, J = 8.5, 2.1 Hz, 1H), 7.80 (d, J = 7.8 Hz, 1H), 7.41 (t, J = 7.6 Hz, 1H), 7.32 (t, J = 7.5 Hz, 1H), 7.16 (d, J = 7.4 Hz, 1H), 7.00 (d, J = 8.5 Hz, 1H), 5.20 (s, 2H), 3.93 (s, 3H).

2-(Trifluoromethyl)-6*H*-benzo[*c*]chromene (CP 9)

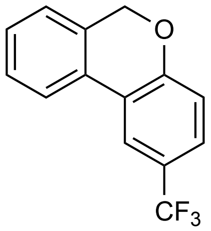

1-Iodo-2-((4-(trifluoromethyl)phenoxy)methyl)benzene (37.8 mg, 0.1 mmol, 1 equiv), tributylamine (24 μL, 0.1 mmol, 1 Eq), K_2CO_3 (27.6 mg, 0.2 mmol, 2 equiv), [Fe(phtmeimb)$_2$](PF$_6$) (1.73 mg, 2.0 μmol, 0.02 equiv), DMSO-d_6 (2 mL) and a stir bar were combined in a 6 mL crimp vial. After closing, the suspension was purged with argon for 15 min. The suspension was subjected to 48 h of irradiation with green light (530 nm, 3.03 W) in a TAK 120 AC photoreactor under vigorous stirring. ^{1}H NMR of a 50 μL aliquot, diluted with 350 μL of DMSO-d_6 showed full consumption of the starting material (product ratio **CP 9**/**HP 9** = 67:33).

The contents of six parallel reactions were added to 100 mL EtOAc in a separatory funnel. The solution was washed with 3 x 200 mL of H_2O before extracting the combined aqueous phases with 100 mL EtOAc. The combined organic layers were washed with brine, dried over anhydrous $MgSO_4$, filtrated, and concentrated in vacuo. The crude was purified by silica gel flash chromatography (2 x 9.5 cm, 2% EtOAc in PE), after which the mixed product fractions were subjected to preparative reverse phase HPLC (MeCN/H_2O 50:50 → 65:35 → 100:0 with 0.1% formic acid (FA) in both eluents) affording 17.1 mg (0.068 mmol, 11%) of **CP 9** as a white solid.

^{1}H NMR (400 MHz, $CDCl_3$) δ 7.97 (d, J = 2.3 Hz, 1H), 7.73 (d, J = 7.8 Hz, 1H), 7.48 (d, J = 8.1 Hz, 1H), 7.42 (t, J = 7.6 Hz, 1H), 7.34 (t, J = 7.5 Hz, 1H), 7.18 (d, J = 7.6 Hz, 1H), 7.06 (d, J = 8.6 Hz, 1H), 5.18 (s, 2H). R_f =

0.33 (EtOAc/PE 1:19); HRMS-EI: m/z calcd for $C_{14}H_9F_3O$: 250.0605; found: 250.0596 $[M]^+$; elemental analysis calcd (%) for $C_{14}H_{11}F_3O\cdot0.15\ H_2O$: C 66.48, H 3.71; found: C 66.89, H 3.65.

1-(Benzyloxy)-4-(trifluoromethyl)benzene (HP 9)

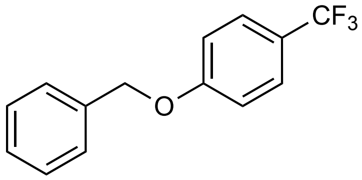

Following the procedure for **CP 9** for purification, 11.5 mg (0.016 mmol, 8%) of **HP 9** was obtained as a white solid.

^1H NMR (400 MHz, $CDCl_3$) δ 7.55 (d, *J* = 8.5 Hz, 2H), 7.46 – 7.30 (m, 5H), 7.03 (d, *J* = 8.5 Hz, 2H), 5.11 (s, 2H). R_f = 0.28 (EtOAc/PE 1:19); HRMS-EI: m/z calcd for $C_{14}H_{11}F_3O$ 253.0840; found: 253.0856 $[M+H]^+$; elemental analysis calcd (%) for $C_{14}H_{11}F_3O\cdot0.05\ H_2O$: C 66.43, H 4.42; found: C 66.31, H 4.48.

2-(*tert*-Butyl)-6*H*-naphtho[2,1-*c*]chromene (CP 11)

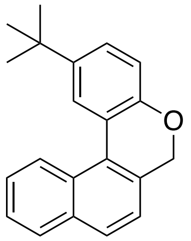

1-Bromo-2-((4-(*tert*-butyl)phenoxy)methyl)naphthalene (36.9 mg, 0.1 mmol, 1 equiv), tributylamine (24 µL, 0.1 mmol, 1 Eq), K_2CO_3 (27.6 mg, 0.2 mmol, 2 equiv), $[Fe(phtmeimb)_2](PF_6)$ (1.73 mg, 2.0 µmol, 0.02 equiv), DMSO-d_6 (2 mL) and a stir bar were combined in a 6 mL crimp vial. After closing, the suspension was purged with argon for 15 min. The suspension was subjected to 48 h of irradiation with green light (530 nm, 3.03 W) in a TAK 120 AC photoreactor under vigorous stirring. ^1H NMR of a 50 µL aliquot, diluted with 350 µL of DMSO-d_6 showed full consumption of the starting material (product ratio **CP 11**/**HP 11** = 87:13).

The contents of four parallel reactions were added to 100 mL EtOAc in a separatory funnel. The solution was washed with 3 x 200 mL of H_2O before extracting the combined aqueous phases with 100 mL EtOAc. The combined organic layers were washed with brine, dried over anhydrous $MgSO_4$, filtrated, and concentrated in vacuo. The crude was purified by silica gel flash chromatography (2 x 10 cm, PE → EtOAc/PE 1:49), after which the mixed product fractions were subjected to preparative reverse phase HPLC (MeCN/H_2O 50:50 → 65:35 → 100:0 with 0.1% formic acid (FA) in both eluents) affording 20.9 mg (0.072 mmol, 18%) of **CP 11** as a white solid.

R_f = 0.34 (EtOAc/PE 1:24); ^1H NMR (400 MHz, $CDCl_3$, δ) 8.59 – 8.56 (m, 1H), 8.08 (d, *J* = 2.4 Hz, 1H), 7.92 – 7.89 (m, 1H), 7.78 (d, *J* = 8.2 Hz, 1H), 7.59 – 7.49 (m, 2H), 7.35 – 7.30 (m, 2H), 7.10 (d, *J* = 8.4 Hz, 1H), 5.13 (s, 2H), 1.42 (s, 9H) ppm; ^{13}C NMR (101 MHz, $CDCl_3$, δ) 154.4, 144.5, 134.7, 133.0, 129.3, 129.2, 128.0, 127.6, 126.9, 125.8, 125.7, 125.3, 123.7, 122.7, 116.9, 70.2, 34.7, 31.8 ppm; elemental analysis calcd (%) for $C_{21}H_{20}O$: C 87.46, H 6.99; found: C 87.23, H 7.06.

2-((4-(*tert*-Butyl)phenoxy)methyl)naphthalene (HP 11)

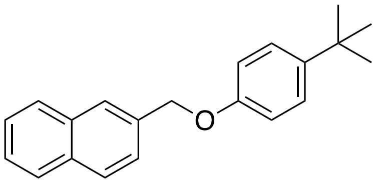

Following the procedure for **CP 11** for purification, 3.6 mg (0.012 mmol, 3%) of **HP 11** was obtained as a white solid.

R_f = 0.29 (EtOAc/PE 1:24); ^{1}H NMR (400 MHz, $CDCl_3$, δ) 7.89 – 7.83 (m, 4H), 7.54 (dd, J = 8.4, 1.7 Hz, 1H), 7.51 – 7.46 (m, 2H), 7.33 – 7.30 (m, 2H), 6.98 – 6.94 (m, 2H), 5.22 (s, 2H), 1.30 (s, 9H) ppm. ^{13}C NMR (101 MHz, $CDCl_3$, δ) 156.7, 143.8, 135.0, 133.5, 133.2, 128.5, 128.1, 127.9, 126.4, 126.4, 126.3, 126.2, 125.5, 114.5, 70.3, 34.2, 31.7 ppm; HRMS-EI; m/z calcd for $C_{21}H_{22}O$: 291.1749; found: 291.1756 $[M+H]^+$ elemental analysis calcd (%) for $C_{21}H_{22}O \cdot 0.1\ H_2O$: C 86.32, H 7.66; found: C 86.41, H 7.67.

2-(*tert*-Butyl)-8-(trifluoromethyl)-6*H*-benzo[*c*]chromene (CP 12)

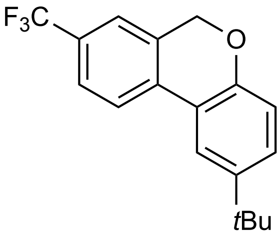

2-((4-(*tert*-Butyl)phenoxy)methyl)-1-iodo-4-(trifluoromethyl)benzene (43.4 mg, 0.1 mmol, 1 equiv), tributylamine (24 μL, 0.1 mmol, 1 Eq), K_2CO_3 (27.6 mg, 0.2 mmol, 2 equiv), $[Fe(phtmeimb)_2](PF_6)$ (1.73 mg, 2.0 μmol, 0.02 equiv), DMSO-d_6 (2 mL) and a stir bar were combined in a 6 mL crimp vial. After closing, the suspension was purged with argon for 15 min. The suspension was subjected to 48 h of irradiation with green light (530 nm, 3.03 W) in a TAK 120 AC photoreactor under vigorous stirring. ^{1}H NMR of a 50 μL aliquot, diluted with 350 μL of DMSO-d_6 showed full consumption of the starting material (product ratio **CP 12**/**HP 12** = 9:1).

The contents of five parallel reactions were added to 100 mL EtOAc in a separatory funnel. The solution was washed with 3 x 200 mL of H_2O before extracting the combined aqueous phases with 100 mL EtOAc. The combined organic layers were washed with brine, dried over anhydrous $MgSO_4$, filtrated, and concentrated in vacuo. The crude was purified by silica gel flash chromatography (2 x 10 cm, PE → EtOAc/PE 1:49), after which the mixed product fractions were subjected to preparative reverse phase HPLC (MeCN/H_2O 50:50 → 65:35 → 100:0 with 0.1% formic acid (FA) in both eluents) affording 71.0 mg (0.232 mmol, 47%) of **CP 12** as a colourless oil.

R_f = 0.44 (EtOAc/PE 1:19); ^{1}H NMR (400 MHz, $CDCl_3$, δ) 7.81 (d, J = 8.1 Hz, 1H), 7.75 (d, J = 2.4 Hz, 1H), 7.62 (d, J = 8.1 Hz, 1H), 7.41 (s, 1H), 7.35 (dd, J = 8.5, 2.4 Hz, 1H), 6.96 (d, J = 8.6 Hz, 1H), 5.13 (s, 2H), 1.38 (s, 9H) ppm; ^{13}C NMR (101 MHz, $CDCl_3$) δ 153.1, 145.4, 134.3, 132.1, 128.1, 125.4 (q, J = 3.9 Hz), 122.3, 121.9 (q, J = 3.8 Hz), 120.4, 117.3, 68.2, 34.6, 31.6 ppm; HRMS-EI: m/z calcd for $C_{18}H_{17}F_3O$: 307.1310; found: 307.1263 $[M+H]^+$, elemental analysis calcd (%) for $C_{18}H_{17}F_3O \cdot 0.25\ H_2O$: C 69.55, H 5.67; found: C 69.41, H 5.87.

1-((4-(*tert*-Butyl)phenoxy)methyl)-3 (trifluoromethyl)benzene (HP 12)

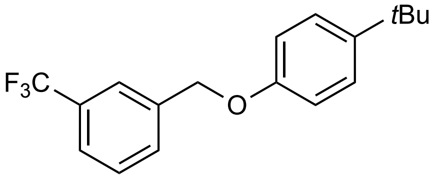

Following the procedure for **CP 12** for purification, 15.0 mg (0.049 mmol, 10%) of **HP 12** was obtained as a colorless oil.

R_f = 0.47 (EtOAc/PE 1:19); ^{1}H NMR (400 MHz, $CDCl_3$) δ 7.71 (s, 1H), 7.63 (d, *J* = 7.7 Hz, 1H), 7.58 (d, *J* = 7.9 Hz, 1H), 7.50 (t, *J* = 7.7 Hz, 1H), 7.36 – 7.29 (m, 2H), 6.92 (d, *J* = 8.7 Hz, 2H), 5.09 (s, 2H), 1.31 (s, 9H) ppm; ^{13}C NMR (101 MHz, $CDCl_3$) δ 156.4, 144.2, 138.5, 130.8, 129.2, 126.5, 124.9, 124.2, 114.4, 69.4, 34.3, 31.7 ppm; HRMS-EI; m/z calcd for $C_{18}H_{19}F_3O$: 309.1466; found: 309.1472 $[M+H]^+$; elemental analysis calcd (%) for $C_{18}H_{19}F_3O$: C 70.12, H 6.21; found: C 70.04, H 6.19.

Literature studies of oxidative quenching

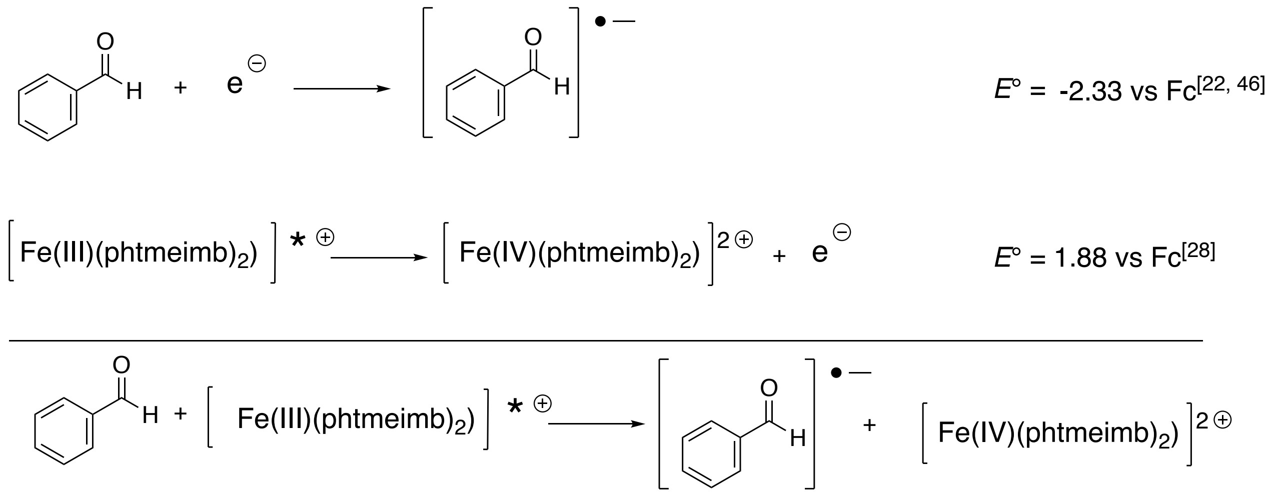

$\Delta E° = -0.45$ vs Fc; $\Delta G° = \{-nF\Delta E°\} = -1 \times 96485 \times (-0.45)$ J/mol = 43 kJmol > 0

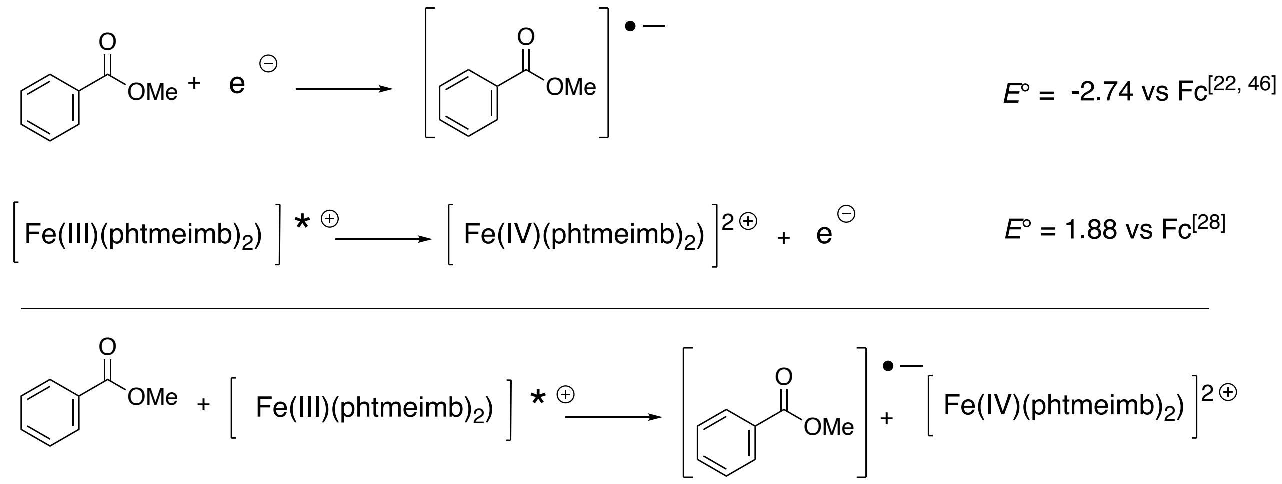

$\Delta E° = -0.86$ vs Fc; $\Delta G° = \{-nF\Delta E°\} = -1 \times 96485 \times (-0.86)$ J/mol = 83 kJ/mol > 0

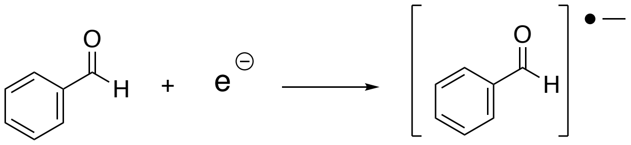

$E°$ = -2.33 vs Fc[22, 46]

$[\mathrm{W(0)(CNDippPh^{OMe3})_3}]^* \longrightarrow [\mathrm{W(I)(CNDippPh^{OMe3})_3}]^{\oplus} + e^{\ominus}$

$E°$ = 2.68 vs Fc[15]

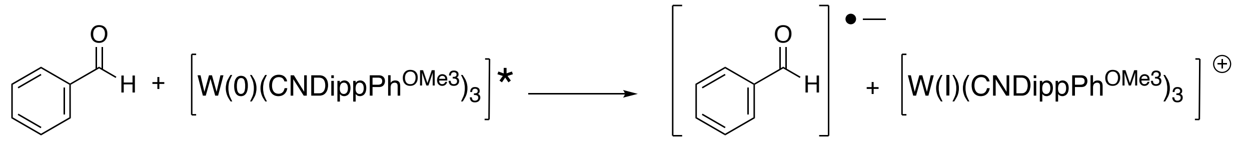

$\Delta E° =$ 0.35 vs Fc; $\Delta G° = \{-nF\Delta E°\} = -1 \times 96485 \times (0.35)$ J/mol = -37 kJ/mol < 0

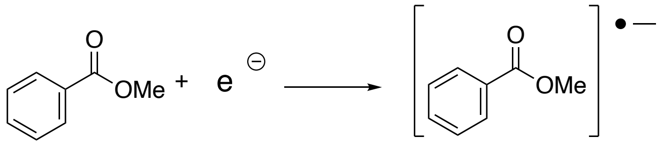

$E°$ = -2.74 vs Fc[22, 46]

$[\mathrm{W(0)(CNDippPh^{OMe3})_3}]^* \longrightarrow [\mathrm{W(I)(CNDippPh^{OMe3})_3}]^{\oplus} + e^{\ominus}$

$E°$ = 2.68 vs Fc[15]

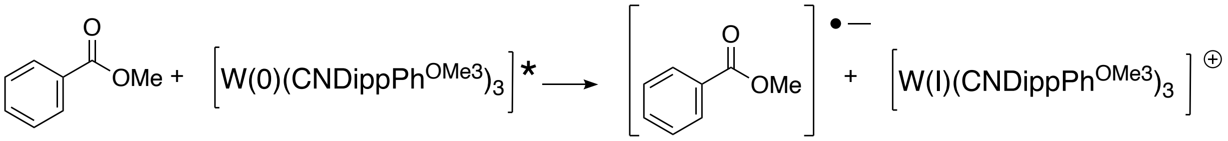

$\Delta E° =$ -0.06 vs Fc; $\Delta G° = \{-nF\Delta E°\} = -1 \times 96485 \times (-0.06)$ J/mol = 6 kJ/mol ≈ 0

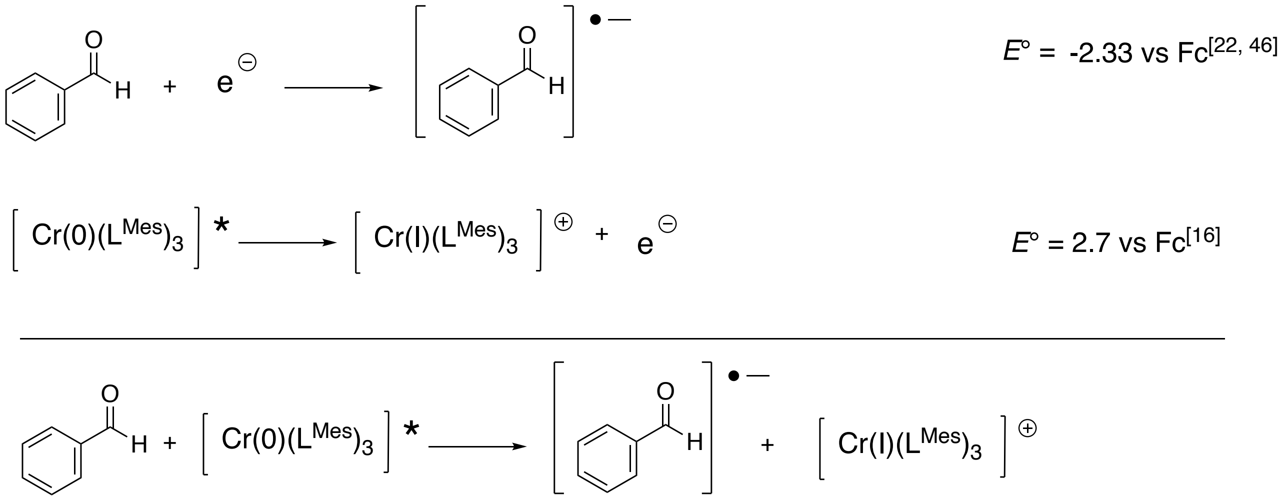

$\Delta E° =$ 0.47 vs Fc; $\Delta G° = \{-nF\Delta E°\}$ = -1 x 96485 x (0.47) J/mol = -45 kJ/mol < 0

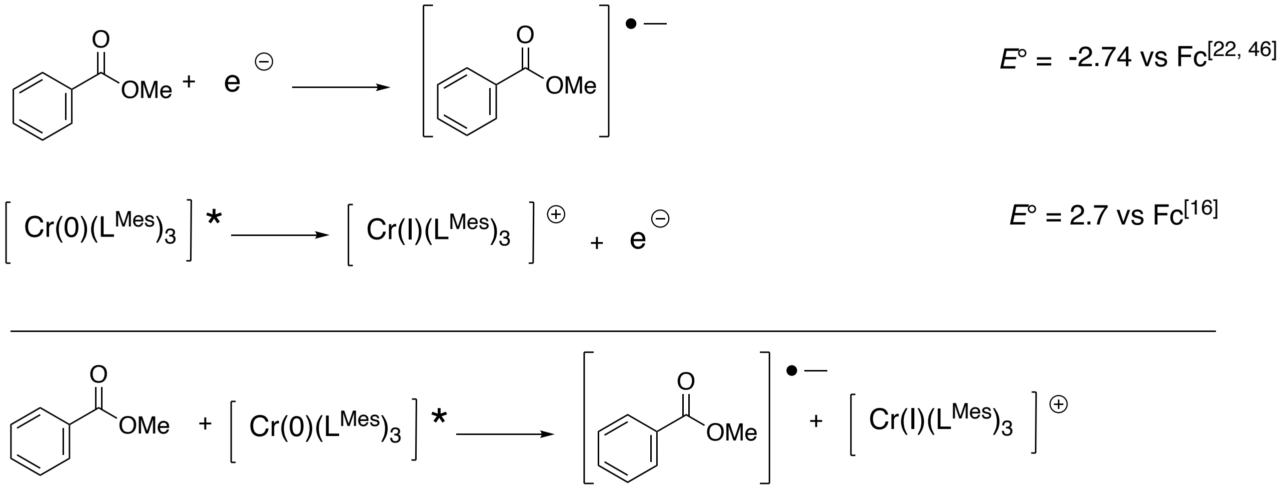

$\Delta E° =$ -0.04 vs Fc; $\Delta G° = \{-nF\Delta E°\}$ = -1 x 96485 x (-0.04) J/mol = 4 kJ/mol ≈ 0

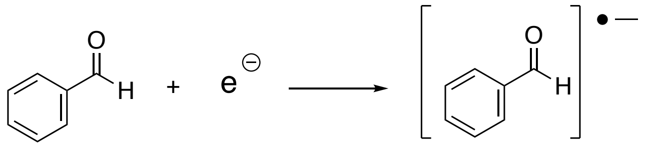

$E° = -2.33$ vs Fc[22, 46]

$$[\mathrm{Mo(0)(L^{\mathit{t}Bu})_3}]^* \longrightarrow [\mathrm{Mo(I)(L^{\mathit{t}Bu})_3}]^{\oplus} + e^{\ominus}$$

$E° = 2.7$ vs Fc[15, 22]

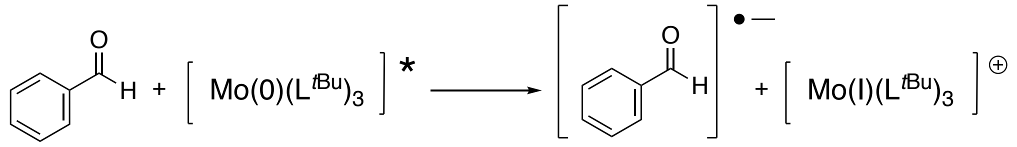

$\Delta E° = 0.37$ vs Fc; $\Delta G° = \{-nF\Delta E°\} = -1 \times 96485 \times (0.37)$ J/mol = -36 kJ/mol < 0

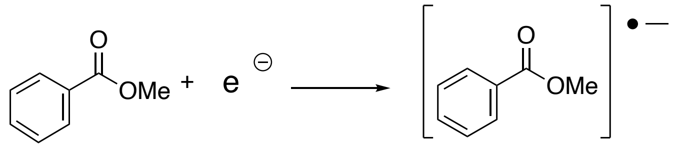

$E° = -2.74$ vs Fc[22, 46]

$$[\mathrm{Mo(0)(L^{\mathit{t}Bu})_3}]^* \longrightarrow [\mathrm{Mo(I)(L^{\mathit{t}Bu})_3}]^{\oplus} + e^{\ominus}$$

$E° = 2.7$ vs Fc[15, 22]

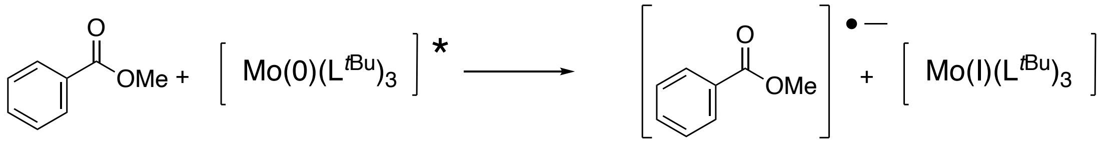

$\Delta E° = -0.04$ vs Fc; $\Delta G° = \{-nF\Delta E°\} = -1 \times 96485 \times (-0.04)$ J/mol = 4 kJ/mol ≈ 0

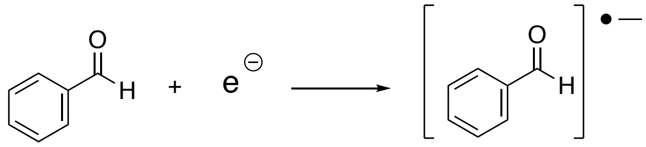

$E°$ = -2.33 vs Fc[22, 46]

$TEA^{\bullet} \longrightarrow TEA^{\oplus} + e^{\ominus}$

$E°$ = 2.1 vs Fc[22, 30]

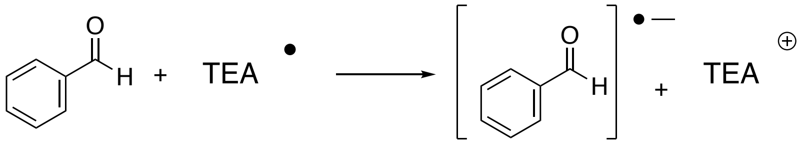

$\Delta E°$ = - 0.23 vs Fc; $\Delta G°$ = {-nF$\Delta E°$}= -1 x 96485 x (-0.23) J/mol = 21 kJ/mol > 0

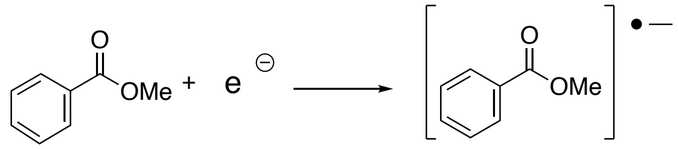

$E°$ = -2.74 vs Fc[22, 46]

$TEA^{\bullet} \longrightarrow TEA^{\oplus} + e^{\ominus}$

$E°$ = 2.1 vs Fc[22, 30]

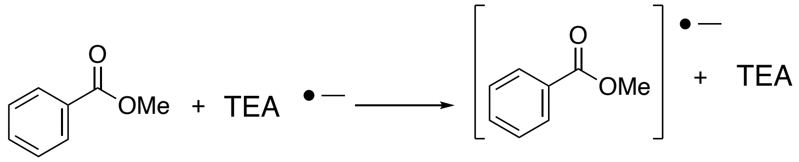

$\Delta E°$ = -0.64 vs Fc; $\Delta G°$ ={ -nF$\Delta E°$} = -1 x 96485 x (-0.64) J/mol = 61 kJ/mol > 0

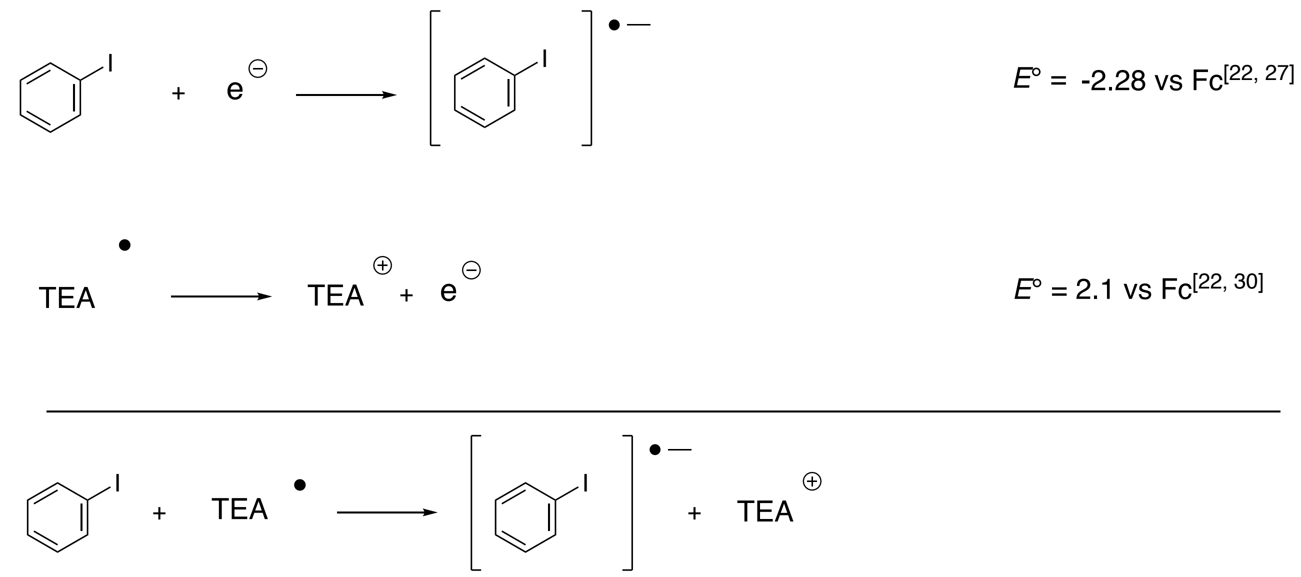

$\Delta E° = -0.18$ vs Fc; $\Delta G° = \{-nF\Delta E°\} = -1 \times 96485 \times (-0.18)$ J/mol = 17 kJ/mol > 0

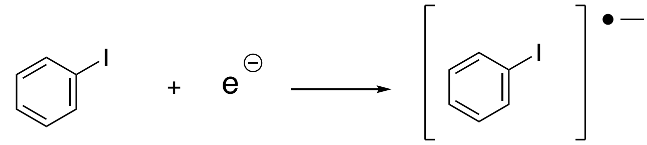

$E°$ = -2.28 vs Fc[22, 27]

$$[\text{Fe(II)(phtmeimb)}_2] \longrightarrow [\text{Fe(III)(phtmeimb)}_2]^{*\oplus} + e^{\ominus}$$

$E°$ = 0.97 vs Fc[28]

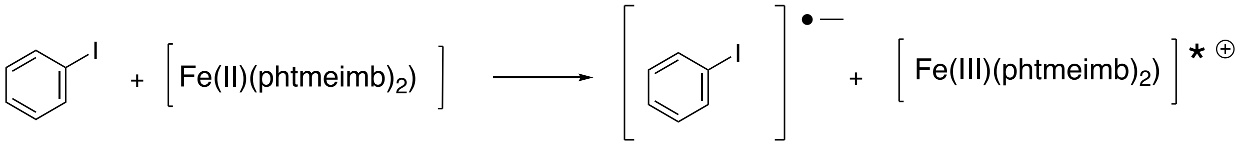

$\Delta E°$ = - 1.31 vs Fc; $\Delta G°$ = $\{-nF\Delta E°\}$ = -1 x 96485 x (-1.31) J/mol = 126 kJ/mol > 0

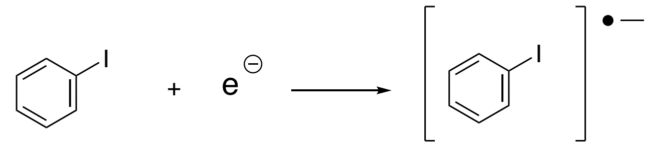

$E°$ = -2.28 vs Fc[22, 27]

$$[\text{Fe(II)(phtmeimb)}_2] \longrightarrow [\text{Fe(III)(phtmeimb)}_2]^{\oplus} + e^{\ominus}$$

$E°$ = 1.16 vs Fc[28]

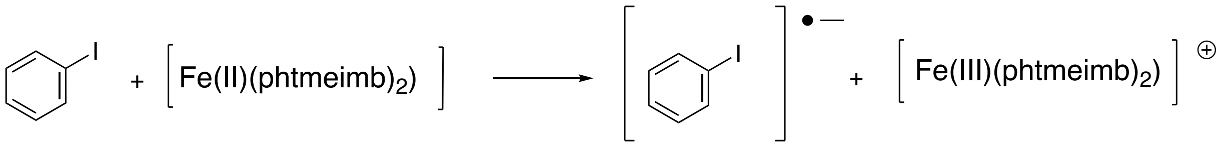

$\Delta E°$ = - 1.12 vs Fc; $\Delta G°$ = $\{-nF\Delta E°\}$ = -1 x 96485 x (-1.12) J/mol = 108 kJ/mol > 0

Mechanistic Investigations

Radical scavenging experiment

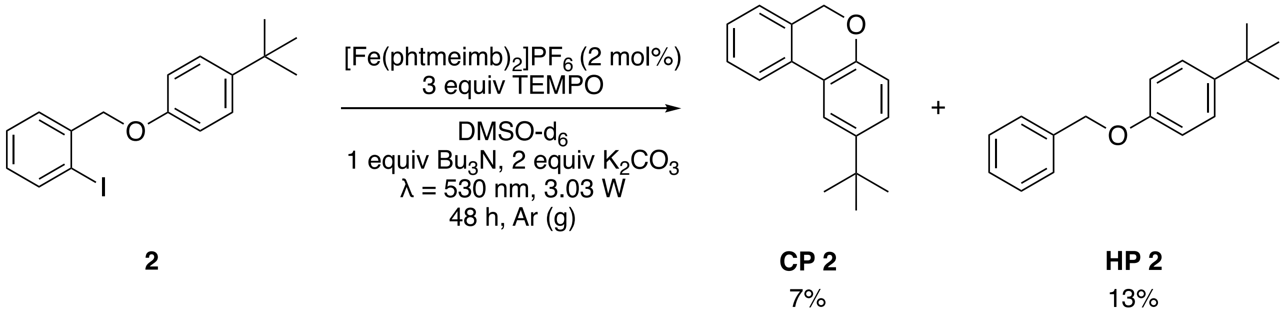

Upon addition of TEMPO (2,2,6,6-tetramethylpiperidine-1-oxyl) (3 equiv) to a reaction system following the general procedure with 1-((4-(*tert*-butyl)phenoxy)methyl)-2-iodobenzene (substrate **2**), the reactivity was severely reduced as only 20% conversion (7% **CP 2** and 13% **HP 2**) was observed by ^{1}H NMR analysis. This provides support for a radical pathway being present in the reaction mechanism.

Quantum yield measurements (actinometry)

The quantum yield for the BHAS reaction was determined using a method developed by Pitre et al.,[47] wherein the oxidation of 9,10-diphenylanthracene (DPA), catalyzed by $[Ru(bpy)_3]Cl_2$ in acetonitrile was used to quantify the number of moles of photons absorbed by the sample. All samples were irradiated for 1 min in the same slot at 530 nm (3.03 W/slot) in a TAK120 AC photoreactor purchased from HK Testsysteme GmbH. UV-Vis absorption measurements were performed on a Probe Drum Lab-in-a-box spectrometer and samples were analyzed in a 10 mm quartz cuvette.

Two duplicate reaction solutions (2 mL) with 0.6 mM $[Ru(bpy)_3]Cl_2 \cdot 6\ H_2O$ and 0.1 mM DPA in acetonitrile in 6 mL crimp vials were irradiated for 1 min. All reaction solutions were prepared under exclusion of light. Samples for UV-Vis spectroscopy were stored in amber glass vials before transferring to the cuvette. Due to the comparatively low absorptivity of $[Ru(bpy)_3]Cl_2 \cdot 6\ H_2O$ at 530 nm, its concentration was not adapted to match the absorbance of the Fe-PC in the reaction system. Instead, a correction factor was introduced to account for the difference in incident photon absorption.

Eq 1.–Eq. 4 were used to calculate the quantum yield of the BHAS reaction (Φ_{BHAS}). Figure S1 shows the absorption spectra obtained for the two replicates before and after irradiation. Table S10–S12 show the obtained experimental values and the calculated values.

$$n \text{ (moles of DPA consumed)} = \left(\frac{A_{initial} - A_{final}}{\varepsilon_{372nm} l}\right) \cdot V \qquad \text{(Eq. 1)}$$

$$f = 1 - 10^{-(Abs)} \qquad \text{(Eq. 2)}$$

$$\frac{Nh\nu}{t} = \frac{n \text{ (moles of DPA consumed)}}{\Phi_{actinometer} t} \cdot \frac{f \text{ (BHAS reaction)}}{f \text{ (Ru –actinometer solution)}} \qquad \text{(Eq. 3)}$$

$$\Phi_{BHAS} = \frac{n \text{ (moles of BHAS product formed)}}{t} \cdot \left(\frac{Nh\nu}{t}\right)^{-1} \qquad \text{(Eq. 4)}$$

- $A_{initial}$ = absorbance of the solution at 327 nm before irradiation

- A_{final} = absorbance of the solution at 327 nm after 1 min of irradiation
- ε_{327nm} = molar extinction coefficient of DPA at 327 nm in acetonitrile (11100 $M^{-1}cm^{-1}$)
- l = path length of the cuvette (cm)
- V = volume (L)
- $\frac{Nh\nu}{t}$ = moles of absorbed photons by sample per time unit
- $\Phi_{actinometer}$ = quantum yield of the actinometer (0.019)
- Φ_{BHAS} = quantum yield of the BHAS reaction
- f = fraction of incident photons absorbed = correction factor
- Abs = absorbance at 530 nm

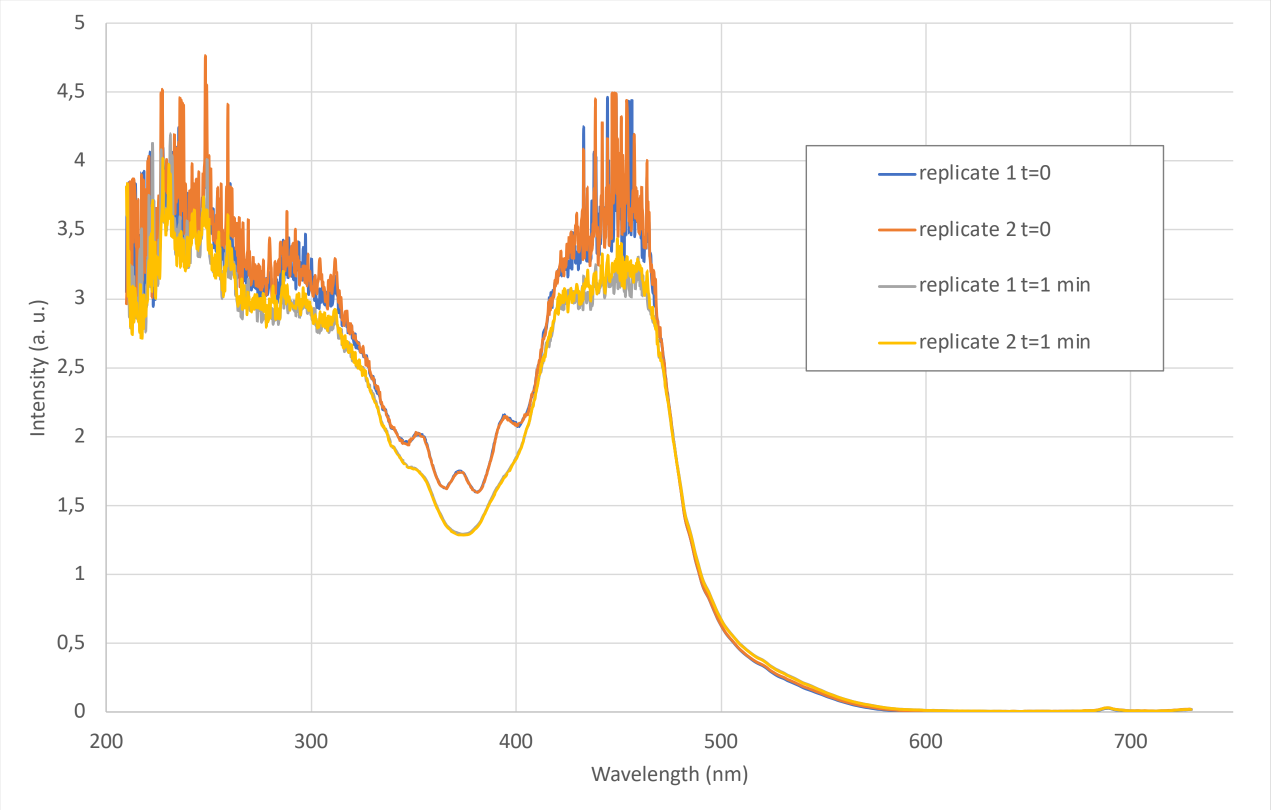

Figure S1. Absorption spectra for the oxidation of DPA using $[Ru(bpy)_3]Cl_2 \cdot 6\ H_2O$.

Table S10. Obtained values used for calculation of n (moles of DPA consumed).

	Replicate 1	Replicate 2
$A_{initial}$	1.7527	1.7467
A_{final}	1.2969	1.2879
ε_{327nm}	11100	11100
l	1	1
V	0.0015	0.0015
n (moles of DPA consumed)	$6.16 \cdot 10^{-8}$	$6.20 \cdot 10^{-8}$

Table S11. Obtained values used for calculation of f (correction factor).

	0.6 mM $[Ru(bpy)_3]Cl_2 \cdot 6\ H_2O$	1 mM $[Fe(phtmeimb)_2](PF_6)$ in DMSO
Abs	0.42	2.82
f	0.616	0.998
f(BHAS)/f(Ru-actinometer)	1.622	

Table S12. Obtained values used for calculation of $\frac{Nh\nu}{t}$.

	Replicate 1	Replicate 2
n (moles of DPA consumed)	$6.16 \cdot 10^{-8}$	$6.20 \cdot 10^{-8}$
$\Phi_{actinometer}$	0.019	0.019
t (min)	1	1
f (BHAS reaction	0.998	0.998
f (Ru-actinometer)	0.616	0.616
$\frac{Nh\nu}{t}$	$5.26 \cdot 10^{-6}$	$5.29 \cdot 10^{-6}$

Table S13. Obtained values used for calculation of Φ_{BHAS}.

	Replicate 1	Replicate 2
n (moles of BHAS product formed)	$3.10 \cdot 10^{-5}$	$1.85 \cdot 10^{-5}$
t (minutes)	1320	1320
$\frac{Nh\nu}{t}$	$5.26 \cdot 10^{-6}$	$5.29 \cdot 10^{-6}$
Φ_{BHAS}	0.004	0.003

As shown in Table S13, the quantum yield for the BHAS reaction was determined to be 0.3–0.4%, which is in line with the quenching being inefficient and the experimentally observed long reaction times.

Cyclic voltammetry measurements

Cyclic voltammograms of the substrates feature irreversible reduction waves close to -3 V *vs.* Fc with corresponding DPV peaks at about -2.8 V. Under the same conditions, essential identical reductive voltammograms are observed with iodobenzene, and the reduction of the substrates can be assigned to their aryl halide moiety. While the irreversible voltammograms cannot provide correct thermodynamic potentials, the electrochemical data shows that reduction of the substrates is occuring at the samepotential as with iodobenzene itself, for which a potential of about -1.9 V *vs.* SCE (-2.2 V *vs.* Fc) is commonly accepted.

For substrates **1**, **2**, and **6**, oxidative scans of the voltammograms show anodic peaks around 0.9, 1.2 and 1.4 V vs. Fc, respectively. With the uncertainties associated to the irreversible nature of the electrode reactions, oxidation by the excited photocatalyst ($E°$(*III/II) = 0.97 V vs $Fc^{+/0}$) can therefore not be excluded on the basis of these potentials, at least for substrates **1** and probably **2**.

Substrate 1

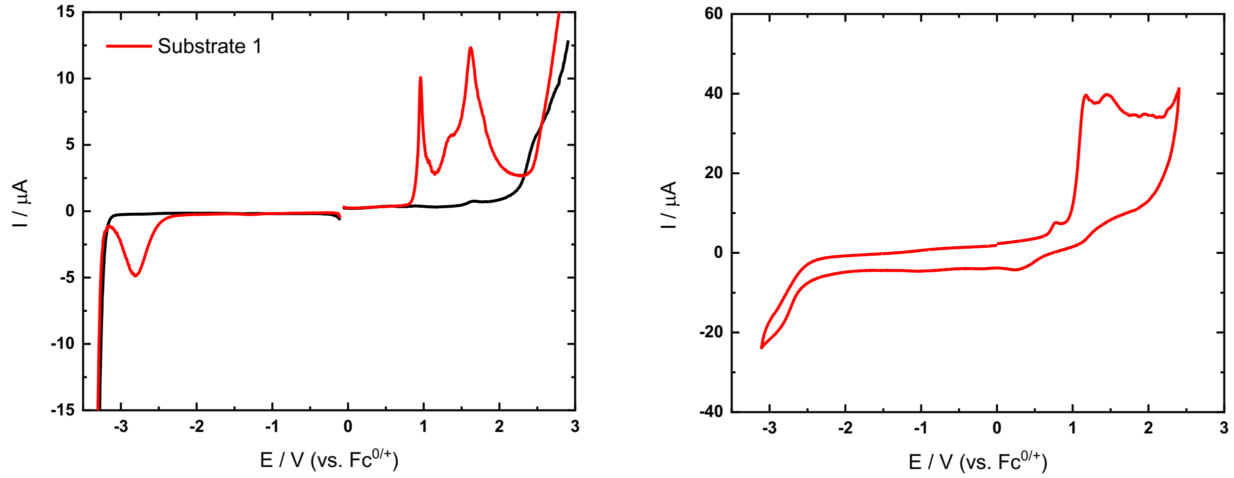

Figure S2. Differential pulse voltammograms (left) (step potential: 5 mV, modulation amplitude: 25 mV, modulation time: 50 ms, interval time: 100 ms) and cyclic voltammogram (right) (scan rate: 0.05 Vs^{-1}) of 1 mM of substrate **1** in acetonitrile and 0.1 M $TBAPF_6$ as the electrolyte.

Substrate 2

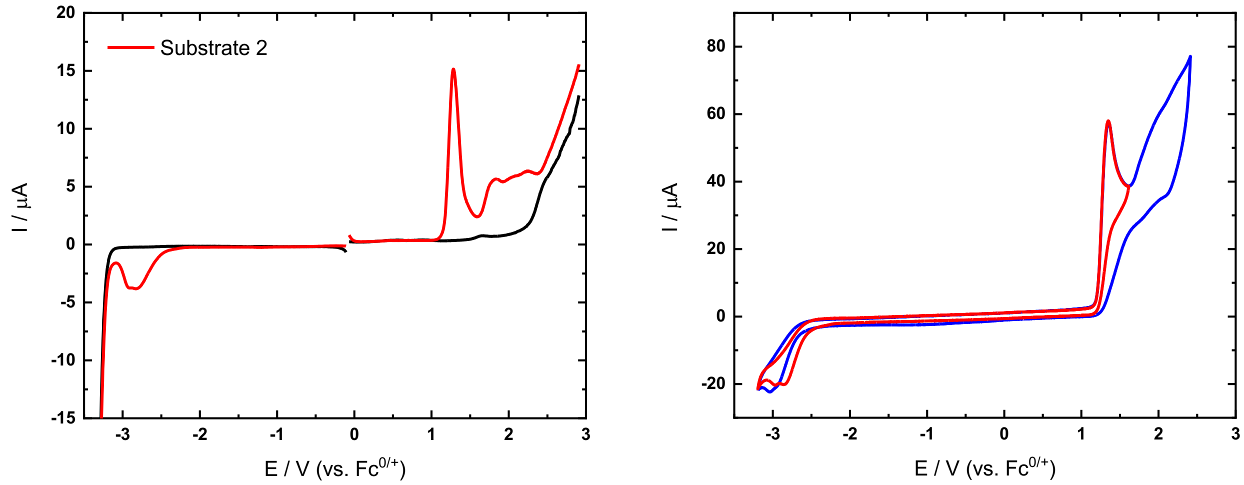

Figure S3. Differential pulse voltammograms (left) (step potential: 5 mV, modulation amplitude: 25 mV, modulation time: 50 ms, interval time: 100 ms) and cyclic voltammograms (right) (scan rate: 0.05 Vs^{-1}) of 1 mM of substrate **2** in acetonitrile and 0.1 M $TBAPF_6$ as the electrolyte.

Substrate 6

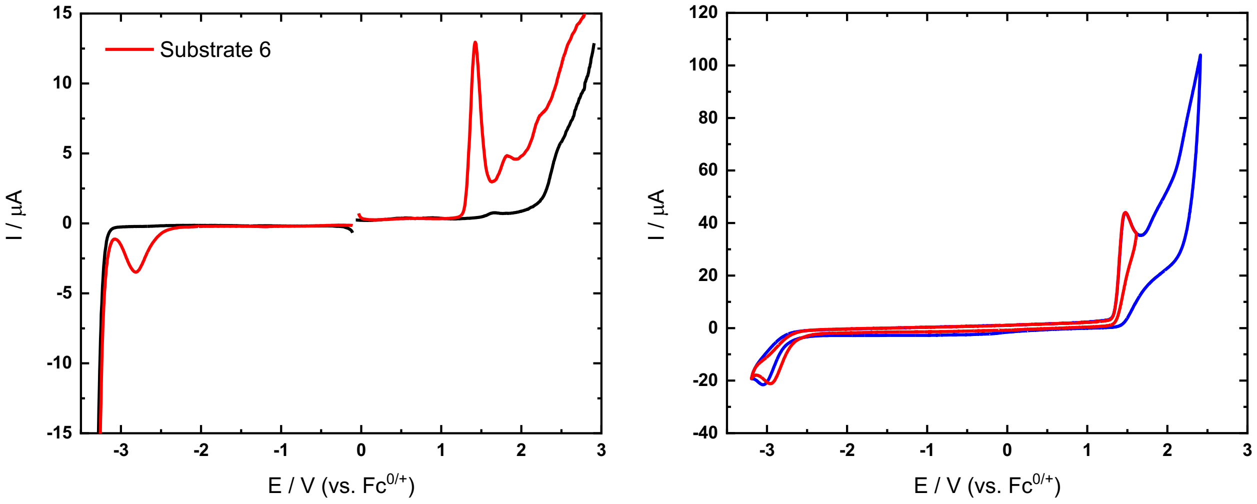

Figure S4. Differential pulse voltammograms (left) (step potential: 5 mV, modulation amplitude: 25 mV, modulation time: 50 ms, interval time: 100 ms) and cyclic voltammograms (right) (scan rate: 0.05 Vs^{-1}) of 1 mM of substrate **6** in acetonitrile and 0.1 M $TBAPF_6$ as the electrolyte.

Iodobenzene

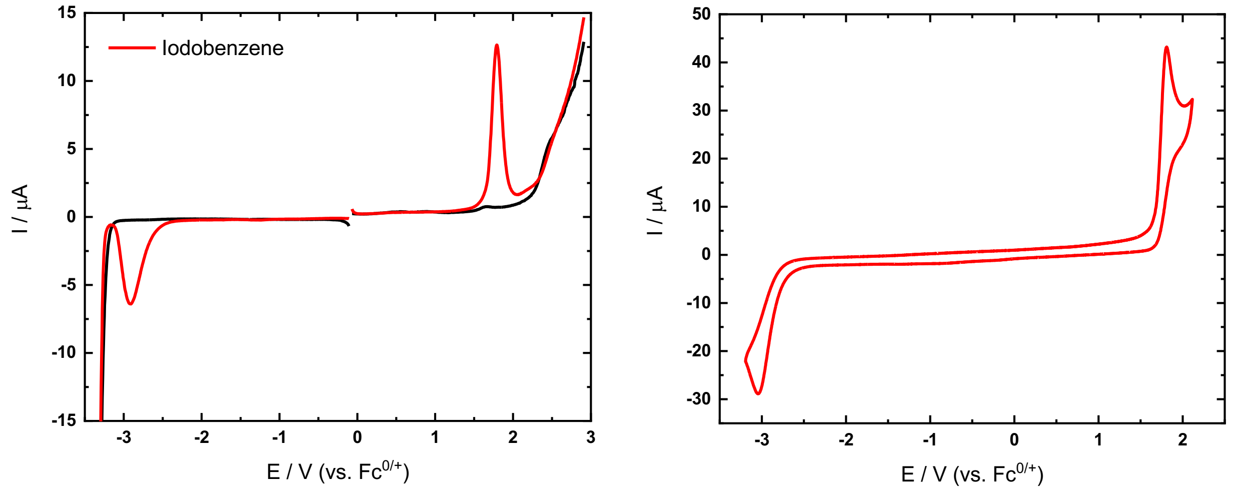

Figure S5. Differential pulse voltammograms (left) (step potential: 5 mV, modulation amplitude: 25 mV, modulation time: 50 ms, interval time: 100 ms) and cyclic voltammogram (right) (scan rate: 0.05 Vs^{-1}) of 1 mM of iodobenzene in acetonitrile and 0.1 M $TBAPF_6$ as the electrolyte.

Excited state lifetime of $[Fe(phtmeimb)_2](PF_6)$ in DMSO

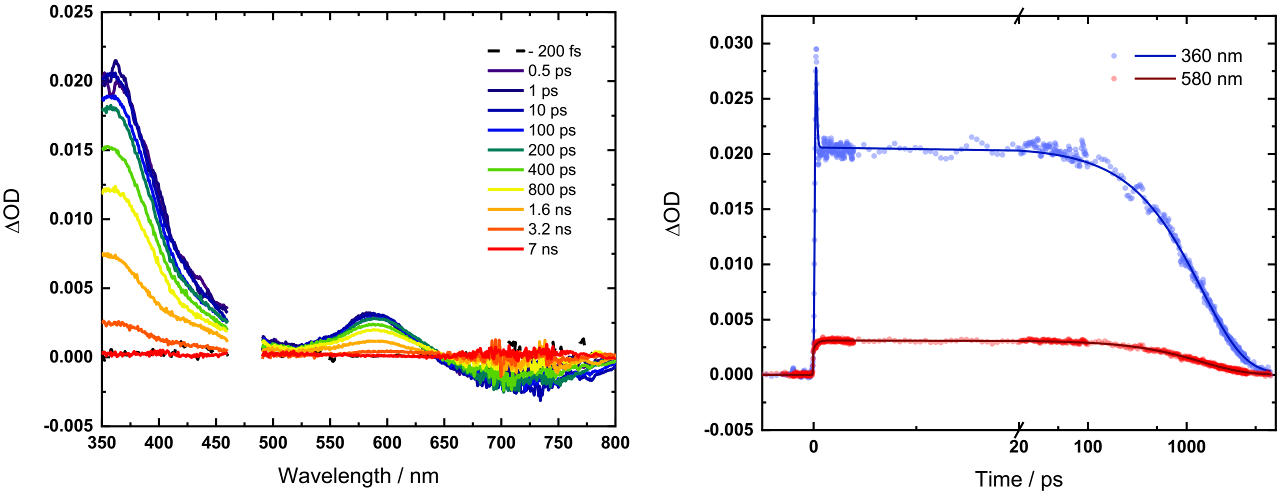

Figure S6. Transient absorption measurement of $[Fe(phtmeimb)_2]PF_6$ in DMSO solution. Left: Transient absorption spectra at indicated time delays after photoexcitation (λ_{ex} = 475 nm, E = 3.0 ± 0.2 mW). Right: Transient absorption kinetics at indicated wavelengths, τ = 1.43 ns.

Excited state quenching

Substrates 1, 2 and 6

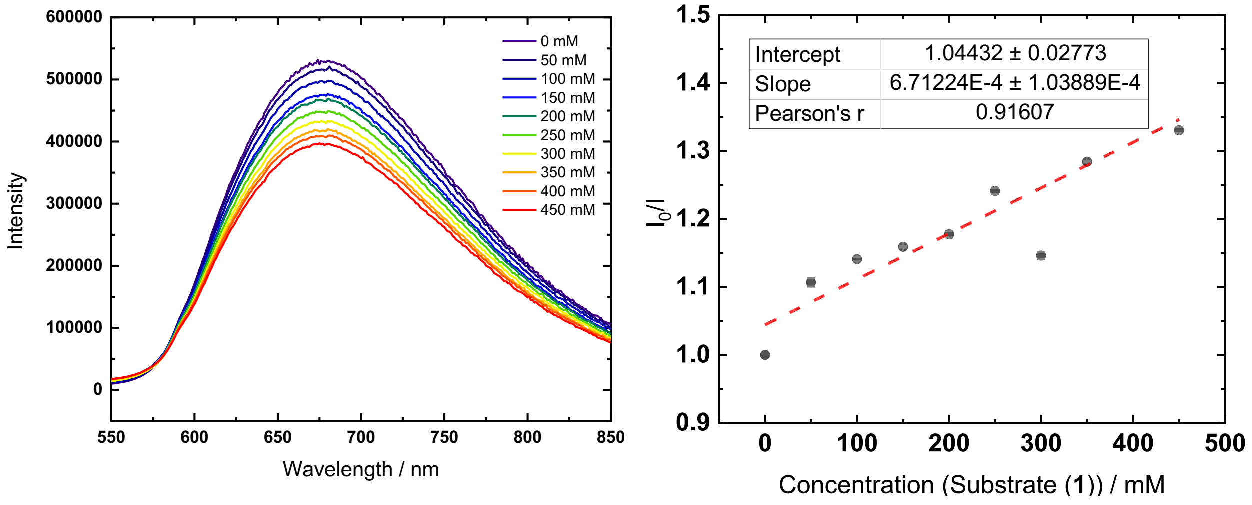

Figure S7. Emission spectra (left) and Stern-Volmer plot (right) of $[Fe(phtmeimb)_2]PF_6$ in DMSO with varying concentrations of substrate **1**.

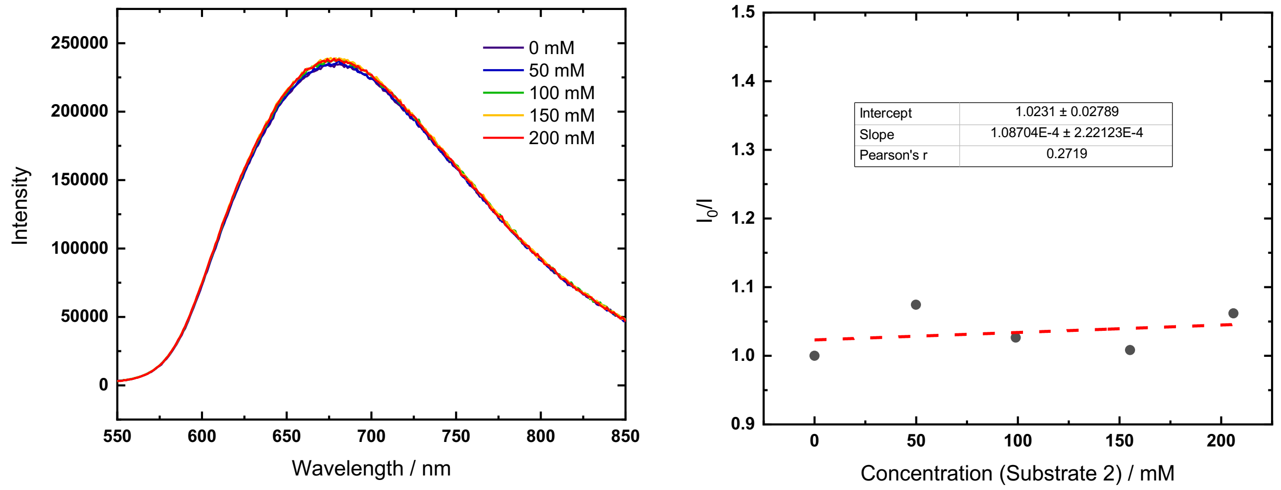

Figure S8. Emission spectra (left) and Stern-Volmer plot (right) of [Fe(phtmeimb)$_2$]PF$_6$ in DMSO with varying concentrations of substrate **2**.

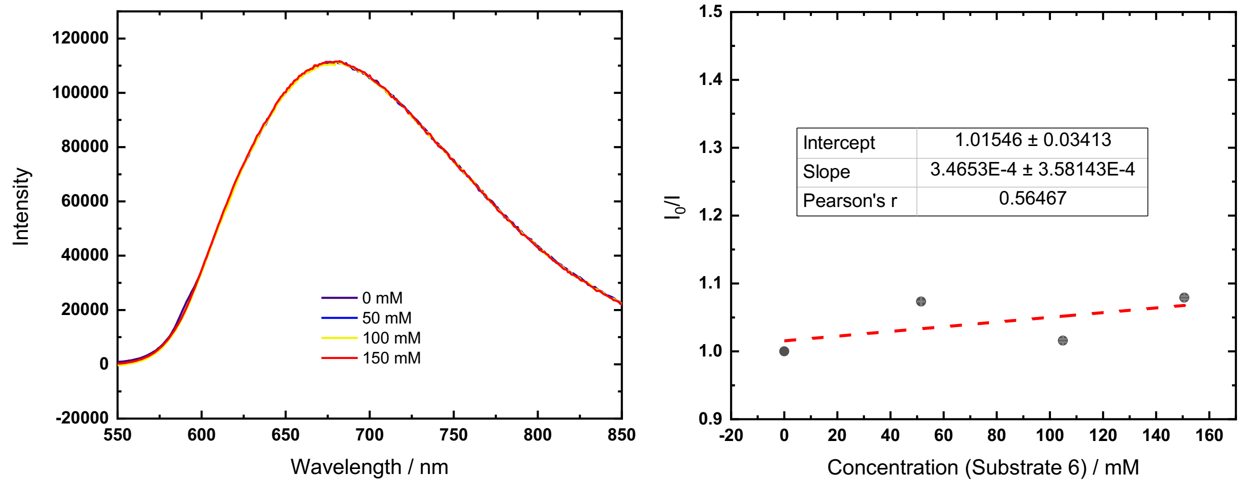

Figure S9. Emission spectra (left) and Stern-Volmer plot (right) of [Fe(phtmeimb)$_2$]PF$_6$ in DMSO with varying concentrations of substrate **6**.

1-Methylpyrrole

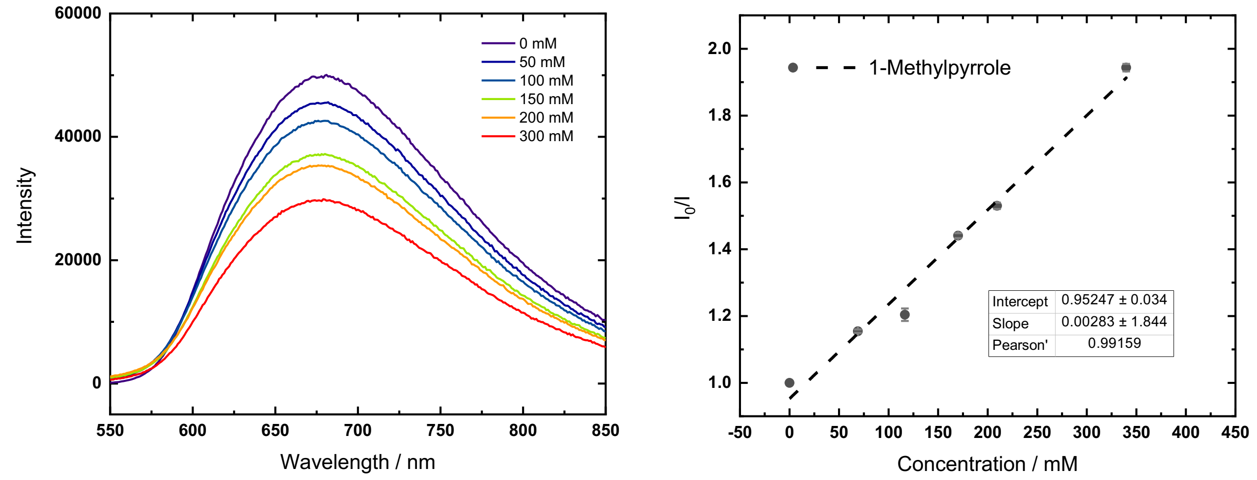

Figure S10. Emission spectra (left) and Stern-Volmer plot (right) of [Fe(phtmeimb)$_2$]PF$_6$ in DMSO with varying concentrations of 1-methylpyrrole.

Benzylpyrrole

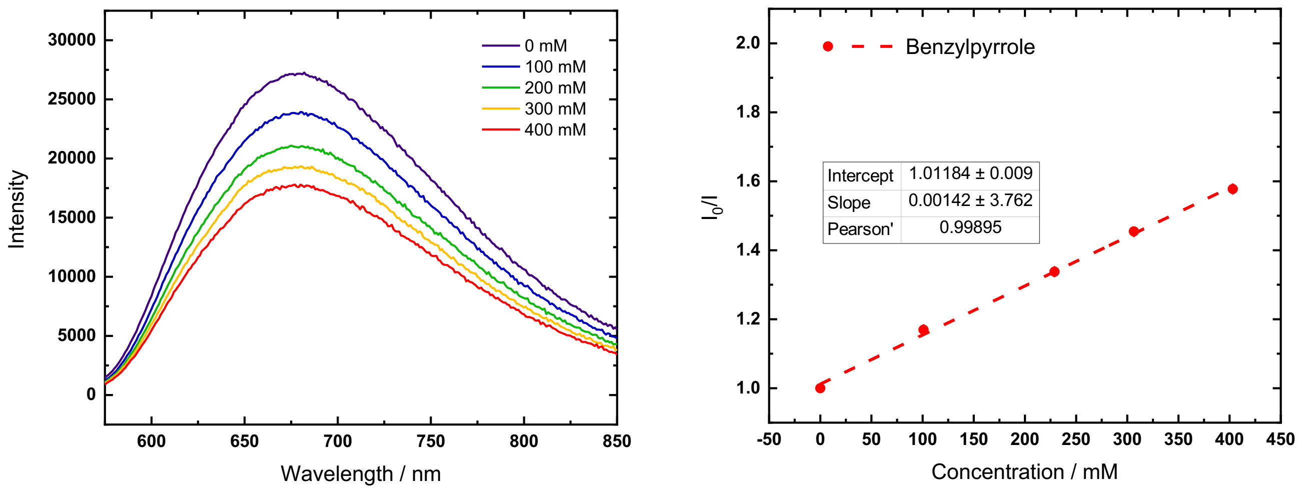

Figure S11. Emission spectra (left) and Stern-Volmer plot (right) of [Fe(phtmeimb)$_2$]PF_6 in DMSO with varying concentrations of benzylpyrrole.

Iodobenzene

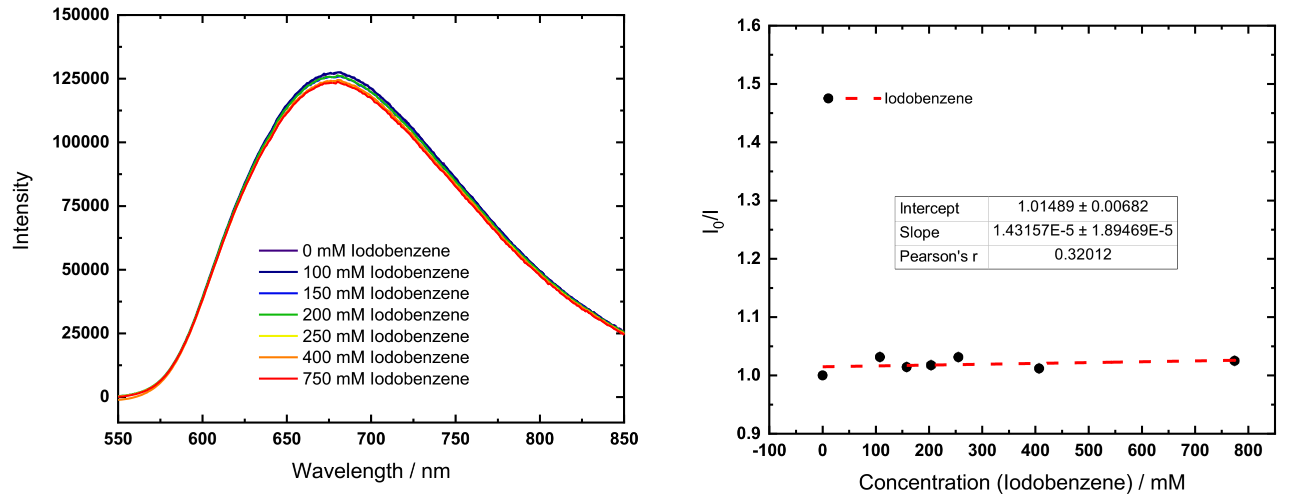

Figure S12. Emission spectra (left) and Stern-Volmer plot (right) of [Fe(phtmeimb)$_2$]PF_6 in DMSO with varying concentrations of iodobenzene.

Bu_3N, Et_3N, TMP

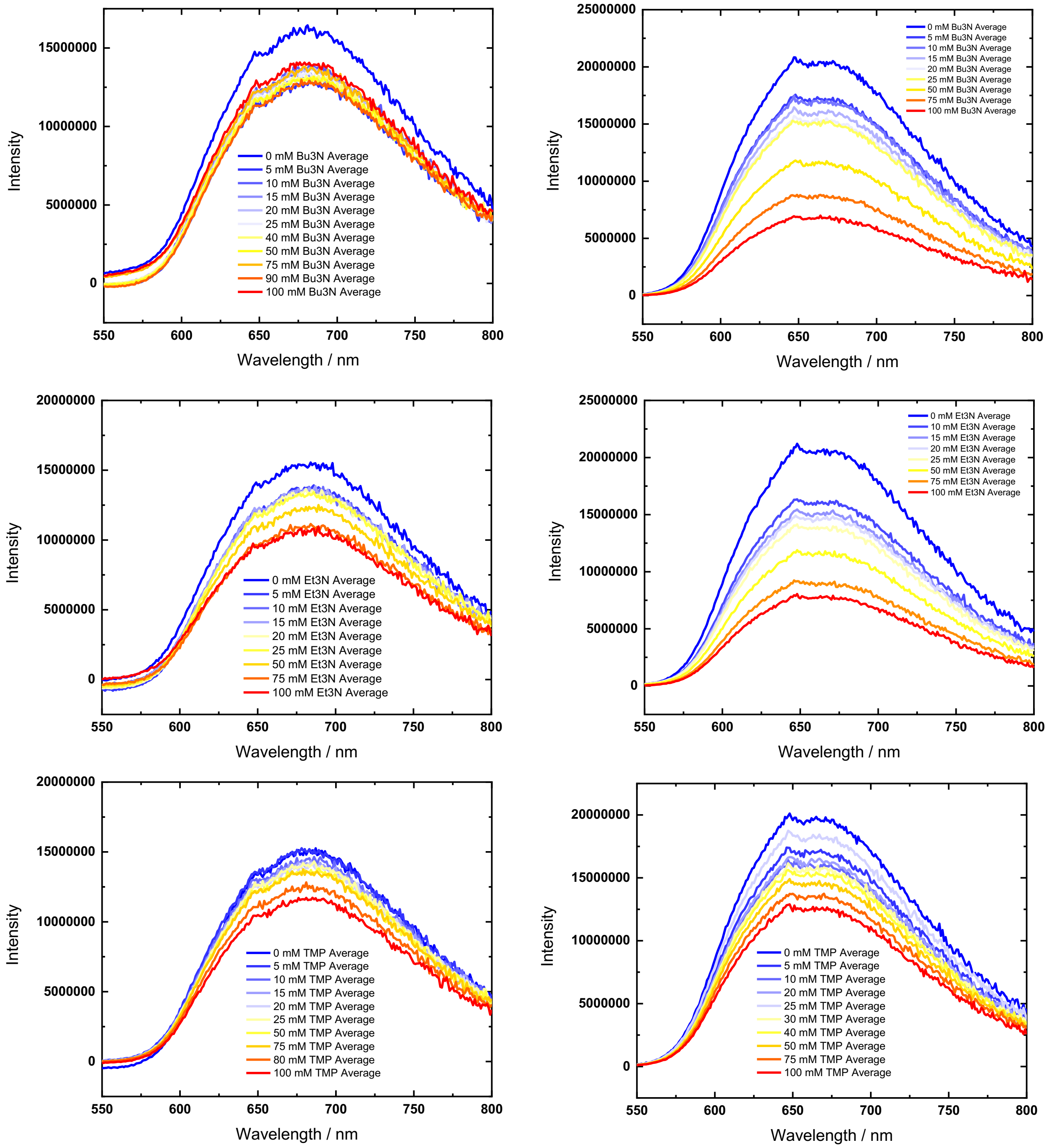

Figure S13. Emission spectra of $[Fe(phtmeimb)_2]PF_6$ in DMSO (left) and acetonitrile (right) with varying concentrations of Bu_3N, Et_3N and TMP.

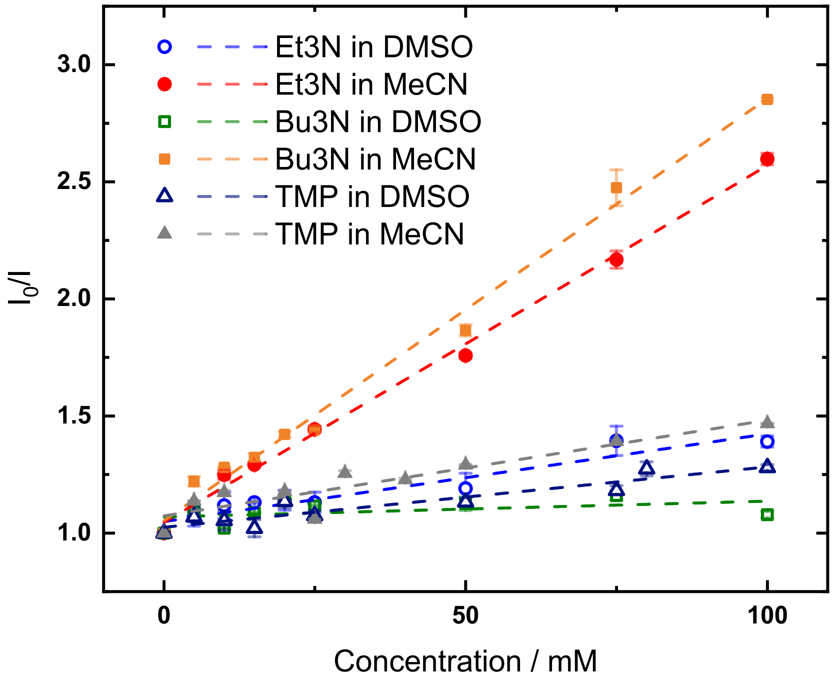

Figure S14. Stern-Volmer plots of $[Fe(phtmeimb)_2]PF_6$ in DMSO and acetonitrile with varying concentrations of Bu_3N, Et_3N and TMP.

Cage escape yield measurements

Solutions of $[Ru(bpy)_3](PF_6)_2$ in DMSO or acetonitrile matching the absorbance of $[Fe(phtmeimb)_2]PF_6$ at the excitation wavelength and excited with the same energy were used as actinometer for the determination of the cage escape yields. Cage escape yields of electron transfer products were calculated using the transient absorption of the Fe(III) → Fe(II) reduction of $[Fe(phtmeimb)_2]^+$ ($\Delta\varepsilon$ = 9863 $M^{-1}cm^{-1}$ at 350 nm, in acetonitrile)[28] and of the ^{3}MLCT excited state of $[Ru(bpy)_3]^{2+}$ ($\Delta\varepsilon$ = -1.1×10^4 $M^{-1}cm^{-1}$ at 452 nm, in acetonitrile).[48] The differences in extinction coefficients for $[Fe(phtmeimb)_2]^+$ and $[Ru(bpy)_3]^{2+}$ in DMSO are not known, hence it is hereby assumed that the differences are similar to those for acetonitrile while determining the cage escape yields for experiments in DMSO.

Table S14. Calculations for cage escape yields of quenching products.

Quencher	A_{Fe} (λ_{ex}) [a]	ΔA_{Fe} (350) [b]	Δ[Fe]/M [c]	A_{Ru} (λ_{ex}) [d]	ΔA_{Ru} (452) [e]	Δ[Ru]/M [f]	f [g]	Φ ([Q]/M) [h]	η_q [i]	η_{ce} [j]
DMSO										
Substrate 1	0.46	0.0013	1.32×10^{-7}	0.47	-0.221	2.01×10^{-5}	1.01	0.007 (0.387)	0.23	0.03
1-Methylpyrrole	0.42	0.0017	1.72×10^{-7}	0.42	-0.202	1.84×10^{-5}	1.00	0.009 (0.301)	0.45	0.02
Benzylpyrrole	0.45	0.0016	1.62×10^{-7}	0.46	-0.286	2.60×10^{-5}	1.01	0.006 (0.410)	0.37	0.02
Bu_3N	0.52	<0.001 [k]	0.36×10^{-7}	0.47	-0.221	2.01×10^{-5}	0.95	0.002 (0.101)	0.12	<0.04 [k]
Et_3N	0.52	0.0014	1.46×10^{-7}					0.007 (0.100)	0.30	0.02
TMP	0.52	0.0014	1.42×10^{-7}					0.007 (0.302)	0.44	0.02
Acetonitrile										
Bu_3N	0.53	0.0014	1.42×10^{-7}	0.52	-0.173	1.57×10^{-5}	0.99	0.009 (0.056)	0.51	0.02
Et_3N	0.51	0.0012	1.26×10^{-7}				1.01	0.008 (0.057)	0.48	0.02
TMP	0.52	0.0010	1.01×10^{-7}				1.00	0.006 (0.150)	0.41	0.02

[a] Sample absorbance at the excitation wavelength (465 nm)
[b] Photo-induced absorbance change of the sample at 350 nm
[c] Photo generated concentration of Fe(III/II) based on $\Delta\varepsilon$ = 9863 $M^{-1}cm^{-1}$ at 350 nm in acetonitrile
[d] Actinometer absorbance at the excitation wavelength
[e] Photo-induced absorbance change of the actinometer at 452 nm
[f] Photo generated concentration of $^*[Ru(bpy)_3]^{2+}$ based on $\Delta\varepsilon$ = -1.1×10^4 $M^{-1}cm^{-1}$ at 452 nm
[g] Correction factor for absorbance difference between sample and actinometer

$$f = \left(1-10^{-A_{Ru}(\lambda_{ex})}\right)/\left(1-10^{-A_{Fe}(\lambda_{ex})}\right)$$

[h] Quantum yield of electron transfer products $\phi = (\Delta[Fe]/\Delta[Ru])f$
[i] Quenching yield from steady state emission quenching
[j] Cage escape yield $\eta_{ce} = \phi/\eta_q$
[k] No obvious observable signal within detector limits hence assume any available signal was less than the lowest observable signal (0.0010 mOD)

1-Methylpyrrole

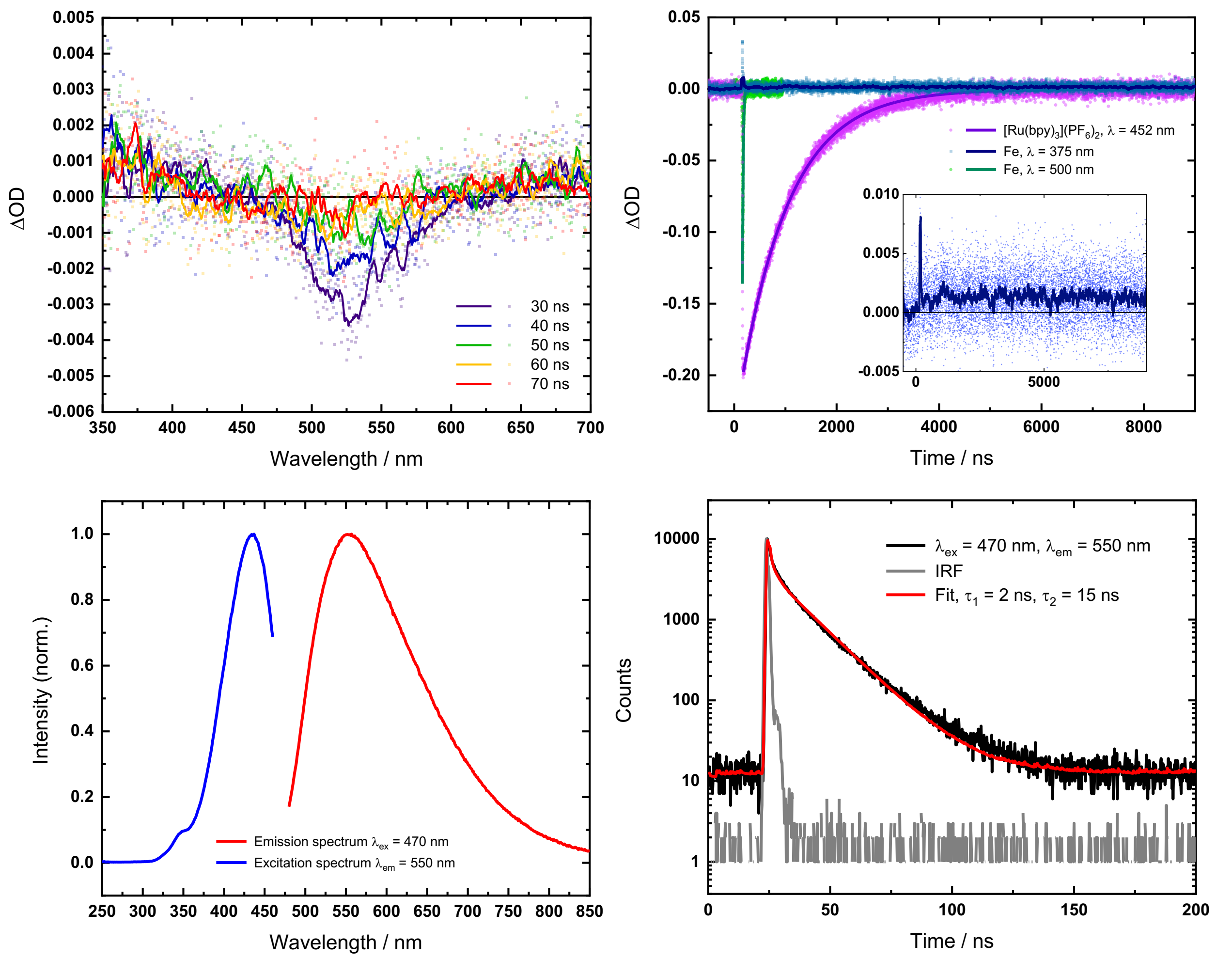

Figure S15. Top left: Transient absorption spectra at indicated time delays of quenching of $[Fe(phtmeimb)_2]PF_6$ with 1-methylpyrrole in DMSO. Top right: kinetic decay curves at indicated wavelengths, compared with ground state bleach recovery signal of actinometer $[Ru(bpy)_3](PF_6)_2$ (inset: zoomed-in graph of decay at 375 nm). Bottom left: emission and excitation spectra of 1-methylpyrrole. Bottom right: emission lifetime of 1-methylpyrrole taken by TCSPC.

Spectra obtained from ns-TAS showed a long-lived positive signal peaking at 375 nm, and a short-lived negative signal peaking at around 525 nm. The long-lived component at 375 nm can be attributed to the formation of ground-state Fe(II), while the shorter-lived one could be the stimulated emission from 1-methylpyrrole. The kinetic decay at 500 nm is pulse-limited due to the short lifetime of 1-methylpyrrole.

Benzylpyrrole

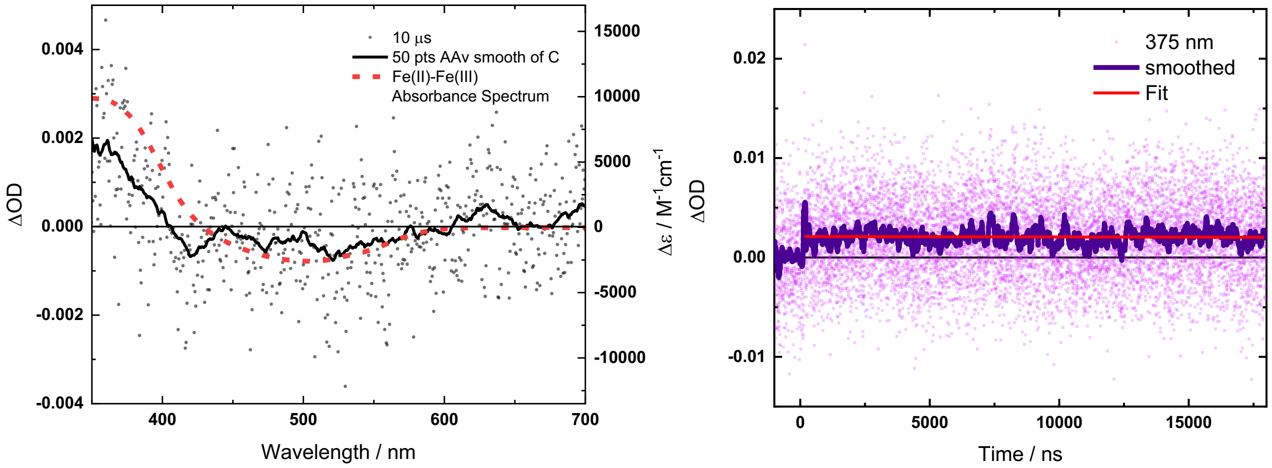

Figure S16. Transient absorption spectra at indicated time delay of quenching of $[Fe(phtmeimb)_2]PF_6$ with benzylpyrrole in DMSO (left); kinetic trace at indicated wavelength (right).

Bu_3N

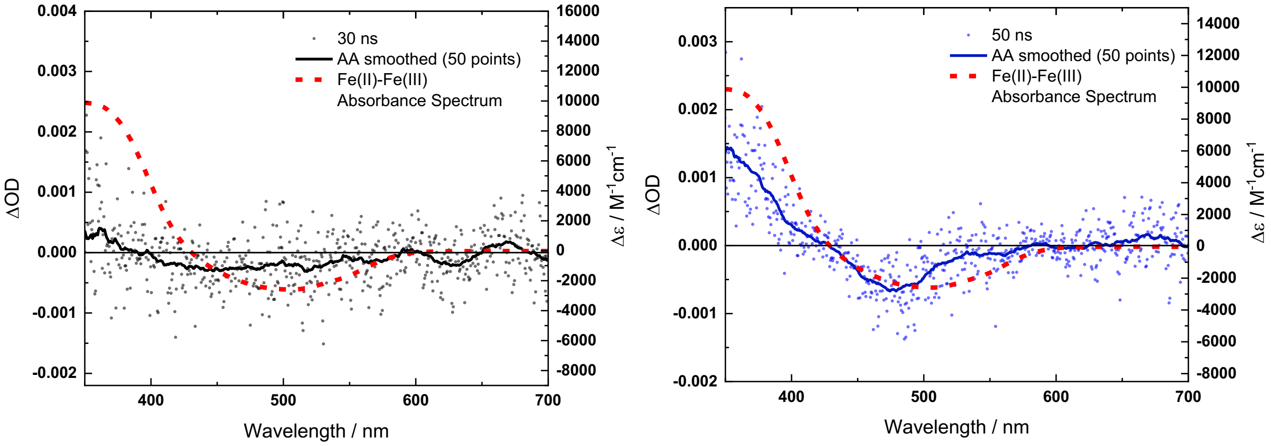

Figure S17. Transient absorption spectra at indicated time delays of quenching of $[Fe(phtmeimb)_2]PF_6$ with Bu_3N in DMSO (left) and acetonitrile (right), along with differential absorption spectrum for the metal centered reduction of $[Fe(phtmeimb)_2]PF_6$.

Et_3N

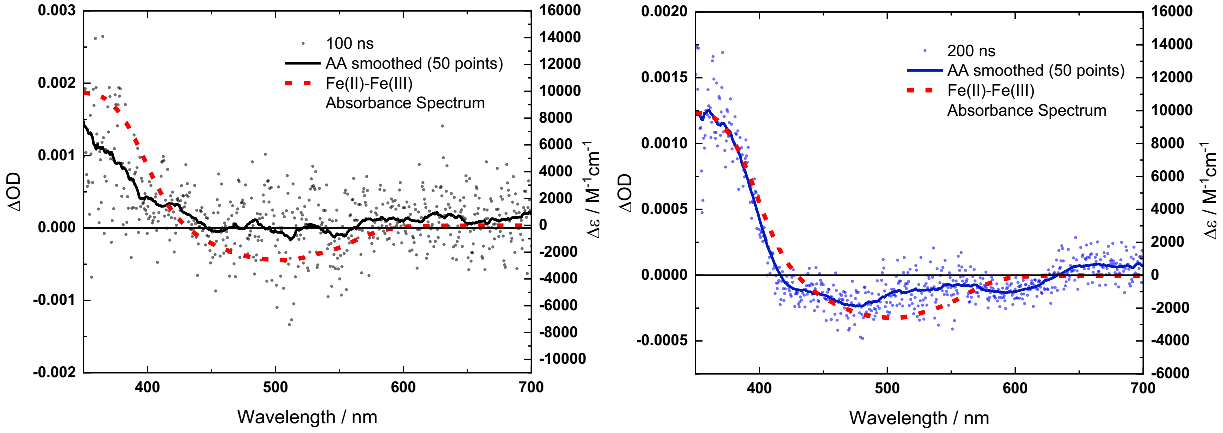

Figure S18. Transient absorption spectra at indicated time delays of quenching of $[Fe(phtmeimb)_2]PF_6$ with Et_3N in DMSO (left) and acetonitrile (right), along with differential absorption spectrum for the metal centered reduction of $[Fe(phtmeimb)_2]PF_6$.

TMP

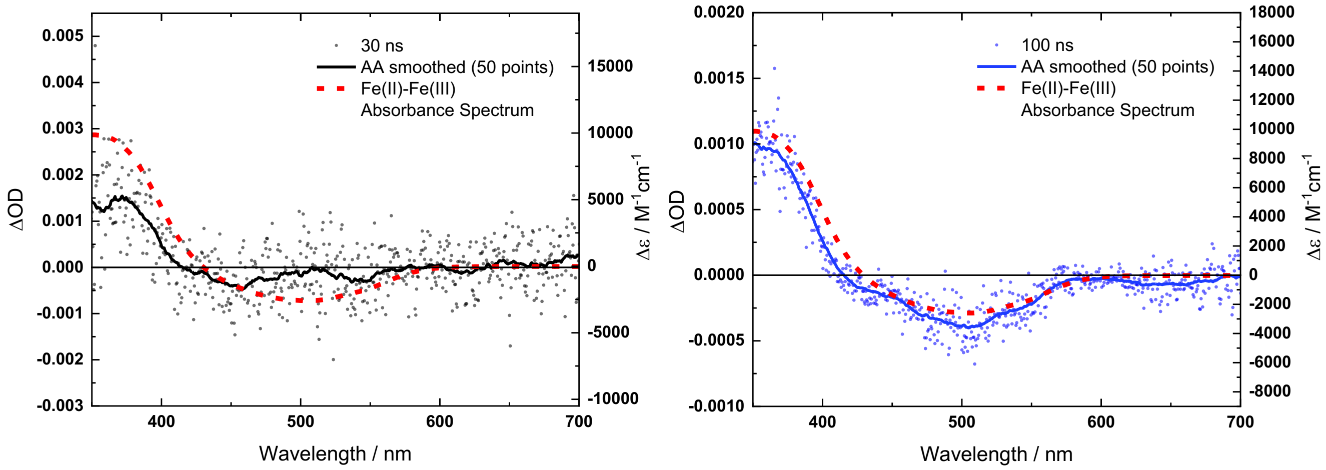

Figure S19. Transient absorption spectra at indicated time delays of quenching of $[Fe(phtmeimb)_2]PF_6$ with TMP in DMSO (left) and acetonitrile (right), along with differential absorption spectrum for the metal centered reduction of $[Fe(phtmeimb)_2]PF_6$.

Substrate 1

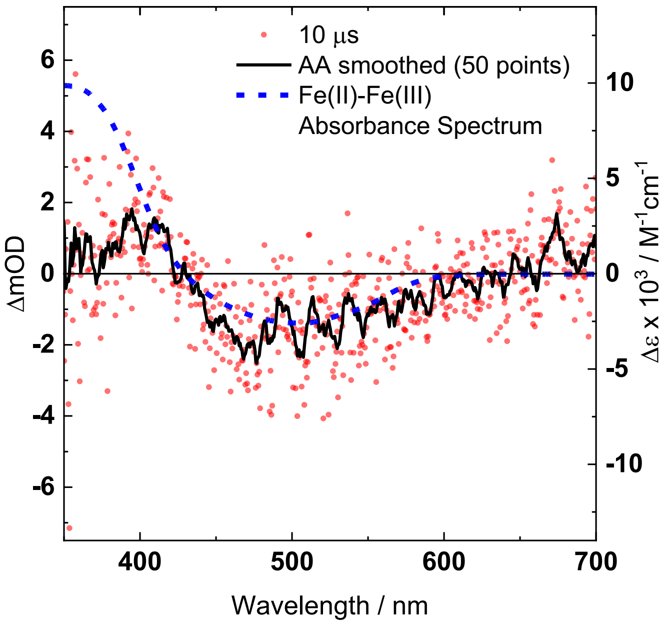

Figure S20. Transient absorption spectra at indicated time delays of quenching of $[Fe(phtmeimb)_2]PF_6$ with Substrate **1** in DMSO, along with differential absorption spectrum for the metal centered reduction of $[Fe(phtmeimb)_2]PF_6$.

Aldehyde side-product

Traces of aldehyde side-products were observed in the BHAS reactions of substrates containing electron-donating substituents in the *para* position relative to the bridging oxygen (**2**, **3**, **4** and **11**), or in absence of substituent (**5**). A proposed mechanism is shown in Figure S21. The pathway is initiated by the formation of the reactive intermediate **E**. This initiation step is the same as has been shown in Scheme 1 of the manuscript. However, the formation of the aldehyde side-product is proposed to proceed following a 5-*exo*-trig cyclization, instead of the traditionally observed 6-*endo*-trig cyclisation, which is hypothesized to instead lead to the formation of **CP** (Scheme 1). An earlier report on BHAS reactions has investigated the 5-*exo* and 6-*endo* cyclization pathways by molecular modelling calculations.[13] It was theoretically determined that the transition state for the 5-*exo* cyclization is higher in energy than in case of the 6-*endo* cyclization.[13] However, the 5-*exo* pathway leads to a radical intermediate which is energetically more favorable than the intermediate formed after the alternative 6-*endo* ring-closure.[13] The formation of **AP** is proposed to continue from the 5-*exo* cyclized radical intermediate by a ring expansion and ring opening step (Figure S21). Subsequently, a deprotonation results in the generation of a ketyl radical anion. This intermediate is stabilized by resonance in which the adjacent aromatic system can participate. In the final step of the proposed mechanism, the ketyl radical anion is oxidized ($E_{1/2}$(benzaldehyde/benzaldehyde$^{\bullet-}$) = –2.31 V vs $Fc^{+/0}$, reported as –1.93 V vs SCE)[49, 50] forming the experimentally observed **AP**.

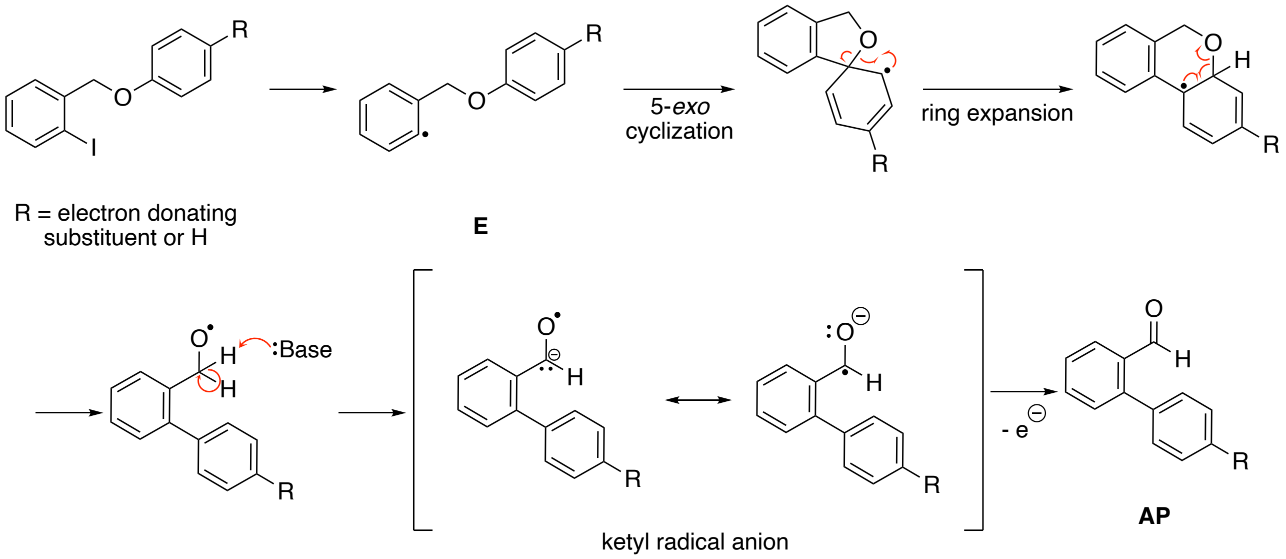

Figure S21. Proposed photoinduced formation pathway of **AP**.

NMR Spectra

5H-Pyrrolo[2,1-a]isoindole (CP 1)

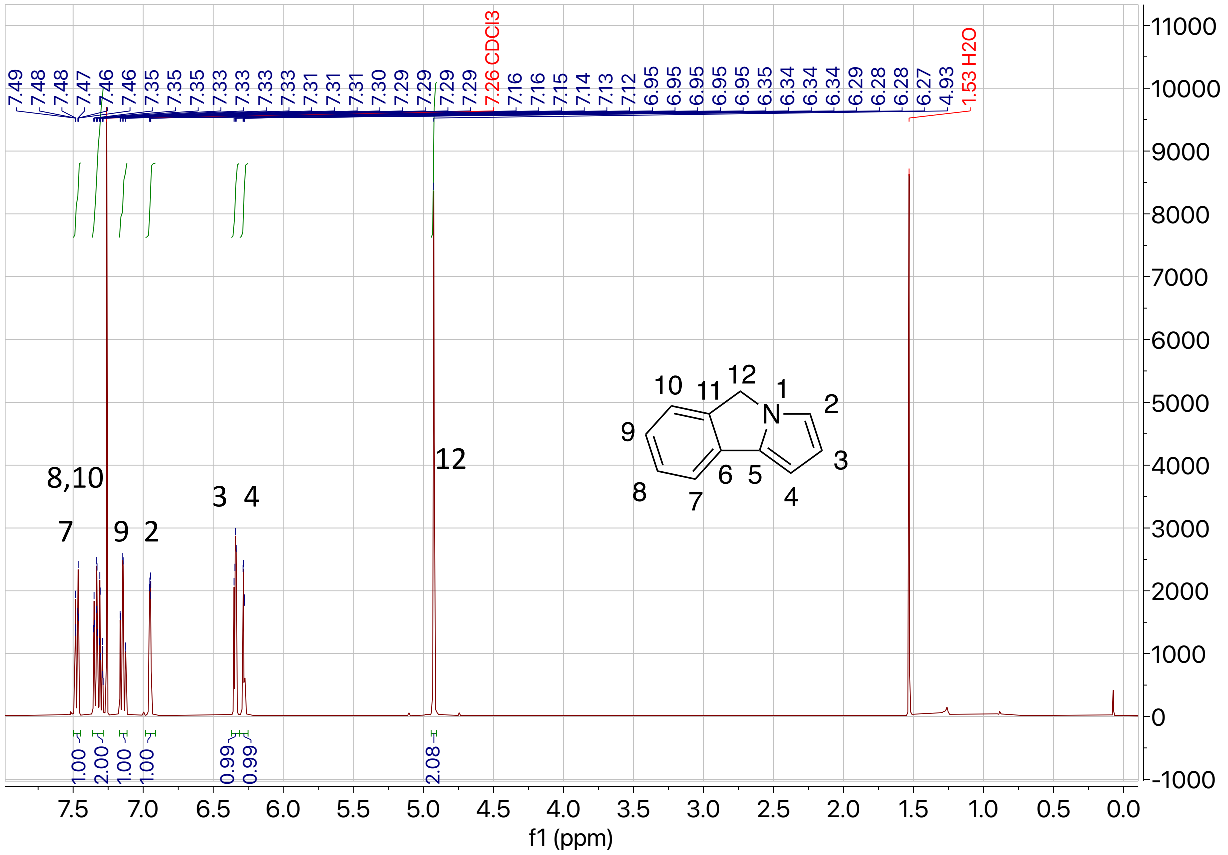

Figure S22: ^{1}H NMR spectrum (400 MHz) of **CP 1** in $CDCl_3$.

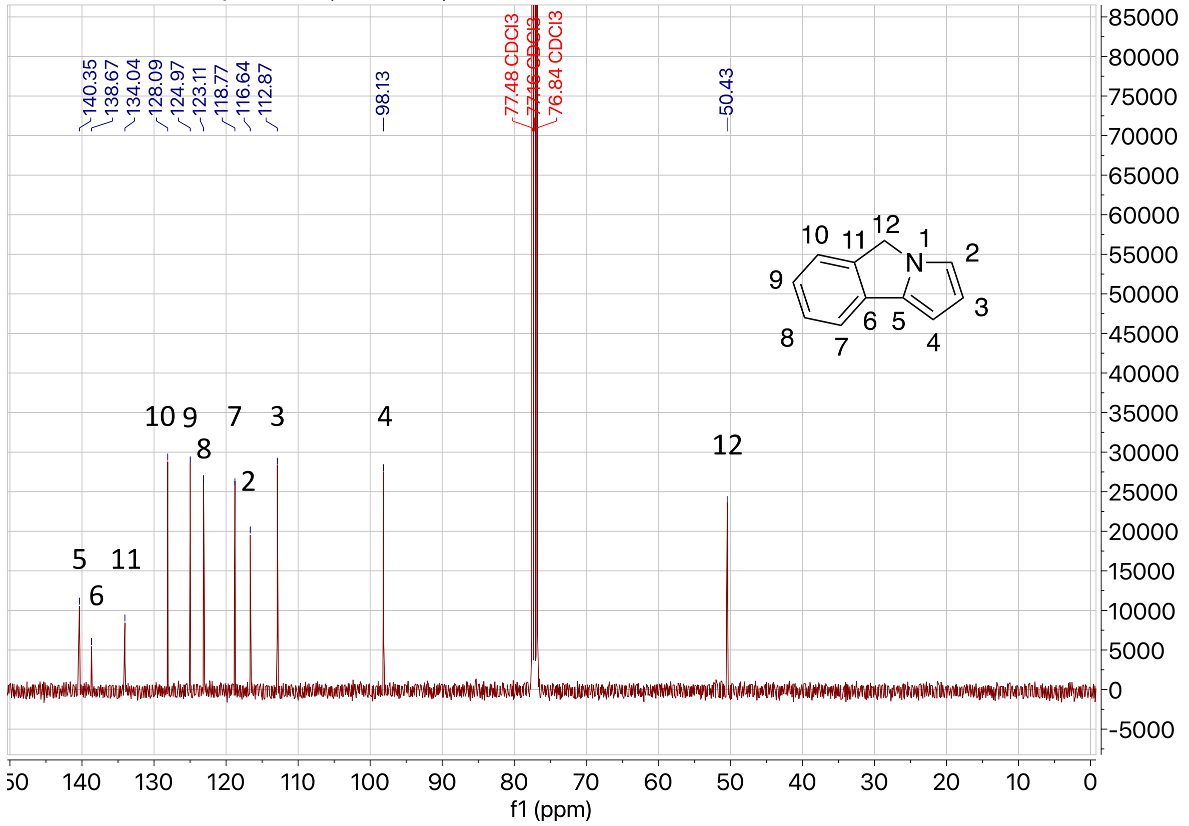

Figure S23: ^{13}C NMR spectrum (101 MHz) of **CP 1** in $CDCl_3$.

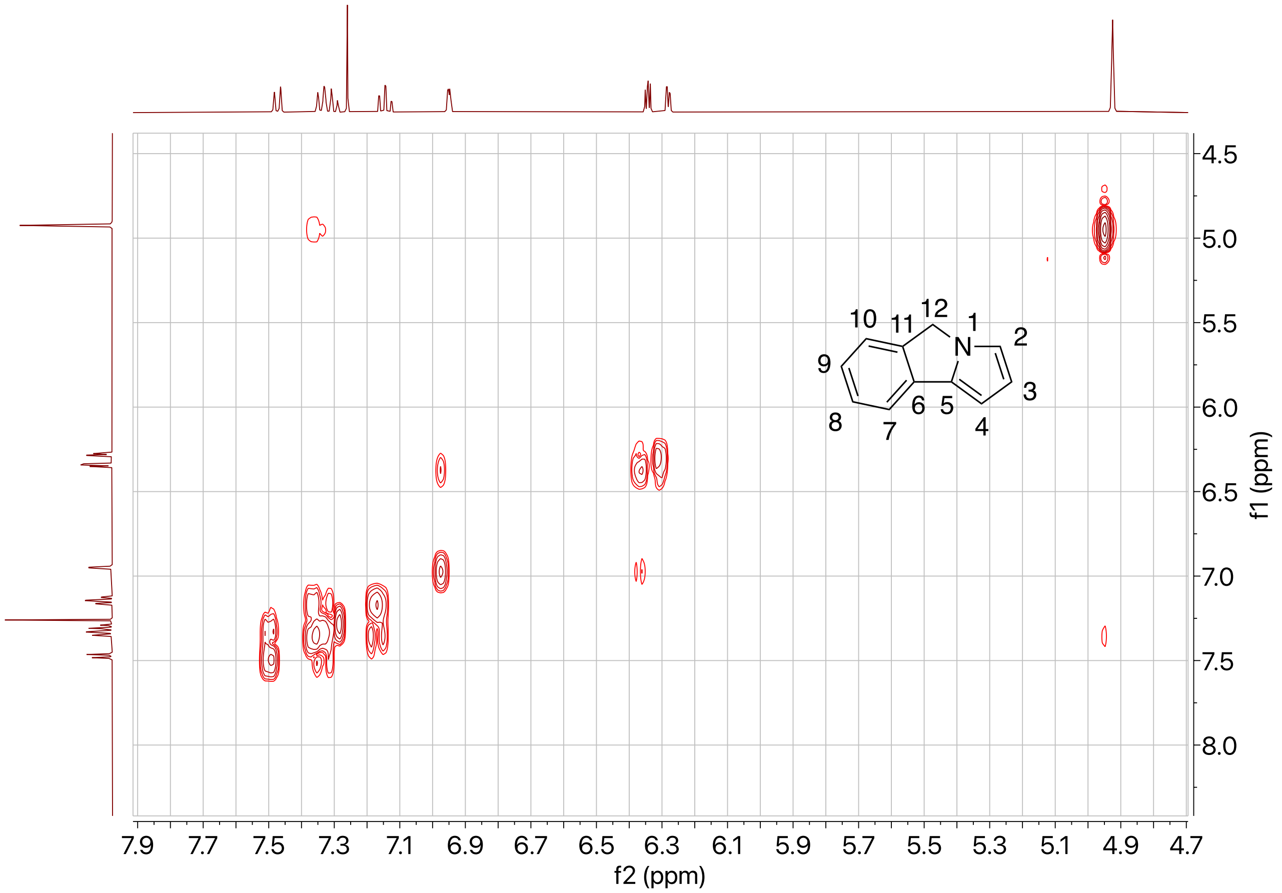

Figure S24: COSY spectrum (400 MHz) of **CP 1** in $CDCl_3$.

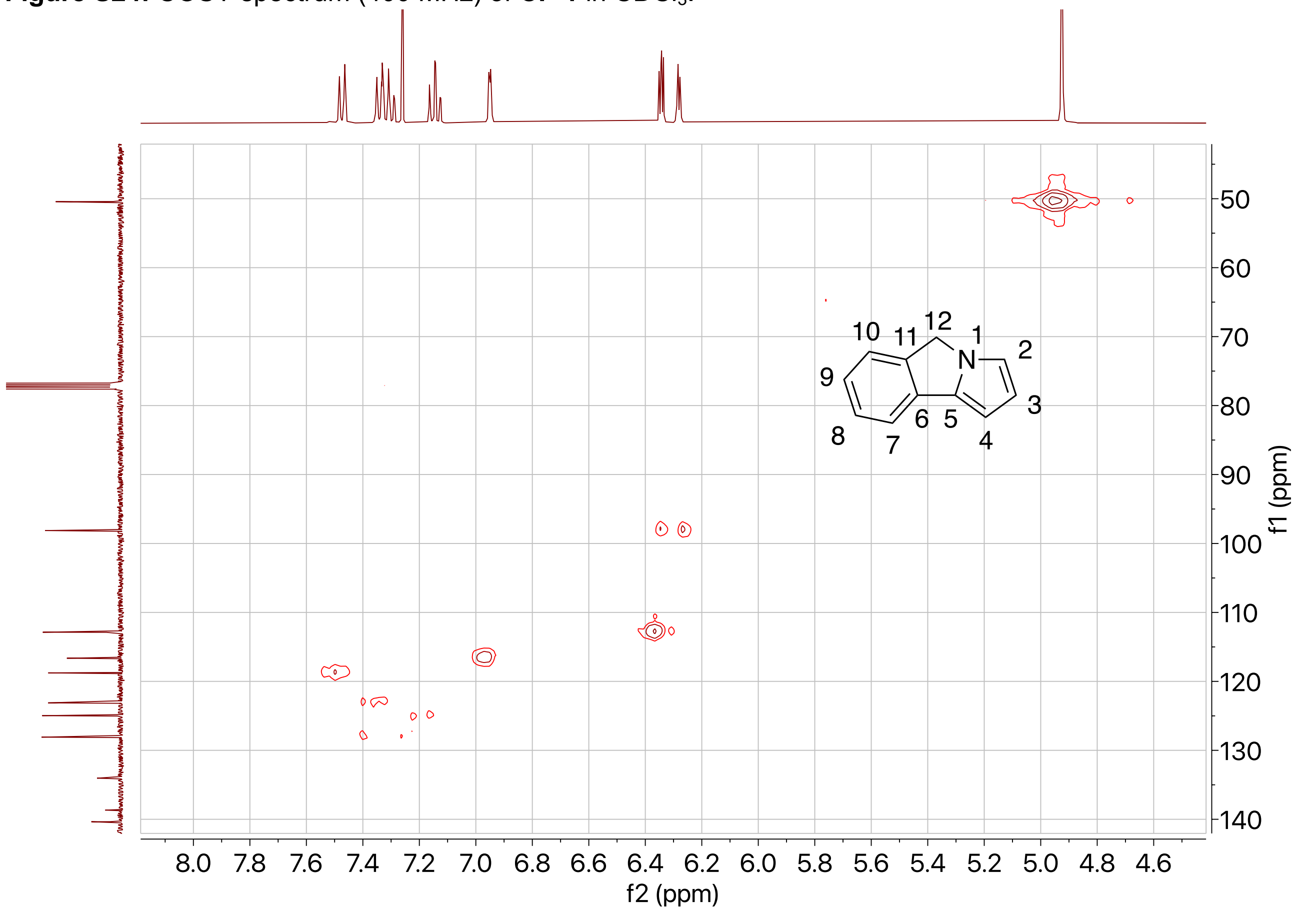

Figure S25: HMQC spectrum of **CP 1** in $CDCl_3$.

1-Benzyl-1H-pyrrole (HP 1)

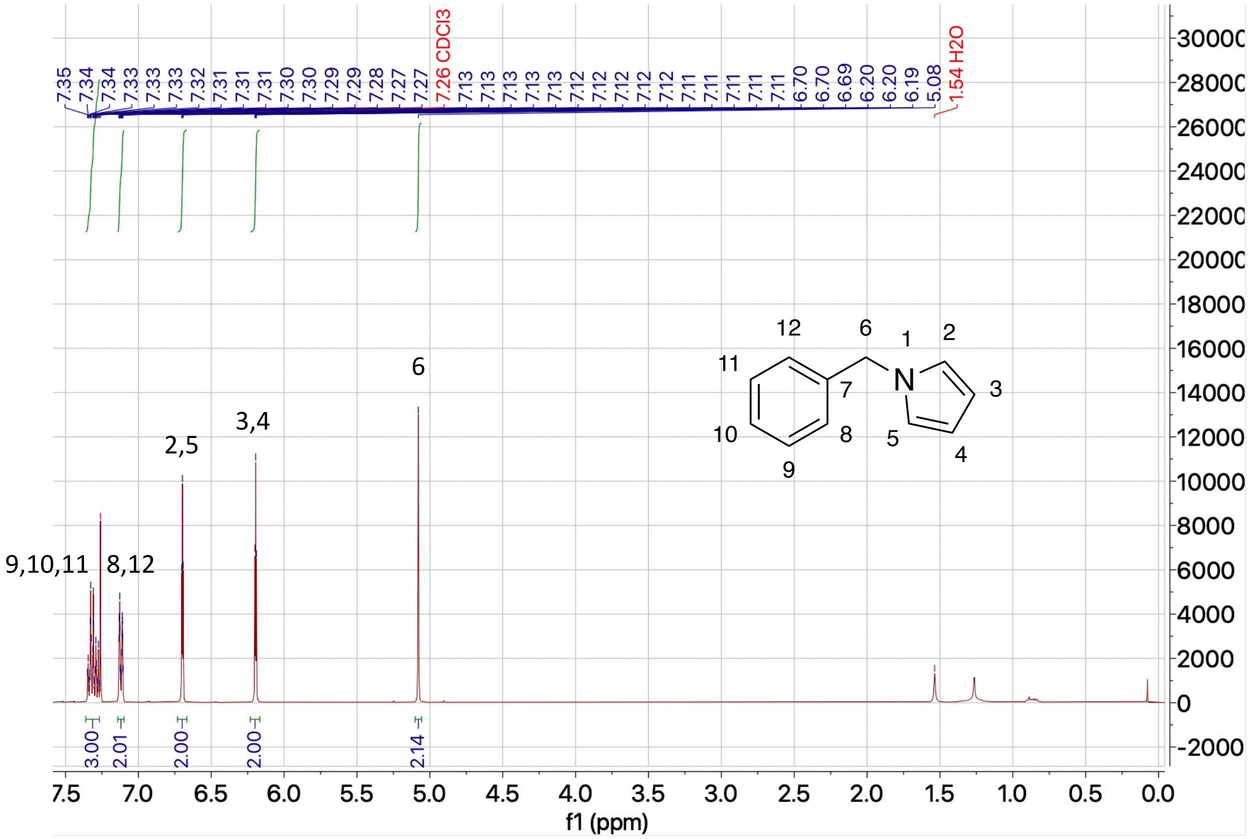

Figure S26: ^{1}H NMR spectrum (400 MHz) of **HP 1** in $CDCl_3$.

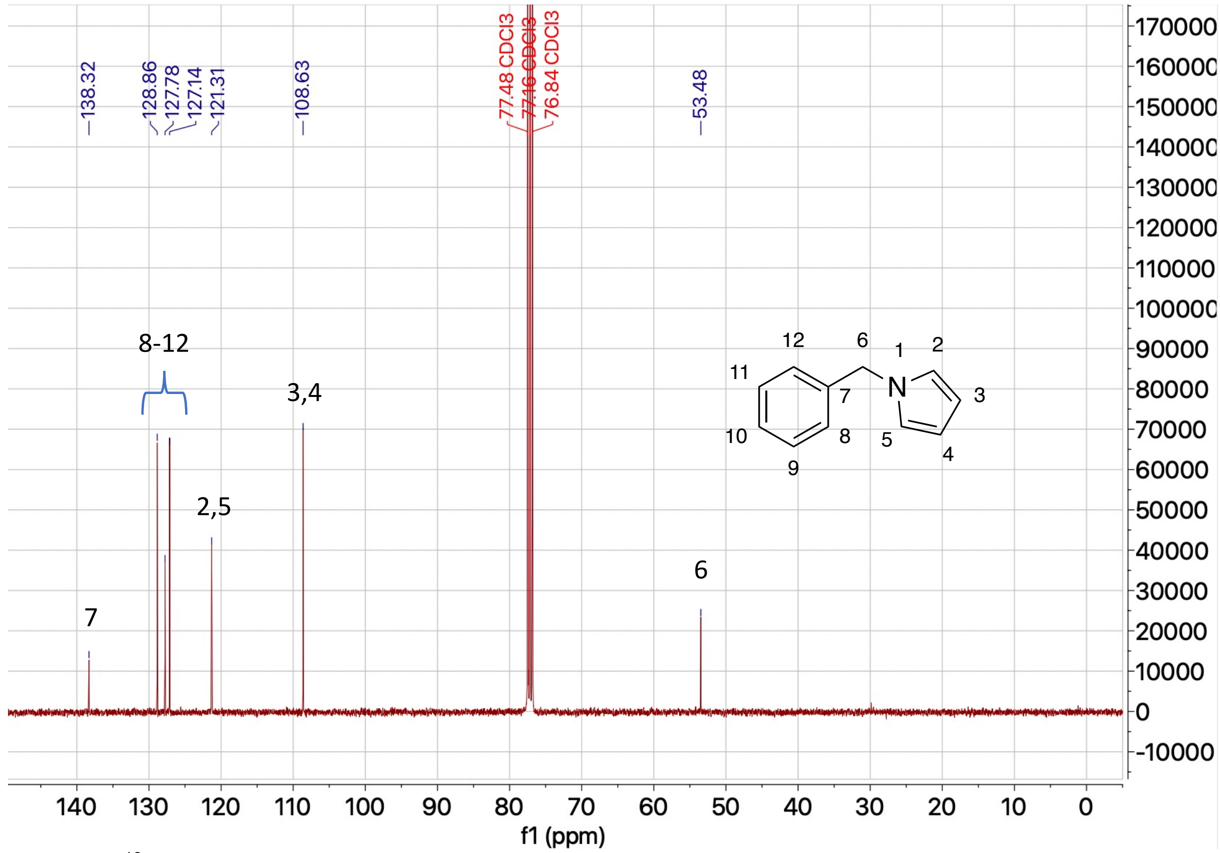

Figure S27: ^{13}C NMR spectrum (101 MHz) of **HP 1** in $CDCl_3$.

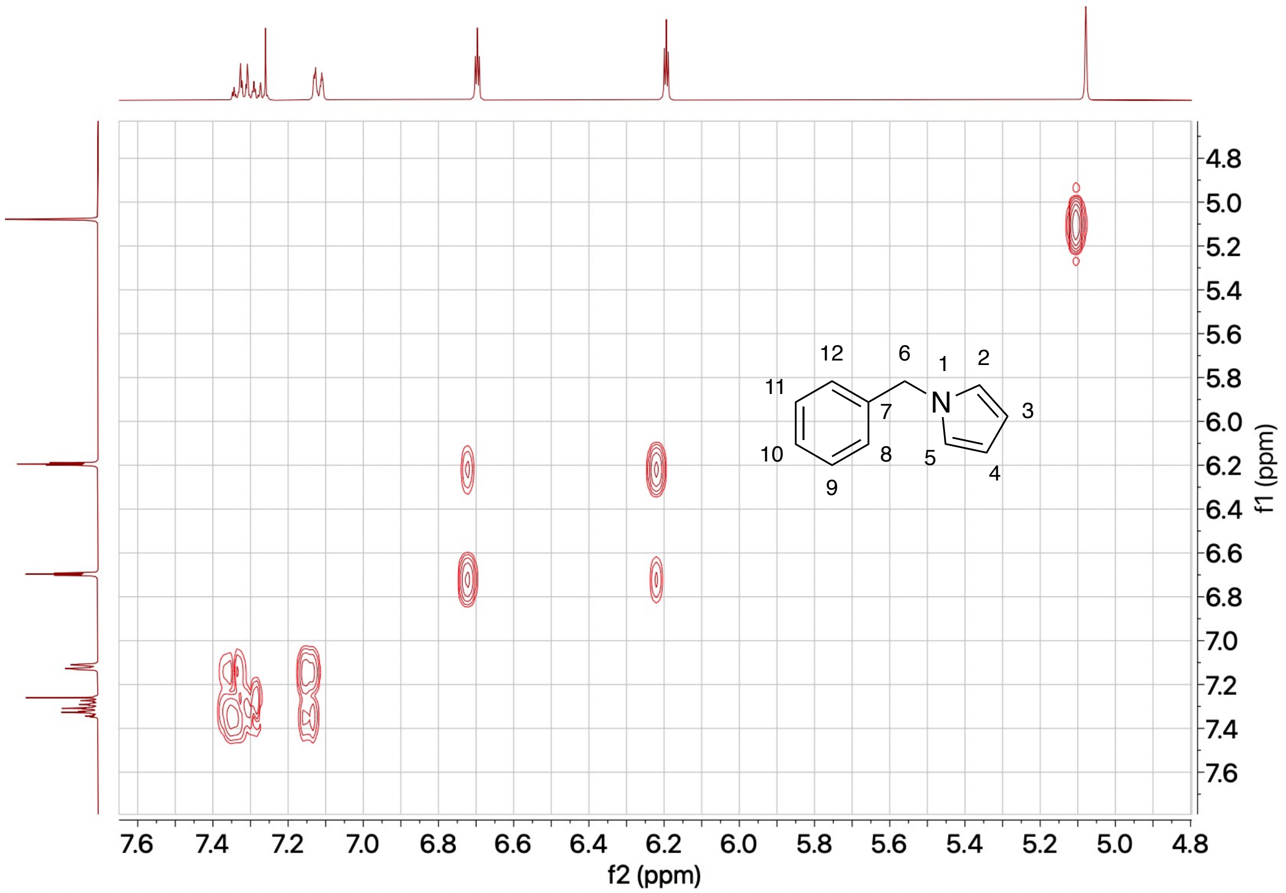

Figure S28: COSY spectrum (400 MHz) of **HP 1** in $CDCl_3$.

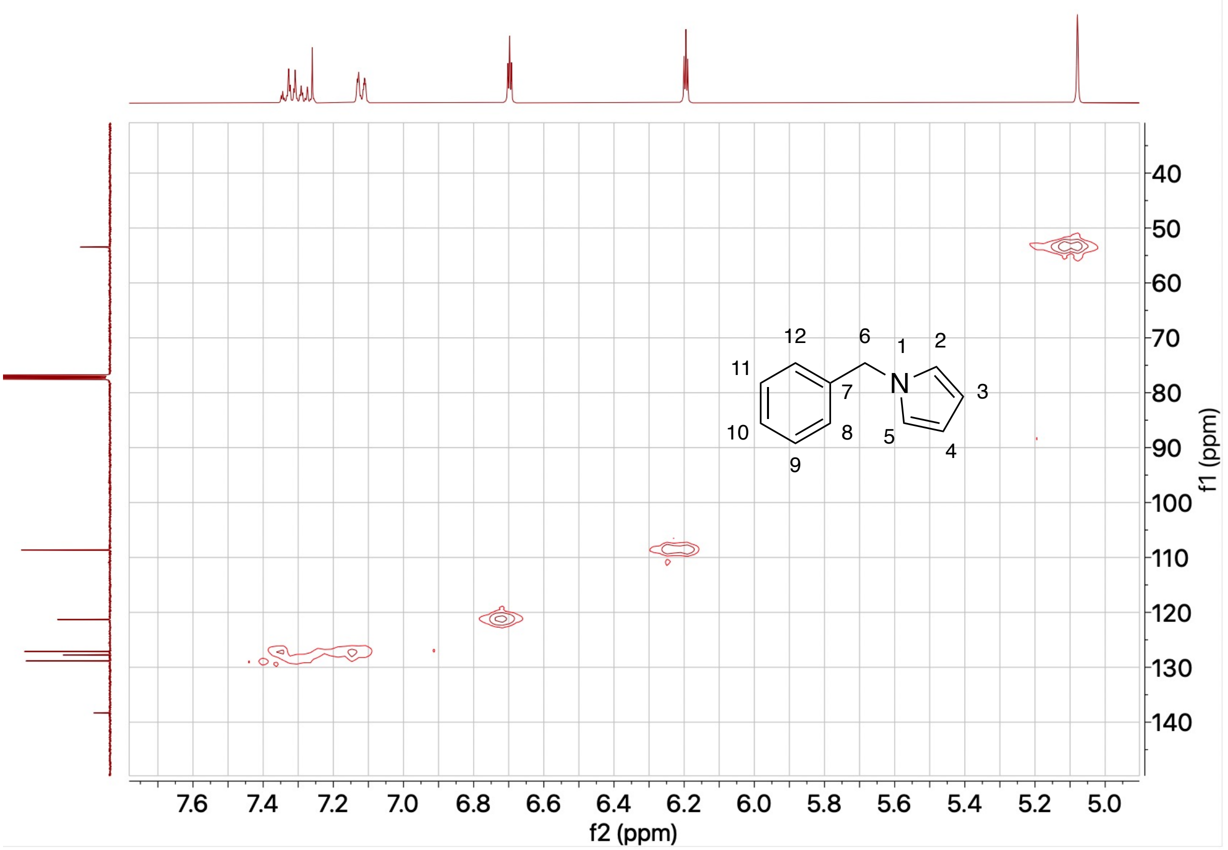

Figure S29: HMQC spectrum of **HP 1** in $CDCl_3$.

2-(tert-Butyl)-6H-benzo[c]chromene (CP 2)

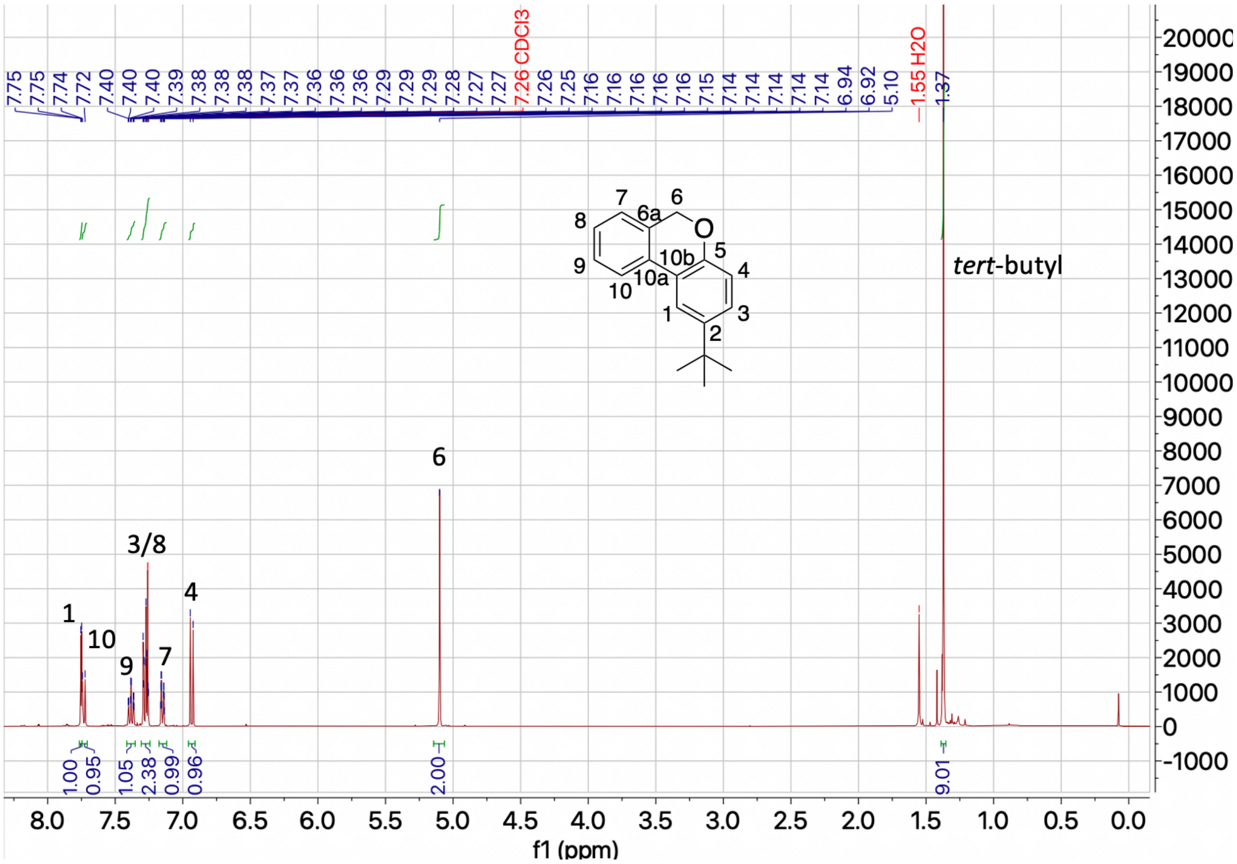

Figure S30: ^1H NMR spectrum (400 MHz) of **CP 2** in $CDCl_3$.

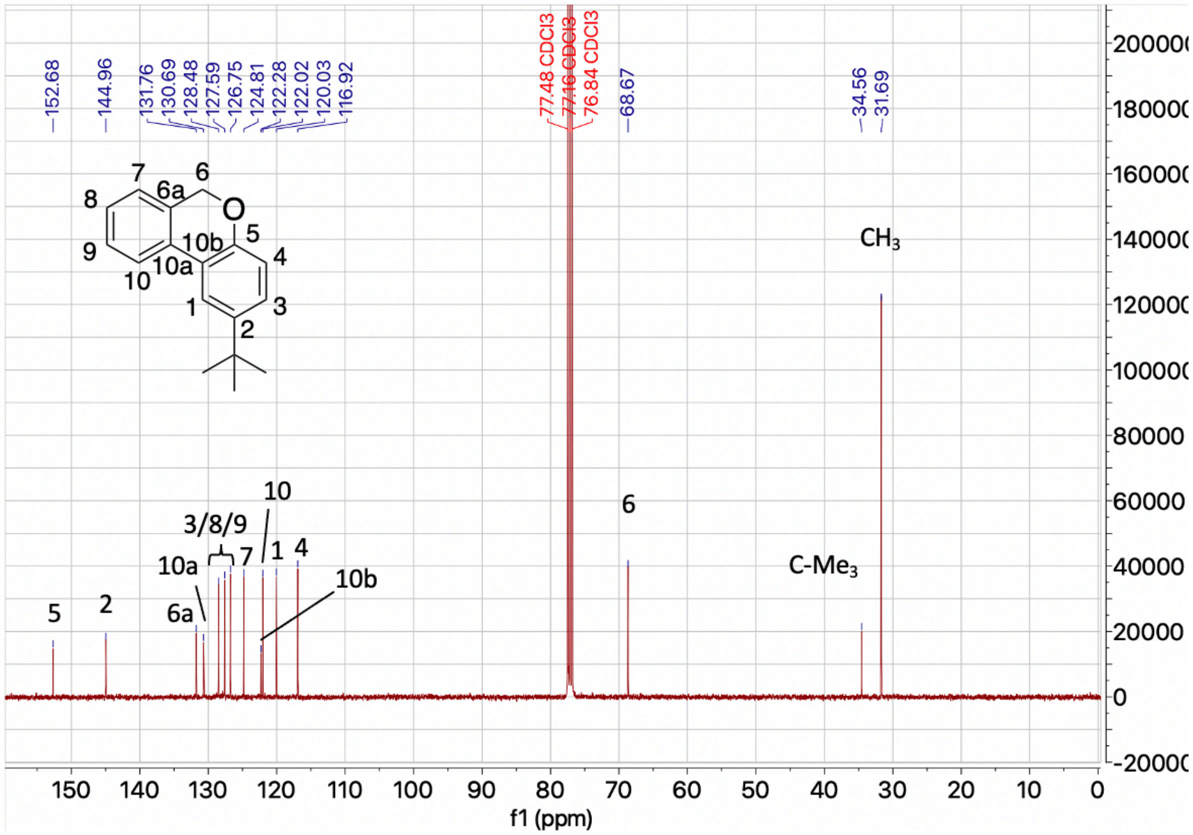

Figure S31: ^{13}C NMR spectrum (101 MHz) of **CP 2** in $CDCl_3$.

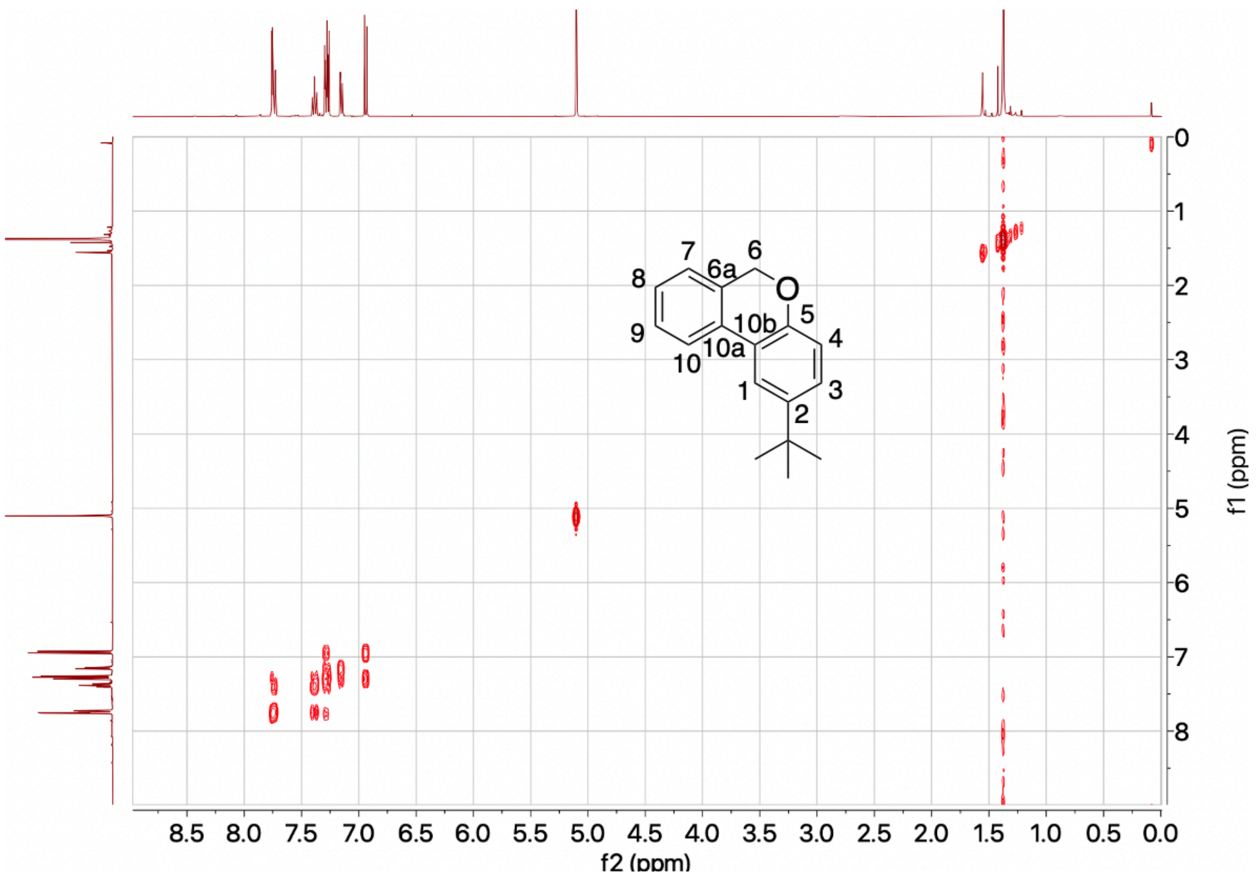

Figure S32: COSY spectrum (400 MHz) of **CP 2** in $CDCl_3$.

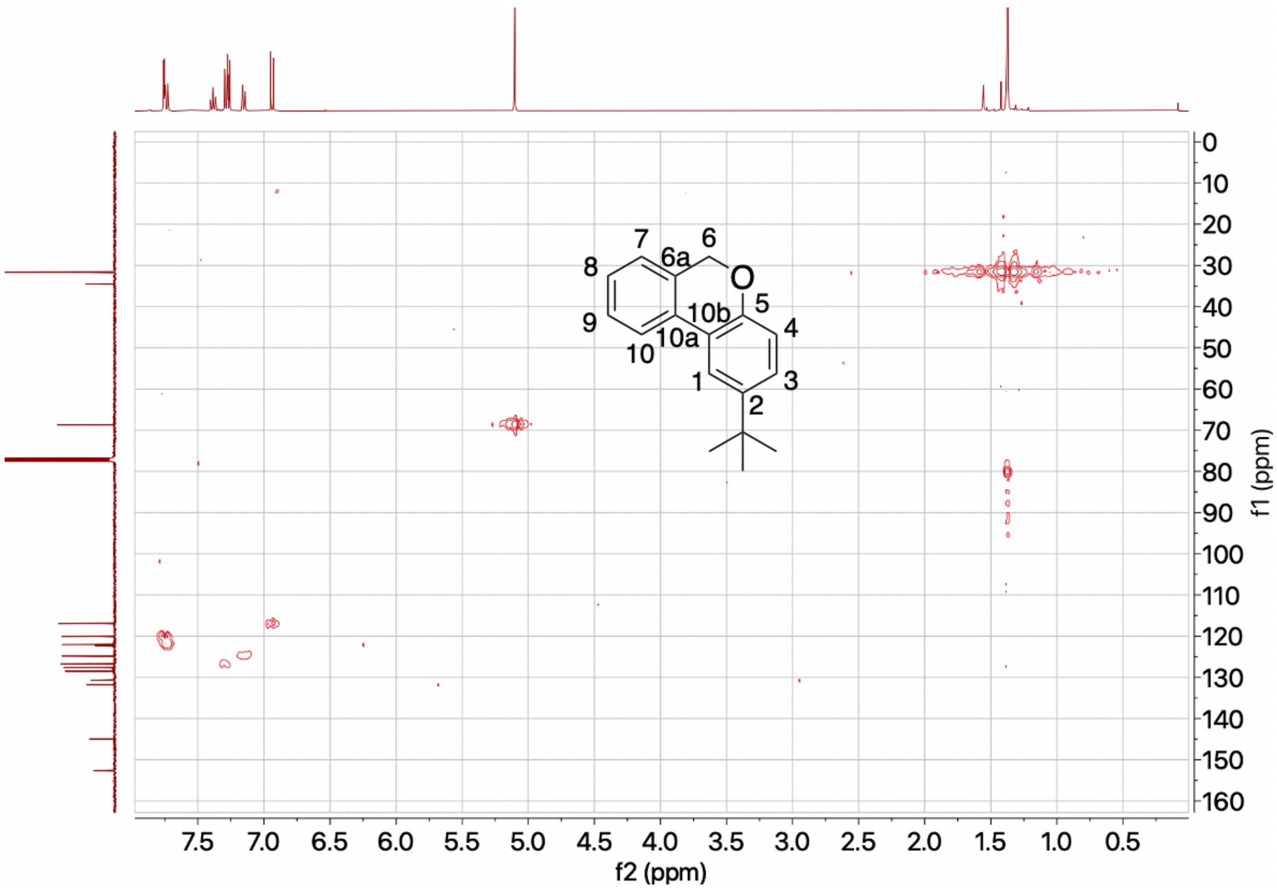

Figure S33: HMQC spectrum of **CP 2** in $CDCl_3$.

1-(Benzyloxy)-4-(tert-butyl)benzene (HP 2)

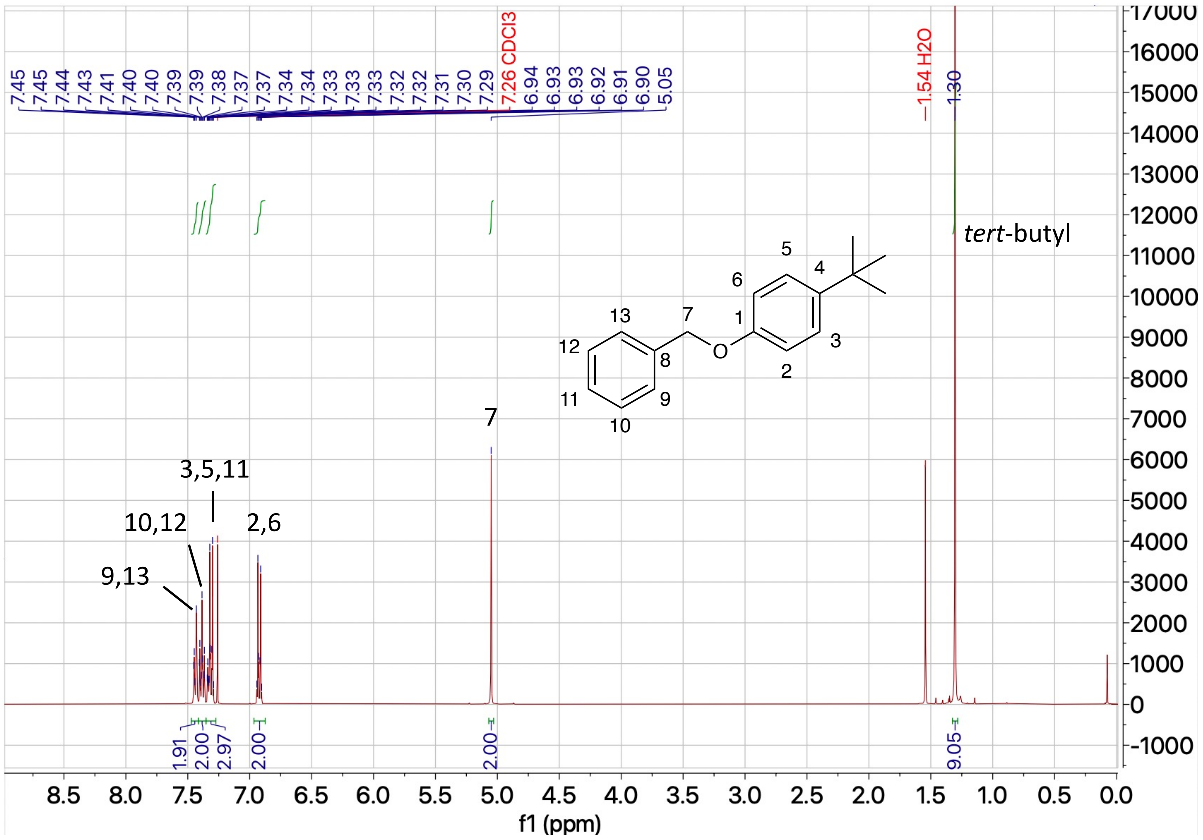

Figure S34: ^{1}H NMR spectrum (400 MHz) of **HP 2** in $CDCl_3$.

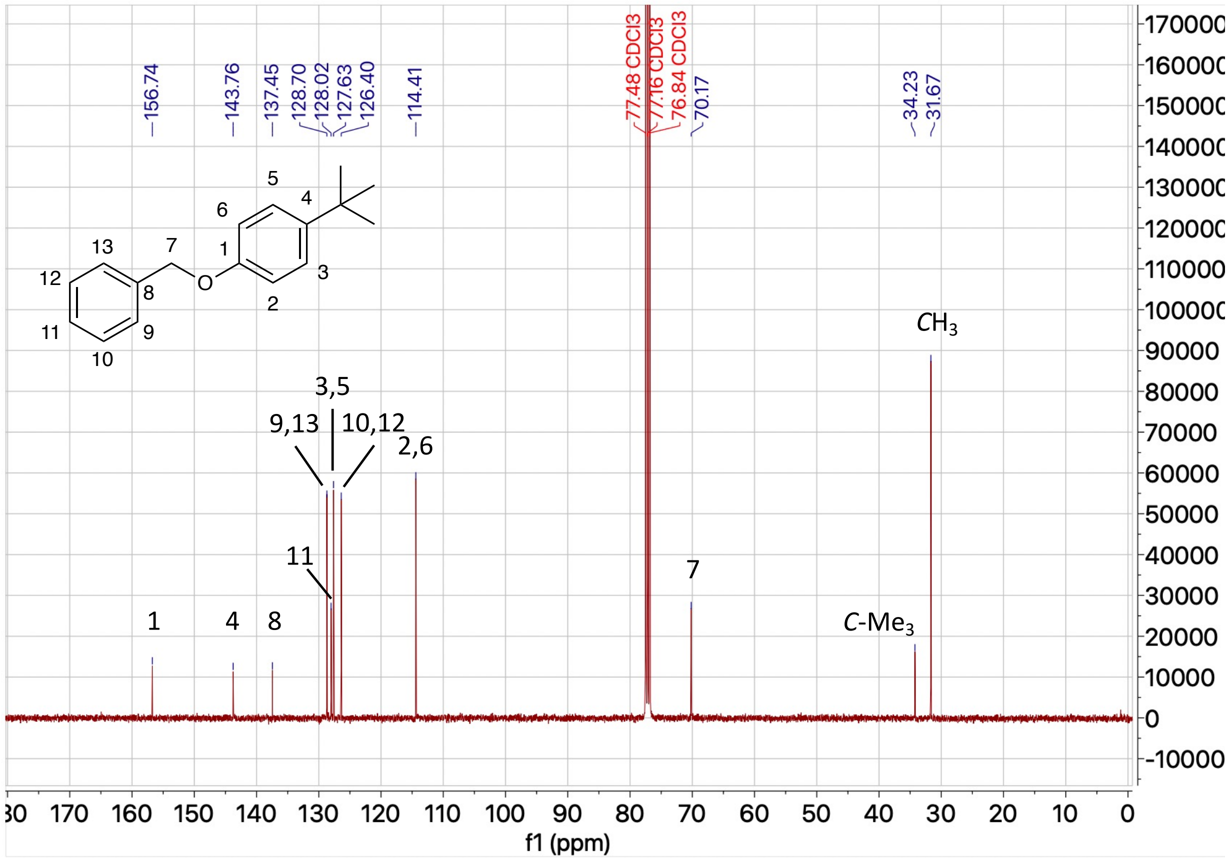

Figure S35: ^{13}C NMR spectrum (101 MHz) of **HP 2** in $CDCl_3$.

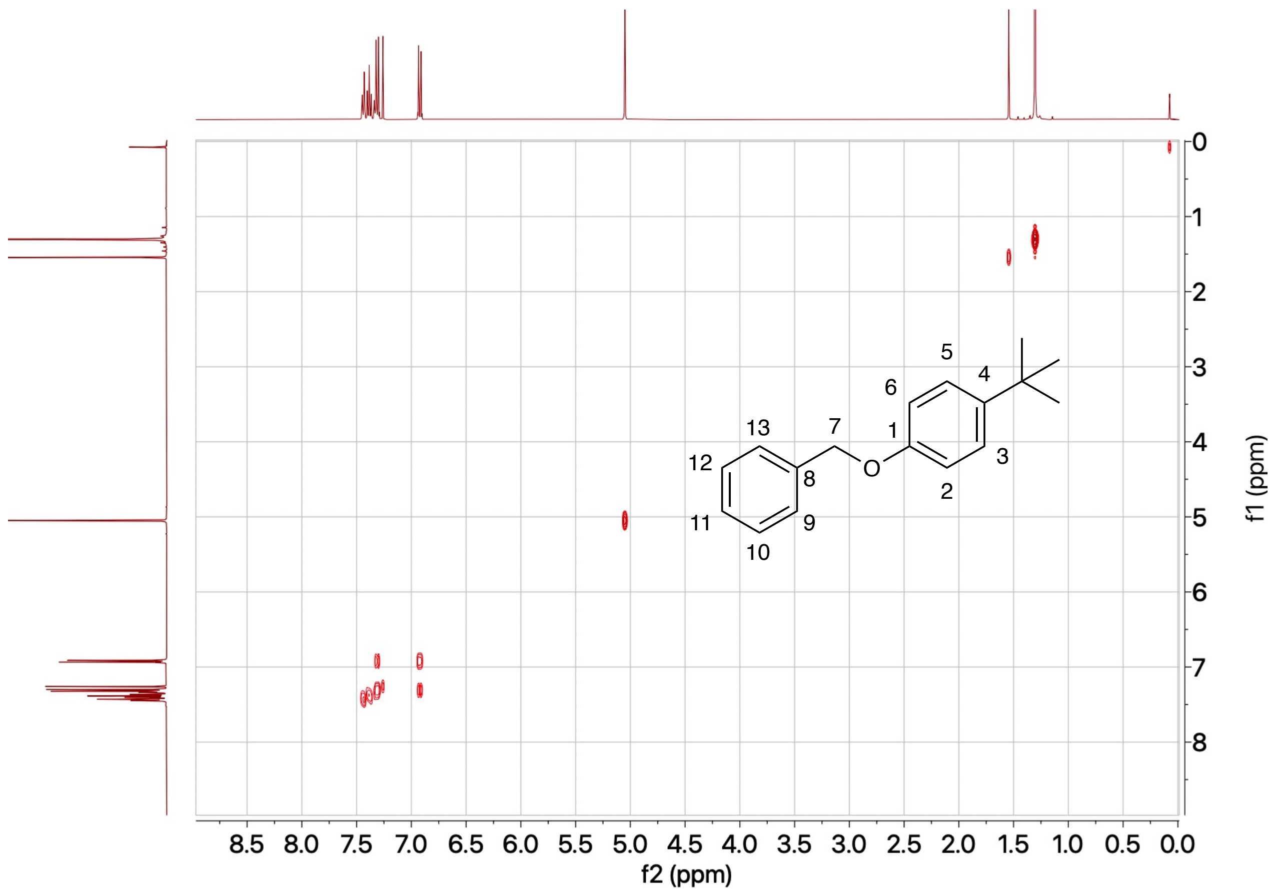

Figure S36: COSY spectrum (400 MHz) of **HP 2** in $CDCl_3$.

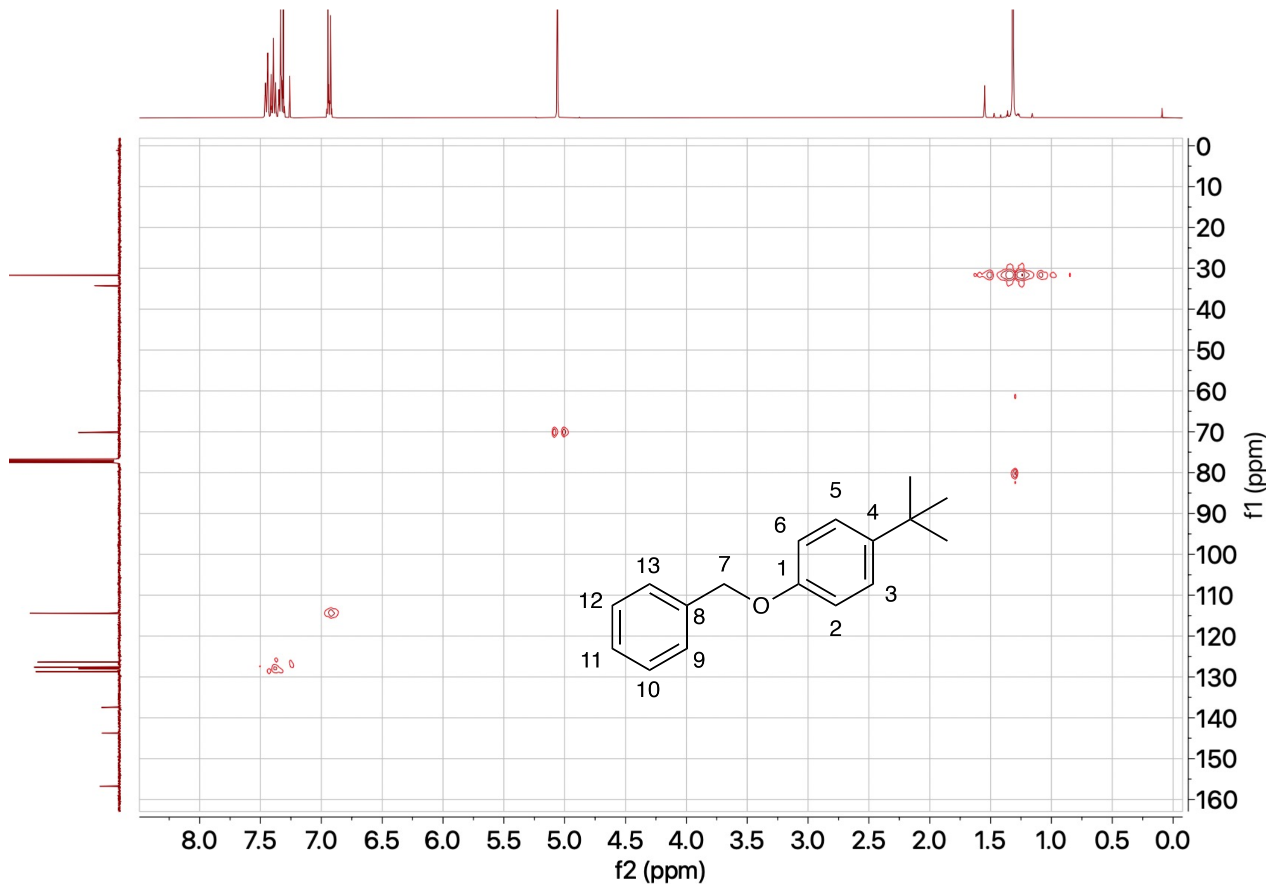

Figure S37: HMQC spectrum of **HP 2** in $CDCl_3$.

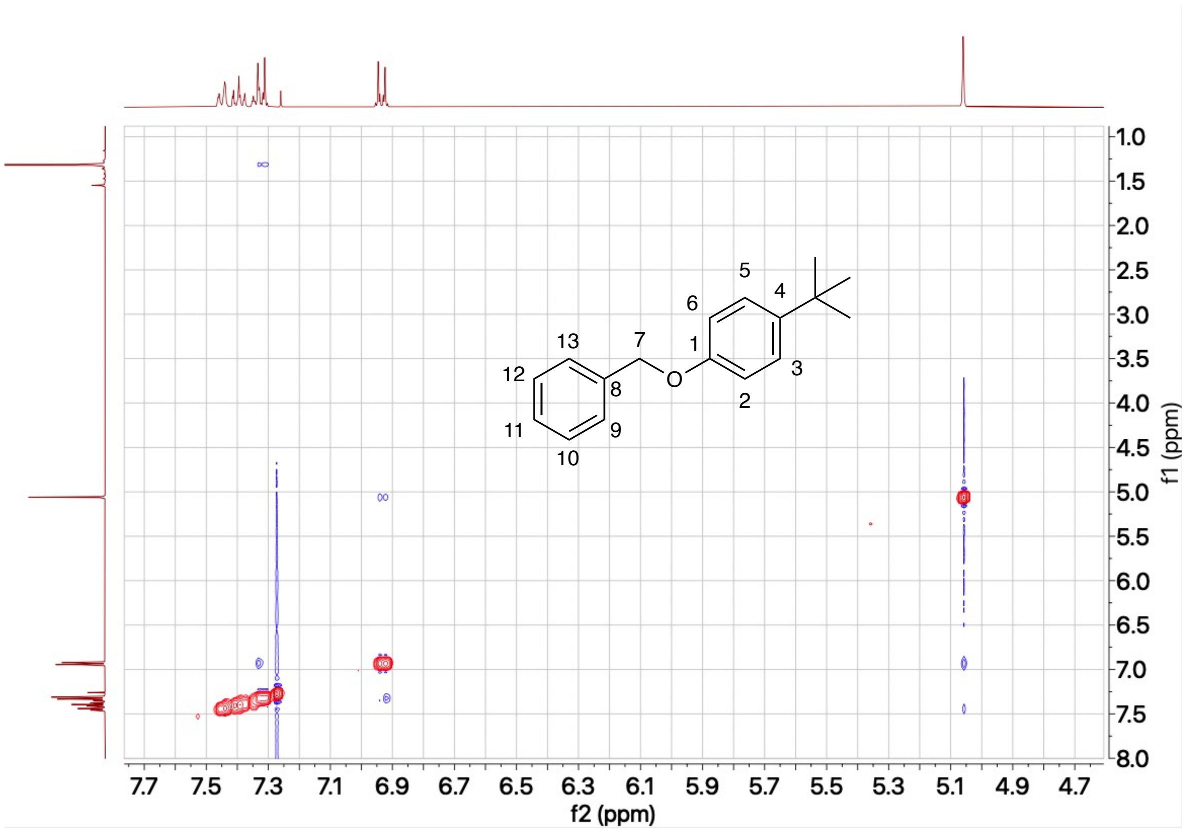

Figure S38: NOESY spectrum of **HP 2** in $CDCl_3$.

2-Methoxy-6H-benzo[c]chromene (CP 3)

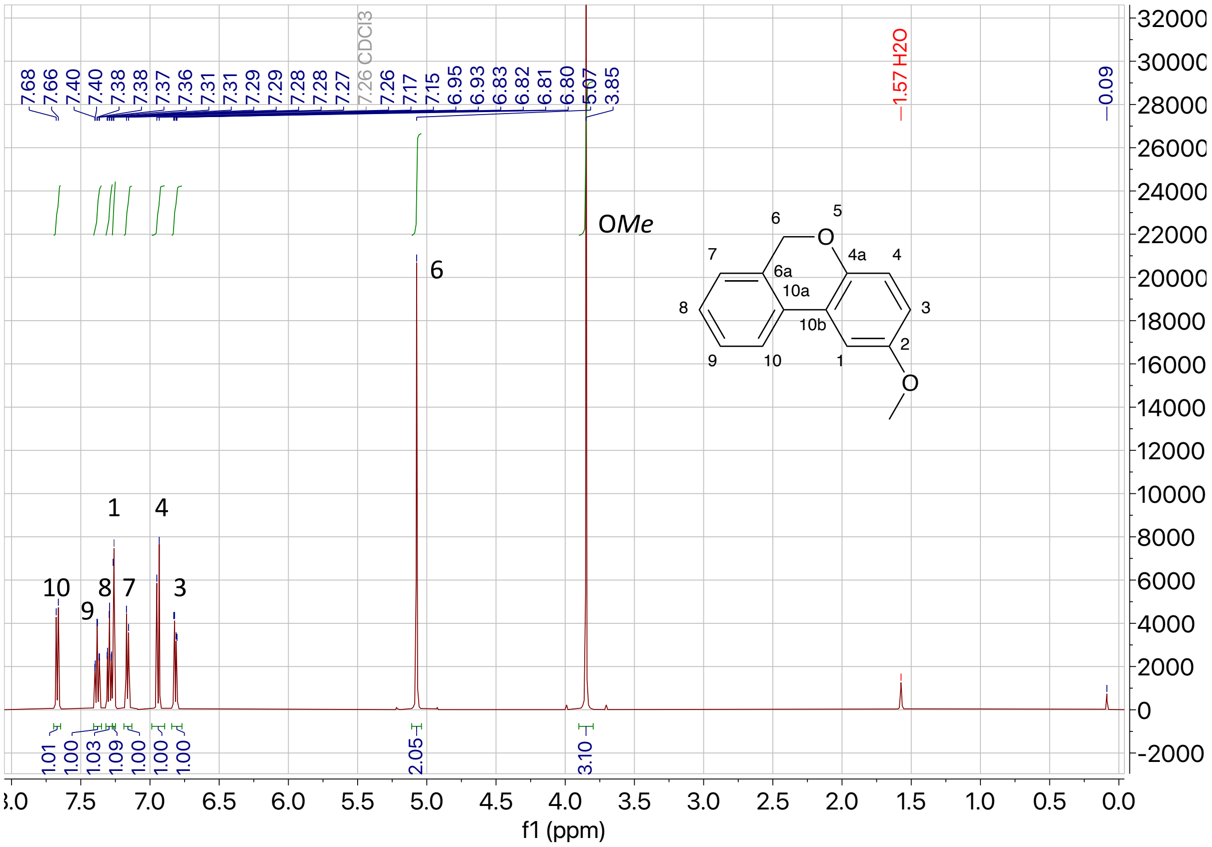

Figure S39: ^1H NMR spectrum (400 MHz) of **CP 3** in $CDCl_3$.

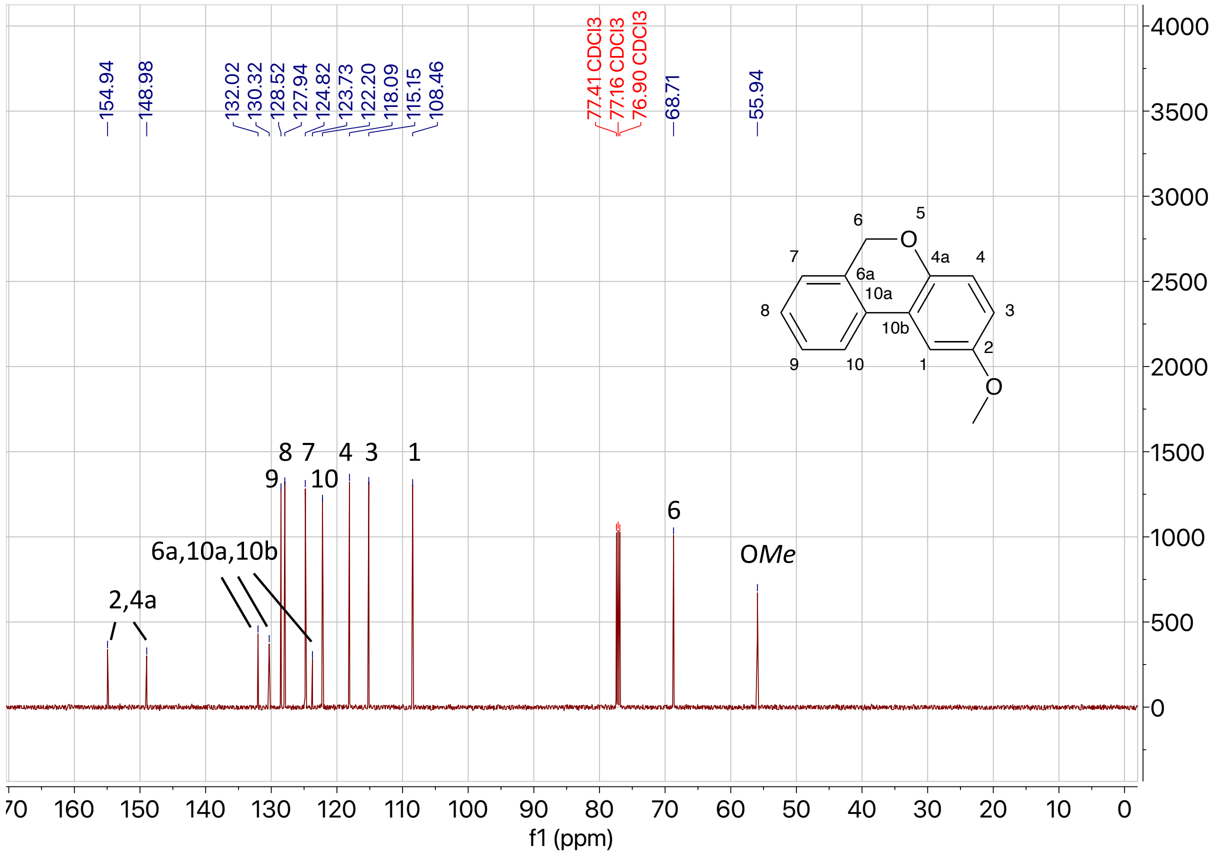

Figure S40: ^{13}C NMR spectrum (101 MHz) of **CP 3** in $CDCl_3$.

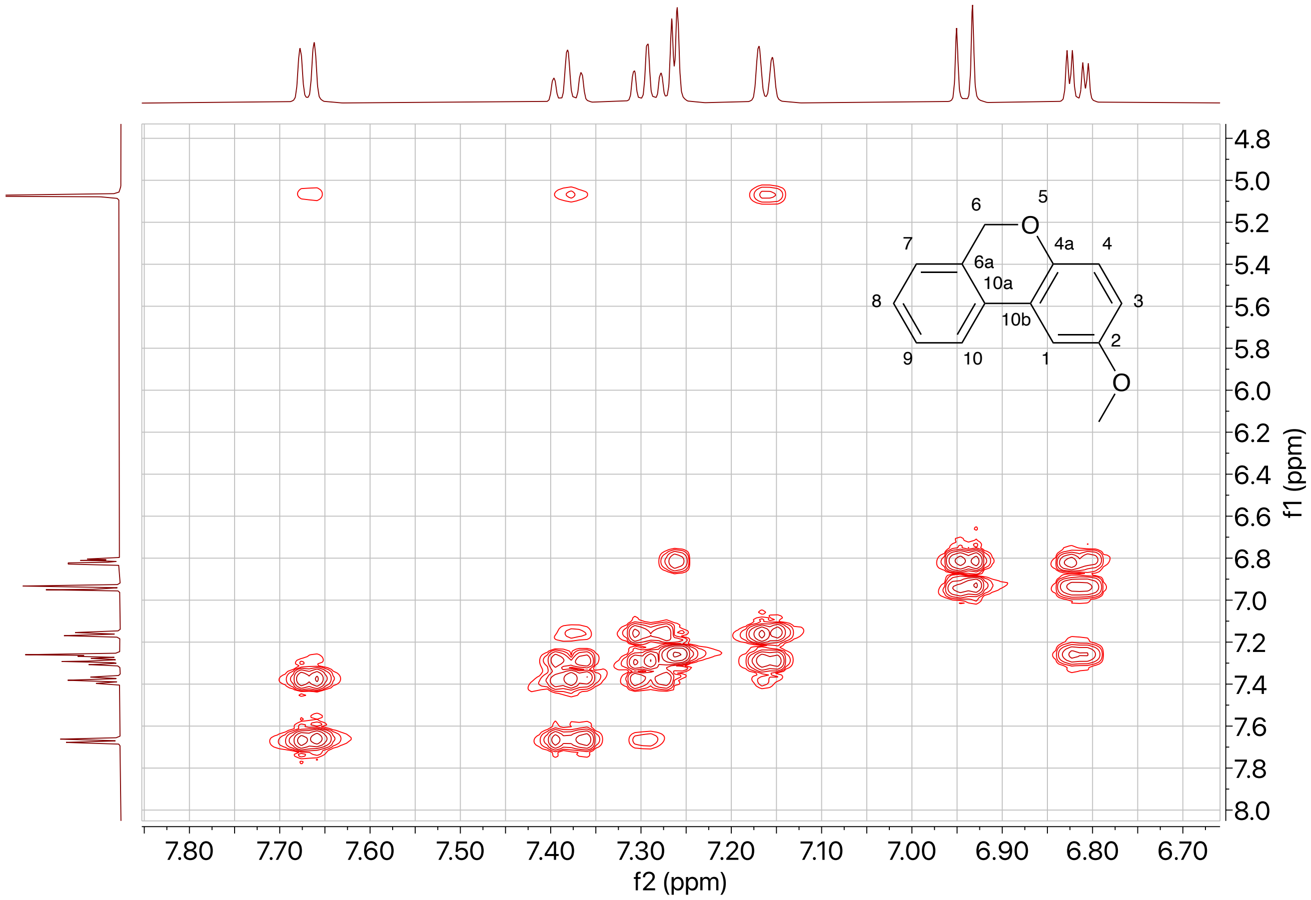

Figure S41: COSY spectrum (400 MHz) of **CP 3** in $CDCl_3$.

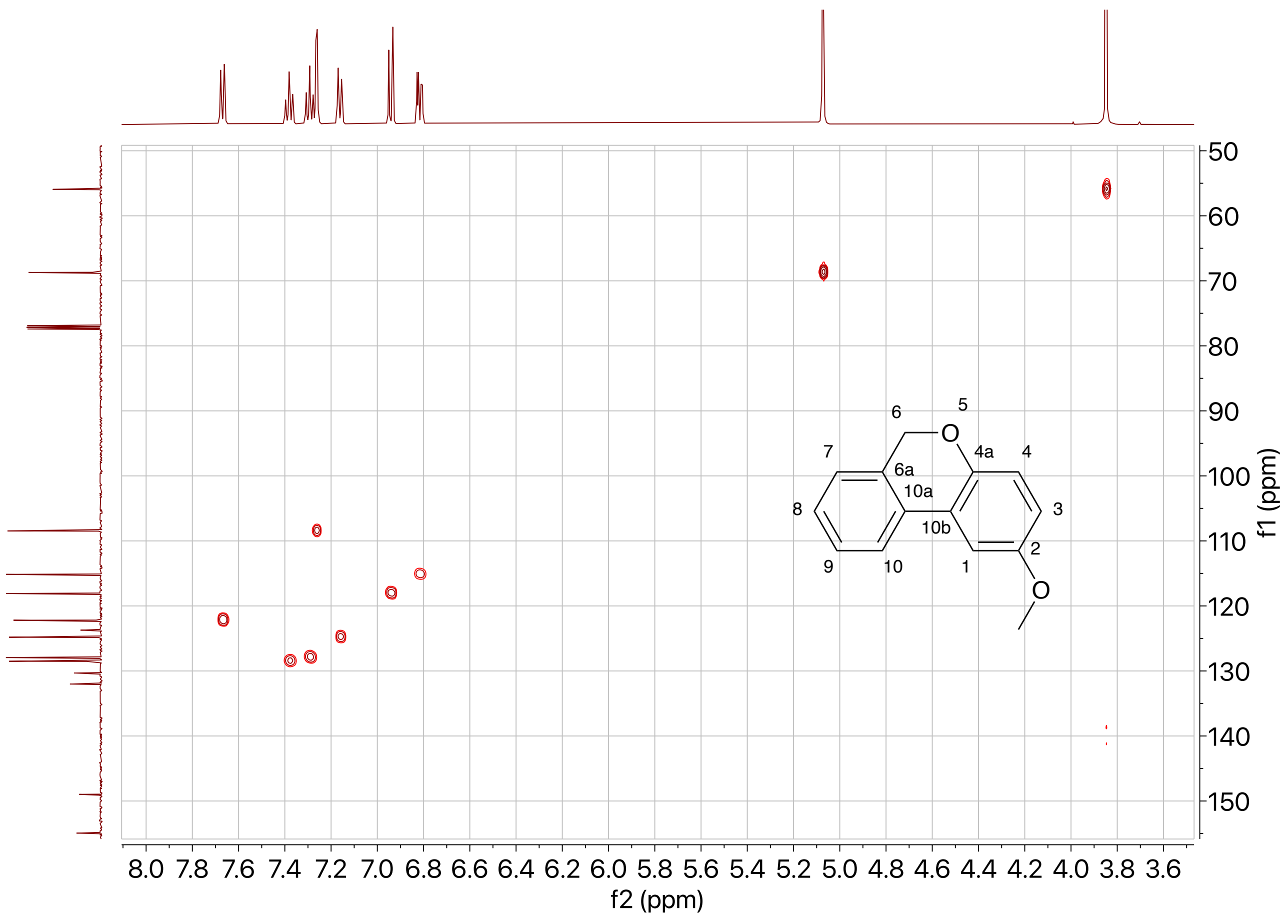

Figure S42: HMQC spectrum of **CP 3** in $CDCl_3$.

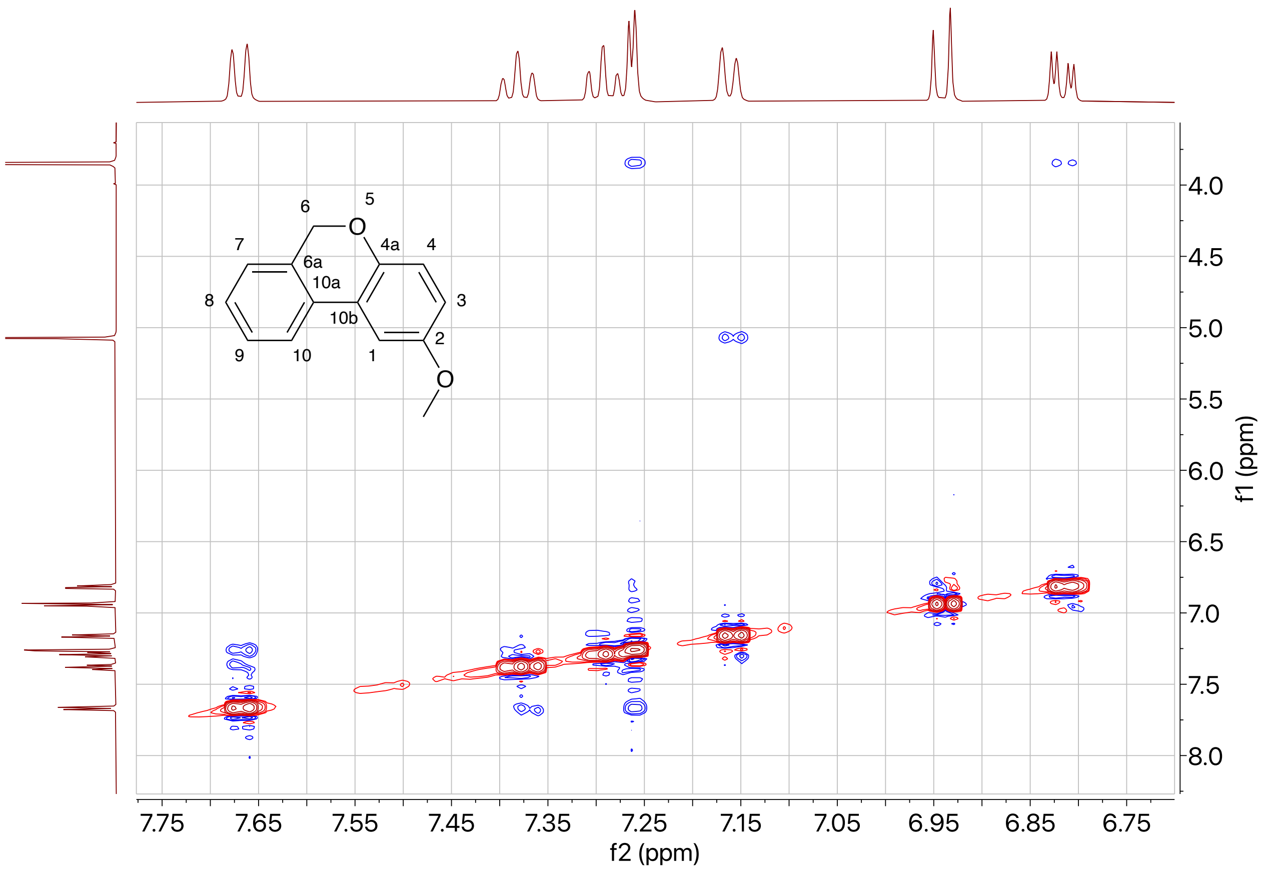

Figure S43: NOESY spectrum of **CP 3** in $CDCl_3$.

1-(Benzyloxy)-4-methoxybenzene (HP 3)

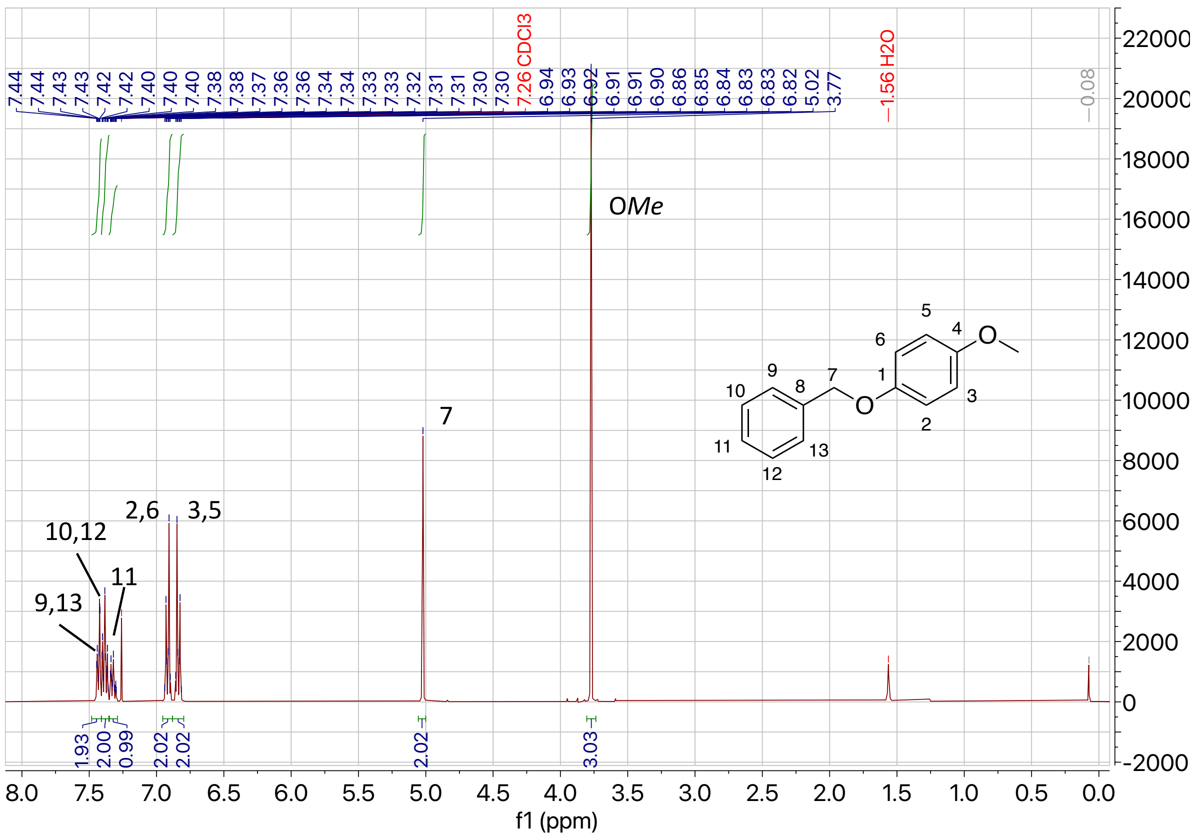

Figure S44: ^{1}H NMR spectrum (400 MHz) of **HP 3** in $CDCl_3$.

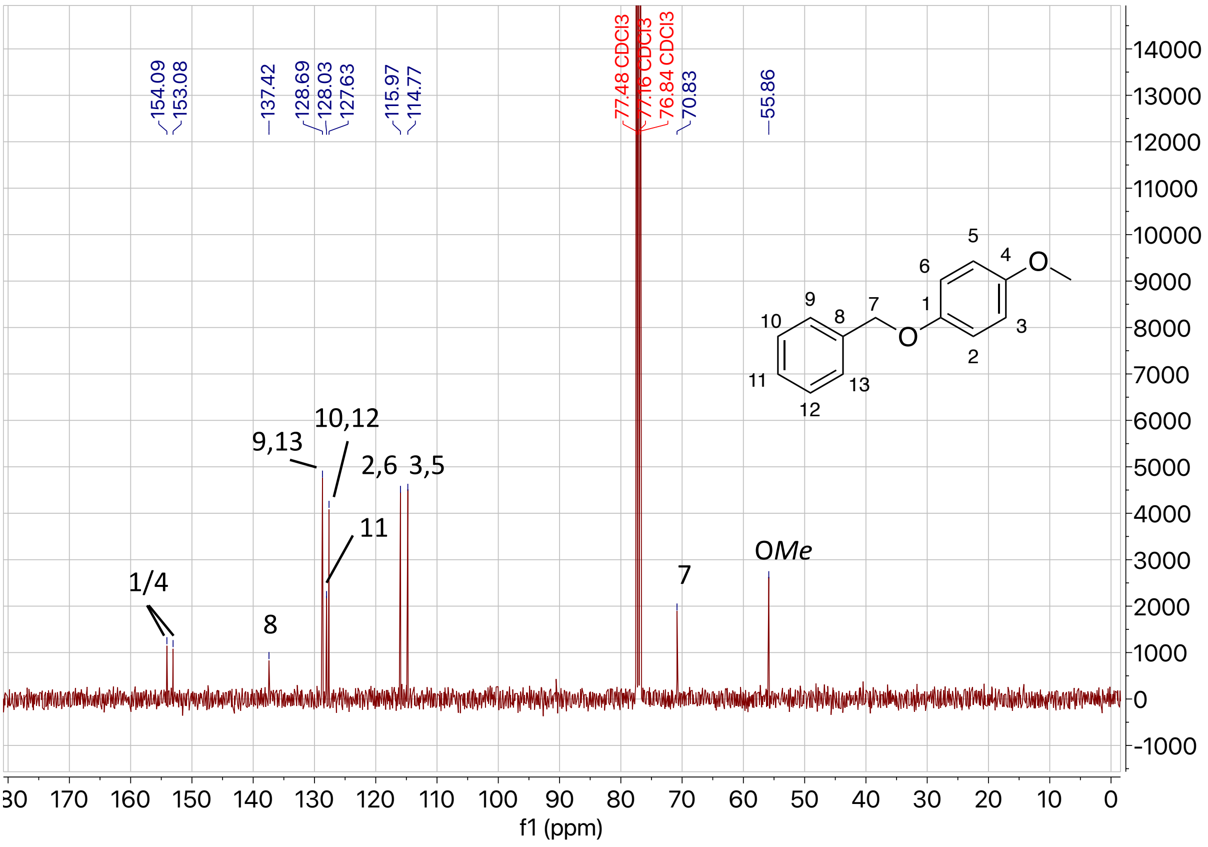

Figure S45: ^{13}C NMR spectrum (101 MHz) of **HP 3** in $CDCl_3$

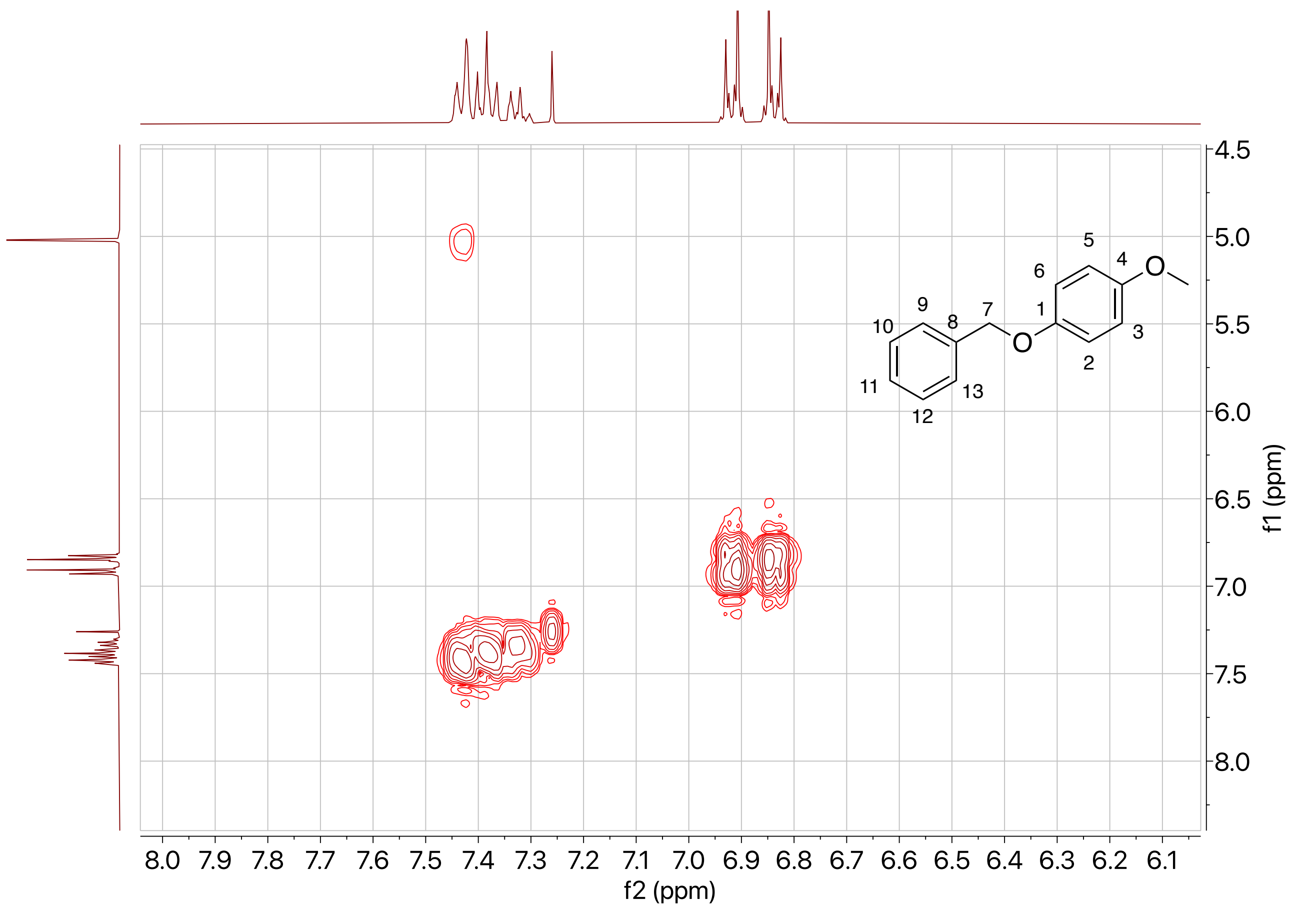

Figure S46: COSY spectrum (400 MHz) of **HP 3** in $CDCl_3$.

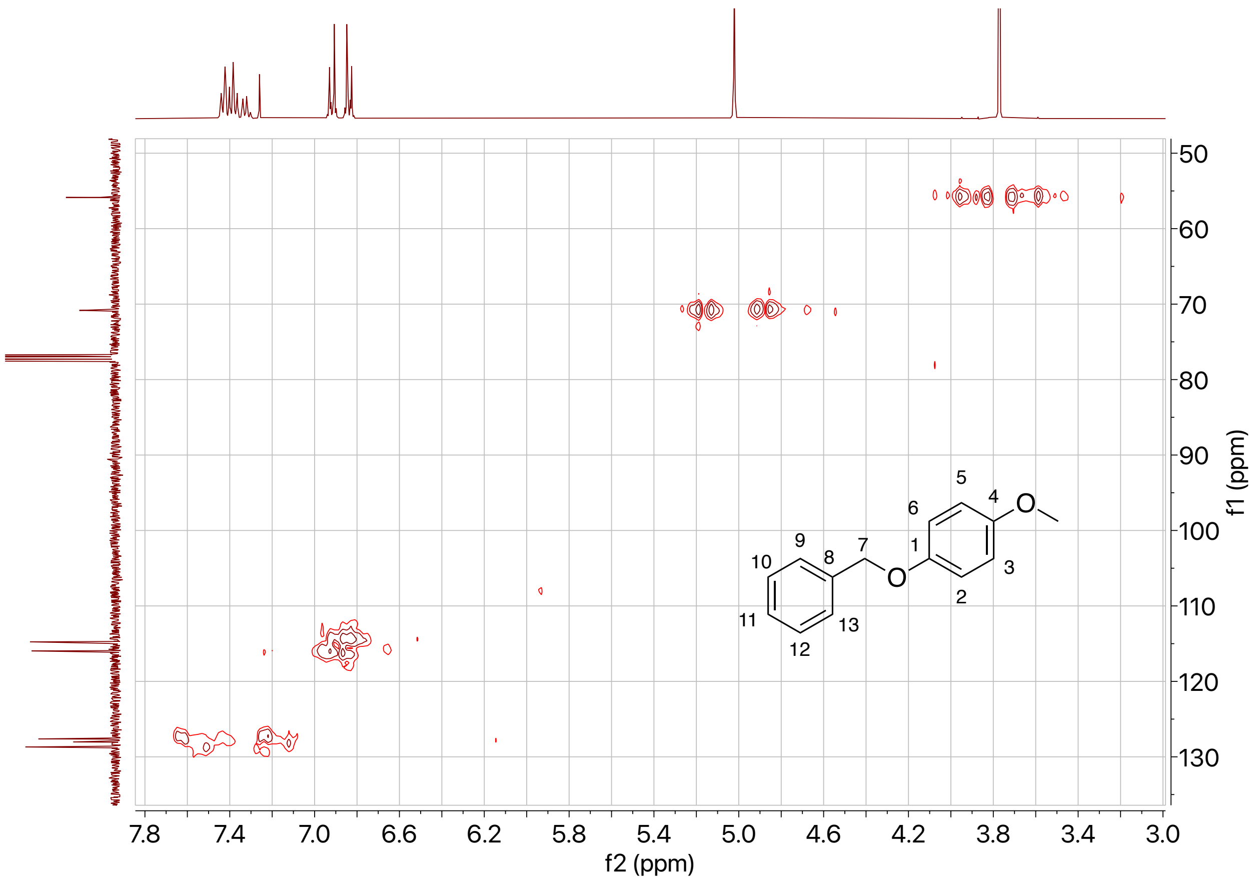

Figure S47: HMQC spectrum of **HP 3** in $CDCl_3$.

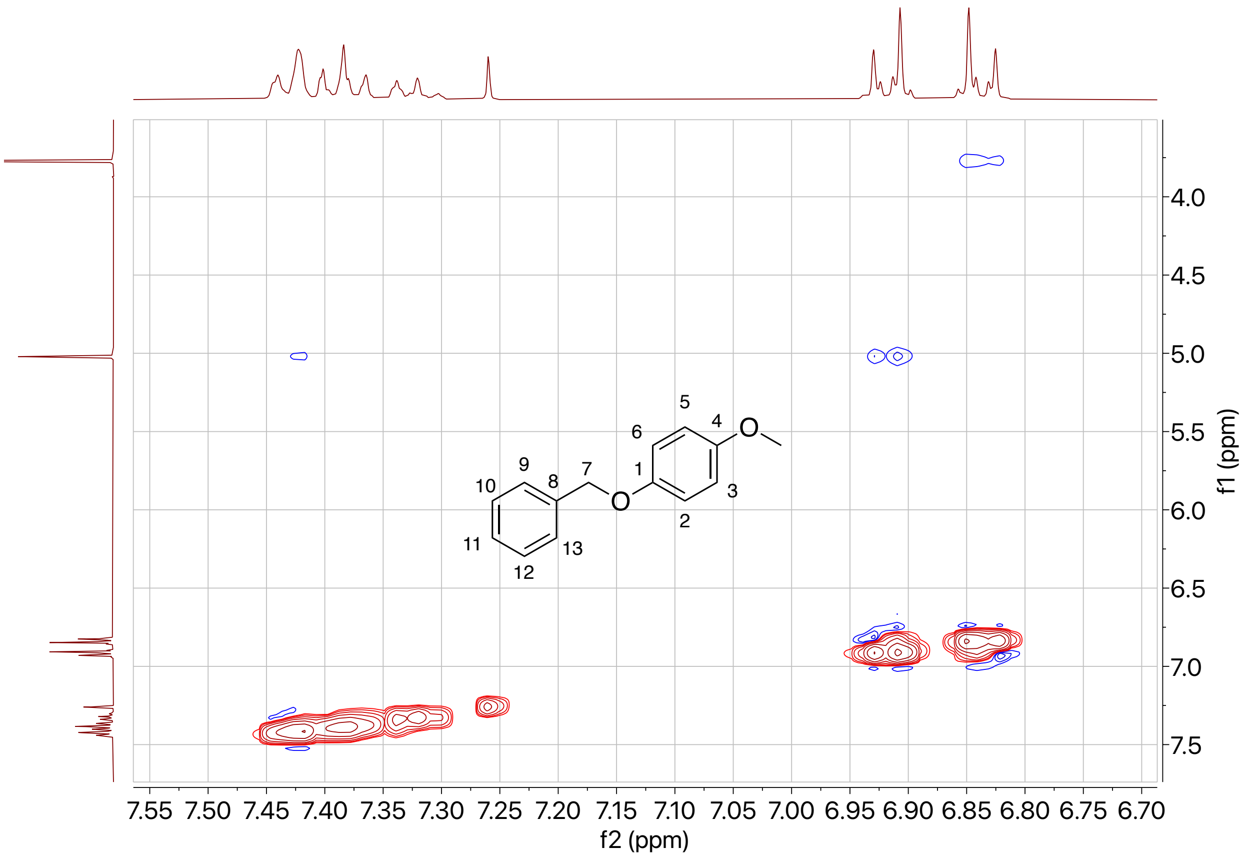

Figure S48: NOESY spectrum of **HP 3** in $CDCl_3$.

2-Methyl-6H-benzo[c]chromene (CP 4)

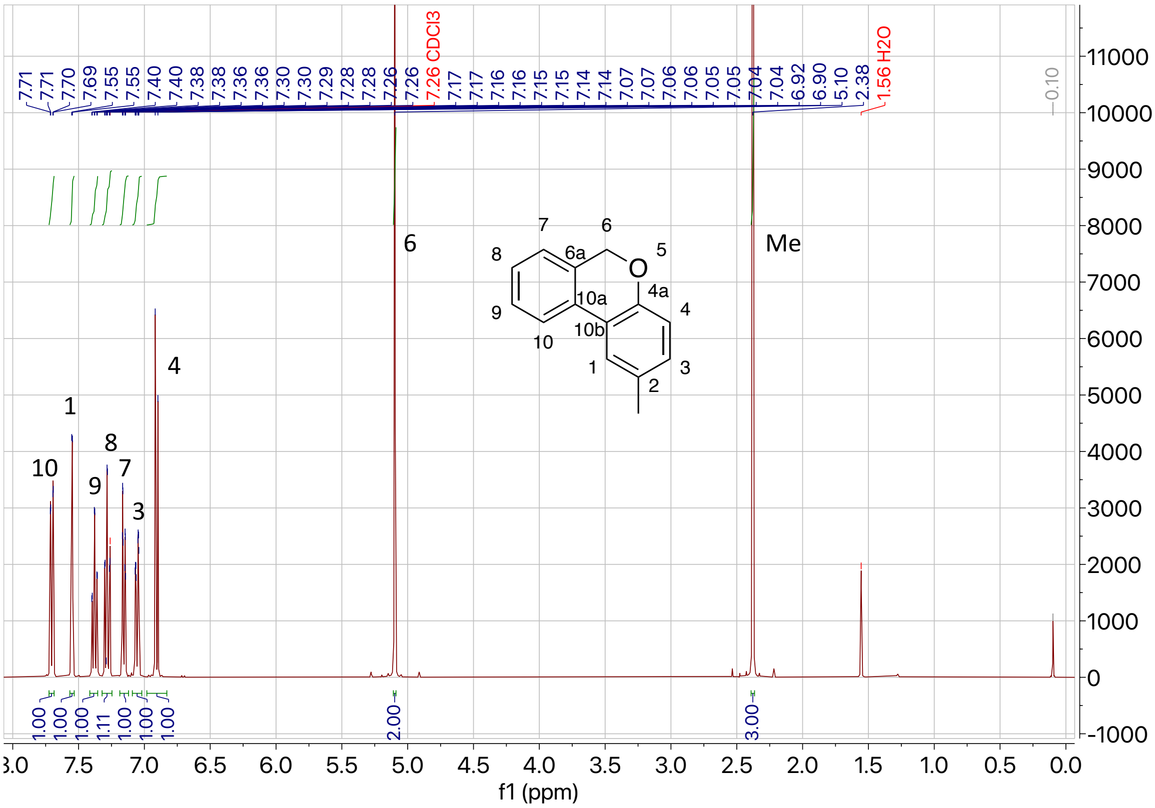

Figure S49: ^1H NMR spectrum (400 MHz) of **CP 4** in $CDCl_3$.

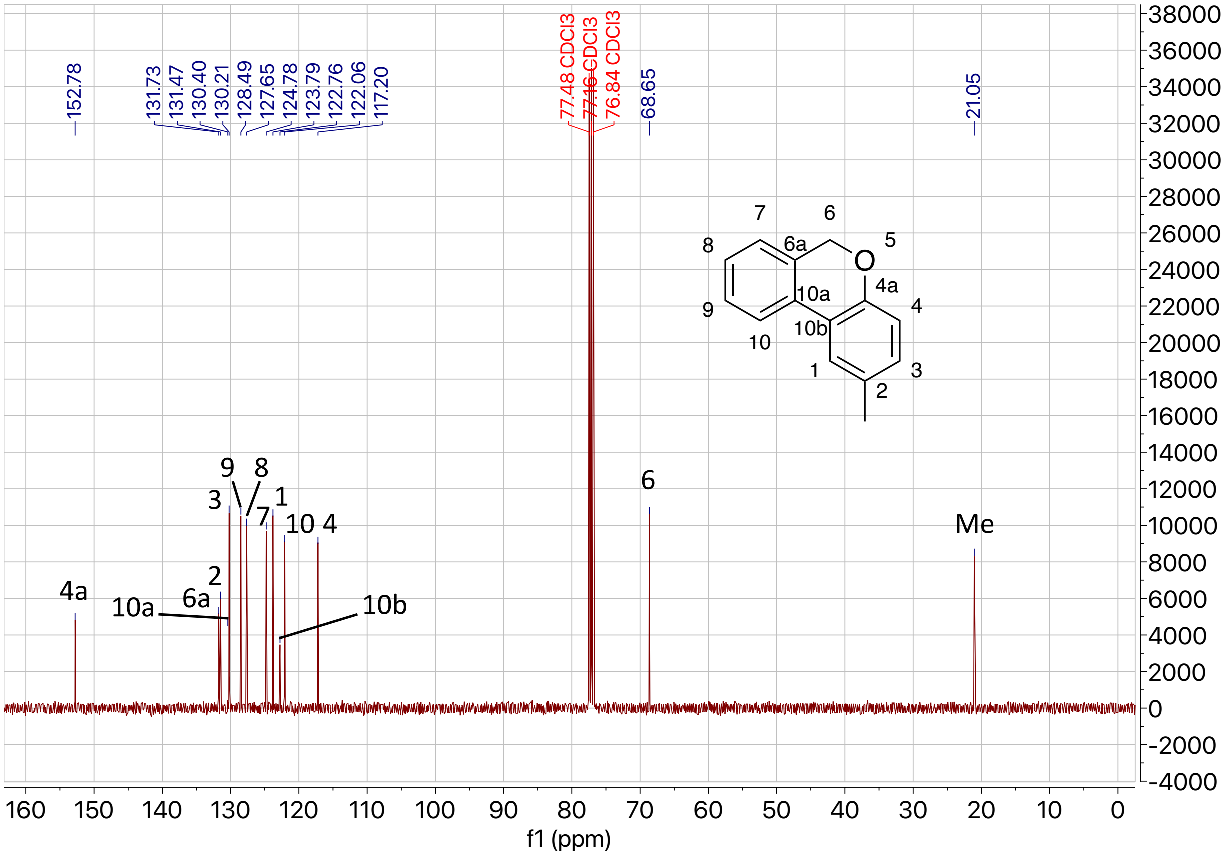

Figure S50: ^{13}C NMR spectrum (101 MHz) of **CP 4** in $CDCl_3$

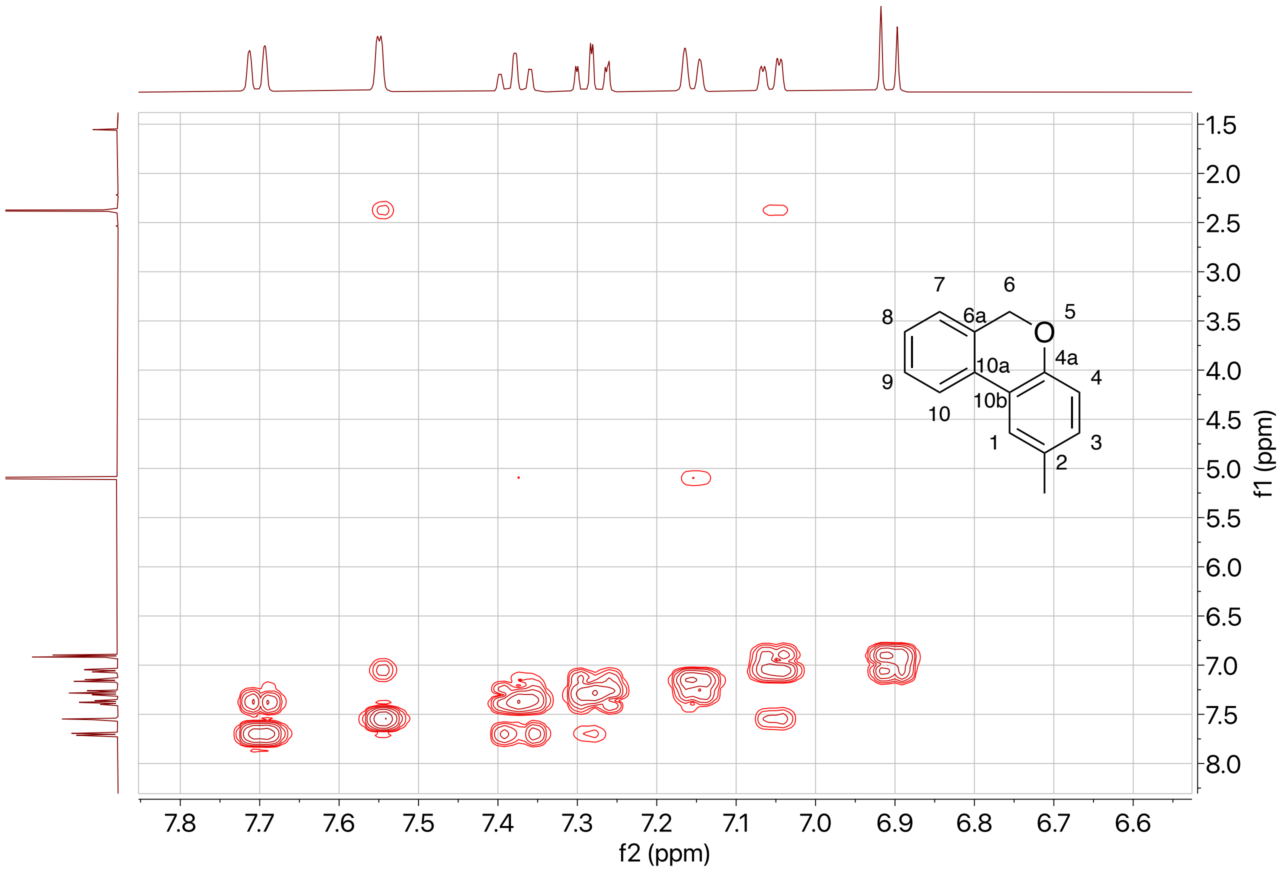

Figure S51: COSY spectrum (400 MHz) of **CP 4** in $CDCl_3$.

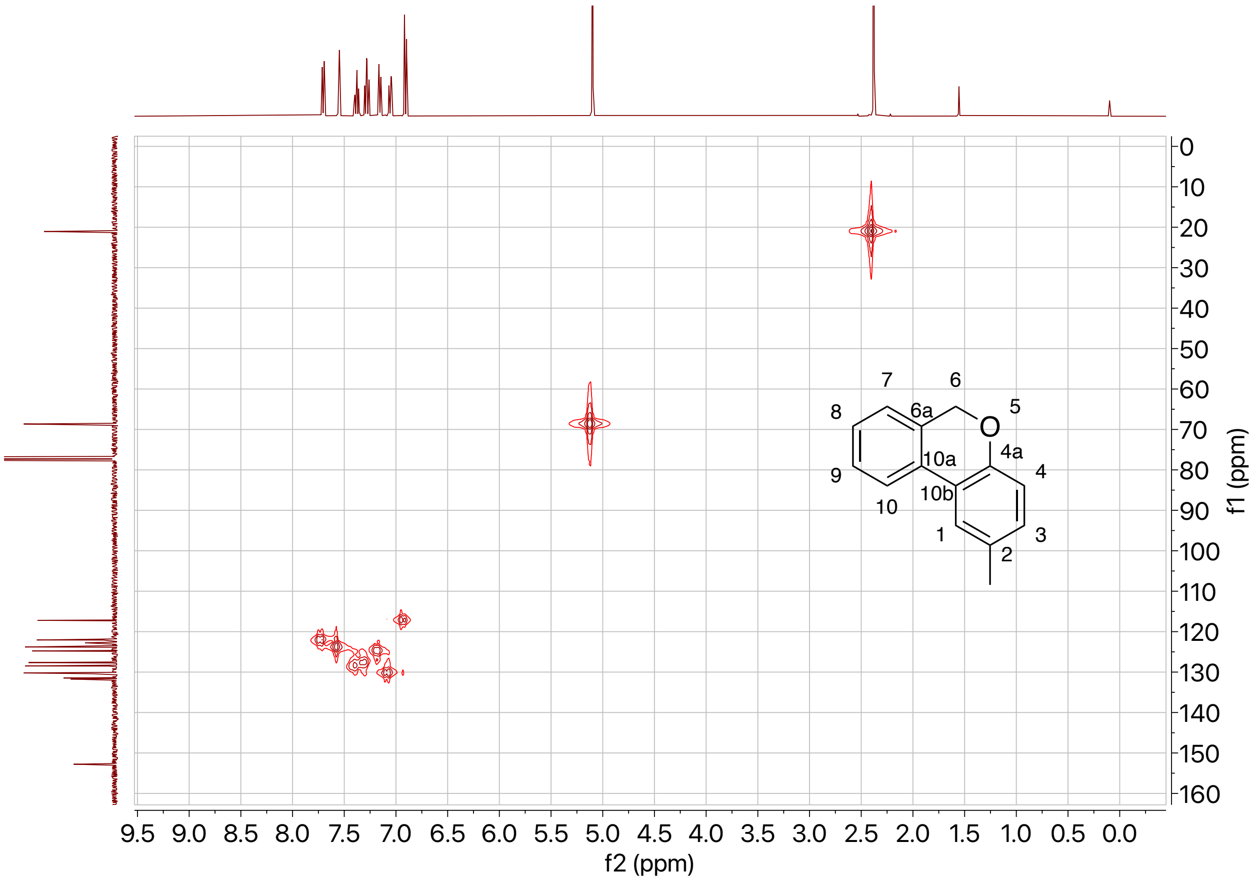

Figure S52: HMQC spectrum of **CP 4** in $CDCl_3$.

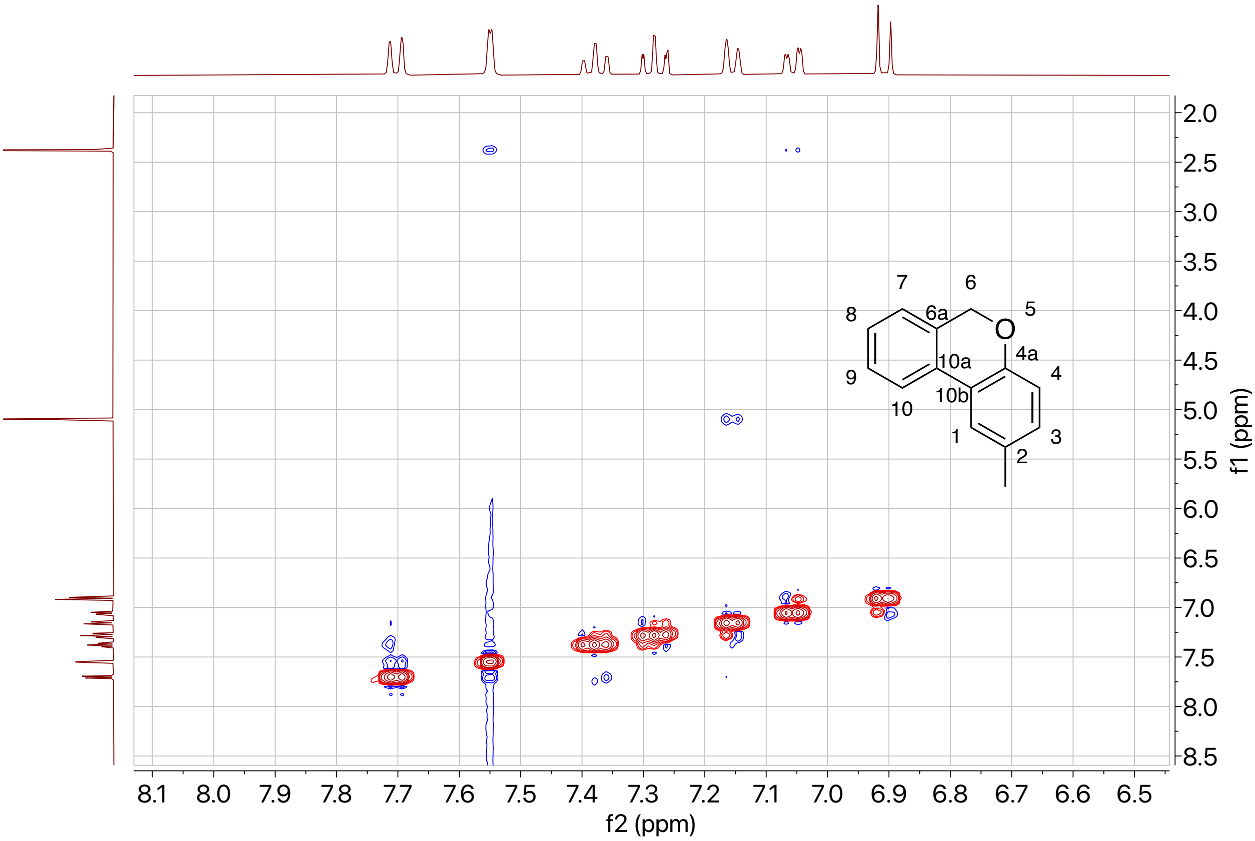

Figure S53: NOESY spectrum of **CP 4** in $CDCl_3$.

1-(Benzyloxy)-4-methylbenzene (HP 4)

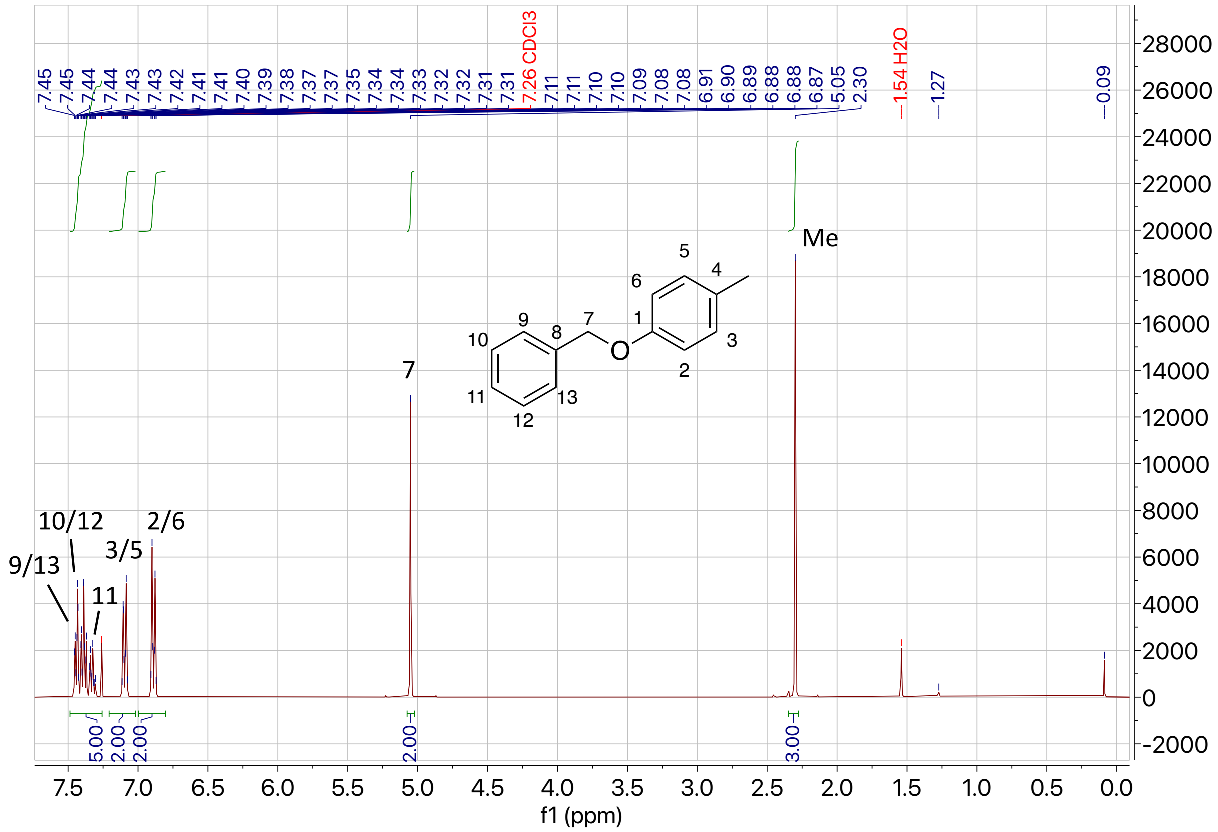

Figure S54: ^{1}H NMR spectrum (400 MHz) of **HP 4** in $CDCl_3$.

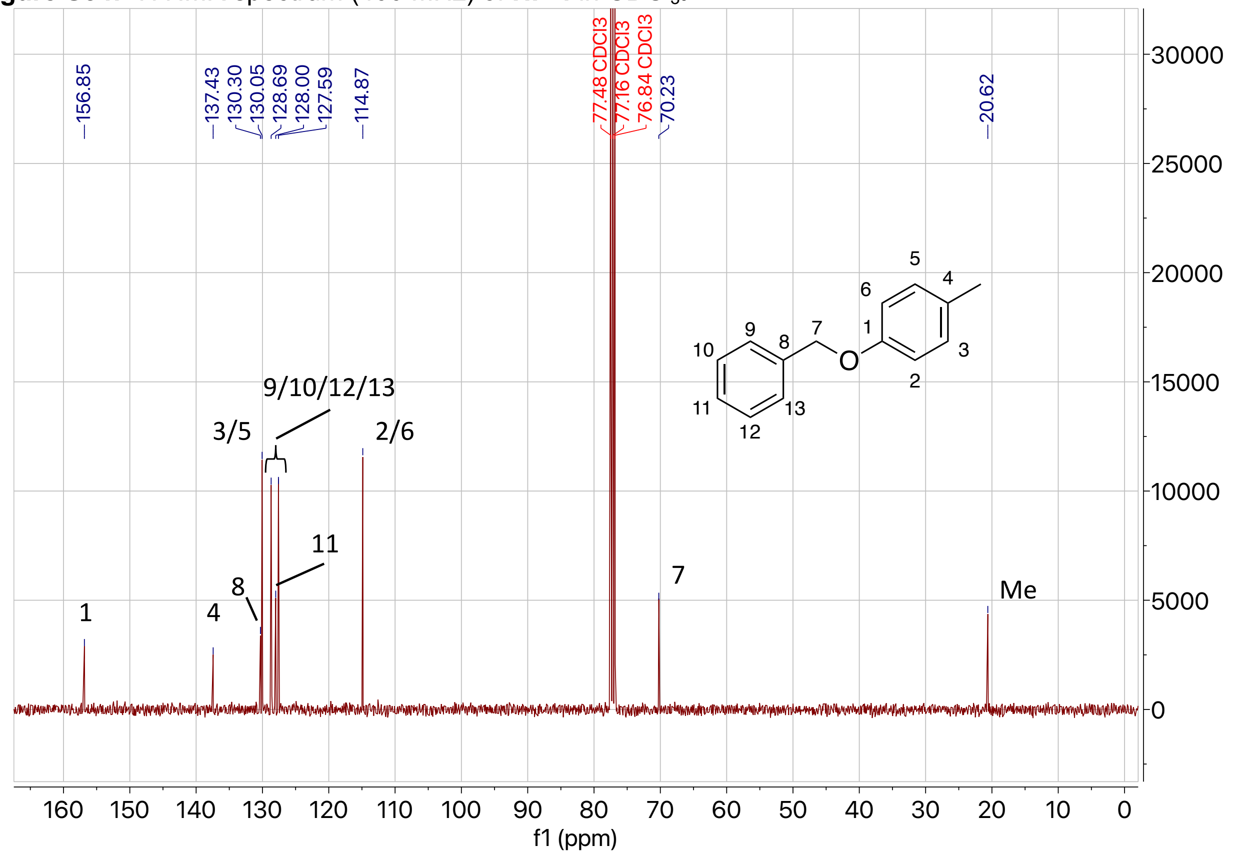

Figure S55: ^{13}C NMR spectrum (101 MHz) of **HP 4** in $CDCl_3$

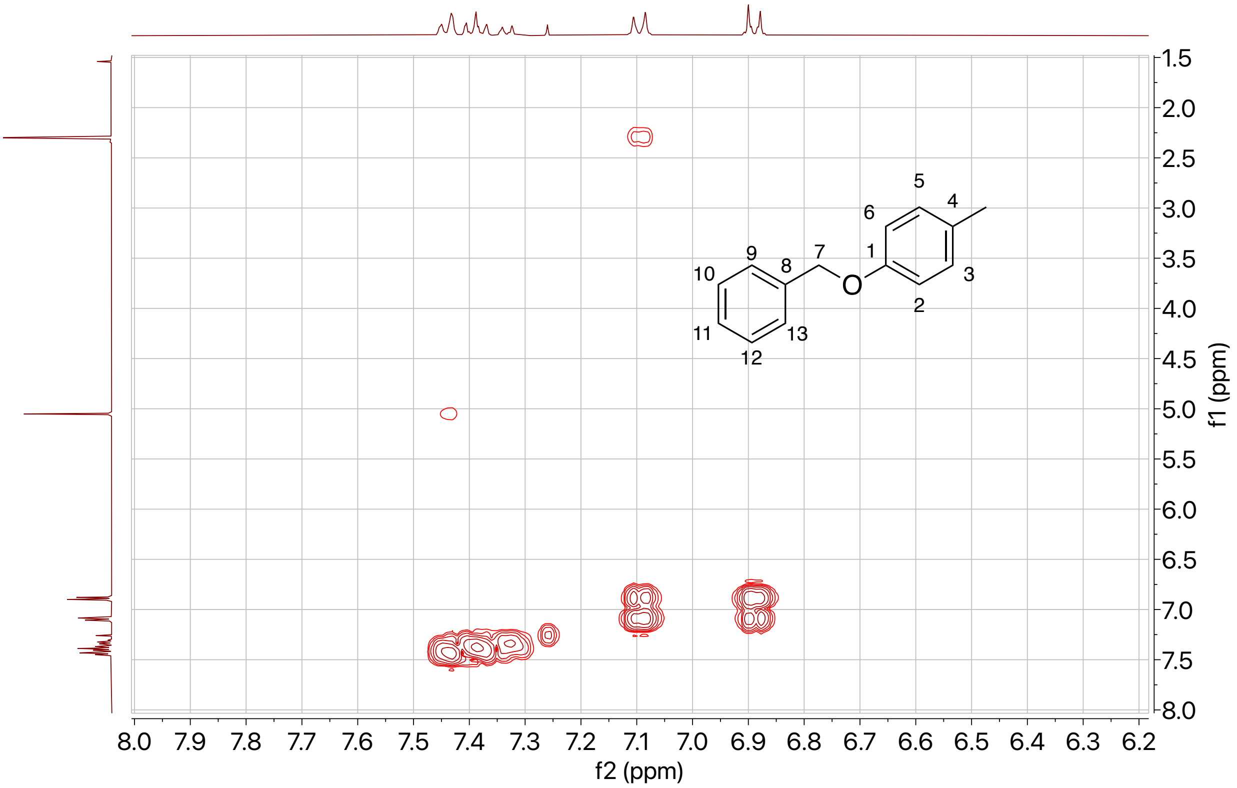

Figure S56: COSY spectrum (400 MHz) of **HP 4** in $CDCl_3$.

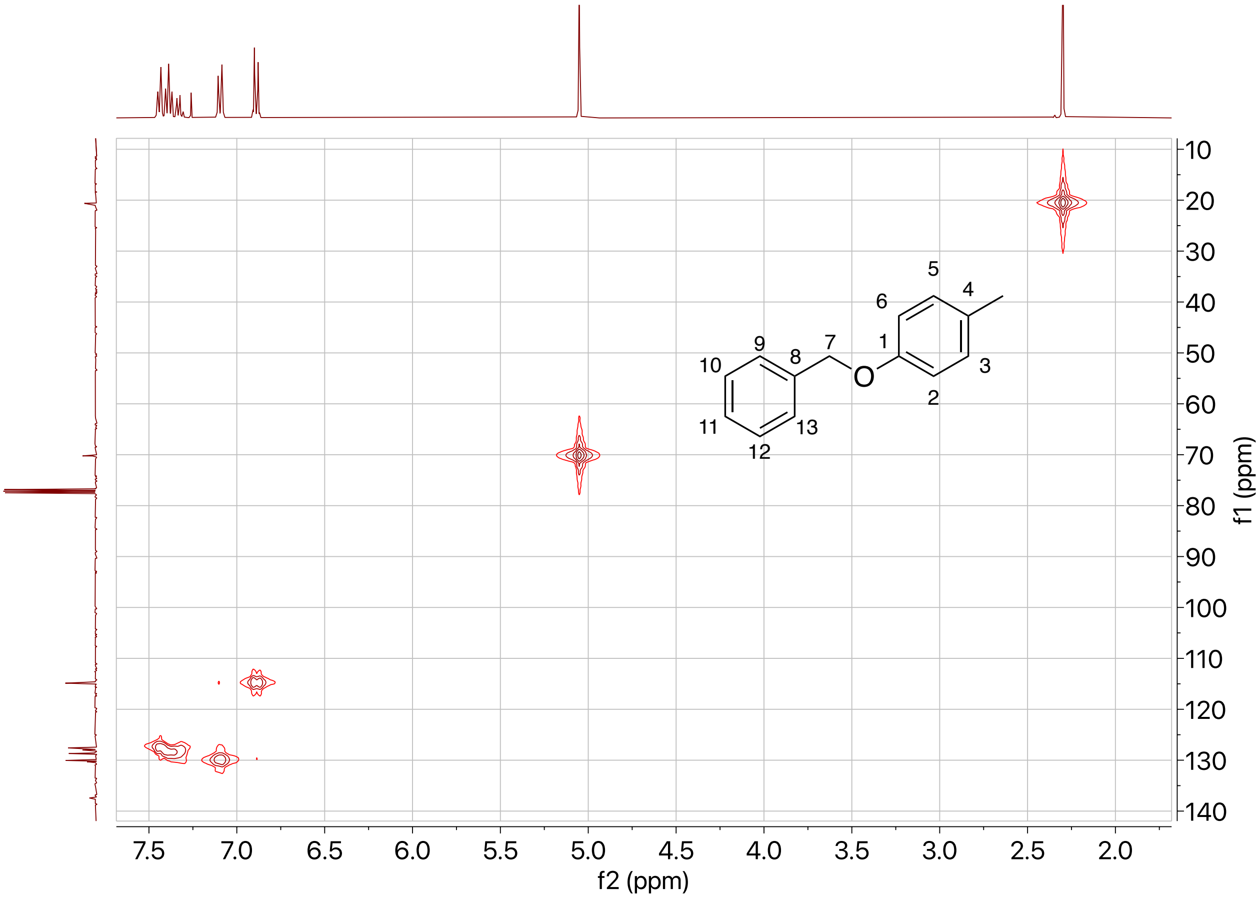

Figure S57: HMQC spectrum of **HP 4** in $CDCl_3$.

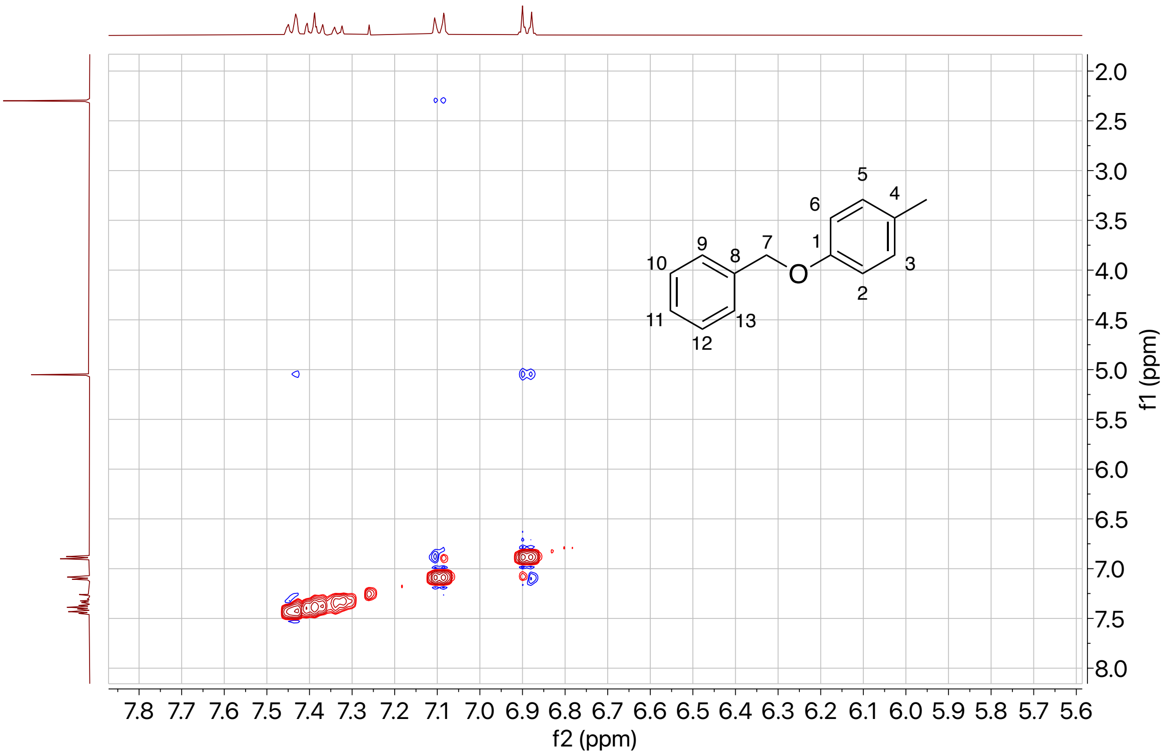

Figure S58: NOESY spectrum of **HP 4** in $CDCl_3$.

6H-benzo[c]chromene (CP 5)

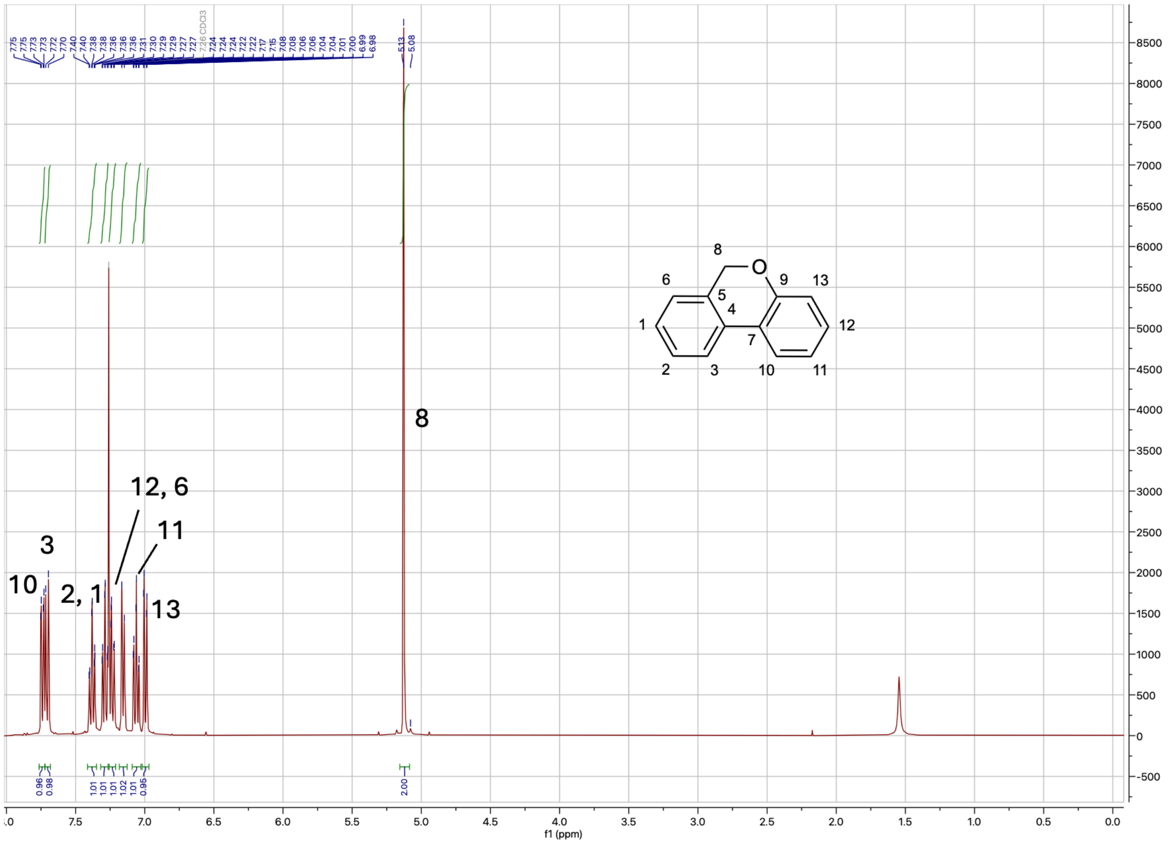

Figure S59: ^{1}H NMR spectrum (400 MHz) of **CP 5** in $CDCl_3$.

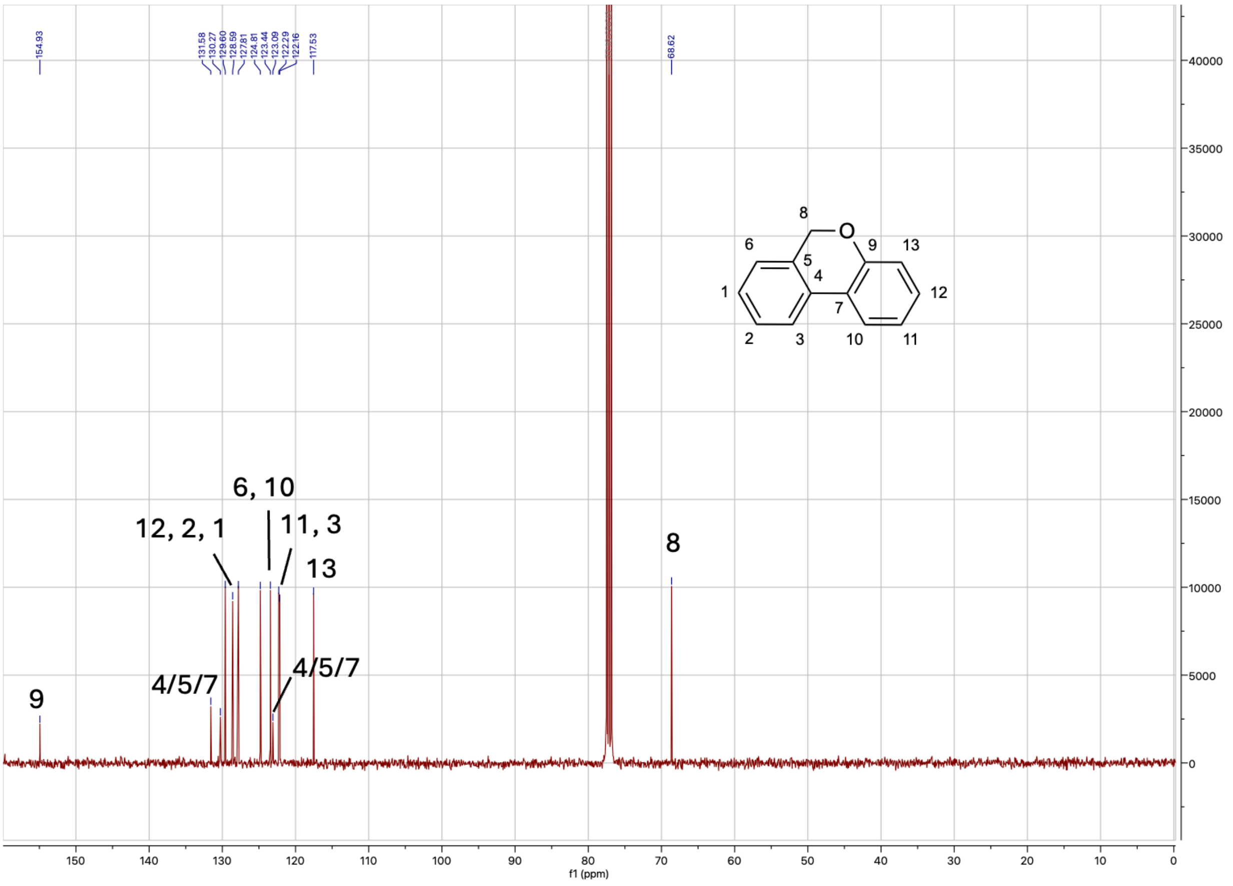

Figure S60: ^{13}C NMR spectrum (101 MHz) of **CP 5** in $CDCl_3$

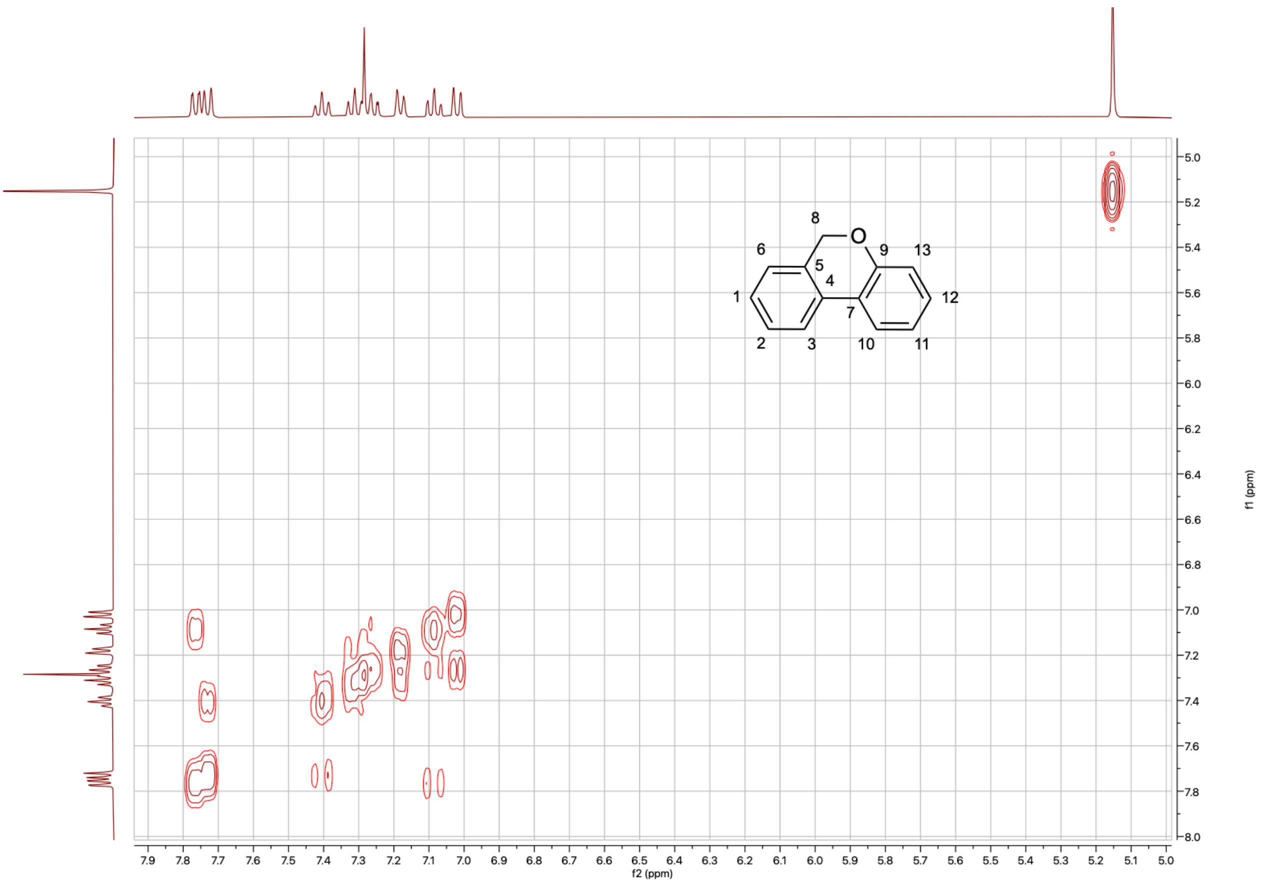

Figure S61: COSY spectrum (400 MHz) of **CP 5** in $CDCl_3$.

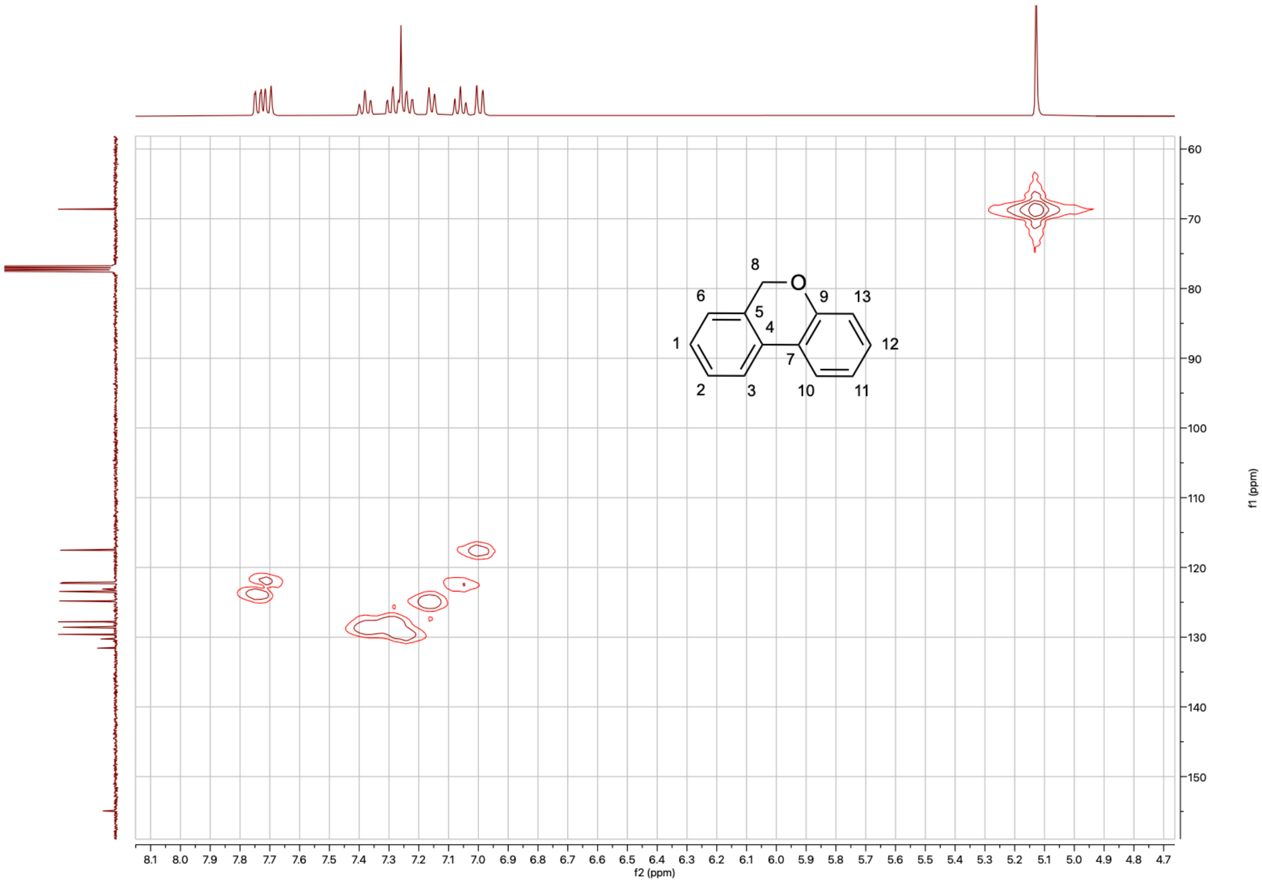

Figure S62: HMQC spectrum of **CP 5** in $CDCl_3$.

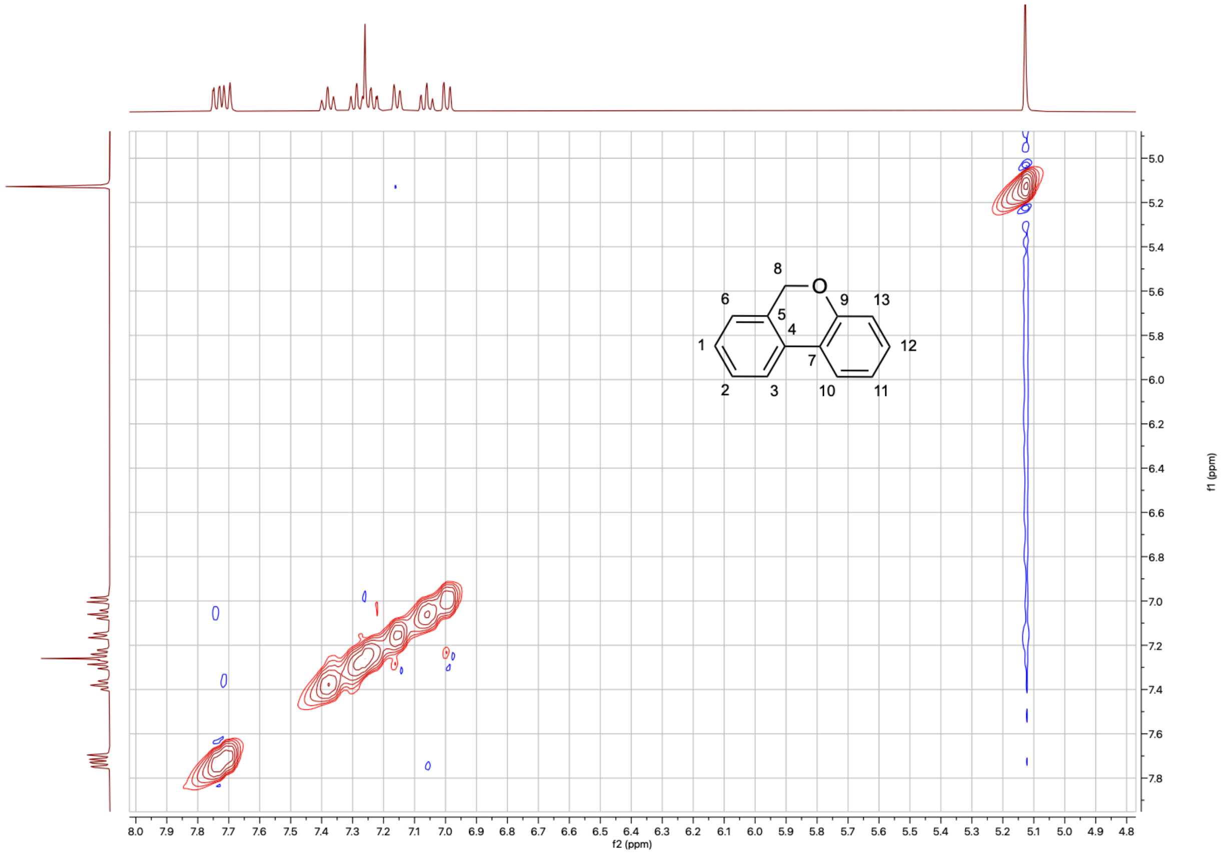

Figure S63: NOESY spectrum of **CP 5** in $CDCl_3$.

Benzyloxybenzene (HP 5)

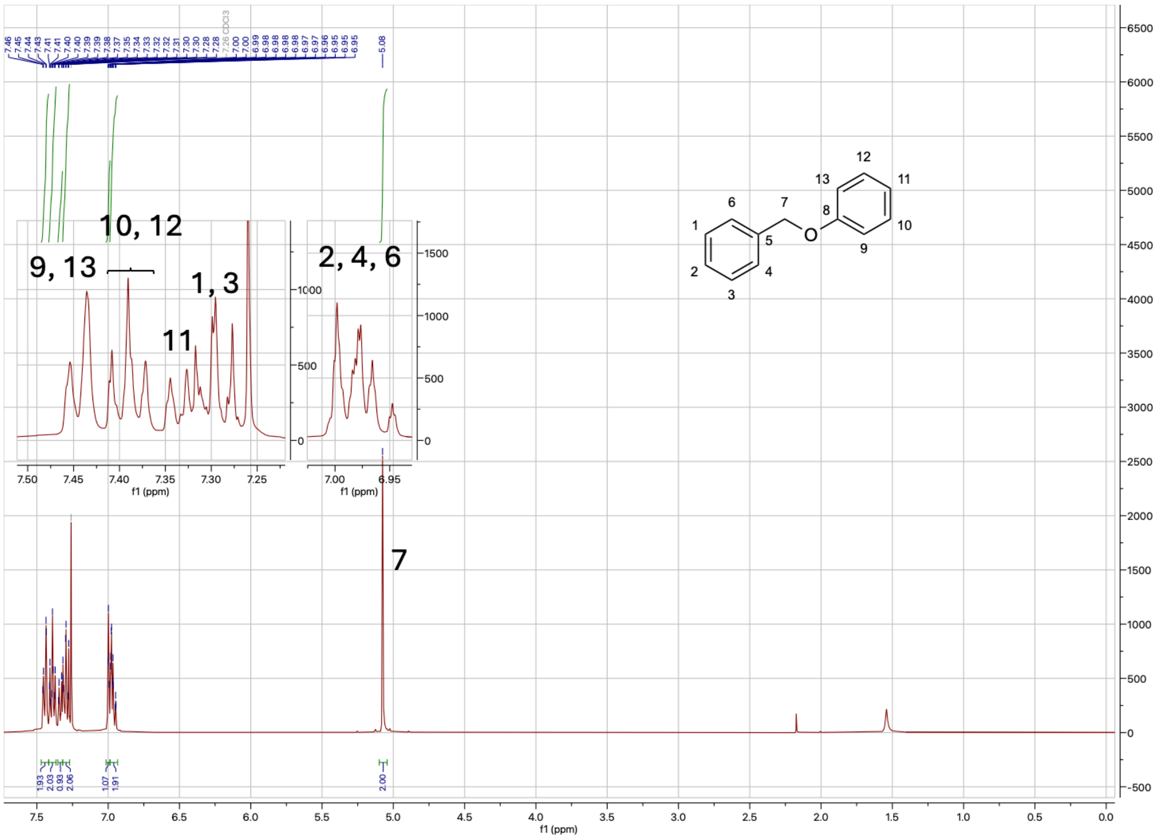

Figure S64: ^{1}H NMR spectrum (400 MHz) of **HP 5** in $CDCl_3$.

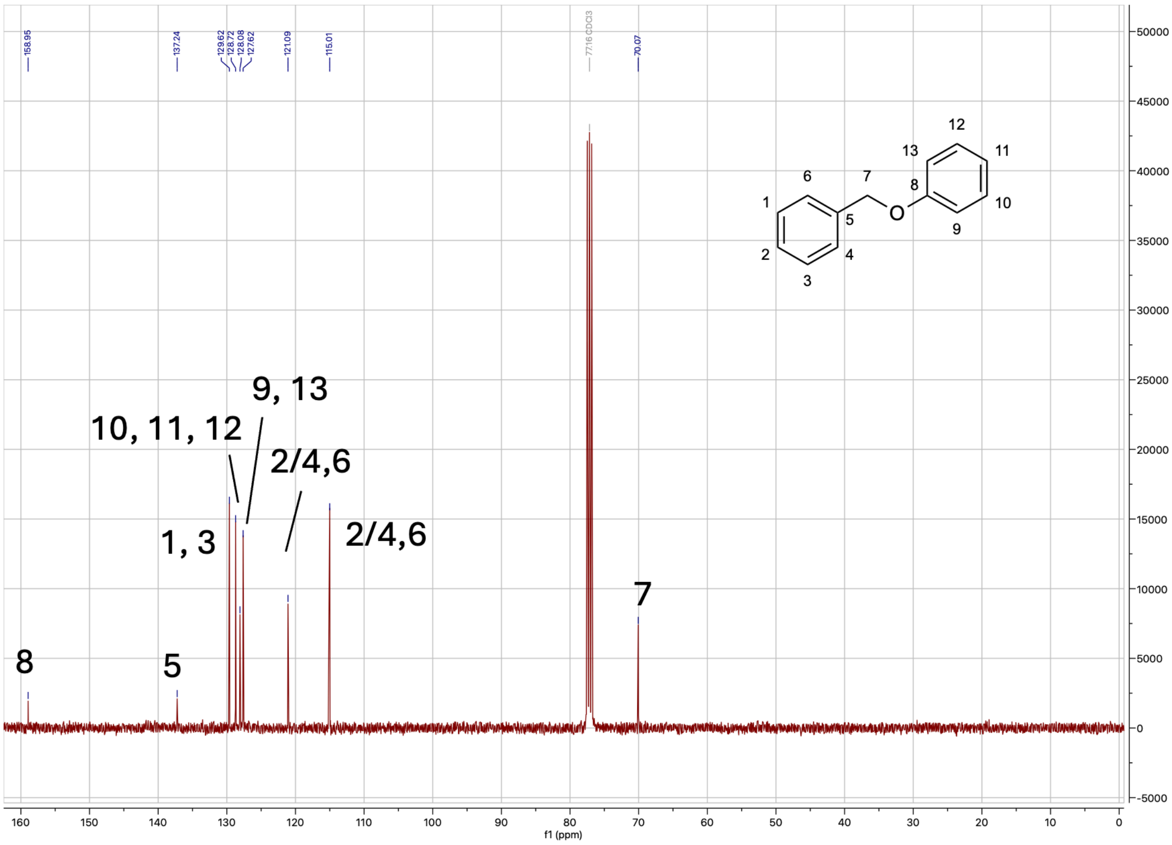

Figure S65: ^{13}C NMR spectrum (101 MHz) of **HP 5** in $CDCl_3$.

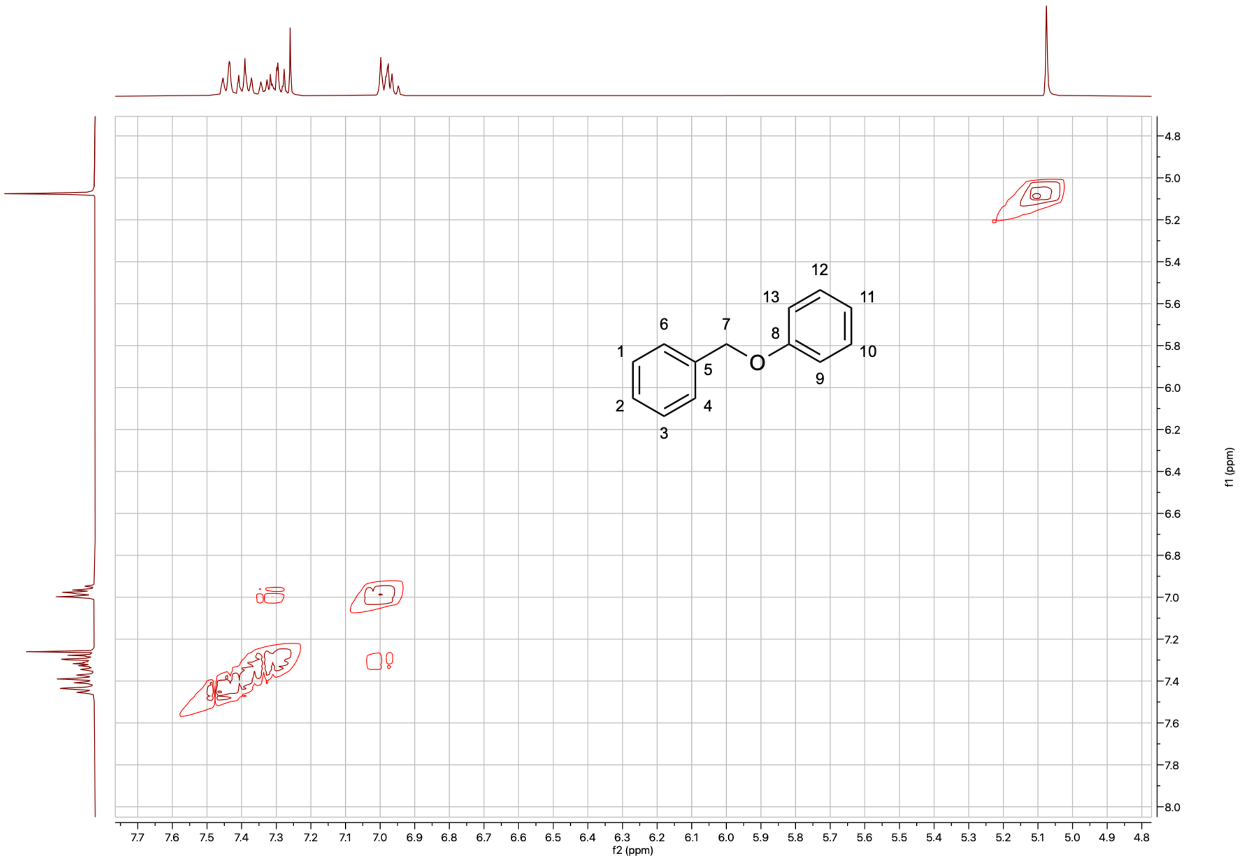

Figure S66: COSY spectrum (400 MHz) of **HP 5** in $CDCl_3$.

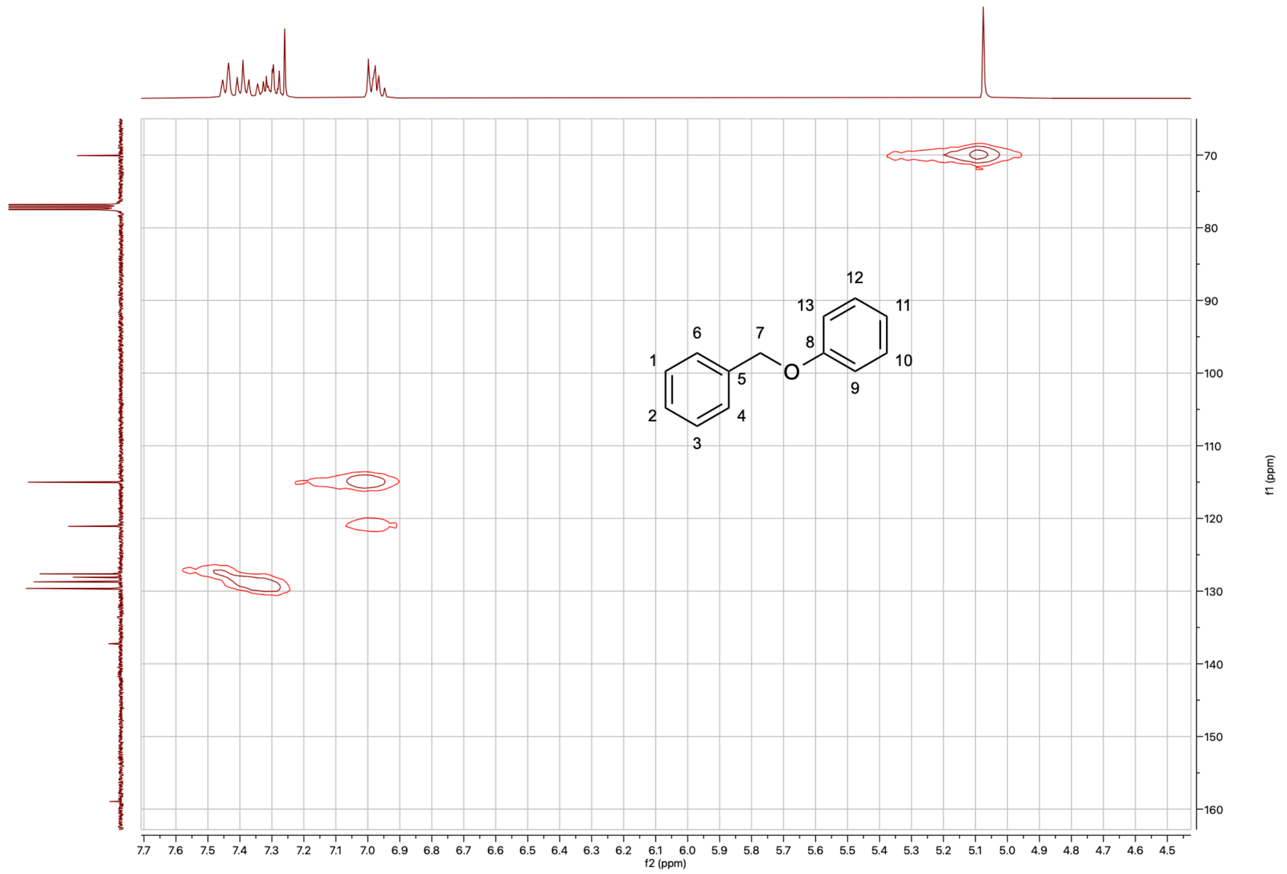

Figure S67: HMQC spectrum of **HP 5** in $CDCl_3$.

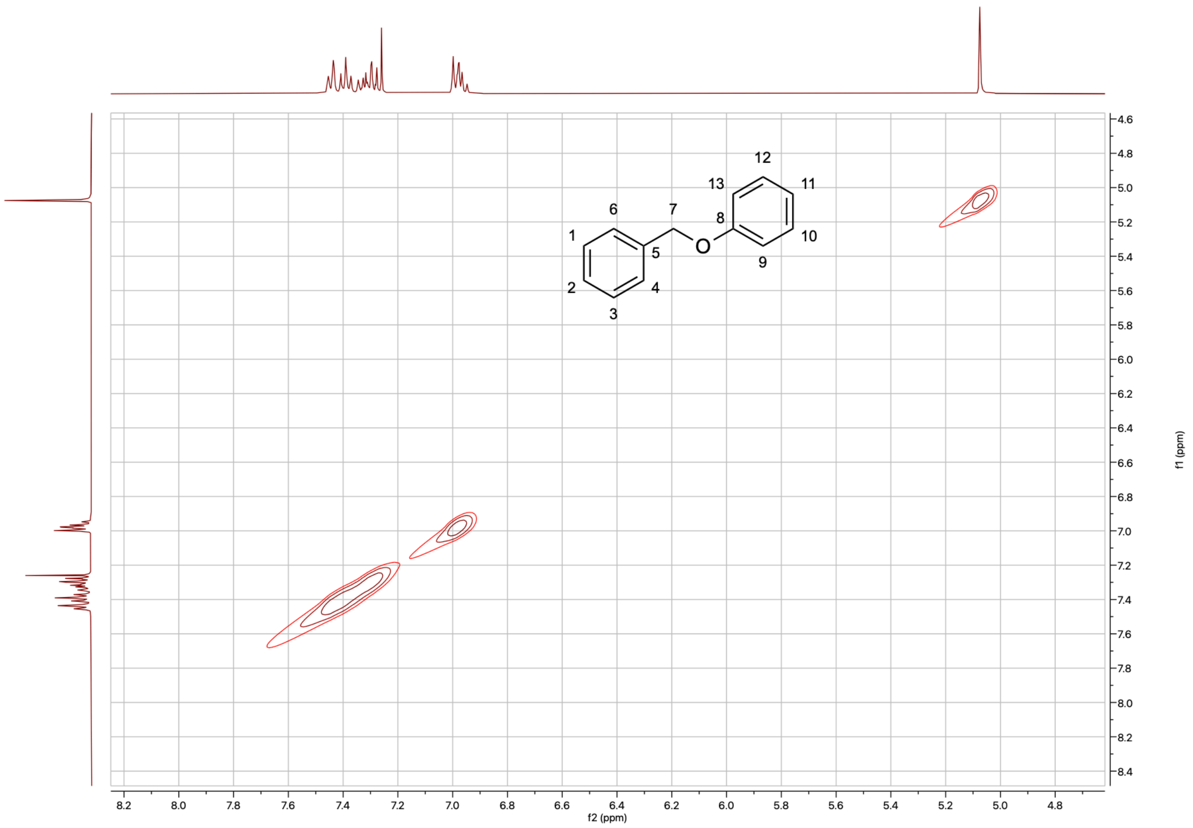

Figure S68: NOESY spectrum of **HP 5** in $CDCl_3$.

8-(tert-Butyl)-6H-benzo[c]chromene (CP 6A)

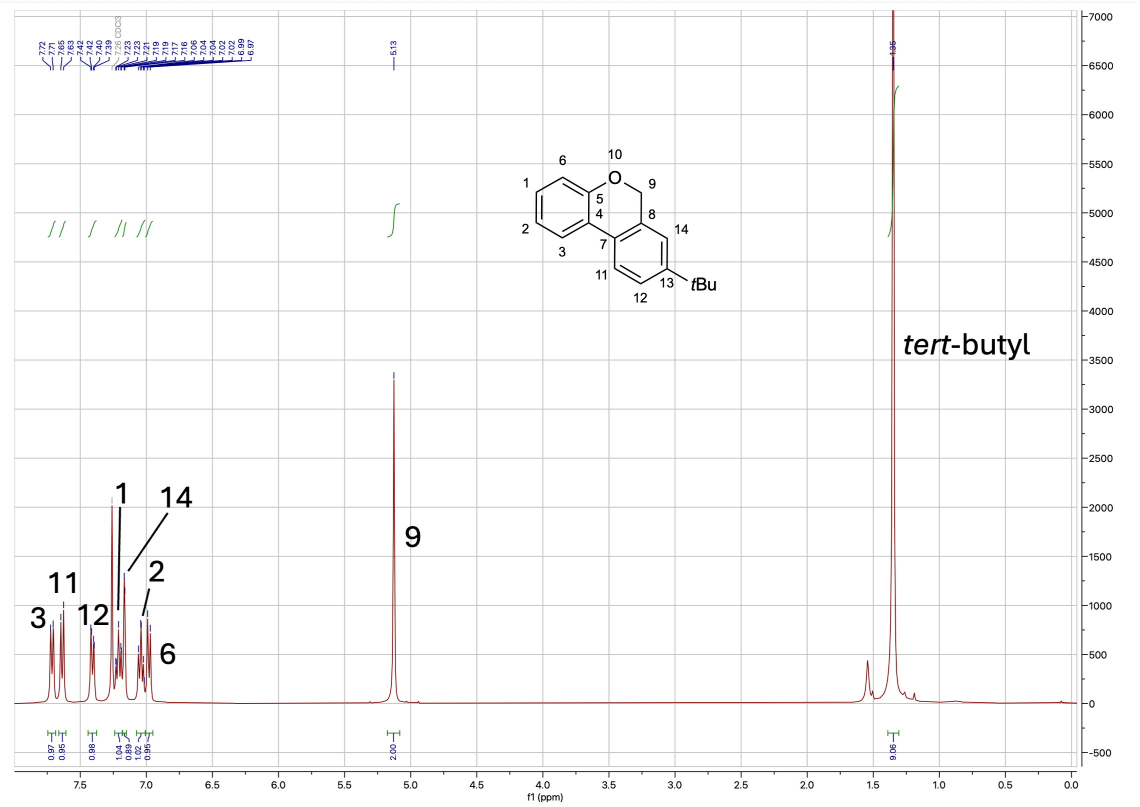

Figure S69: ^{1}H NMR spectrum (400 MHz) of **CP 6A** in $CDCl_3$.

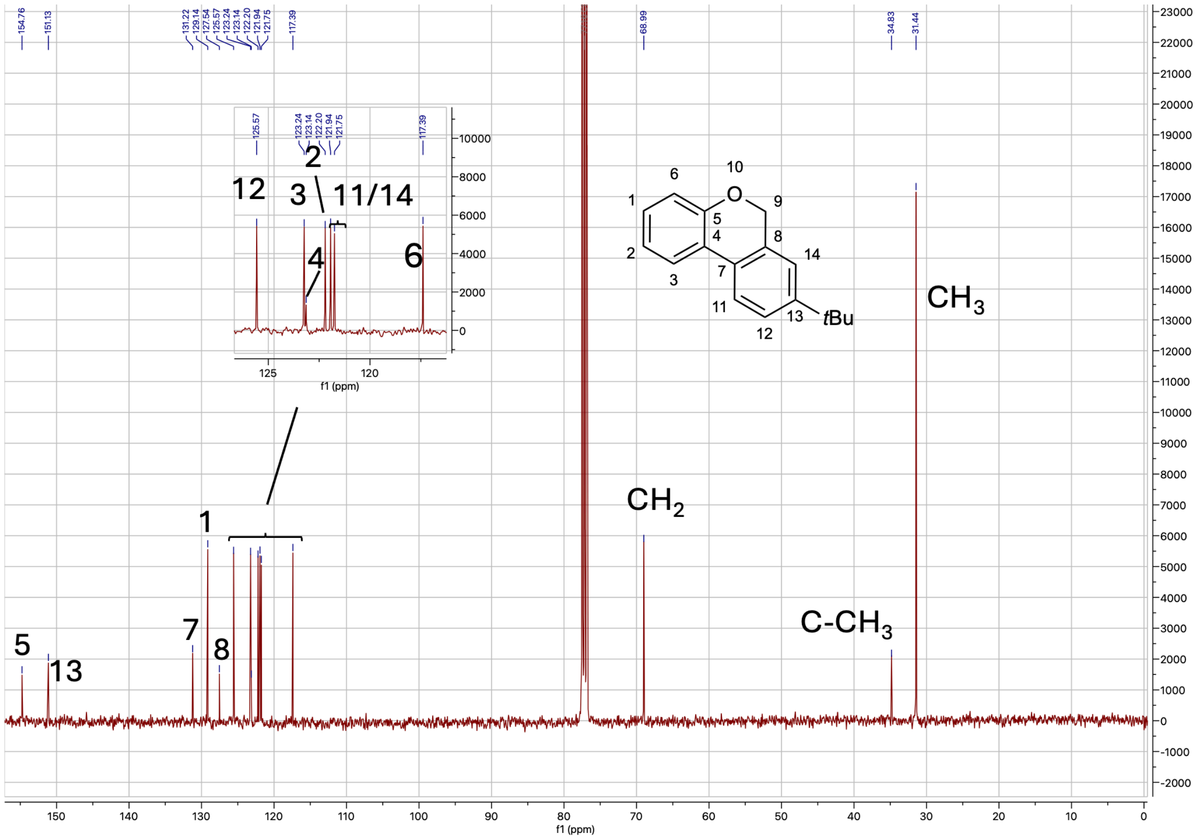

Figure S70: ^{13}C NMR spectrum (101 MHz) of **CP 6A** in $CDCl_3$.

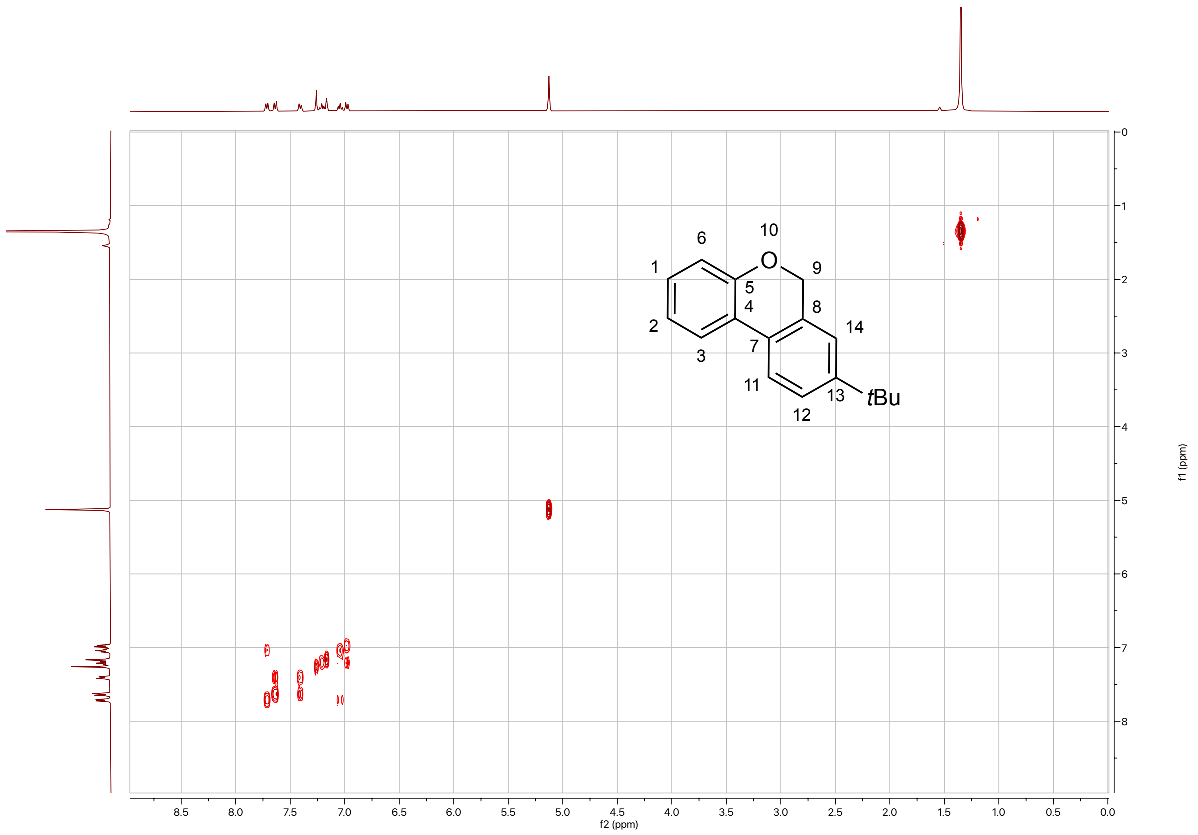

Figure S71: COSY spectrum (400 MHz) of **CP 6A** in $CDCl_3$.

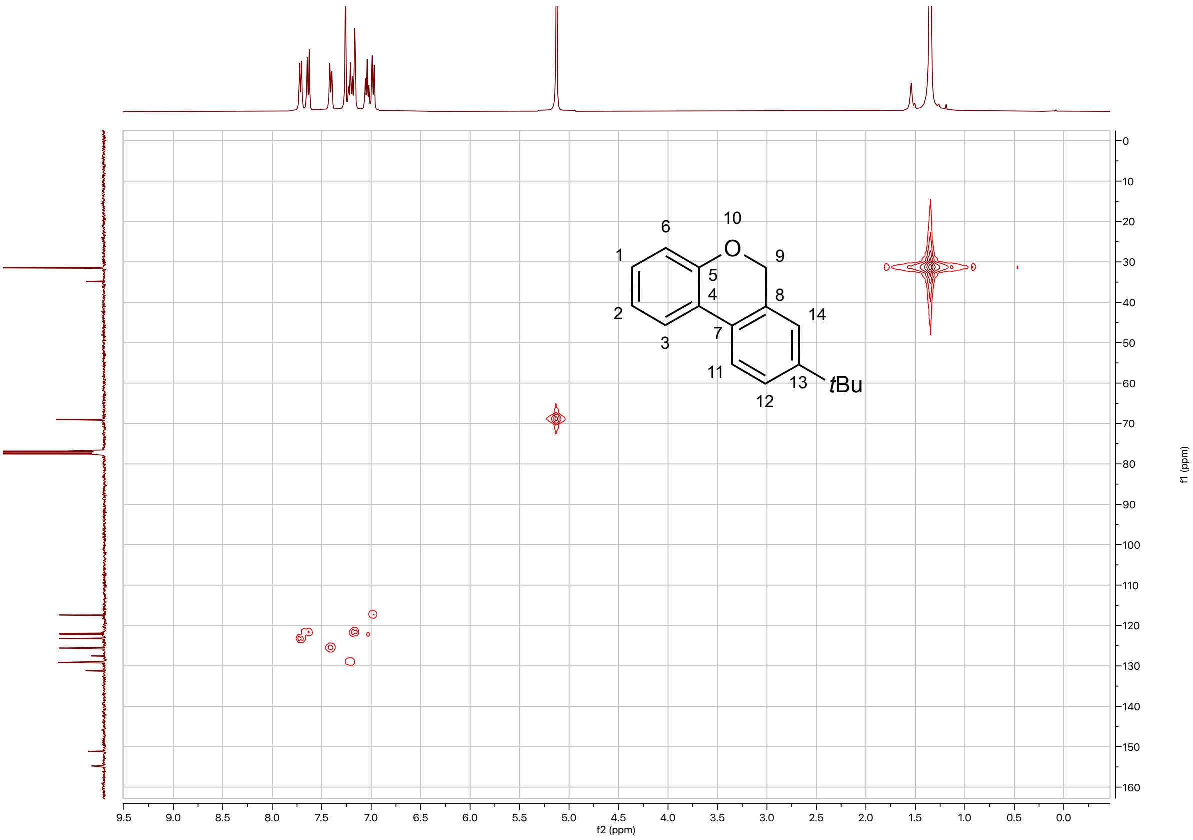

Figure S72: HMQC spectrum of **CP 6A** in $CDCl_3$.

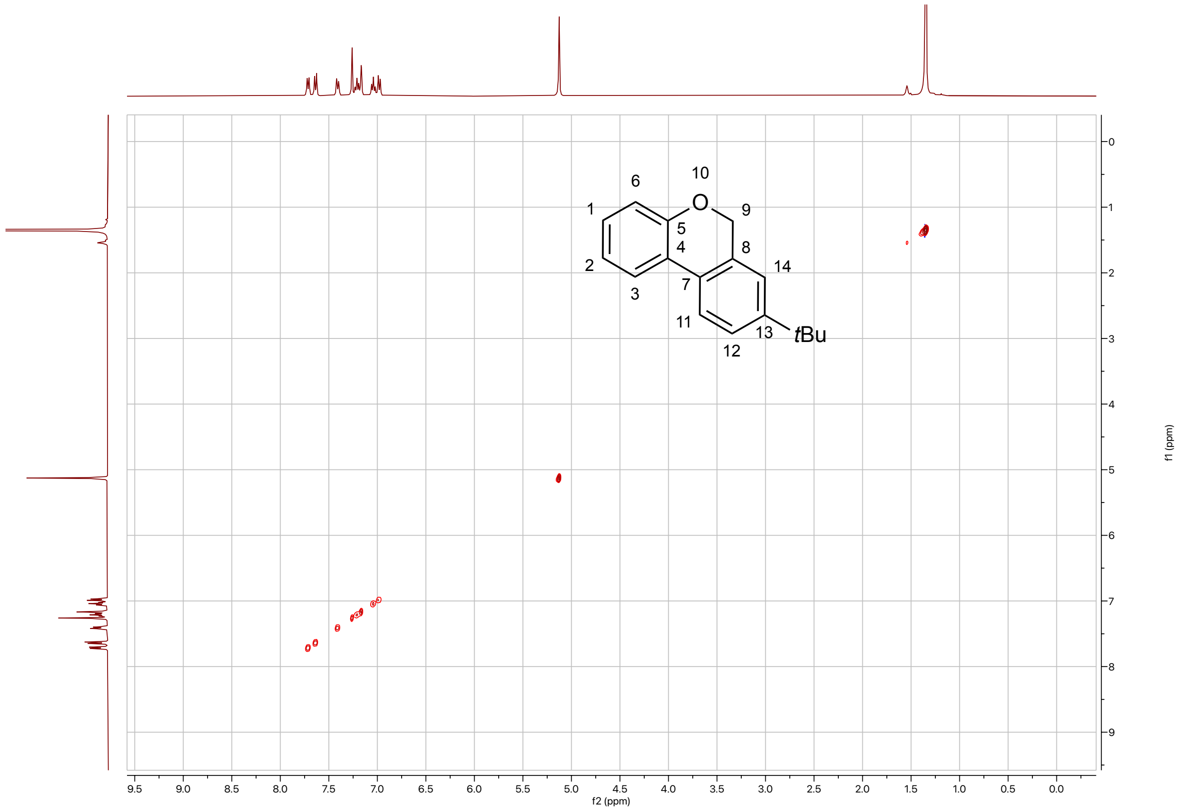

Figure S73: NOESY spectrum of **CP 6A** in $CDCl_3$.

9-(tert-Butyl)-6H-benzo[c]chromene (CP 6B)

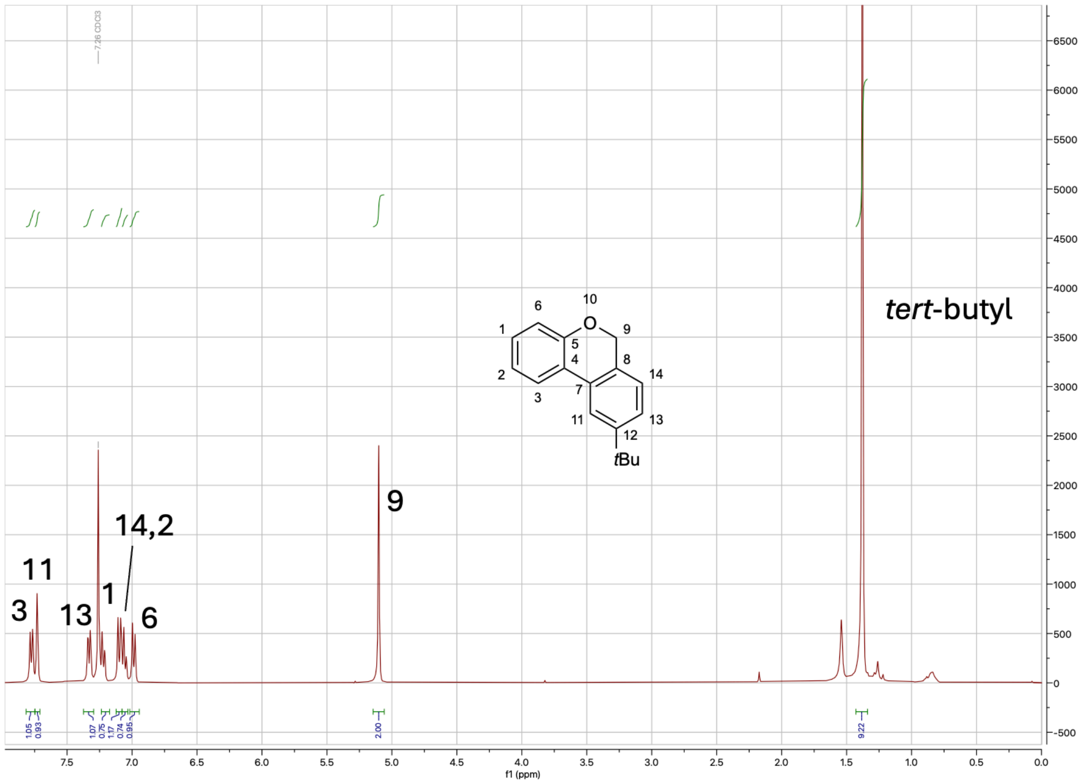

Figure S74: ^{1}H NMR spectrum (400 MHz) of **CP 6B** in $CDCl_3$

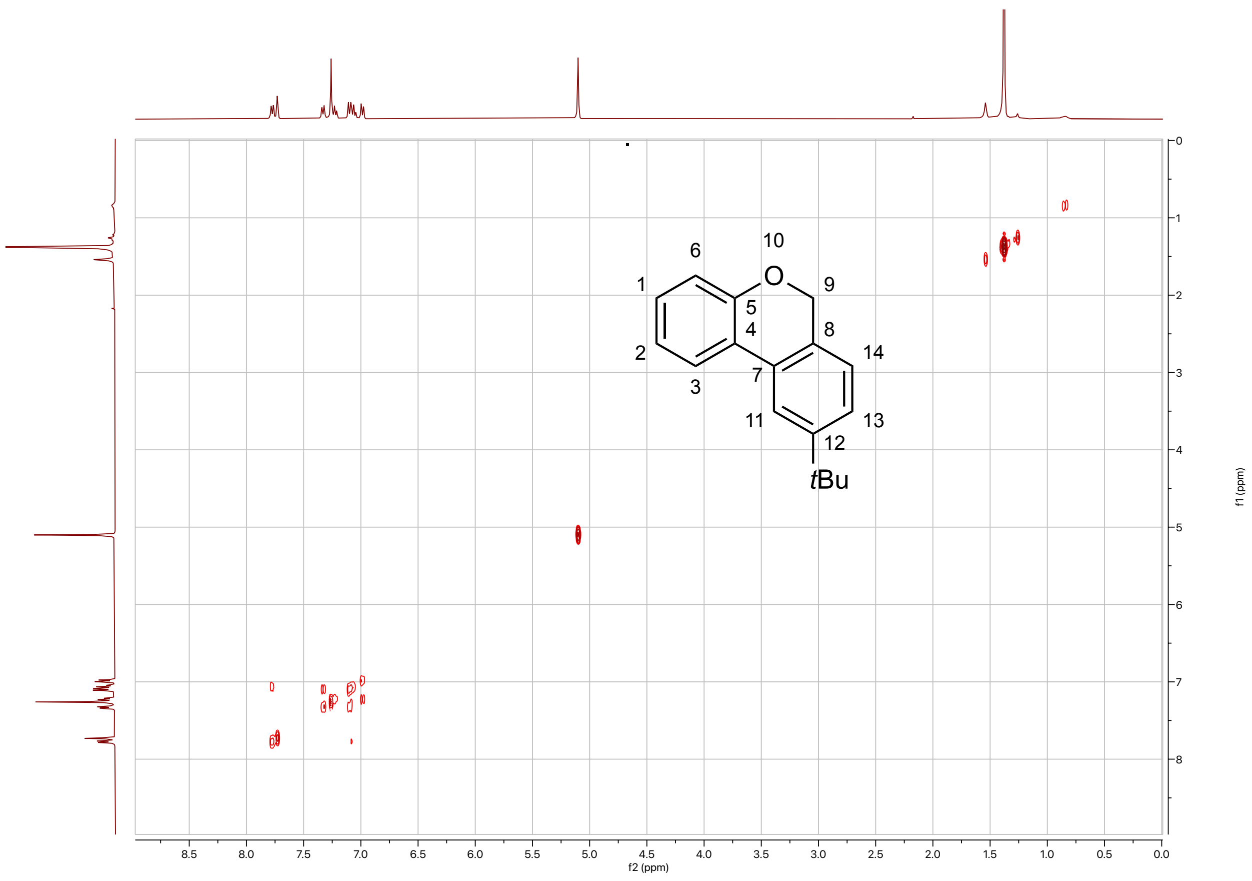

Figure S75: COSY spectrum (400 MHz) of **CP 6B** in $CDCl_3$.

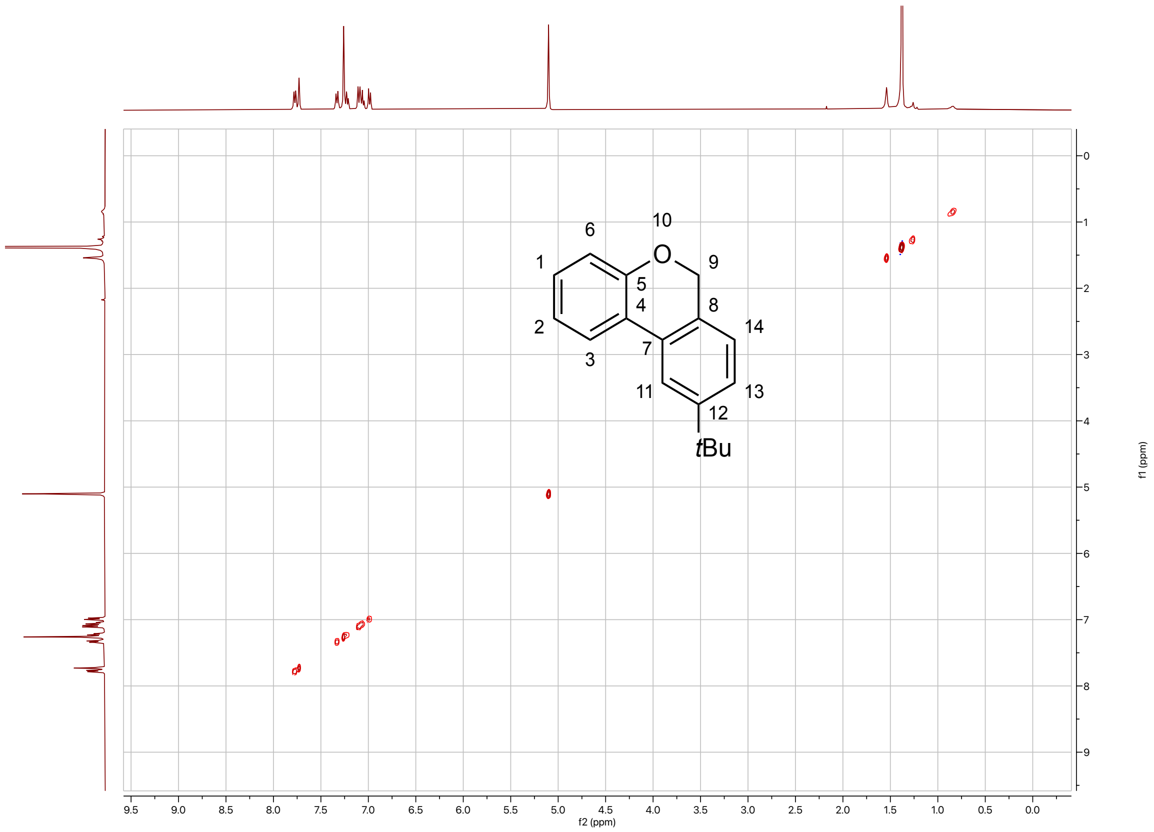

Figure S76: NOESY spectrum of **CP 6B** in $CDCl_3$.

6H-benzo[c]chromene-2-carbaldehyde (CP 7)

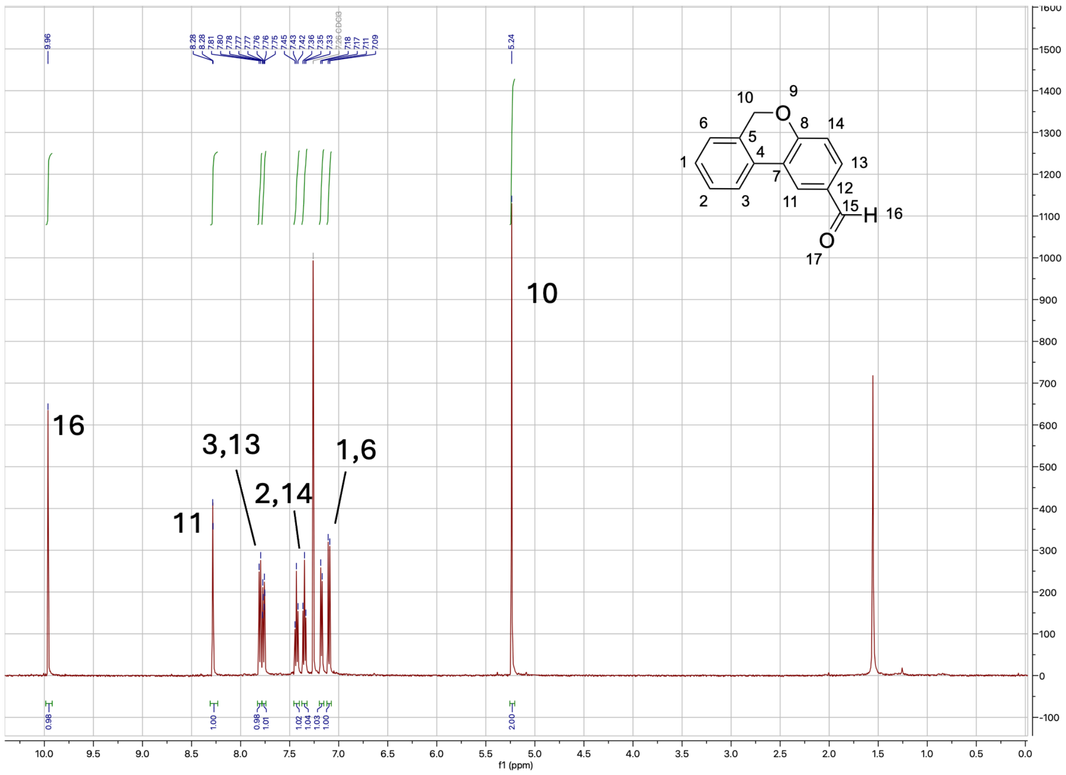

Figure S74: ^{1}H NMR spectrum (500 MHz) of **CP 7** in $CDCl_3$.

4-(Benzyloxy)benzaldehyde (HP 7)

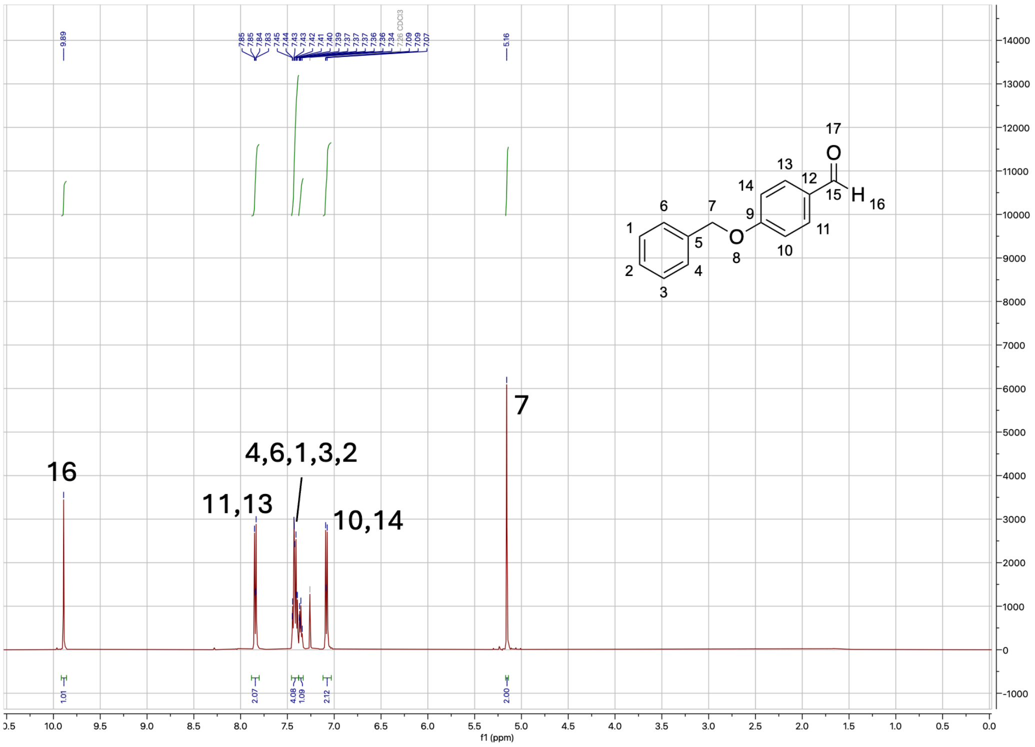

Figure S77: ^{1}H NMR spectrum (500 MHz) of **HP 7** in $CDCl_3$.

Methyl 6H-benzo[c]chromene-2-carboxylate (CP 8)

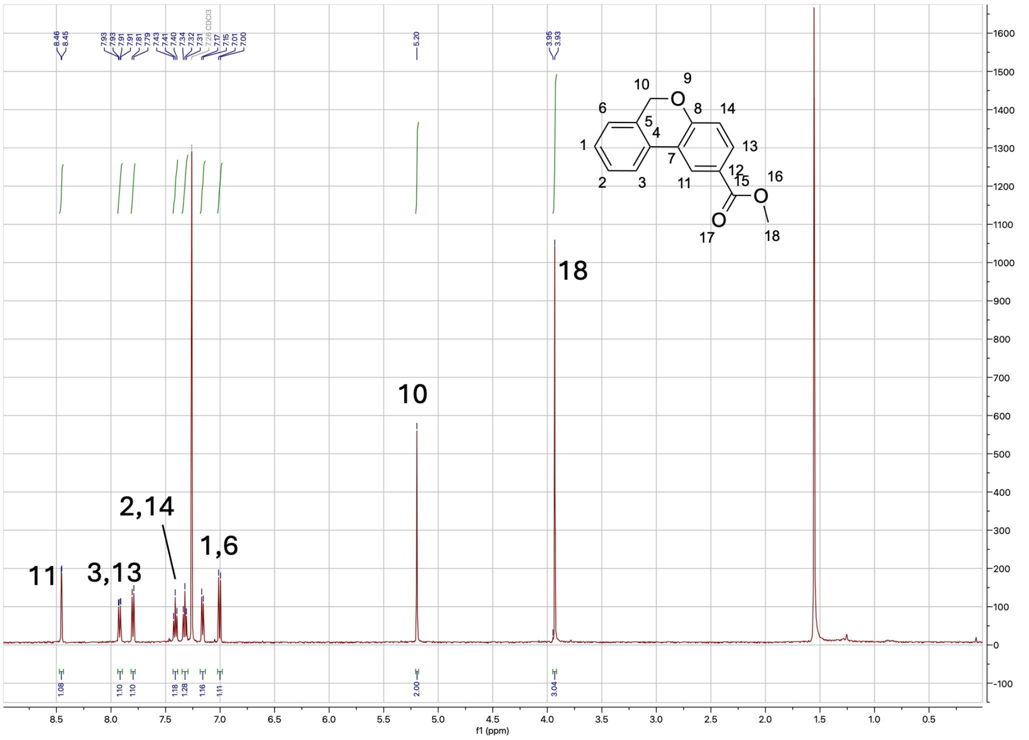

Figure S78: ^{1}H NMR spectrum (500 MHz) of **CP 8** in $CDCl_3$.

2-(Trifluoromethyl)-6H-benzo[c]chromene (CP 9)

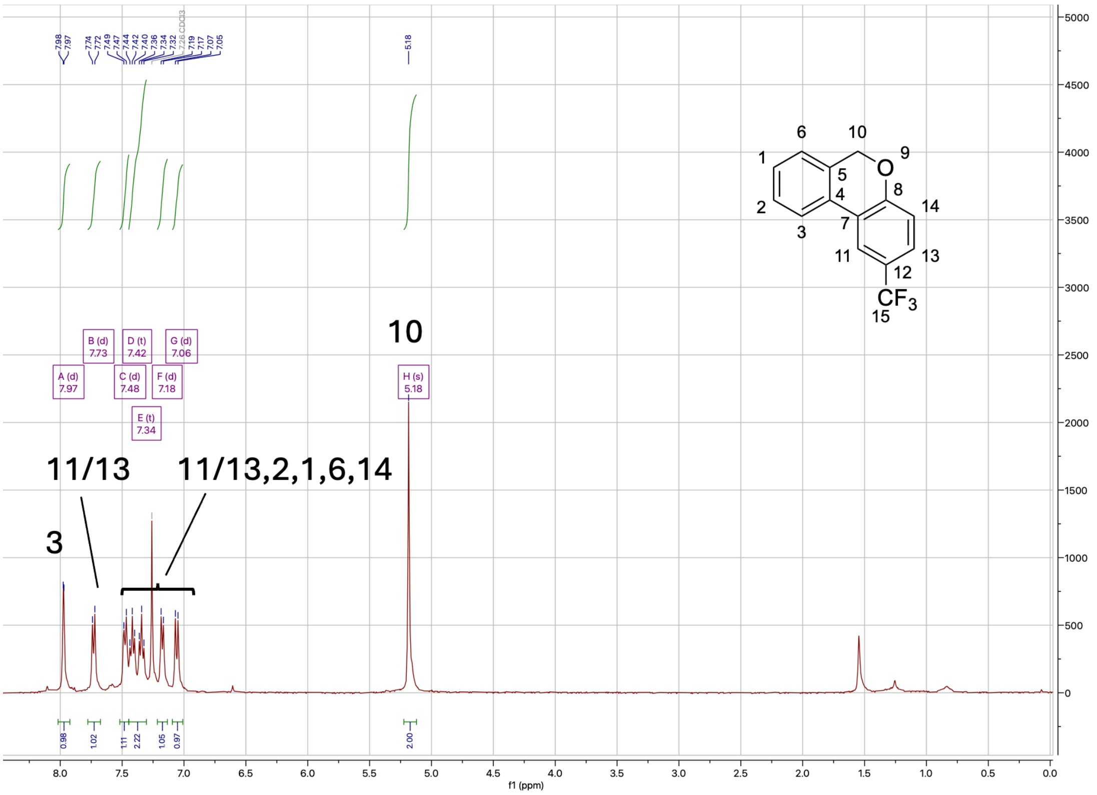

Figure S79: ^{1}H NMR spectrum (400 MHz) of **CP 9** in $CDCl_3$.

1-(Benzyloxy)-4-(trifluoromethyl)benzene (HP 9)

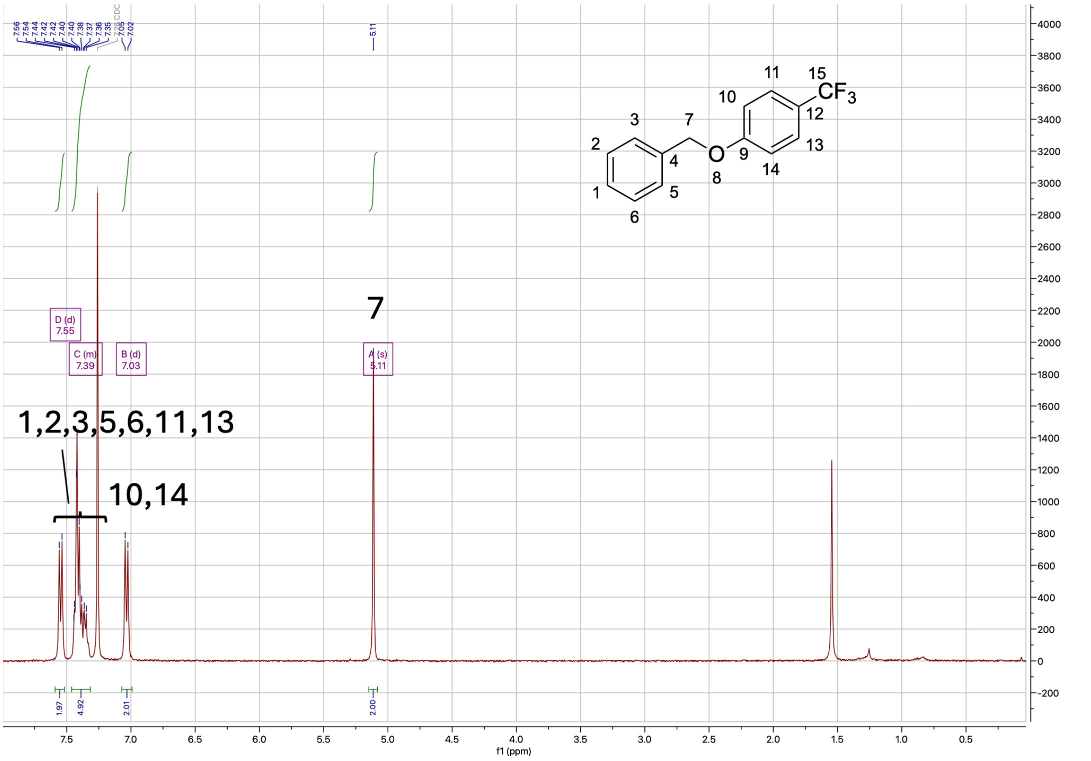

Figure S80: ^{1}H NMR spectrum (400 MHz) of **HP 9** in $CDCl_3$.

2-(tert-Butyl)-6H-naphtho[2,1-c]chromene (CP 11)

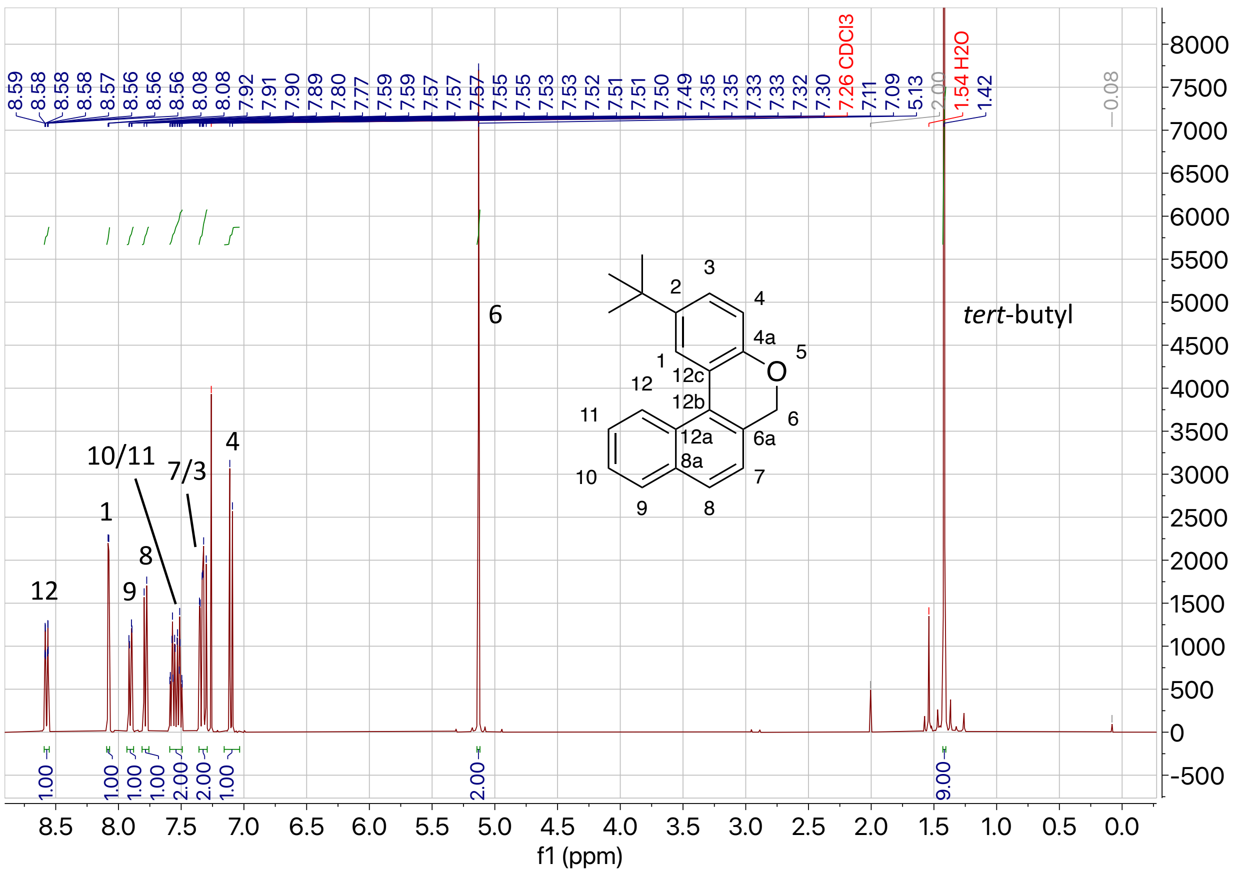

Figure S81: ^{1}H NMR spectrum (400 MHz) of **CP 11** in $CDCl_3$.

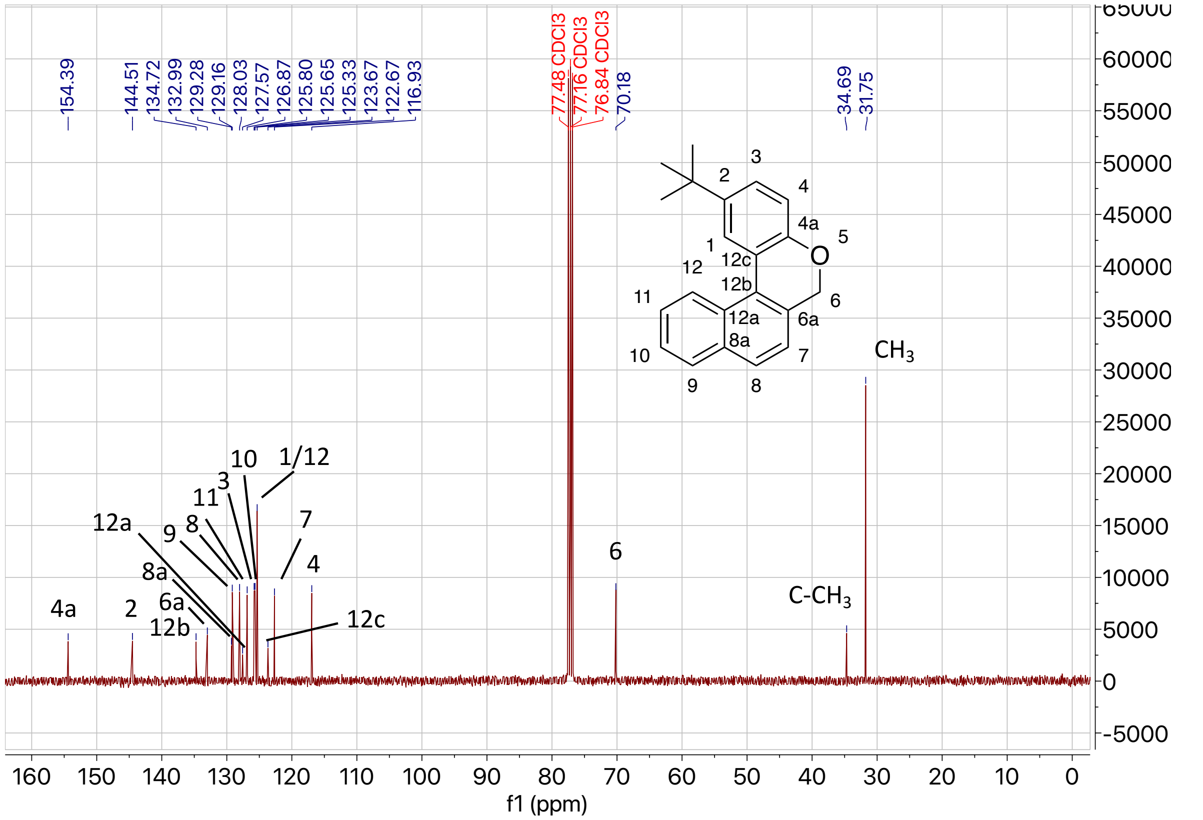

Figure S82: ^{13}C NMR spectrum (101 MHz) of **CP 11** in $CDCl_3$.

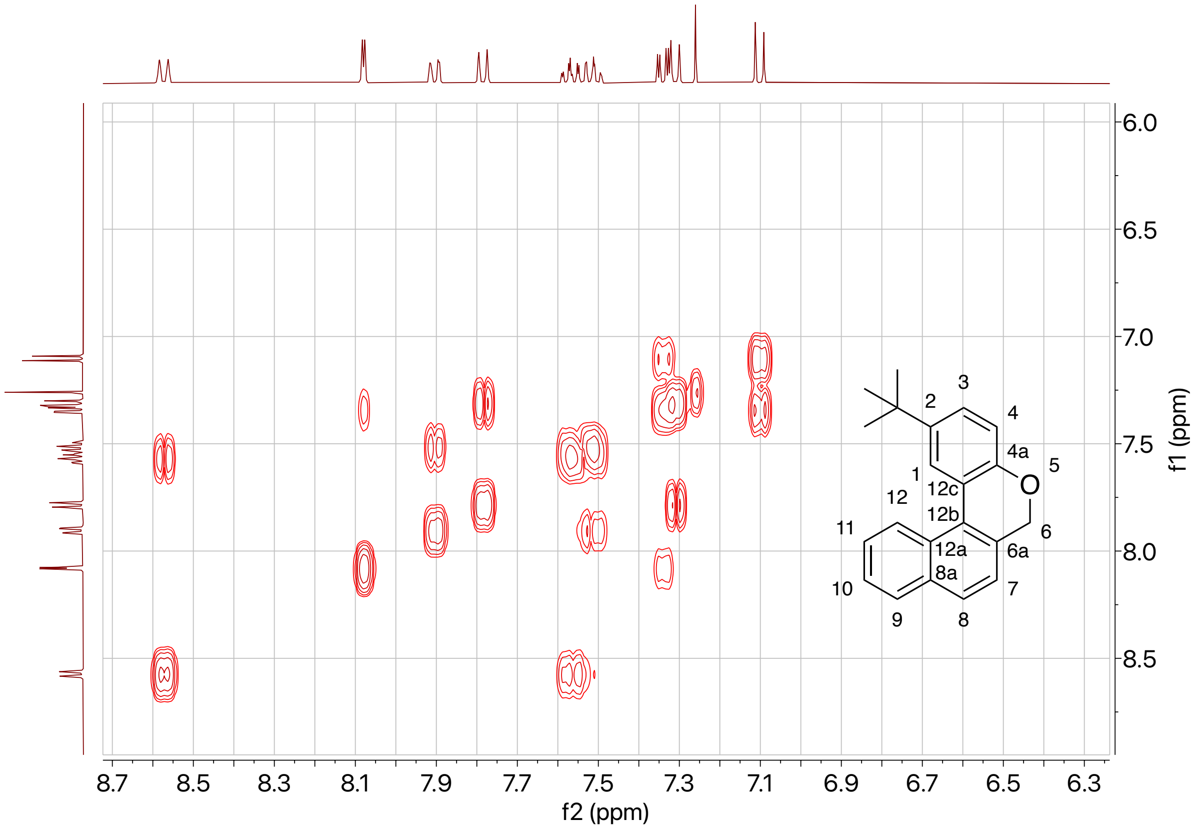

Figure S83: COSY spectrum (400 MHz) of **CP 11** in $CDCl_3$.

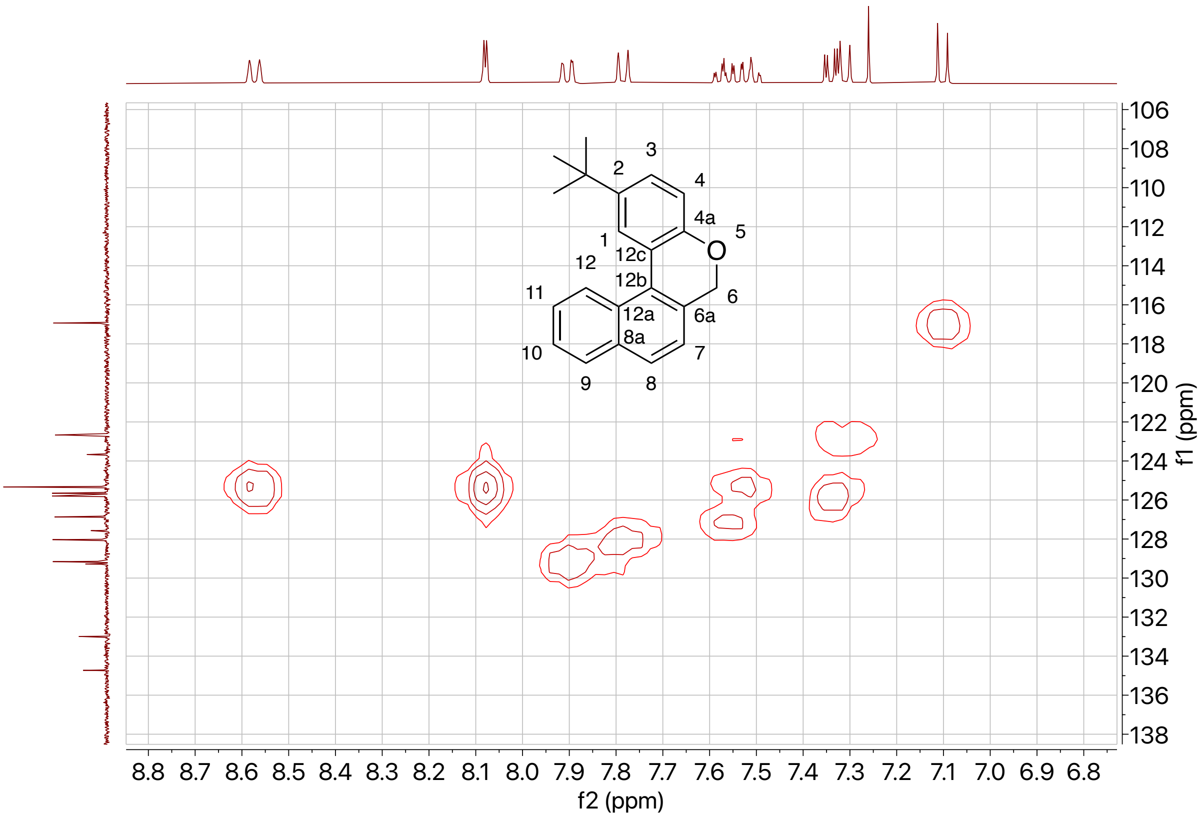

Figure S84: HMQC spectrum of **CP 11** in $CDCl_3$.

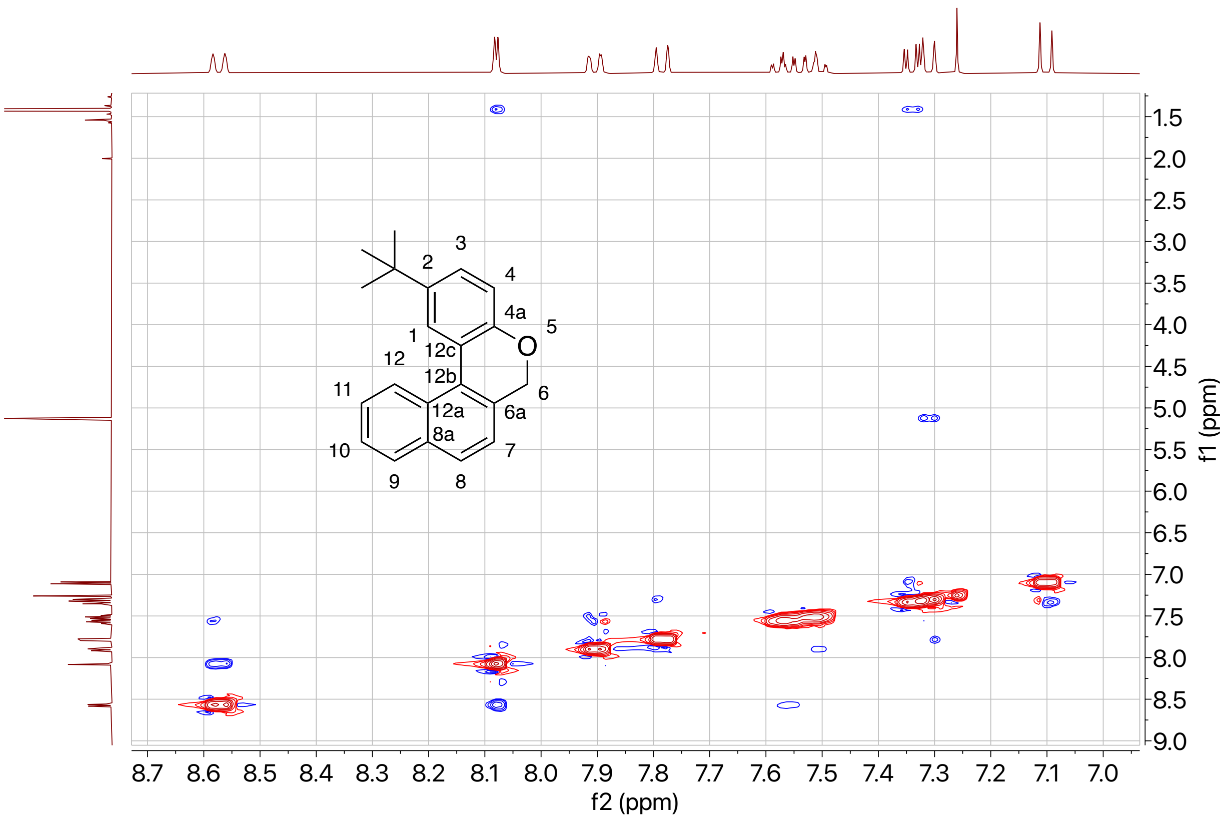

Figure S85: NOESY spectrum of **CP 11** in $CDCl_3$.

2-((4-(tert-Butyl)phenoxy)methyl)naphthalene (HP 11)

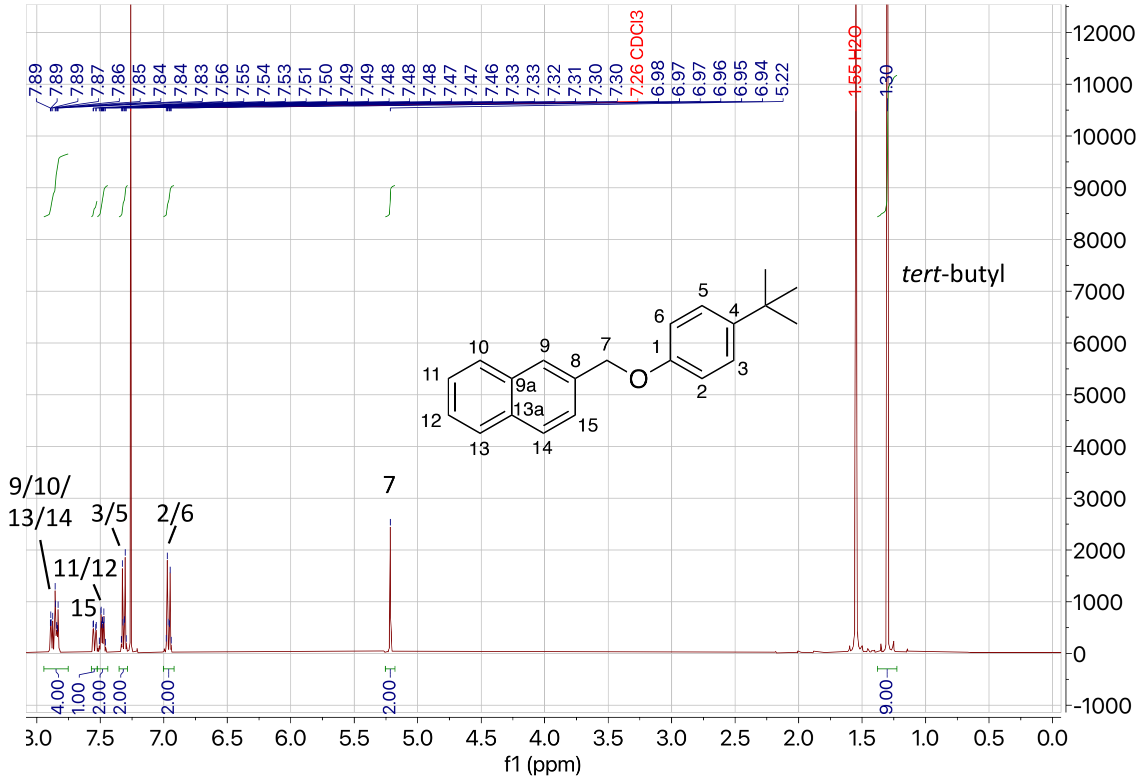

Figure S86: ^{1}H NMR spectrum (400 MHz) of **HP 11** in $CDCl_3$.

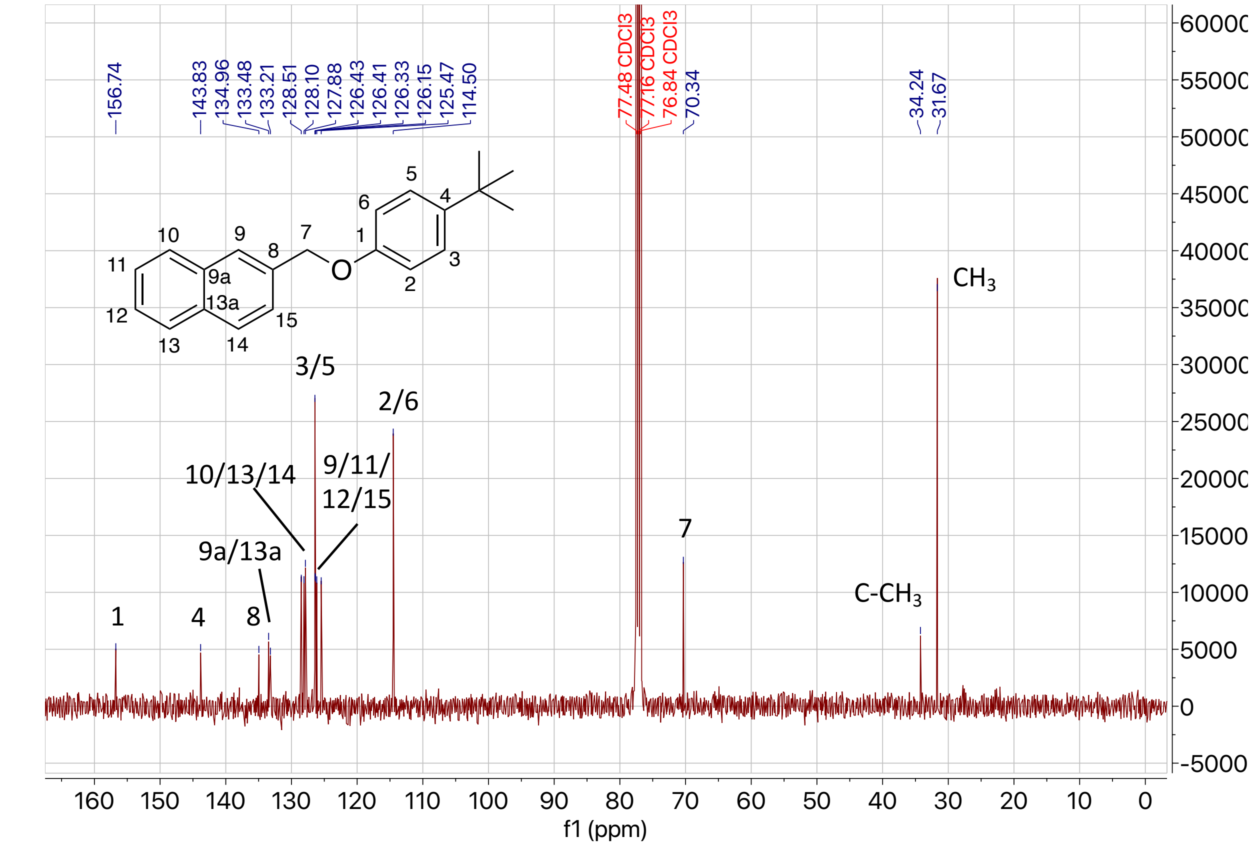

Figure S87: ^{13}C NMR spectrum (101 MHz) of **HP 11** in $CDCl_3$.

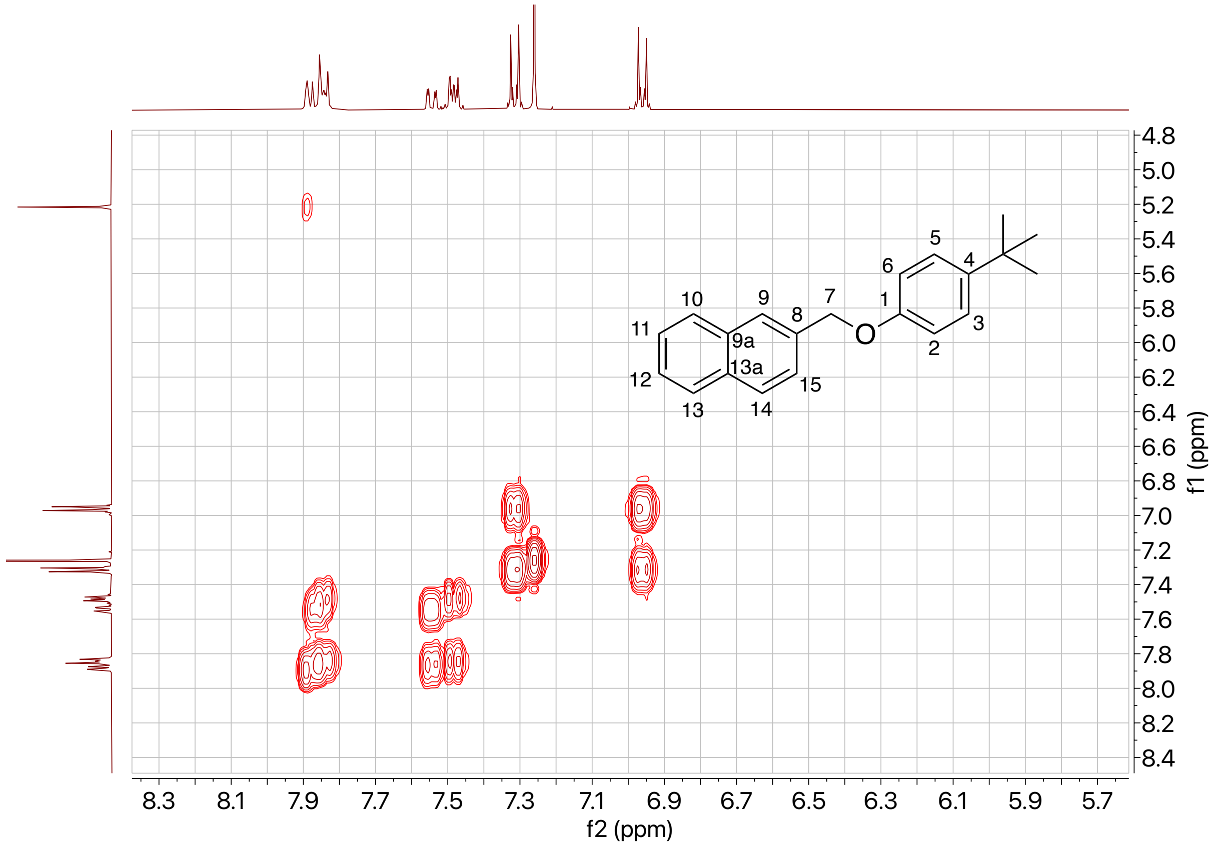

Figure S88: COSY spectrum (400 MHz) of **HP 11** in $CDCl_3$.

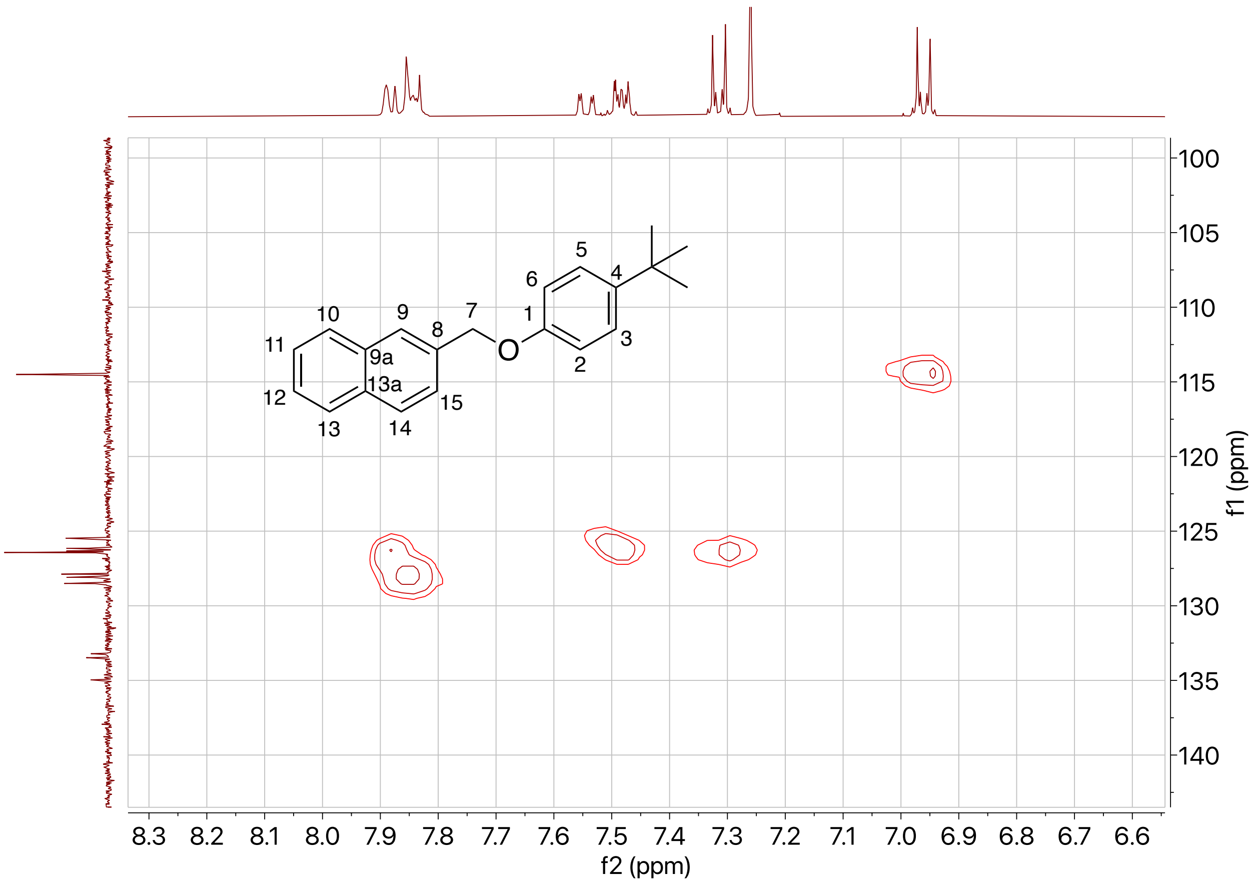

Figure S89: HMQC spectrum of **HP 11** in $CDCl_3$.

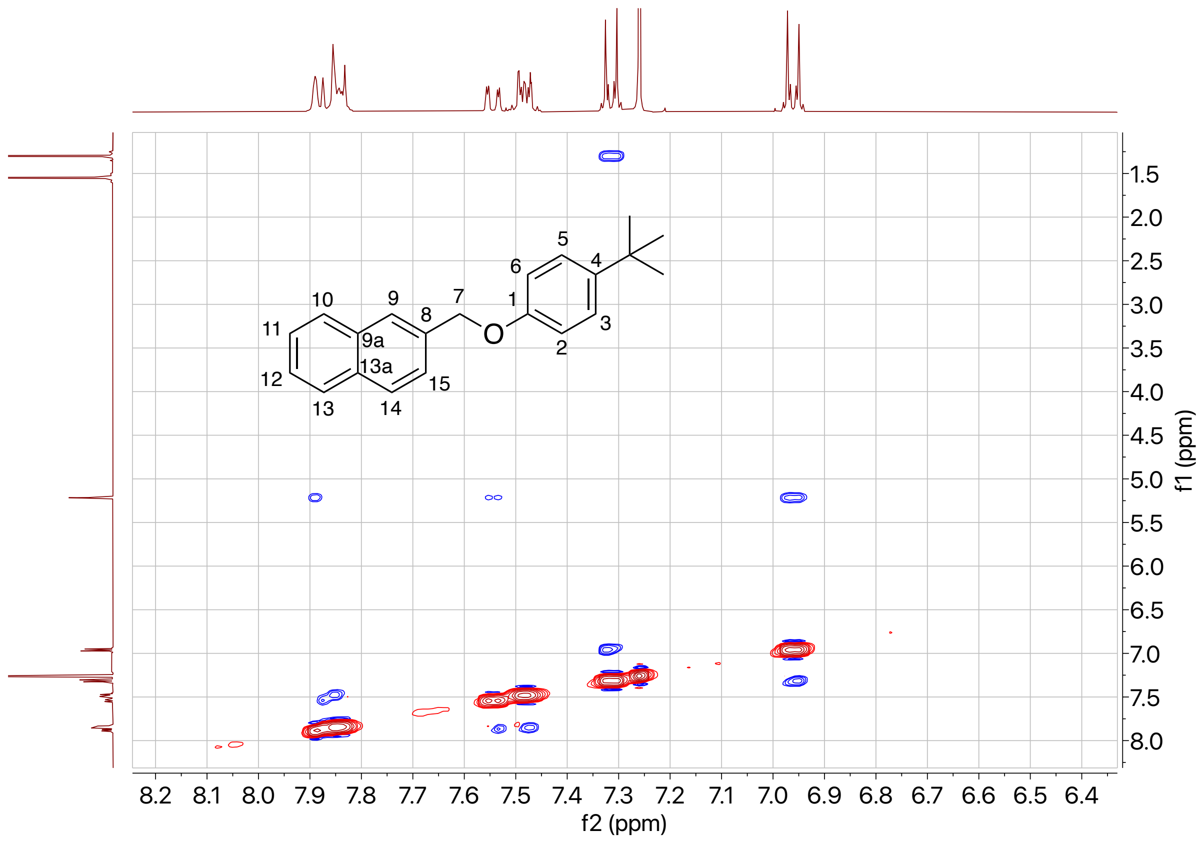

Figure S90: NOESY spectrum of **HP 11** in $CDCl_3$.

2-(tert-Butyl)-8-(trifluoromethyl)-6H-benzo[c]chromene (CP 12)

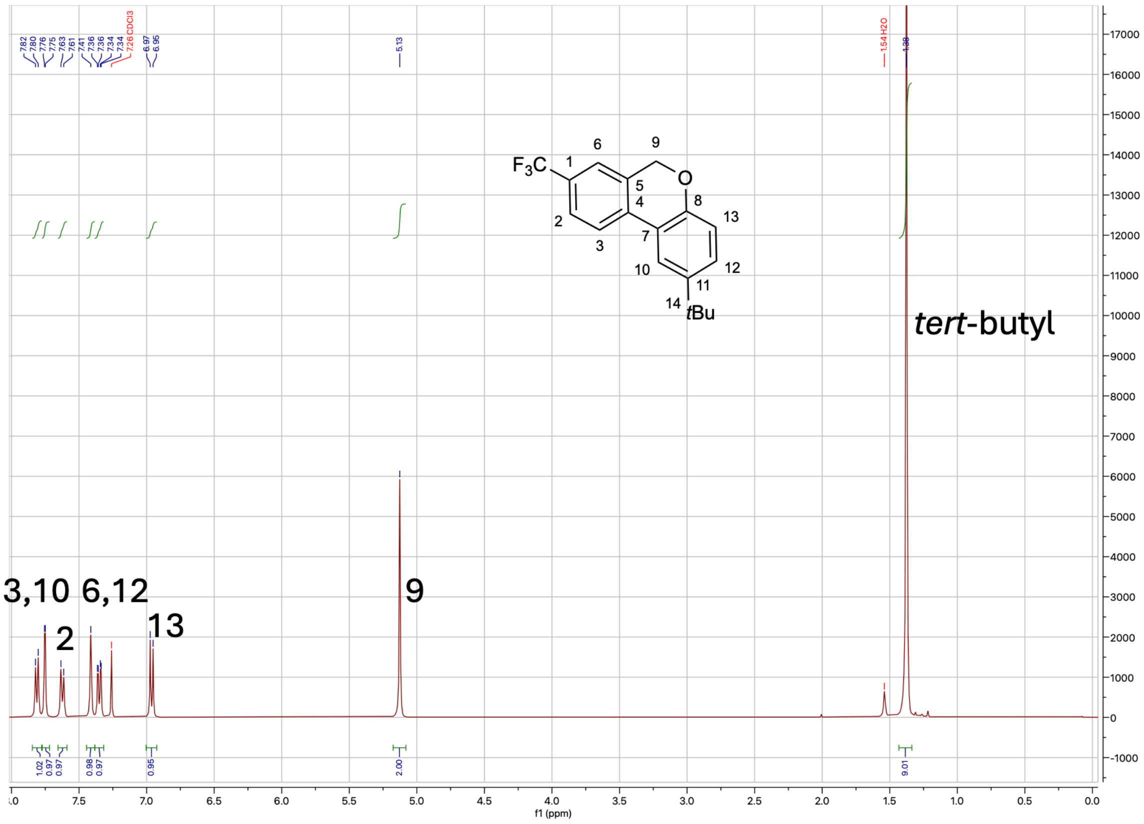

Figure S91: ^{1}H NMR spectrum (400 MHz) of **CP 12** in $CDCl_3$.

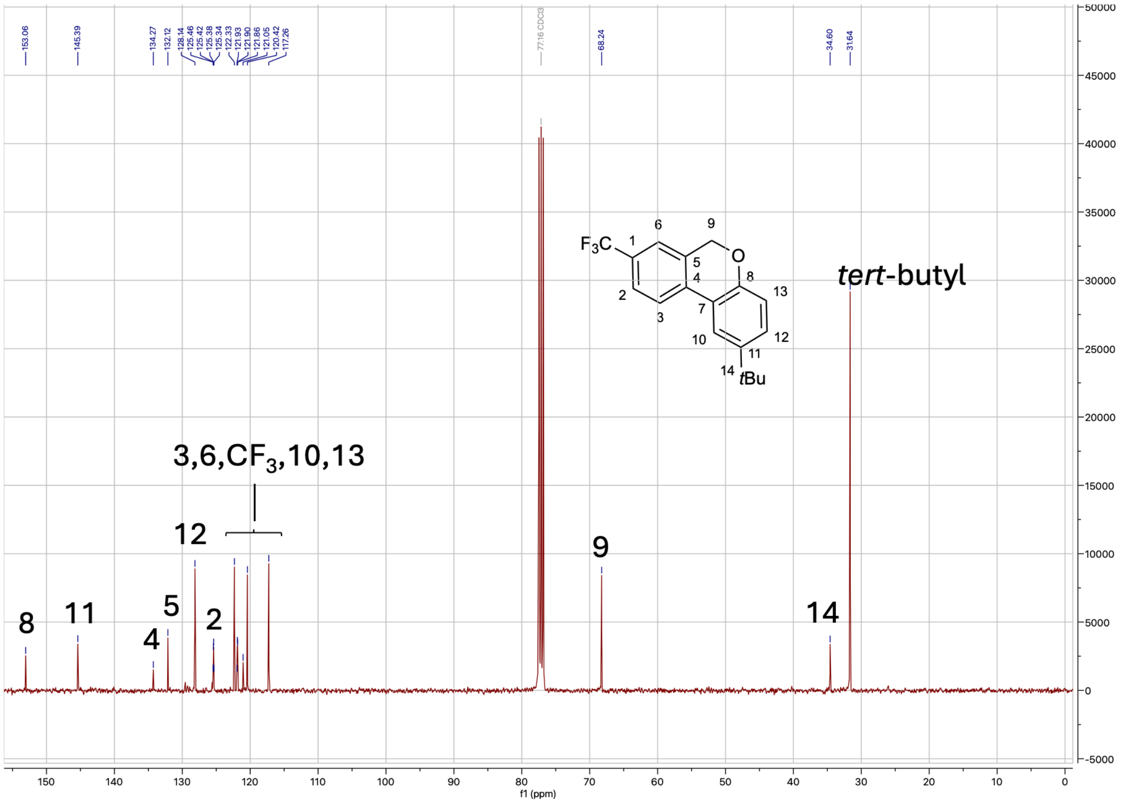

Figure S92: ^{13}C NMR spectrum (101 MHz) of **CP 12** in $CDCl_3$.

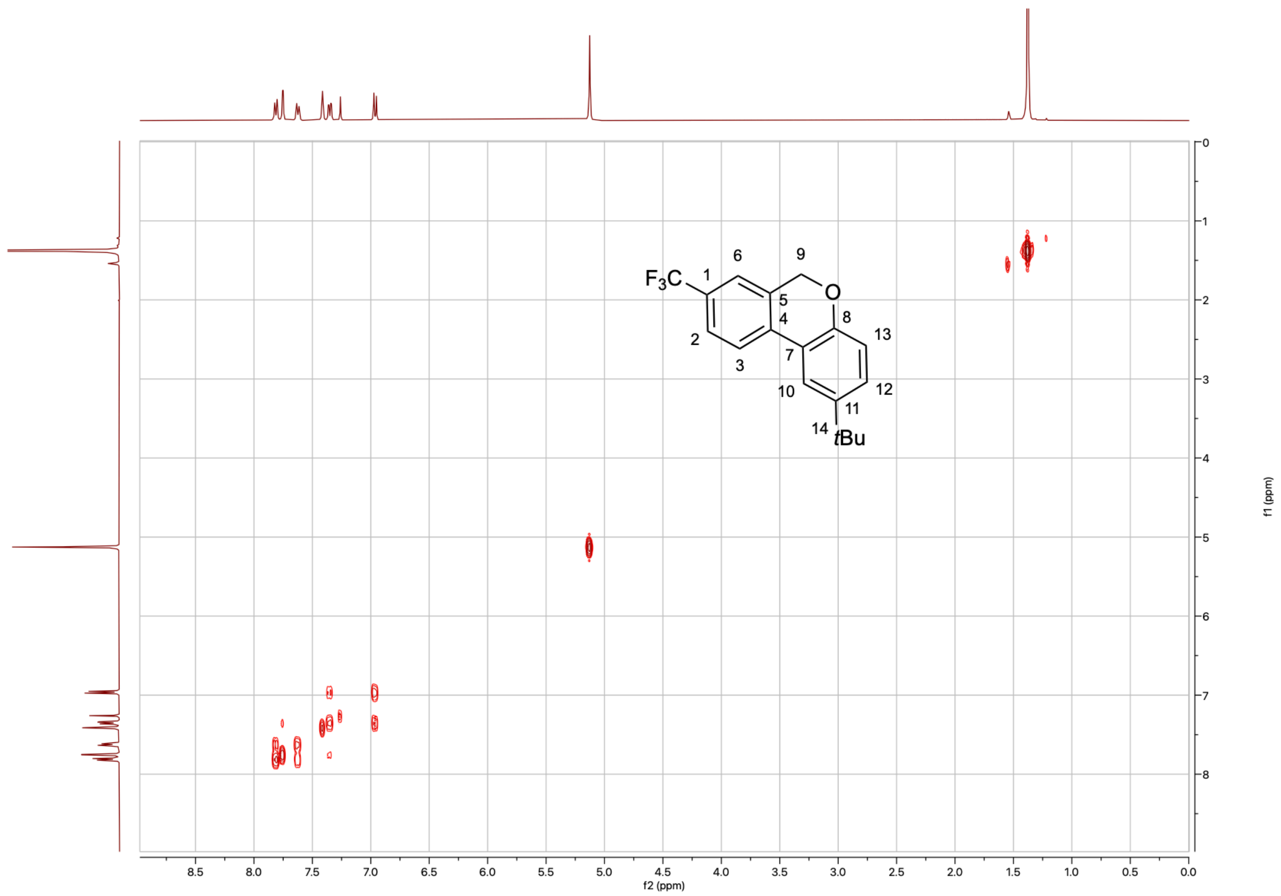

Figure S93: COSY spectrum (400 MHz) of **CP 12** in $CDCl_3$.

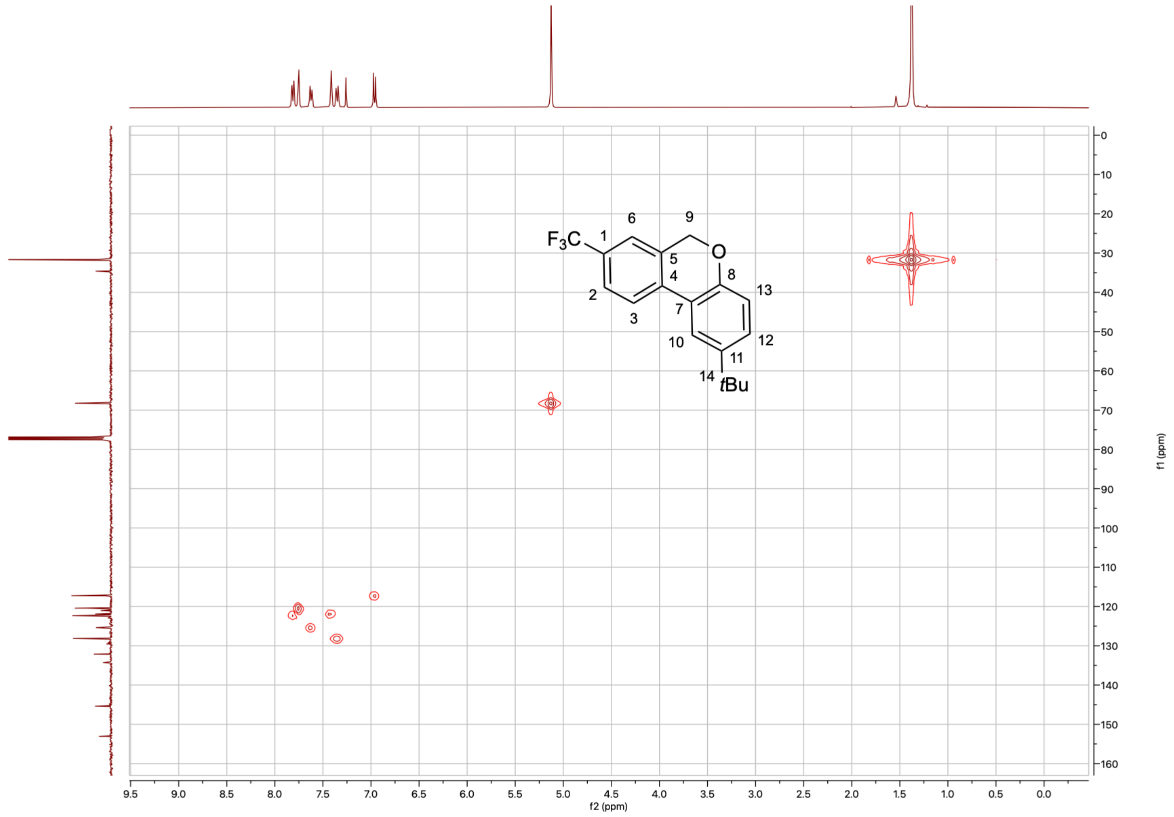

Figure S94: HMQC spectrum of **CP 12** in $CDCl_3$.

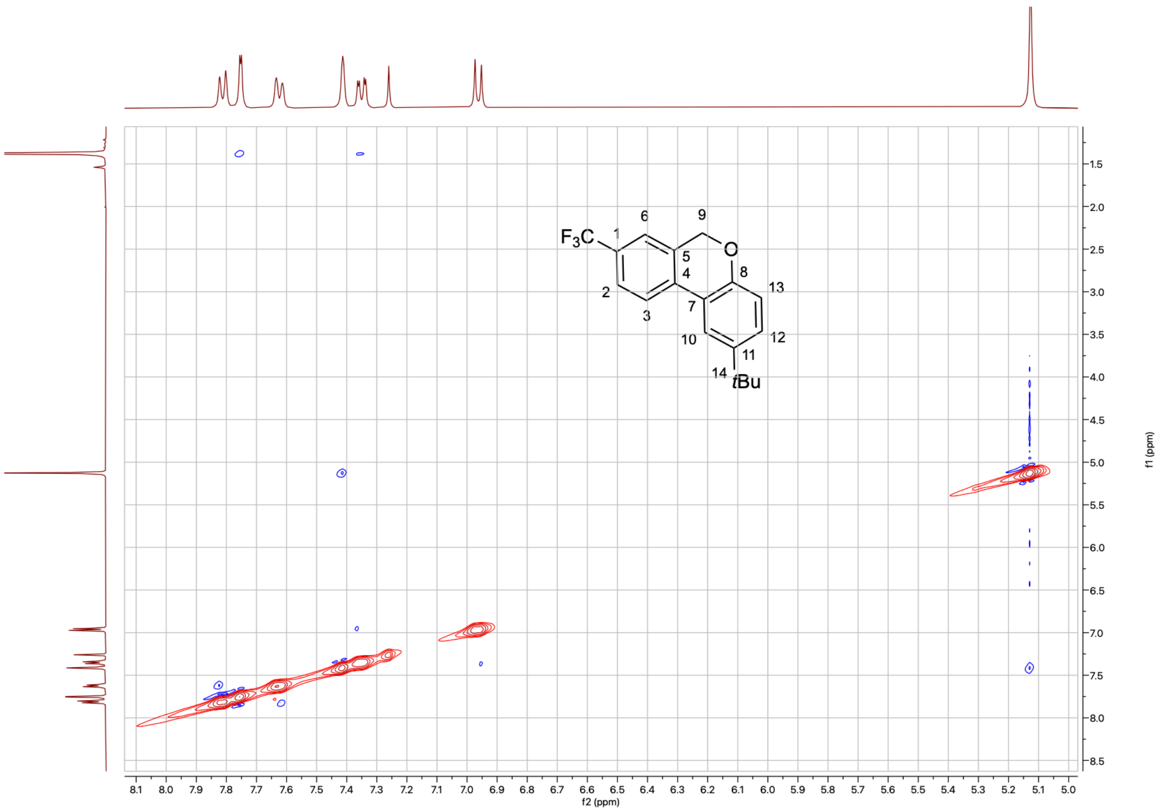

Figure S95: NOESY spectrum of **CP 12** in $CDCl_3$.

1-((4-(tert-Butyl)phenoxy)methyl)-3 (trifluoromethyl)benzene (HP 12)

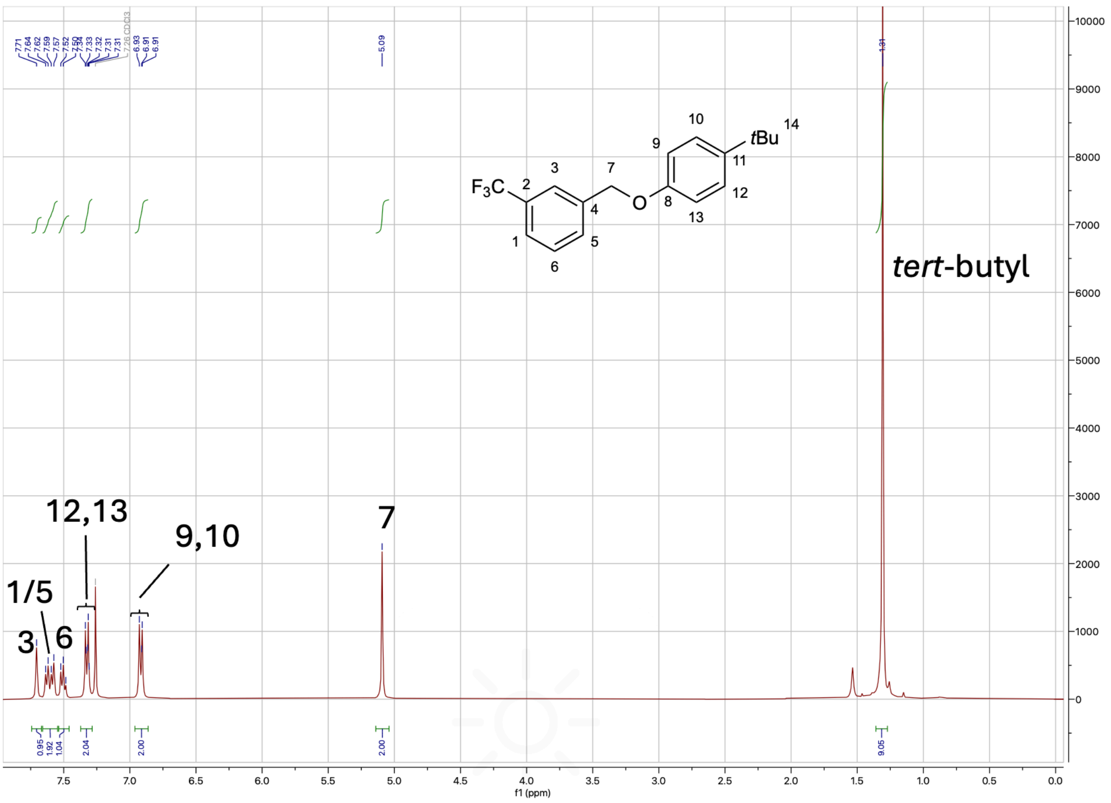

Figure S96: ^{1}H NMR spectrum (400 MHz) of **HP 12** in $CDCl_3$.

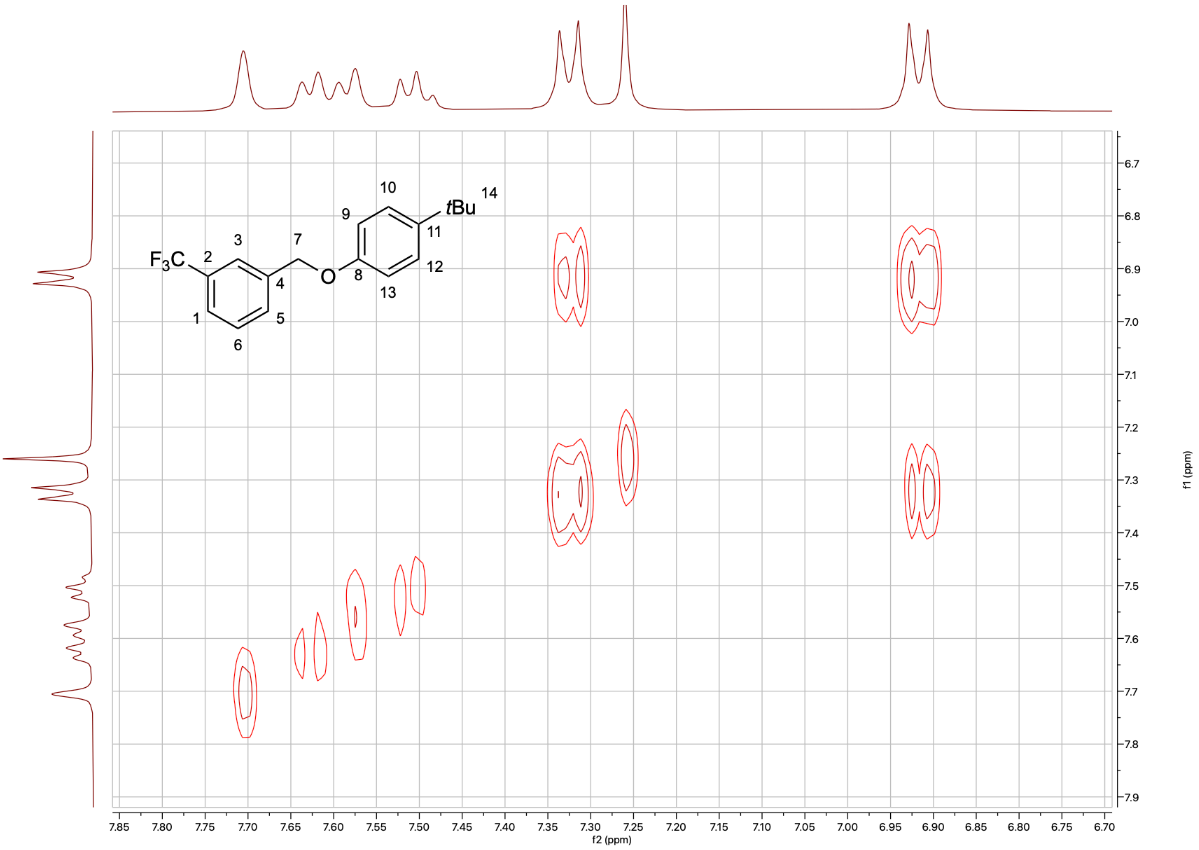

Figure S97: COSY spectrum (400 MHz) of **HP 12** in $CDCl_3$.

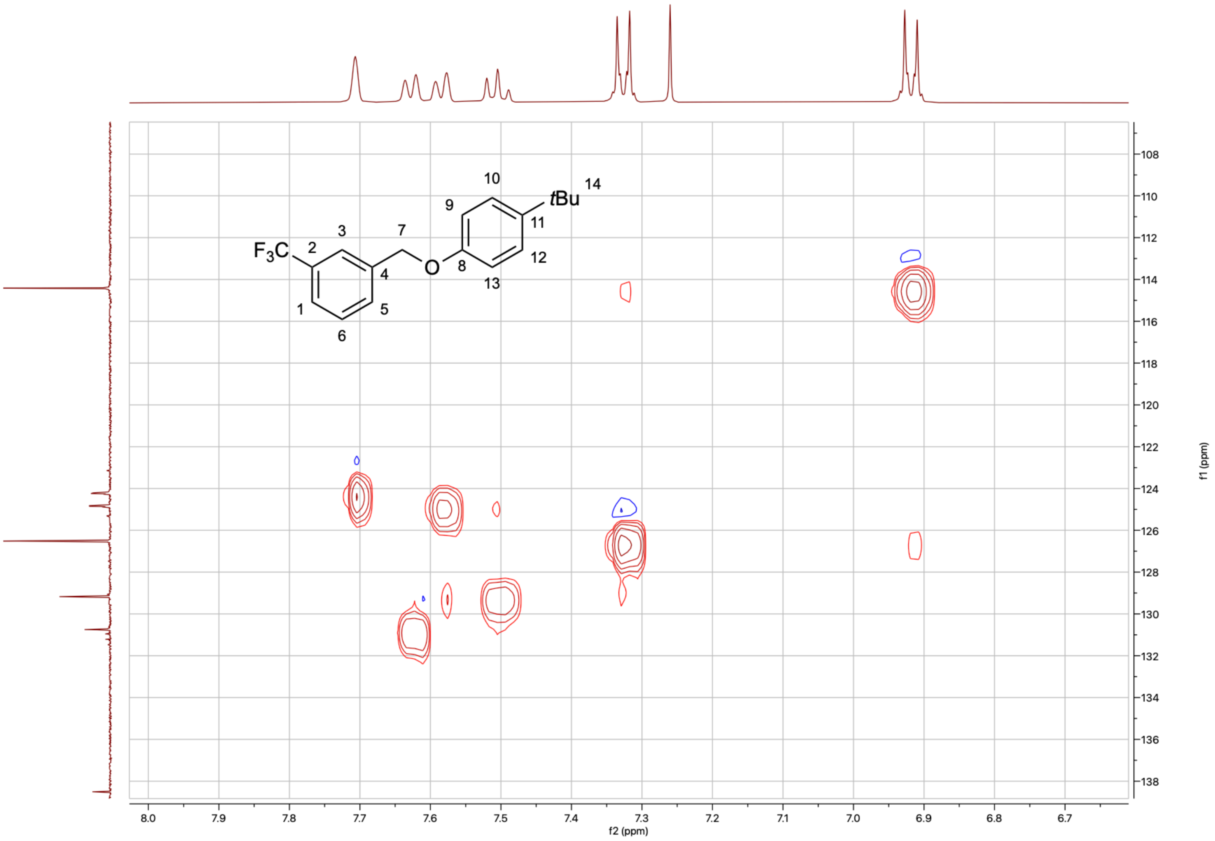

Figure S98: HMQC spectrum of **HP 12** in $CDCl_3$.

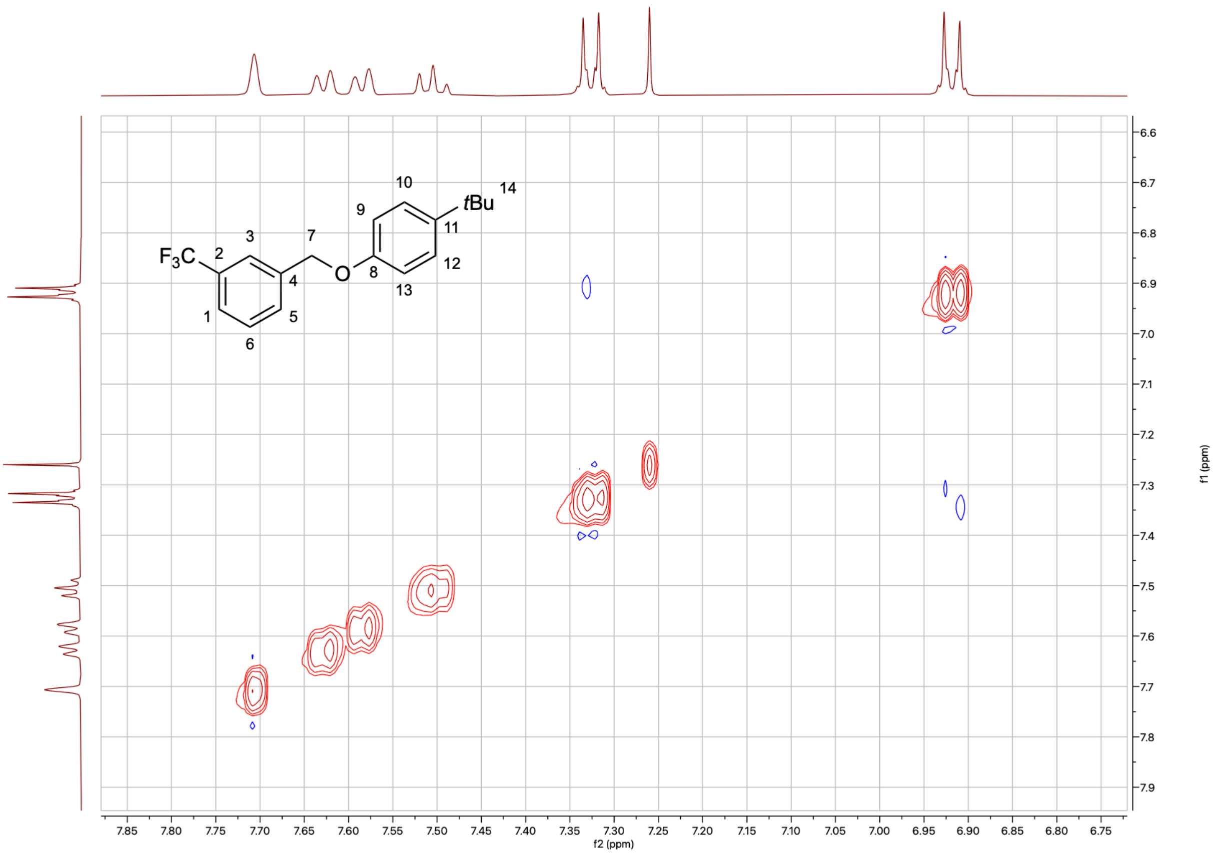

Figure S99: NOESY spectrum of **HP 12** in $CDCl_3$.
